# A systemic, multistakeholder participatory approach to research, innovation and action agenda setting for more integrated and effective prevention of HIV and other STIs: the case of Catalonia, Spain

**DOI:** 10.3389/fpubh.2026.1783030

**Published:** 2026-09-23

**Authors:** Rosina Malagrida, Carina A. C. M. Pittens, Marjolein B. M. Zweekhorst, Jacqueline E. W. Broerse

**Affiliations:** 1 Living Lab for Health, IrsiCaixa Research Institute, IGTP, Badalona, Spain; 2 Athena Institute, Vrije Universiteit Amsterdam, Amsterdam, Netherlands

**Keywords:** integrated healthcare, multistakeholder participation, participatory agenda setting, prevention of sexually transmitted infections, promotion of affective-sexual health, systemic approach

## Abstract

**Introduction:**

It is increasingly acknowledged that transformative change is needed to effectively address the complex and persistent health challenges facing society today. Realizing transformative change requires multistakeholder processes that co-define and implement research and innovation (R&I) priorities and actions that better respond to the complexity of these challenges in a contextualized manner. However, there is limited insight into the operationalization of participatory systems approaches for identifying the systemic elements of these complex challenges and for defining and implementing R&I topics and action agendas to address them. In this paper, we therefore aimed to analyze the results of a participatory agenda-setting process on the prevention of HIV and other sexually transmitted infections (STIs) that used a systems approach and took place in Catalonia, Spain.

**Methods:**

For the agenda-setting process, the innovative System-oriented Dialogue Model (SDM) was used, focusing on the exploration, consultation and integration phases of the model. It involved a wide variety of participants (*n* = 687), including secondary school students (*n* = 660) and professionals (*n* = 27), i.e., researchers, healthcare professionals, civil society organization representatives, communicators and policymakers. Twenty focus groups were organized to identify (1) a broad range of systemic elements of the problem (i.e., needs and barriers) and (2) solutions (i.e., R&I topics and actions) for the prevention of HIV and other STIs, and a dialog meeting took place to validate the final agenda.

**Results:**

The participants identified a wide range of needs, barriers and solutions covering multiple relevant R&I topics and actions in relation to prevention strategies, healthcare access, community-based activities and policy development. The results were compiled in a final agenda structured around eight missions. Two were related to health services and products: “access to prevention, diagnosis, treatment and services” and “development of biomedical tools for prevention and diagnosis.” Others were related to systemic elements such as “assessment of stakeholders’ needs and interventions,” “resources for R&I, prevention, interventions and health inequalities” and “governance, regulation and human rights.”

**Discussion:**

Using the SDM phases exploration, consultation and integration, we generated a shared agenda for prevention of HIV and other STIs, covering multiple areas with key systemic elements within (underserved) R&I topics and actions. To guarantee subsequent implementation of the resulting agenda with sustained participatory, systems approaches, we recommend implementing the next phases of the SDM with participatory research with specific attention for governance, accountability and funding constraints. This may require mixed-methods research involving the stakeholders that may already agree on the ownership of the missions and taking into account key quantitative studies on benchmarks, realistic implementation pathways and opportunity costs.

## 1 Introduction

The health system faces many complex challenges, such as providing good quality care to an increasing population with a higher life expectancy and more complex care demands, while budgets become more restricted (1). The supply-driven research and innovation (R&I) advancements that defined the health system’s historic successes are unlikely to be able to sufficiently address these current challenges (1). One reason for this is that current R&I advances do not adequately respond to the real-world needs and realities of healthcare providers and patients. R&I are still mostly focused on drug treatments involving biomedical and clinical disciplines, while the needs of healthcare providers and patients often focus on other research areas and require other (trans)disciplinary perspectives (2–5).

Researchers and policymakers increasingly acknowledge that, to enable R&I to effectively address the complex and persistent health challenges of today, the R&I process needs to change (6, 7). To this end, many governments, institutions and communities around the globe are opening up strategic conversations on R&I to include a variety of stakeholders. This paradigm change is being supported by frameworks such as “Responsible Research and Innovation”, “Open Science and Innovation”, “Transdisciplinary Research” or “Participatory Action Research”, which are inclusive, multi-stakeholder initiatives calling for R&I processes and outcomes that better respond to the different stakeholders’ needs and expectations (8–10).

Previous studies have shown how multiple stakeholders, such as patients, caregivers, health professionals, and researchers and innovators in different disciplines, can be involved in co-defining R&I priorities, thereby taking into account a broader spectrum of perspectives (11–16). These participatory agenda-setting initiatives show that a multistakeholder approach may identify unmet needs and result in new original research questions and innovative solutions (12). However, studies evaluating what happens after such initiatives show that it is often business as usual, with little change in research practices and topics addressed (12, 17, 18).

In part, this lack of change occurs because the participatory R&I agenda-setting process often targets complex challenges for which there is a lack of consensus on both the definition of the problem and the solutions. In addition, researchers do not consider the new R&I topics brought up by other stakeholders as a priority, and therefore, “elements of the problem situation relevant to other actors are (often) overlooked or denied” (19). Furthermore, researchers who could implement these new R&I topics may not have been involved in the process and are thus not engaged in implementing the emerging R&I topics.

Another issue is that the resulting R&I agendas often lack a deep understanding of the complex problem situation and therefore also lack (R&I) topics and actions related to key systemic elements of the context. These systemic elements refer to (a) current routines and ways of working, (b) rules, policies, guidelines, power structures and distribution of resources, and (c) mental and cultural models (20, 21). Therefore, scholars suggest that systemic approaches are needed that engage a wider variety of stakeholders in agenda-setting processes, including those who can contribute to better describing the problem situation and identifying key systemic elements (17, 18) as well as those who may be able to implement the solutions needed to address them. These may or may not be the same stakeholders for a given problem situation.

The aim of this study was to analyze the results of a participatory agenda-setting project on the prevention of HIV and other sexually transmitted infections (STIs) using a systems approach in the context of Catalonia, Spain. To this end, we developed the System-oriented Dialogue Model and applied it for the first time in this study. The following research questions were addressed: (1) what are the problems (needs, barriers) and solutions (R&I topics and actions) regarding the prevention of HIV and other STIs identified by the different stakeholders, and (2) to what extent do the identified solutions cover relevant R&I topics as well as key systemic elements related to HIV and other STI prevention, when compared with more “conventional” R&I agendas, within and beyond the health system.

## 2 Methods

The challenge of the prevention of HIV and other STIs was identified in 2015, when the first author initiated a wide consultation in which the health needs of 1,000 secondary school students in Catalonia, Spain, were identified and analyzed. These students determined that ‘HIV/AIDS’ was their second most important health-related concern after ‘mental health and well-being’. The topic was taken up in a project called Co-ResponsHIVility (2017–2019). The agenda-setting process included a wide diversity of stakeholders – professionals from various research fields, healthcare professionals, civil society organisation (CSOs) representatives, communicators and policy makers, as well as secondary school students from schools in Catalonia.

### 2.1 Research approach

To develop an agenda on HIV and other STI prevention using a systems approach, we designed the System-oriented Dialogue Model (SDM) (22). It combines the participatory Dialogue Model (12) with a System Analysis Tool (23). This study was the very first implementation of the SDM (see Box 1 and Figure 1). Phase 2 specifically includes the use of the System Analysis Tool (23), which classifies the barriers to system transformation according to the different characteristics of the system (see Box 2). We report here on three phases: exploration, consultation and integration. Based on the agenda, five research pro-jects and the necessary dissemination actions were subsequently selected and implemented to respond to four of the priorities as part of Phases 5 and 6. Next, a new iteration of the SDM was implemented (from 2020 to 2023). These later phases and the new iteration are beyond the scope of this article.

**Figure 1:**
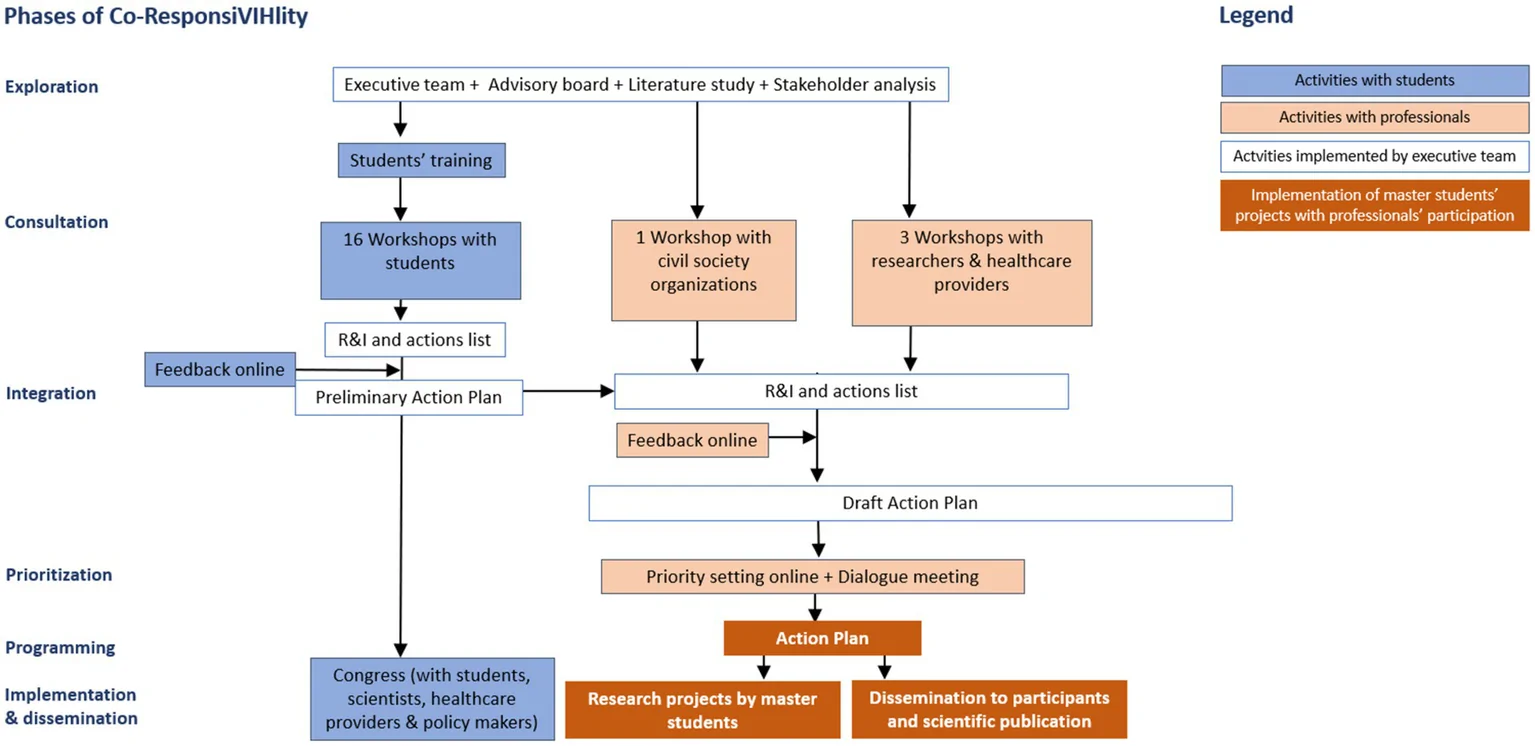
Scheme of the actions conducted within each phase of the SDM.

BOX 1 **System-oriented Dialogue Model (SDM)**

Although the present study reports the first implementation of the SDM, a separate publication describing the model was previously published following its second iteration by Malagrida et al. (24).

The SDM combines the Dialogue Model (DM) with a System Analysis Tool (23). The DM is a tested methodology for patient involvement that is radically dialogical in its orientation and offers clear prescriptive guidelines on how to consult and integrate the issues of various stakeholders, including patients, in research agenda setting (12). The DM has six underlying key principles that guide a meaningful participatory process: (1) active engagement of patients, (2) conducive social conditions, (3) respect for patients’ experiential knowledge, (4) ongoing dialogue, (5) emergent and flexible design and (6) neutral process facilitation. It engages patients as partners with health researchers and other stakeholders rather than just consulting them through focus groups or committees.

This model facilitates mutual learning processes through ongoing dialogues, which aim to include all stakeholders’ perspectives in ways that have real impact on the agenda (12, 24). Over the years, the DM has been applied and validated within a wide range of case studies of pathologies and disabilities, such as burns (25), visual impairments (26), spinal cord injuries (27), asthma (28), Parkinson’s disease, dental health (29), and COVID-19 prevention (22). The DM is structured in six phases:

1. *Exploration*: Via document analysis and exploratory interviews, first insights are gained into the needs of the process, the scope of the agenda and the needs, barriers and ideas/solutions of the different stakeholder groups. Supportive conditions for meaningful involvement are created.
2. *Consultation*: Stakeholder groups are consulted separately to establish an agenda from each group’s perspective. Consultation often occurs via focus groups or interviews. During this phase, the SDM includes a system analysis.
3. *Prioritization*: Stakeholder groups assign value to their agenda topics and rank them in order of importance, often through questionnaires or Delphi studies.
4. *Integration*: The individual stakeholder group agendas are integrated into one agenda. Integration is often realized via dialogue meetings, in which mutual learning and shared ownership are essential conditions.
5. *Programming*: The integrated agenda is translated into an R&I program or action plan.
6. *Implementation & dissemination*: Implementation of the program/action plan, including a call for proposals, matching of subjects and departments, and funding. Actors (including patients) implement and take action, monitor progress and evaluate results. The implementation processes and results are disseminated.

### 2.2 Data collection

#### 2.2.1 Phase 1: exploration phase

This first phase aimed to (1) establish an advisory board and executive team, (2) identify relevant areas in the field of HIV/AIDS prevention, (3) identify and recruit participants, (4) provide facilitation training for teachers and (5) prepare students for their participation. These steps are described below:

1. An *advisory board* was formed to act at a strategic decision-making level to provide advice on scope, recruitment and interpretation of the data. Their role was explicitly defined as consultative, with final decision-making authority retained by the research team. The board was composed of members with diverse expertise to ensure a plurality of perspectives. It included the director and a researcher/healthcare provider from the IrsiCaixa Research Institute; members of IrsiCaixa’s Community Advisory Board, which included CSO representatives and policy makers working on public health; the director of a CSO not participating on the IrsiCaixa board; and the director of CEEISCAT, an institute of public health research. An *executive team* was also formed with the first author, a research assistant and three master students who helped with dissemination and monitoring activities.
2. A literature review was conducted on HIV/AIDS prevention to *identify relevant research areas* so that we could be sure to invite experts from each area who would enrich the analysis with broader perspectives. The resulting list of areas was validated by the advisory board, which requested that the scope of the project be expanded to include the prevention of other STIs as well (see Supplementary File 1 for the outcomes of this literature review).
3. To ensure stakeholder diversity, for each research area, *stakeholders were identified and recruited* through purposive sampling. Researchers, healthcare providers, CSO representatives, policy makers, educators and communicators who had at least 5 years of experience in each of the areas and who were currently active in their field were contacted through informal conversations and emails. *High school students were recruited* through purposive sampling via motivation letters sent to schools that were part of a community of practice (CoP) for innovative R&I implementation initiatives within education. All teachers in the CoP had previously received training on Responsible Research and Innovation (30, 31) and innovative teaching techniques and were selected based on their motivation to engage. The resulting participants list was discussed with the advisory board for validation.
4. Secondary school teachers (*n* = 13) were *trained to facilitate the consultation with the support of semi-structured facilitation guidelines and observation templates to ensure consistency* (see Supplementary File 2). To further ensure quality and resolve any additional questions or concerns, teachers attended monthly meetings during the consultation process and were provided with backstopping services, including access to researchers for further support.
5. As students were the only stakeholder group without expert knowledge in the field of HIV/AIDS and STIs, teachers were provided with educational guidelines (see Supplementary File 3) and *facilitated activities* to empower students to give input during the consultation.

The following participants were excluded: professional participants who did not provide informed consent and secondary school students in focus groups whose teachers did not complete the data collection phases.

#### 2.2.2 Phase 2: consultation phase

In this phase, 20 focus groups (FGs) were organized with students, key professionals in the different research areas and professionals from CSOs to identify (1) needs for and barriers to HIV and other STIs prevention and (2) solutions – (new) R&I topics and actions. To stimulate identification of key systemic elements during student FGs, reflection on different characteristics of the system affected by HIV and other STIs was facilitated using the System Analysis Tool, which was adapted for this target audience (23) (see Box 2). Similar reflections were also introduced at the beginning of the FGs with professionals.

BOX 2 **System analysis tool’s characteristics adapted for discussions during FGs with students**

- *Access to knowledge and research*: on HIV and other STIs, including access to knowledge disseminationand access for stakeholders to participate in research processes
- *Physical or virtual environment*: including school facilities and facilities for night, sports, leisure and healthcare activities and virtual spaces such as computers, tablets and social networks
- *Legislation and regulation*: both inside and outside schools
- *Values, culture and climate*: in schools and within families, affecting relationships with teachers and among students and families
- *Spaces for interaction among actors*: such as teachers, students, psychologists, CSOs or healthcare providers, and including both time and space for reflection and training
- *Funding model*: for research and innovation and for prevention interventions

The consultation phase took place first with the secondary students and then with the professionals. In total, 16 FGs with students were conducted in 16 different schools as part of an academic course between the 1st and 30th of April 2017. A total of 660 students participated (approximately 30 students per FG). The student FGs were facilitated by teachers in the CoP who also contributed to the data collection (as a form of citizen science). Out of the 16 FGs, teachers sent 13 observation schemes to the first author, resulting in three FGs being excluded. Between the 1st of October and the 30th of November 2017, four FGs were conducted with professionals from CSOs and researchers from different research areas (see Table 1). Three of these FGs were facilitated by the first author, and one FG (with CSOs) was facilitated by a CSO representative.

**Table 1:** Number of participants in the FGs of professionals in each research area.

Focus groups	Research areas	Number of professionals
FG 1	Behavioral and structural aspects	7 (4 researchers, 2 healthcare providers/researchers, 1 policy maker)
FG 2	Biomedical prevention and barrier methods	5 researchers
FG 3	Biomedical prevention and barrier methods	6 professionals from CSOs
FG 4	Information, education and communication	9 (1 policy maker, 5 researchers, 3 science communicators)
Total participants		27

In line with ethical procedures at that time, before their participation in the FGs, all professionals signed a written informed consent approved by the ethics committee of the Hospital Germans Trias I Pujol where IrsiCaixa is located. As no personal data were collected from the students, no specific consent procedures were required for their participation.

#### 2.2.3 Phase 3: integration phase

The FG results were gathered, color-coded to distinguish the inputs of students and different types of professionals and analyzed for similarities separately following a thematic analysis. This resulted in two preliminary agendas – from students and professionals. Students validated this agenda in May 2016. Subsequently these agendas were merged into one single thematic agenda without deleting any items from either. The resulting agenda was shared with the professionals who had participated in the FGs for their feedback and approval in December 2017. After several iterations and edits, the document was approved, resulting in an agenda on HIV and other STIs prevention from the perspective of the different stakeholders. The students could not participate in this round of iterations and editions, as it was not possible to contact them after their academic course had finished in June 2017. In their absence, the first author ensured that no student items were deleted and that all changes made to student items were recorded.

A dialogue meeting was organized in February 2018 with representatives from each FG of professionals to present and verify the results of the integration phase and to discuss disagreements; it included researchers and healthcare providers (*n* = 4), education professionals (*n* = 2) and CSO representatives (*n* = 3). The teachers and students were not present at this meeting, as the academic year had changed and it was difficult to contact them again. The first author acted as facilitator and minutes were taken by an assistant. The facilitator ensured that participants did not sideline students’ items. The resulting integrated agenda formed the input for the subsequent phases: priority setting, programming and implementation.

### 2.3 Data analysis

During the third phase, for the analysis of needs, barriers and solutions (i.e., R&I topics and actions), we performed reflexive thematic analysis, inductively coding the data (32). The first author conducted a process of iterative open and axial coding of the data from students and other stakeholders, comparing refining and clustering codes of needs and barriers, and solutions, separately. All these codes were considered, without any privilege for professionals’ inputs, to develop the themes. Subsequently, the themes were clustered according to the purpose that needed to be achieved; these clusters were named “Missions.” This resulted in an agenda comprising missions, each comprising several themes, which were in turn grounded in the underlying codes (needs, barriers and solutions). Next, a second thematic analysis was conducted with the integrated agenda, resulting in a final, more concise agenda that specifies the themes of R&I topics and actions within each “Mission.” Finally, a peer debriefing and independent verification were performed with the second and last authors of this paper. A detailed overview of the items, codes, themes and missions generated during the analysis is available in Supplementary File 4.

To analyze the relevance of involving a wide diversity of stakeholders, their perspectives on needs, barriers and solutions were compared, giving specific attention to (1) the overlap and differences between students and other stakeholder groups and (2) the variety of identified needs, barriers and solutions. We also analyzed whether the new agenda covered different R&I areas and key systemic elements by classifying the R&I topics and actions among the different research areas, described in Supplementary File 1, and the systemic characteristics of the System Analysis Tool. We then analyzed whether the resulting R&I topics went beyond those in the conventional R&I agenda by conducting a literature review. Beyond the focus of our research, this final list was aimed at facilitating progression towards the phases of prioritization, programming and implementation in new iterations of the SDM process.

## 3 Results

This section presents the results of three analyses: (1) the reflexive thematic analysis of needs, barriers and solutions, (2) the second thematic analysis leading to the final agenda and (3) the analysis of whether that agenda covers different R&I areas and key systemic elements across the different systemic characteristics of the System Analysis Tool and whether it extends beyond a conventional agenda. As the prioritization phase is not part of this study, we describe its methodology and results in Supplementary File 5.

### 3.1 Analysis of needs, barriers, and solutions

The needs, barriers and solutions that students and professionals identified for the prevention of HIV and other STIs were grouped into eight missions: (1) Resources for R&I, prevention interventions and health inequalities, (2) Governance, regulation and human rights, (3) Information, education and communication, (4) Access to prevention, diagnosis, treatment and services, (5) Psycho-social aspects related with culture, values, stigma and discrimination, (6) Use of psychotropic substances and night leisure, (7) Assessment of stakeholders’ needs and interventions and (8) Development and innovation of biomedical tools for prevention and diagnosis. The missions included a total of 65 themes (see Supplementary File 6).

#### 3.1.1 Analysis of needs and barriers

Needs and barriers were identified by professionals within all themes except for two themes that emerged exclusively from students’ contributions. Although professionals were fewer in number than students, they contributed to a larger number of themes, reflecting the breadth of knowledge and system-level perspective derived from their professional roles. While students contributed to 40 of the 65 themes identified, their perspectives showed considerable convergence with those of other stakeholder groups. Importantly, in the themes where students participated, their contributions extended beyond merely confirming existing priorities, enriching the themes with additional needs, barriers, and solutions and, in some cases, highlighting issues not raised by professionals. These contributions are illustrated in the mission-specific analyses below.

##### 3.1.1.1 Mission 1: resources for R&I, prevention interventions and health inequalities

This Mission revealed a perceived structural mismatch between the societal importance of prevention and R&I in this field and the resources allocated to support them. Professionals and students consistently framed insufficient funding as a fundamental barrier limiting the development, innovation, implementation and sustainability of prevention interventions and research activities. Two themes emerged as particularly salient.

The first is the “lack of funding for the implementation of (…) prevention interventions,” for which both students and professionals highlighted the need for more investment, with expressions such as “funding for prevention methods should be more accessible and affordable” (highlighted by students) or “the lack of resources for STIs and drugs” and “lack of economic and human resources” (highlighted by professionals).

The second is “the lack of resources for R&I” (all items mentioned by students) with expressions questioning investment policies and therefore highlighting the need to improve them (“how much has already been invested and why are there not more prevention methodologies?”) or others highlighting the need for more “resources for research” because “governments and private companies do not invest enough in research.”

Professionals and students also claimed the need for a new funding model that needs to be co-defined with a diversity of stakeholders. Professionals highlighted some systemic elements that a funding model should address, including early detection and control, social services, communication, education, inequalities and evaluation. Among these elements, students only mentioned the need for funding for communication.

The predominance of professional contributions within these themes may reflect their closer engagement with implementation challenges and service delivery contexts. By contrast, students’ contributions were largely concentrated on broader concerns regarding research and prevention funding, with communication being the only specific funding priority raised by both groups.

##### 3.1.1.2 Mission 2: governance, regulation and human rights

This Mission included 13 themes of needs and barriers related to governance, regulation and human rights within prevention systems. The many themes mentioned reveal a perceived need to rethink how decisions are made, whose perspectives are included and how human rights considerations are integrated into prevention policies and practices.

The most prominent theme “Co-responsibility model in decision-making (…)” was raised primarily by professionals, with statements such as “decision-making is not based on a consensus” and the need for “a new model of interaction between different stakeholders, including those in the biomedical community and those in the third sector (i.e., non-governmental and non-profit-making organizations or associations), that facilitates evolving towards a model of shared responsibility.” Some professionals also highlighted that this new model of interaction should include civil society: “the model does not respond to the current transformative change in the relational models between civil society, the community and other stakeholders” (where “the community” refers to the professionals working in the third sector). Students also showed broad agreement on the need for more inclusive decision-making processes with items such as: “need to arrive at a consensus among different values and opinions in order to define a response that can be addressed by governments and to have strategies based on (…) collaborative efforts.” These accounts suggest a perception that current governance arrangements remain fragmented and hierarchical, limiting the capacity to address complex prevention challenges that require coordinated action within and across sectors.

The second most prominent theme was the “need to consider sexual and reproductive rights in a transversal way in all prevention actions.” Concerns were raised that legal and institutional frameworks may not fully reflect sexual and reproductive rights. Items in this theme were mainly expressed by students – for example, “more laws to prevent discrimination against people living with HIV, both as professionals and as citizens” and “old-fashioned laws based on religious beliefs.” Other items highlighted by professionals, such as: “need for compliance of rights to sexual and reproductive health,” reinforced the importance of better considering human rights in prevention policies.

The less frequently mentioned themes further elaborated on the need to clarify and disseminate the rights of patients, healthcare professionals and volunteers in clinical research, suggesting that the sexual and reproductive rights, on the one hand, and the biomedical rights, on the other hand, are not being respected enough in these different domains. Participants also emphasized the need for better balance and coordination between the biomedical and the socio-educational model of prevention for STIs, and for stronger coordination with other health promotion initiatives. These concerns point to a perceived disconnect between different prevention paradigms and sectors, indicating that more coherent and integrated approaches may be needed to address the multifaceted nature of (sexual) health challenges.

##### 3.1.1.3 Mission 3: information, education and communication

This Mission included 13 themes of needs and barriers, four of which had higher numbers of items. Across these themes, participants consistently framed the challenge of HIV and STI prevention as not merely a lack of information, but also as a limitation of the current educational and communication model.

The most prominent theme concerned the “need to increase the quantity and quality of information and education interventions (…),” indicating dissatisfaction with conventional approaches. Participants suggested the use of diversity of channels; innovative approaches to bring research and education closer together; strategies to better target specific audiences within and beyond high-risk groups with personalized approaches; other strategies targeting more general audiences; and focusing less on preventing risk and more on promoting pleasure. Participants perceived current approaches as insufficiently responsive to population diversity and too centered on fear, advocating instead for approaches that engage individuals through positive and meaningful narratives about affective-sexual health. These included items from students, such as “the interventions are not effective and there are rarely opportunities to engage students with the challenge,” “lack of knowledge on how to design effective strategies,” “difficulties for adolescents to access the research and researchers” or need for “opportunities for students performing research projects to collaborate within researchers in order to increase their impact.” The items from professionals also highlighted similar aspects, such as “need to design more personalized interventions,” “need to also address groups that are not at high risk” or “need to innovate in communication channels (i.e., stop using leaflets).”

The themes with the second and third highest numbers of items concerned, respectively, “lack of affective-sexual education in formal education; currently education takes a very biological approach” and lack of “non-formal education targeting different stakeholders, such as professionals (currently this education also has a strong focus on biological aspects) and citizens.” Students highlighted items on formal education, such as “all schools should facilitate education activities on HIV and other STIs (also for families)” and “lack of sexual literacy among teachers,” and items on non-formal education, such as “lack of services available for young students” or “lack of awareness campaigns.” Professionals also focused on formal and non-formal education, with items such as “lack of affective-sexual education,” “sexual education: who is in charge? where? how?” and “education models lack participatory methodologies” (regarding formal education) and “lack of training for healthcare (…)” and “(…) for education professionals” or “lack of health literacy” (regarding non-formal education). The prominence of non-formal education shows that schools are not perceived as the sole or sufficient context for sexual health learning. Instead, participants envisioned a broader educational ecosystem involving citizens and other stakeholders with participatory approaches and structural support. Together, these themes reveal a demand for more holistic, continuous and community-based approaches to sexual health education.

Finally, the theme with the fourth high number of items concerned “the low knowledge on HIV and STIs as well as on transmission routes, preventive methods (…), the consequences of infections and the need to take care of one’s health (…).” Most of the items were expressed by students, such as “lack of knowledge on prevention strategies.” Some focused on specific knowledge needs, such as: “do you always get the infection when you maintain a sexual relationship with a person living with HIV?,” “are there post-infection treatments?” or “if I have HIV, what should I do?” This theme also included items from professionals, such as “lack of knowledge on HIV and other STIs and their consequences,” “lack of access to scientific literature” and “lack of knowledge among citizens and decision-makers.” This indicates that participants understood knowledge deficits not simply as a public health issue but as a systemic issue affecting multiple sectors and mainly caused by problems of accessibility and relevance of existing information resources.

The remaining themes highlighted other barriers of the current information, communication and educational model, such as the lack of public awareness on the need to take care of one’s health, the misconceptions about HIV and other STIs acting as a barrier to getting tested, the low level of social reflection on sexuality, and the difficulty in transferring knowledge from the scientific community to the media and community agents and in distinguishing between rigorous and non-rigorous information. Taken together, the findings indicate that participants viewed improving HIV and STI prevention as requiring the transformation of how knowledge is produced, communicated and integrated into different sectors.

##### 3.1.1.4 Mission 4: access to prevention, diagnosis, treatment and services

This Mission included 10 themes of needs and barriers of which three were most prominent: “lack of universal access and use of diagnostic and prevention methods (…),” “need to strengthen the promotion of HIV testing” and “need to promote alternative methods to condoms for certain groups, such as pre-exposure prophylaxis (PrEP), in combination with non-pharmaceutical interventions.” Together, these themes suggest that participants viewed HIV and STI prevention not only as a matter of individual responsibility but also as dependent on equitable access to preventive resources and healthcare services combined with non-pharmaceutical interventions, with specific attention to promoting HIV testing and alternative methods to condoms for certain groups.

Students proposed items such as the “need to improve accessibility to condoms, especially within spaces where adolescents and other social communities with behavioral risk meet,” “need to regulate to ensure that key communities get tested” or “extension of pre- and post-exposure prophylaxis to make them more accessible.” Professionals similarly highlighted the “difficulties for young people to access prevention methods” and the broader “lack of universal accessibility.” These contributions indicate concerns that existing prevention tools are not reaching all population groups equally, particularly younger and more vulnerable populations.

The opportunities and challenges associated with PrEP emerged as a recurring topic. Although scientific evidence has demonstrated its effectiveness, professionals highlighted a lack of clarity on “how to determine the target population and to make sure it is accessible for the most vulnerable communities” and “how to combine it with non-pharmaceutical interventions,” as “it does not cover other STIs” and “it has a high cost” that will not be easy to cover due to “the lack of social support to cover the expenses.” This suggests that participants viewed biomedical innovations as useful but insufficient on their own, requiring systemic approaches and evidence of their cost-effectiveness to achieve the needed public support. Professionals also highlighted the “need to study if PrEP could be a facilitator to accessing the healthcare system,” pointing to its potential role beyond STI prevention.

This Mission also included a theme on health inequalities at both local and international levels. Students suggested items highlighting causes of health inequalities such as “health literacy and self-security,” “social and intra-family violence and economic difficulties” and “massive immigration of people with low incomes.” Professionals referred to “the lack of access to prevention for vulnerable populations” or “the need to intervene in certain communities, such as in institutional children’s shelters.” These accounts suggest that participants understood vulnerability to HIV and other STIs as shaped by broader social and structural determinants, rather than solely by individual behaviors.

Other themes with lower numbers of items highlighted “the high cost of preventive methods,” the “lack of perspective about social equity and protection factors in research projects and prevention interventions,” the “low use of prevention methods,” the “rejection or lack of adherence to antiretroviral treatment and its impact on transmission risk” the “need to improve access through sexual networks that can facilitate the spread of infections but can also be a good tool for prevention” and the lack of “spaces for face-to-face and online interactions between different stakeholders (i.e., psychologists, teachers, families, young people, citizens from different social communities and communities with high risk (…)).” Together, these themes suggest that participants viewed effective prevention as requiring accessible and integrated strategies that combine pharmaceutical and non-pharmaceutical interventions, are supported by spaces for community engagement and interaction, and explicitly address issues of equity.

##### 3.1.1.5 Mission 5: psycho-social aspects related with culture, values, stigma and discrimination

This Mission included 12 themes of needs and barriers regarding culture, values, stigma and discrimination. The theme with the highest number of items was “the fight against stigma and discrimination is not part of the prevention model in a transversal way.” Participants referred to discrimination according to sexual orientation or LGTBI-phobia, serophobia, social inequalities, accessibility to the health system, gender inequalities and masculinities. Together, these contributions suggest that stigma was understood not as an isolated problem affecting certain population groups, but as a cross-cutting factor that shapes multiple dimensions of prevention.

Another theme with a high number of items referred to “the high level of stigma and discrimination against people according to their sexual orientation and serological status makes prevention difficult.” Students highlighted “prejudices against people living with HIV” and the “exclusion of communities that people consider to follow risk practices such as men who have sex with men.” Many students emphasized the “need to make sure HIV is not frightening” and claimed that interventions should focus on ending stigma: “considering HIV infection as a normal health burden will not mean that prevention will not be a priority, but on the contrary, it will improve prevention because people will consider HIV as something they might face” while “stigmatized people will avoid talking about the infection and perhaps even following the treatment, and this situation will affect prevention.” Professionals raised similar concerns, referring to “discrimination,” “homophobia,” “serophobia,” “stigma regarding sexual orientation” and “gender inequalities.” As a response to these barriers, professionals highlighted “the need for more structural interventions,” and they argued that beyond the work of civil society organizations “there should also be other strategies to abolish stigma.” Students also contributed with similar responses. These accounts suggest that participants viewed stigma as a priority challenge that should be addressed with a collaborative effort among different sectors because it is not only a social injustice but also a barrier that undermines prevention and treatment adherence.

A related theme with a high number of items focused on the consequences of stigma, such as discrimination, prejudices, taboos, fears and shame. Students highlighted items such as the “shame when buying condoms” and “the need to focus interventions on the shame of talking about sexuality and asking questions,” while professionals also emphasized that sexuality is a “taboo” and highlighted how “stigma influences accessibility to prevention facilities for young people.” These findings indicate that the consequences of stigma operate through everyday social interactions, limiting open discussions about sexuality and hindering both engagement in preventive behaviors and access to prevention tools and services.

Another theme with a similar number of items focused on “the lack of a gender perspective in research projects and interventions.” Students highlighted barriers such as “although gender equality is improving, women still need to have more decision-making power in sexual relations” and “inequalities between sexes and predetermined roles.” They called for more “interventions in schools to work on equality and non-discrimination.” Similarly, professionals stressed the “lack of gender perspective in research projects” and “(…) in prevention interventions,” and they stressed the “need to consider prevention of sexual violence in STI prevention interventions” and the “lack of knowledge about sexual, functional and cultural diversity and its incorporation into research projects and prevention interventions.” They also pointed to the “difficult balance between promoting pleasure and prevention while dealing with stigma” and “the paradox between trivialization and stigmatization whereby prevention may be trivialized, but once a person is addicted to drugs (such as those being used in chemsex) or infected, they are discriminated against and stigmatized.” Together, these themes suggest that participants viewed gender and diversity perspectives as essential components of effective prevention rather than complementary considerations.

Finally, participants reflected on the relationships between stigma, culture and values. Some themes focused on how “stigma can influence changes in values and how sexuality is conceived” and how religions may shape attitudes towards sexuality: “rejection of people who get infected, they are considered guilty because of the values religions associate with certain sexual behaviors.” However, this religious influence coexists with another theme that refers to the “paradigm shift in sexuality regarding culture and values that influences prevention” with items expressed mainly by professionals such as “new trends in sexual relationships” and an “increase of sexual relationships: with more diversity of relationships and combined with drugs.” Some professionals stressed the importance of “responsibility as a key value for prevention” within a theme on “the lack of training on values and how they affect informed and reflective decision-making for prevention” and suggested there is a need “to increase education about values” and “to promote self-responsibility.” These themes suggest that participants viewed prevention as embedded within broader cultural norms, values and social transformations that can both facilitate and hinder HIV and STI prevention efforts, underscoring the importance of ethical education in supporting informed and reflective decision-making.

##### 3.1.1.6 Mission 6: use of psychotropic substances and night leisure

The three themes within this Mission focused on the relationship between night leisure, drug consumption and sexual health risks. Together, these themes reveal how drug consumption is not viewed merely as an individual behavior, but as a phenomenon embedded in broader social norms, leisure cultures, and peer dynamics that shape young people’s decision-making and opportunities for prevention.

The theme with the most items, stressed by both professionals and students, focused on the barrier caused by the “increase in the perception of a nightlife model associated with sex and group sexual practices and with the use of drugs (i.e., chemsex).” Within this theme, professionals expressed problems regarding the increased risk for HIV and other STIs because of “the consumption of legal and illegal drugs” and the “difficulties to respond to new emerging problems such as chemsex.”As a response to this barrier, this theme also included needs stressed by students such as the promotion of “critical thinking among young people and to take an active role to show others that different habits are better and possible” and “to raise awareness about the collateral effects of alcohol and drugs.” Therefore, both groups of participants framed this trend as an important barrier to HIV and STI prevention because it creates contexts in which risk-taking behaviors may become more common or socially reinforced.

The second most prominent theme focused on the “controversy about how to regulate accessibility to alcohol and other drugs.” Students presented different and sometimes opposing perspectives, revealing tensions between restrictive and harm-reduction approaches. Some participants advocated stronger regulatory measures, calling for policies “to implement policies that bring barriers to accessing drugs, especially alcohol, as (…) it is widely accepted at the social level,” “to increase control on accessibility to drugs.” These statements indicate a perception that the widespread social acceptance of alcohol, despite its potential health consequences, represents a challenge for prevention efforts. In contrast, other students proposed “to make drugs legal in order to be able to improve controls regarding safety, economic and social aspects,” reflecting a perspective that prioritizes regulation and oversight over prohibition. The coexistence of these viewpoints illustrates the diversity in public attitudes toward the regulation of substance use and suggests that young people do not necessarily perceive drug-related harms as solely determined by availability, but also by the broader policy frameworks governing consumption, safety, and social norms.

The third most prominent theme focused on the role of social pressure in decision-making, limiting the uptake of prevention measures. Students emphasized that “the main obstacle of adolescents (to implement prevention measures) is social pressure, to avoid losing support from peers.” This theme highlights the role of interpersonal dynamics and fears of exclusion in shaping decision-making and engagement with prevention measures within nightlife settings. Students described how dominant social norms, peer-group expectations, and individual needs for belonging, acceptance, and a shared group identity can make it difficult for adolescents to adopt responsible behaviors.

The findings suggest that prevention interventions should move beyond an exclusive focus on individual knowledge and attitudes and instead address these interpersonal dynamics. Strengthening skills related to autonomy, resistance to peer pressure, and collective norm change may therefore be essential components of effective prevention strategies.

Overall, the themes identified in this Mission indicate that effective responses will require not only raising awareness about the health risks associated with alcohol use, drug consumption, and sexual behaviors, but also addressing the social and cultural environments that contribute to the normalization of unhealthy practices and shape young people’s choices.

##### 3.1.1.7 Mission 7: assessment of stakeholders’ needs and interventions

This Mission highlighted a perceived gap not only in measuring effectiveness of interventions but also in understanding their potentially unintended consequences – a concept considered essential for ensuring safe and responsible implementation.

The theme with the highest number of items focused on the “lack of rigorous evaluation methods that include the analysis of iatrogenesis [adverse effects and risks associated with an intervention].” An example was mentioned where this lack of assessment of iatrogenesis was key: “in studies to detect contacts, where the stigma and stress generated is not being analyzed.” A second major theme identified referred to the “lack of rigorous evaluation (…) to design interventions with more effective methods and channels and to identify the actors to involve.” This concern points to the need for more robust assessments capable of informing intervention design with stakeholder engagement strategies. Professionals and students gave several specificities for this lack of evaluation, such as “lack of evaluation of public policies,” “, “need for a systematic review of the evaluations performed in Catalonia” and “lack of resources for research on effectiveness of communication campaigns.” Collectively, these observations indicate that current evaluation frameworks are perceived as fragmented and insufficient for informing intervention design and capturing long-term outcomes, contextual factors, and potential adverse effects. They also highlight the resource constraints that may limit the development and implementation of more comprehensive and rigorous evaluation practices.

This Mission also included other themes with lower numbers of items that nevertheless highlighted important knowledge gaps. For instance, “the lack of needs assessments to design innovative solutions more aligned with the needs of different communities and more personalized.” This theme reflects concerns about the extent to which existing interventions are informed by the diversity of stakeholders’ experiences and needs. Overall, the findings suggest that all participants considered robust evaluation and systematic needs assessment as critical prerequisites for developing evidence-informed and context-sensitive interventions.

##### 3.1.1.8 Mission 8: development and innovation of biomedical tools for prevention and diagnosis

This Mission comprised three themes that focused on the development of biomedical tools aimed at strengthening the prevention and diagnosis of HIV and STIs. Although represented by a limited number of items, the themes highlighted a shared interest in expanding the range of available preventive tools and improving their effectiveness, accessibility, sustainability and equity.

Students expressed “the need to develop a preventive vaccine,” highlighting the perceived potential of biomedical innovation to complement existing prevention strategies and contribute to reducing the burden of HIV and STIs.

Professionals also highlighted the need for a prevention vaccine and identified additional areas requiring research and innovation. These included “the need for more research on microbicides or other new solutions that are more equitable and sustainable,” highlighting concerns about ensuring that future biomedical tools are accessible and adaptable to the needs of diverse populations. Professionals also stressed the need “o evaluate treatment as prevention to reduce HIV and STIs in communities with high prevalence, suggesting interest in generating stronger evidence on the potential preventive impact of treatment-based strategies in contexts where transmission rates remain elevated.

Overall, this Mission indicates a demand for continued investment in biomedical research and innovation, with particular attention to the development of effective, accessible, sustainable and equitable preventive technologies and the evaluation of treatment-based strategies for communities at high risk.

#### 3.1.2 Analysis of solutions

The solutions identified by students and professionals were present in all Missions and included both R&I topics and other solutions that may not require R&I. Most of the solutions were related to Missions 2, 3 and 4 on governance, funding and accessibility. However, some of the solutions classified under these Missions also showed interconnections with other Missions, revealing the interconnected nature of the challenges participants sought to address. For instance, a solution to improve interventions so that they better respond to the needs of the end users (Mission 7: Assessment of stakeholders’ needs and interventions) will require new forms of interconnections among stakeholders (Mission 2: Governance, regulation and human rights) and new approaches to better take into account the values and beliefs of the target populations (Mission 5: Psycho-social aspects (…)). This suggests that participants viewed effective prevention as dependent on coordinated changes across multiple domains rather than isolated actions. Consequently, classifying the solutions among the Missions facilitated the analysis but also forced fragmentation, resulting in a loss of information about the interconnections between the solutions across the Missions.

The students’ proposed solutions were highly diverse, spanning research needs, practices and policy-innovation measures. Research needs were sometimes expressed as questions, such as “can we investigate whether it is true that the use of condoms among young people is decreasing with the increased use of contraceptive pills?” (Mission 3: Access to research results and other sources of information, education and communication). Students also suggested actions, such as “facilitate information leaflets when selling contraceptive pills in pharmacies to inform about the prevention of STIs” (Mission 4: Access to prevention, diagnosis, treatment and services). Notably, students identified research needs that were complementary to other types of solutions and that could facilitate evidence for better decision-making during implementation. For instance, together with “political strategies to fund research,” they proposed a complementary question that may require research, such as “do we have enough funding for research on HIV and other STIs?” (Mission 1: Resources for R&I, prevention interventions and health inequalities). This pattern suggests that students understood research not as an end in itself but as a resource for informing action and evaluating whether existing responses are adequate.

Professionals proposed solutions within Missions that students mostly did not address; they also identified more and a broader range of solutions than students did. Most solutions were aimed at transforming the system of prevention towards a system of co-responsibility with shared decision-making among different stakeholders in order to address different areas, such as policy making, knowledge transfer, R&I priority setting, design and implementation of communication campaigns and design of methodologies to conduct needs assessments and address them with collaborative processes. This aim is visible in the following examples of proposed solutions: “how can we promote the transformation of the model of prevention towards a model of shared responsibility?” (biomedicine professionals, Mission 2: Governance, regulation and human rights); “how can we ensure that the stakeholders involved in prevention have access to information and can participate in the decision-making process on which messages to disseminate” (CSO professionals, Mission 2). Collectively, these proposals indicate a concern with strengthening governance arrangements and stakeholder engagement as prerequisites for improving outcomes across multiple areas of prevention.

Like the students, some professionals identified research needs that were complementary to the other types of solutions and that could facilitate evidence for better decision-making during implementation. For instance, professionals in communication and education proposed the solutions “promotion of personalized interventions” (Mission 3: Information, education and communication) and “research on the needs of different groups” (Mission 7: Assessment of stakeholders’ needs and interventions). Professionals also suggested complementary solutions affecting the shape of both R&I and actions. For instance, researchers suggested that “cultural and sexual diversity should be incorporated in prevention” (Mission 5: Psycho-social aspects (…)), while CSO representatives suggested a need for research on the “analysis of how diversity is being addressed in research and in prevention interventions.” Taken together, these proposals suggest that professionals also perceived the need to complement practices and policy innovation with research to support their design and ensure that interventions are responsive to cultural and sexual diversity.

### 3.2 Final integrated R&I and action agenda

The needs, barriers and solutions that students and professionals identified were integrated into a preliminary agenda for HIV and other STI prevention by the researchers. Professionals and master students had the opportunity to reflect on and edit the preliminary agenda online. In total, they made 42 comments, most of which included suggestions for editing and improving the agenda, such as changing homophobia to LGTBI-phobia and seroconversion to serologic status or moving themes from one Mission to another. In most cases, the final phrasing was adapted according to the suggestions. Six comments raised conflicts or disagreements. These were all focused on ethical issues, stigma and power relations, and they mainly came from the CSO representatives. The conflicts related to (1) culpability, present in some phrases such as “lessening of societal concern”, (2) stigmatization (i.e., through interventions only aimed at people living with HIV), (3) data protection and the right to confidentiality in healthcare (i.e., regarding the need to regulate health professionals’ rights and duties so they can balance patients’ right to confidentiality when faced with a transmission risk and address that risk in an integral way within the health system) and (4) implicit power relationships (i.e., regarding the need to empower patients, which was raised by some healthcare professionals). The conflicts were discussed and alternative suggestions were proposed; for example, the conflicts on culpability were addressed by editing the agenda to promote a model focused on shared responsibility. Furthermore, proposals that contributed to stigma or that did not follow ethical principles were eliminated and improvements on how power relations are addressed were made – healthcare professionals do not empower patients, but they offer facilitating elements for empowerment.

This preliminary agenda was then used in the priority-setting phase. During the dialogue meeting, participants agreed that the agenda clearly had added value:

*I think that this agenda shows that there are topics that are not being tackled or sufficiently covered. They are important topics that are not solved.* (Participant 2)

A few participants also expressed their concern about the conflicts that came up during the integration phase. To them, this confirmed the need to pay more attention to underserved needs, such as those related to stigma, in the agenda-implementation phase. Many stressed that more collaboration among community professionals and researchers would be needed for successful implementation. However, some noted the difficulties involved in bringing some stakeholders together to co-create R&I and actions, referring to barriers that would need to be addressed to move towards a model with shared responsibility. By the meeting’s end, participants agreed on which projects needed to be implemented.

Next, the second thematic analysis led to the final integrated agenda for the prevention of HIV and other STIs, which includes 18 recommendations for R&I topics and actions (related to prevention strategies, healthcare access, community-based activities and policy development) distributed among the eight Missions (see Table 2). In Table 2 we also present the research areas and the systemic characteristics of each solution, showing that all areas and characteristics are addressed. More details are provided in Supplementary Files 4, 6.

**Table 2:** Integrated agenda of R&I topics and actions for HIV and other STIs prevention.

R&I topics and actions	Research areas	System characteristics
Mission 1: New funding model and sufficient resources		
Better balance between biomedical and socio-educational models with more human, structural and economic resources for communication and educational interventions, for research and rigorous evaluation (qualitative and longitudinal), for early detection and control of STIs and for complementary services, such as social services, especially focused on vulnerable communities Redesign of the funding model for interventions using a participatory process that includes administration and the third sector	Evaluation of public policies Integrated healthcare	Funding model
Mission 2: New models of governance, regulation and promotion of human rights		
3. Raise awareness and facilitate open discussions on sexual and reproductive rights and other human rights to improve their coverage in all prevention actions and research projects, to avoid controversies regarding whether it is necessary to define new specific legislations for people living with STIs and to clarify when health professionals can bypass the right to confidentiality because of HIV transmission risk 4. Redesign the prevention model to make it more focused on health promotion rather than disease prevention, more integrated (i.e., with other health problems and healthcare services, with social services from different geographical areas) and more focused on ethics, human rights and social aspects, such as inequalities. The governance of the new model should be based on co-responsibility in decision-making (e.g., on R&I priorities, priority target groups and design and implementation of interventions) including the participation of diverse stakeholders (i.e., policy makers, healthcare professionals, researchers from different disciplines, social services and the social community), should use innovative relational dynamics with a special focus on dealing with power relations 5. Improve the doctor-patient relationship: the patient currently perceives the doctor as someone who is paternalistic, solves problems, does not empathize, blames them and does not understand the new paradigm of sexuality	Bioethics and law Education and communication Evaluation of behavior interventions Health inequalities Integrated healthcare	Access to knowledge and research Legislation and regulation Spaces for interaction among actors Values and culture
Mission 3: Equal access to information, education and communication		
6. Increase literacy on STIs with quantity and quality of information and with (non-)formal education interventions that use a salutogenesis approach, make use of different channels, facilitate reflection spaces, bring research and education closer together and target both different audiences with personalized approaches and the general public to increase: Knowledge on transmission routes, preventive methods (including alternative methods such as PrEP), the consequences of infections and the need to take care of one’s health (including but not limited to one’s sexual health, wrong information that acts as barriers to getting tested, and sexuality with a good balance between the perception of risk and the focus on pleasure) Competencies of researchers to increase application of results, of professionals to promote affective-sexual education, and of all stakeholders to distinguish between non-rigorous information and rigorous and contrasted information 7. Facilitate training for professionals on how to educate about affective-sexual aspects (currently, they mostly focus on biological aspects) 8. Offer communication and education interventions for people living with STIs on how to communicate during sexual relationships and how to avoid transmitting the infection, especially for high risk communities	Education and communication	Access to knowledge and research Spaces for interaction among actors
Mission 4: Equal access to prevention, diagnosis and treatment and services		
9. Strengthen and improve accessibility to diagnostic and prevention methods in combination with other interventions, both pharmaceutical and non-pharmaceutical (e.g., tests, condoms, PrEP and PEP*, treatment as prevention, syringes, a vaccine if it becomes effective). Accessibility should be improved by: (1) using personalized approaches for people at risk, (2) investing in research and innovation with initiatives such as facilitating spaces for face-to-face and online interactions between different actors (i.e., counselors, psychologists, teachers, families, young people, citizens from different social communities and communities at high risk, etc.), e.g., by using online tools, such as sexual networks, and (3) focusing on social equity and protection factors for certain vulnerable groups (vulnerability due to factors such as intra-family and institutional violence or disadvantaged socioeconomic situations) in Spain and in countries with low per capita incomes 10. Improve and strengthen programs targeted at people who reject or do not adherence to antiretroviral treatment, highlighting the impact on the risk of HIV transmission	Integrated healthcare Health inequalities Psychology of the perception of risk and behavior change	Spaces for interaction among actors Physical or virtual environment
Mission 5: Psycho-social aspects related to culture, values, stigma and discrimination		
11. Design and implement policies to ensure that all prevention research and interventions address the fight against stigma, discrimination**, violence and taboos, while balancing this with the promotion of pleasure, as it is key for effectiveness 12. Increase literacy about sexual, functional and cultural diversity and how to incorporate them in prevention research and interventions 13. Design and implement policies to ensure that all prevention research and interventions address cultural aspects, values (e.g., when people get infected, it is their own fault due to their immoral behavior) and the current paradigm shift, including how these values are conditioned by stigma and religions and how they influence prevention plans, conceptions of sexuality and discrimination against people for sexual, cultural and functional diversities 14. Offer training in values involved in informed and thoughtful decision-making for the prevention of HIV and other STIs	Stigma Diversity Education and communication Medical sociology and anthropology Bioethics	Values, culture and climate Legislation and regulation Access to knowledge and research
Mission 6: Zero use of psychotropic substances and night leisure		
15. Promote a participatory process to reflect on nightlife leisure activities and the increased perception of their association with sex, group sexual practices and drug use, including chemsex, the controversy surrounding the regulation of access to alcohol and other drugs, and the group pressure that makes it difficult to make informed and reflective decisions in this leisure environment	Drugs and alcohol Psychology of perception of risk	Physical and virtual environment
Mission 7: Assessment of stakeholders’ needs and interventions		
16. Facilitate resources for needs assessments and evaluations of interventions that demonstrate changes in habits and that improve their design with innovative methods and channels and with more personalization and alignment with the needs of different communities 17. Facilitate access to rigorous and participatory evaluation methods that include the analysis of iatrogenesis, among other aspects, and that also facilitate the identification of stakeholders to involve in co-designing interventions	Integrated healthcare Education	Funding model Access to knowledge and research Spaces for interaction among actors
Mission 8: Development and innovation of biomedical tools for prevention and diagnosis		
18. Invest in R&I to develop vaccines, microbicides and other pharmaceutical treatments to reduce STIs in the community	Biomedical prevention and barrier methods	Funding model

*PrEP (Pre-Exposure Prophylaxis) and PEP (Post-Exposure Prophylaxis) are drug treatments to prevent HIV.

**According to sexual orientation or LGTBI-phobia, serophobia, social inequalities, accessibility to the health system, gender inequalities and masculinities.

Finally, a literature review was conducted to assess whether the R&I topics in the final agenda include topics beyond those in the conventional (implicit) research agenda and therefore contribute to redressing “the research agenda bias in clinical research” as described by Estany et al. (4), who analyzed the percentages of papers indexed in Web of Science on the specific topics of HIV/AIDS. They concluded that most publications about HIV/AIDS are produced in Clinical medicine (31.6%) and Biomedicine and molecular biosciences (25.6%), followed by Health sciences (16.5%), whereas other academic areas such as Social sciences (4%) and Psychology (3.3%) produce very few. This research agenda bias has also been described for other health conditions (5, 33). As only one Mission in the integrated agenda, “Development and innovation of biomedical tools for prevention and diagnosis”, is related to the academic areas that focus most on HIV/AIDS (i.e., clinical medicine and biomedicine), seven of the agenda’s eight missions and 17 of its 18 R&I topics may be considered “underserved topics” (33) and therefore contribute to redressing the “research agenda bias” in health research.

## 4 Discussion

This study contributes to emerging efforts to improve the resolution of complex health challenges by engaging a wide variety of stakeholders and using systemic approaches to set up R&I and action agendas. It shows how applying a participatory and systems-oriented approach led to identifying a broad range of needs and barriers to HIV and other STI prevention. This approach resulted in an agenda spanning different academic areas and covering key systemic elements within and beyond the health system, elements found in all the system characteristics described in the System Analysis Tool. The resulting agenda covers all the research areas identified in our literature review (see Supplementary File 1), including “evaluation of public policies,” “evaluation of interventions,” “integrated healthcare,” “bioethics and law,” “education and communication,” “perception of risk and behaviour change” and “stigma and health inequalities,” among others. The final agenda is structured around eight missions that cover multiple relevant solutions, including recommendations for R&I topics such as research on preventive vaccines, microbicides and treatment as prevention, and actions related to prevention strategies, healthcare access, community-based activities, and policy development. Some of the recommendations are highly related to the health system, such as “equal access to prevention, diagnosis and treatment tools and services” (Mission 4), “assessment of stakeholders’ needs and interventions” (Mission 7) and “development and innovation of biomedical tools for prevention and diagnosis” (Mission 8). It also covers other recommendations highly related to other systemic elements, such as “new funding model and sufficient resources” (Mission 1), “new models of governance, regulation and promotion of human rights” (Mission 2), “equal access to information, education and communication” (Mission 3), “psycho-social aspects related to culture, values and stigma and discrimination” (Mission 5) and “zero use of psychotropic substances and night leisure” (Mission 6).

A wide diversity of students and professionals provided input. The results show that the diversity of perspectives from the different types of professionals within academia, the community affected by HIV and other STIs, policy makers and the education community (i.e., teachers and students) complemented each other. We demonstrate that each participant group contributed valuable input to the agenda and that the process facilitated an urgently needed mutual learning, particularly evident during the integration phase when disagreements were discussed. This is in line with Harting et al.’s (34) analysis of the underlying rationales of patient and public involvement in health promotion research. They highlight the need to include “more diverse, extensive and context-specific bodies of knowledge” in order to obtain “new and more reliable, credible and valid knowledge” because “non-experts see problems, issues and solutions that experts miss” (34). They also conclude that these processes lead to a “socially robust, holistic and shared understanding of research problems and objectives” and that “giving lay participants a say in the formulation of research questions promotes relevant research which is meaningful to the communities affected by the issue being studied” (34).

Most of the research topics in the resulting agenda are described in the scientific literature as underserved research needs, given that current publications are mostly focused on clinical medicine and biomedicine and molecular biosciences (4). These fields were only represented in one of the agenda’s missions and R&I topics, meaning that current scientific publications do not cover the majority of the academic areas in the agenda. Therefore, we conclude that the SDM has been shown to be a useful approach for (1) integrating the experiential and professional knowledge of the wide diversity of stakeholders engaged, including researchers and professionals working in different (academic) areas and in the community as well as teachers and students, (2) identifying key systemic elements and (3) identifying underserved R&I needs.

Our findings contribute to current global strategies for HIV and STIs (35), which highlight the need to change the way professionals address social and structural barriers by “engag[ing] communities and civil society (…) and support[ing] their self-empowerment and pivotal role in advocacy, service delivery and policy making” (35). These global strategies also call for a systems-oriented approach “that promotes synergies with primary health care, health governance, financing, workforce commodities and service delivery while also fostering multisectoral responses” (35). According to Plough (36), such an approach would provide opportunities to fundamentally change the status quo so as “to eliminate health disparities based on the social, environmental and economic determinants of health and well-being; and catalyze a national movement that demands and supports a widely shared, multifaceted vision for a culture of health to replace the siloed approach to health and health care”.

Other research has also combined participatory research processes with systems approaches, aiming to design and evaluate public health interventions in complex systems, while avoiding a narrow focus and the targeting of a limited number of determinants at a single environmental level (37–39). However, most of these studies have still taken a multicomponent approach rather than a complex systems approach (37). In addition, although “the relationship and responsive adaptation between the intervention and the complex adaptive system has not been the focus of the programmes”, they have still been conceived as “packages of isolated actions, rather than coordinated actions that are indivisible from their complex adaptive systems” (38). Programmatic efforts are mostly still “trapped in a short-term cycle of planning (of 1 year or less), scoping for 1 year and reporting in a final year. This results in action within strictly defined roles and competition for external funding and resources between different groups within the same community” with limited collaboration and effectiveness (40).

Therefore, although there is growing consensus on the need to evolve towards systems approaches for complex problems, there are still few public health interventions applying them. Thus, due to the insufficient evidence base, no definitive conclusion can yet be drawn regarding the effectiveness of their implementation (41). The only long-running intervention of this type that has been documented is the “Amsterdam Healthy Weight Approach”, which has been shown to be effective (38).

### 4.1 Strengths and limitations

Although the study shows that the SDM is a useful tool to operationalize systems approaches with a wide diversity of stakeholders, its implementation had some limitations. First, during the data interpretation stage, the students who participated earlier could not be found to validate the results. This is a major limitation and had implications for the validity of the final agenda, as interpretative authority may have shifted towards professional stakeholders. Care was taken to ensure that students’ inputs were not sidelined and that all changes made by professionals to their items were kept to a minimum and closely monitored. Nevertheless, for the legitimacy of the final agenda involvement of students in the validation of the merged agenda would have been strongly preferred. To avoid this limitation in future studies, we suggest that the calendar for the implementation of the SDM be better adapted to the schools’ calendar (42, 43).

Second, during the data collection, the teachers acted as co-researchers using citizen science (42), which was crucial for engaging a wide diversity of young students. However, the teachers’ involvement as co-researchers meant relying on their data collection and analytical skills, which is a common limitation of citizen science (43). This anticipated limitation was addressed by offering training, facilitation guidelines, monthly meetings and backstopping services, including access to researchers for further support, when needed.

Third, three FGs with students were excluded out of the total of 16, because the teachers did not send in the results. However, given the large number and diversity of participants, we assume that the influence on the final integrated agenda was minimal. Nevertheless, we cannot rule out that a few items might have been missed because of this.

Fourth, the generalizability of the results – that is, to what extent the results are representative of the entire community of professionals and students in Catalonia and to what extent they can be generalized to other geographical areas – is unknown. The participating schools were part of the community of practice for innovative R&I implementation strategies in schools, providing a willing community to participate in the study, and this may set these participants apart from other professionals and students in Catalonia. This limitation is intrinsic to participatory processes. To assess generalizability, we recommend implementing this study in other schools in Catalonia and other geographic areas.

Further research is also needed to explore how the SDM could be improved by adding an extra phase in which the resulting solutions are contrasted with scientific information to ensure they are evidence-based whenever possible. This might avoid inconsistencies such as the need to “get rid of leaflets” because they are perceived as not being effective, identified in Mission 3 (information, education and communication), and the suggestion to provide “information leaflets when selling contraceptive pills in pharmacies to inform about prevention of STIs,” identified in Mission 4 (equal access to diagnostics).

Engaging a wide diversity of stakeholders was not only key to identifying recommendations for R&I topics, prevention strategies, healthcare access, community-based actions and policy development within and beyond the health system, but also to increasing the likelihood of the priorities being implemented. However, to ensure that both the key aspects within the different areas and key systemic elements are implemented, new iterations of the SDM will be required for each mission of the integrated agenda, involving a wider diversity of stakeholders. Because each mission has specific complexities, the new iterations will require the involvement of stakeholders who may not have been involved in this first iteration and who may be key for identifying R&I topics and actions and for designing and implementing projects to address them.

Regarding the implementation of the integrated agenda achieved during subsequent phases of this project, although some projects were implemented and a new iteration of the SDM was performed, the Barcelona City Council, together with other key stakeholders, decided to implement the new agenda within participatory groups led by stakeholders who are not authors of this paper. Therefore, we cannot guarantee that the SDM will continue to be implemented to continuously explore and address systemic barriers. Further research should therefore explore how to address governance, accountability and funding constraints for sustained institutional uptake of participatory systems approaches to R&I. We recommend implementing this research as part of the next phases of the SDM with diversity of stakeholders (from the public administration, the research and innovation community, and the private and third sectors) that may already agree on the ownership of the missions. As recent scientific literature has shown for other health conditions, systems benefit when priorities are organised around actionable missions designed and prioritized based on external quantitative benchmarks, realistic implementation pathways and with opportunity costs in mind (44–47). Accordingly, the strategic research should be designed as a mixed-methods process that integrates quantitative evidence, in order to address existing implementation constraints and prevent the development of utopian, non-actionable agendas.

## 5 Conclusion

Using the SDM to engage a wide variety of stakeholders and including a systems analysis resulted in a more integrated agenda for HIV and other STIs prevention that covers relevant academic areas and addresses key systemic elements. The agenda contains eight missions covering recommendations for R&I topics and actions related to prevention strategies, healthcare access, community-based actions and policy development. These recommendations are within and beyond the health system, and most of them can be considered underserved research needs, given their low presence in current scientific publications. We therefore recommend that HIV and other STIs prevention programs be designed through iterative and participatory systems processes involving a wide diversity of stakeholders with a shared responsibility. Such iterations should apply the SDM to better address current key systemic barriers. To ensure implementation of integrated agendas and sustained uptake of participatory and systems approaches, we recommend applying subsequent phases of the SDM through participatory research with a focus on enabling conditions – addressing governance, accountability, and funding constraints – while engaging a diverse range of stakeholders and integrating key quantitative evidence on benchmarks, feasible pathways, and opportunity costs.

## Data Availability

The original contributions presented in the study are included in the article/Supplementary material, further inquiries can be directed to the corresponding author.
