# Supplementary material for: A systemic, multistakeholder participatory approach to research, innovation and action agenda setting for more integrated and effective prevention of HIV and other STIs: the case of Catalonia, Spain
Source: Front Public Health. 2026 Sep 23;14:1783030. doi: 10.3389/fpubh.2026.1783030 (PMC13644724; doi:10.3389/fpubh.2026.1783030)
Supplement: Supplementary file 1 [file Supplementary_file_3.pdf]

Additional file 3. Educational guidelines

TEACHING GUIDE

Xplore Health

Towards AIDS eradication!

DEVELOPED BY:

Obra Social "la Caixa" eduCaixa IrsiCaixa Institut de Recerca de la Sida

www.xplorehealth.eu

WORKING SCENARIO

This module is divided into four distinct sections each containing a range of activities for learning about diverse aspects of HIV and AIDS. The first section uses a series of videos and a virtual game guided by questions to explore HIV and the cycle of HIV infection. After introducing the main topic – HIV – chart analysis is used to accompany the student in identifying the different stages of this viral infection and the immune response that characterises it. After setting the context for HIV and AIDS, the students are invited to conduct a virtual experiment to find out the current state of research on HIV and the available pharmaceuticals, and to create a hypothetical vaccine that, if it were real and effective, would help to eradicate AIDS. Before ending, the module suggests holding a debate to discuss the day-to-day ethical aspects affecting HIV prevention, diagnosis, treatment and research. Lastly, the guide suggests a project to summarise what the students have worked on in this module: to create a poster for the prevention of HIV, directed either to the students of the class or to other students at the school.

CONTENT

The specific content covered in this module is as follows:

- **HIV**: origin and development (phylogenetic tree), concept and characterisation, type, geographical distribution, replication capacity, components, cycle of infection, transmission, stages of infection, treatment and pharmaceutical resistance, HIV vaccine, HIV research projects, ethical aspects of HIV.
- **AIDS**: concept and characterisation.
- **Genetic material**: DNA, RNA and its components, variability, random mutations, mutation rate.
- **Immune system**: immune reactions, lymphocytes and opportunistic diseases.
- **Laboratory materials**: pipettes (Pasteur, multi-channel micropipette, serological), centrifuge, tubes, waste containers.
- **Scientific method**: hypothesis, experimentation, results and conclusions.
- **Laboratory techniques**: aseptic techniques, ELISPOT techniques.
- **Charts**: variables, interpreting charts, units of concentration (cells/mm^3, RNA copies/ml).

The outline below describes the content in more detail, divided by educational level:

Year 10 Biology and Geology

- Investigation and experimentation:
  - The stages of research.
  - Designing research to validate hypotheses that require the control of variables.
  - Supporting conclusions.
- The responses of the body:
  - Health and disease. Infectious diseases.

- The immune system as an effector responding to foreign substances in the body.
- Vaccination.

- Human reproduction:
  - Sexually transmitted diseases. Human sexual response. Sexual health and hygiene.

Year 11 Biology and Geology (optional subject)

- Research and experimentation:
  - Research project. Possible strategies for searching for answers to a question in a school science context: formulating researchable questions, hypothesis, experiment design, obtaining data, results and conclusions.
- Life, preservation and change:
  - DNA. Composition, structure and biological functions. Concept of gene. Mutations.

Year 12 Biology

- From genotype to phenotype:
  - Analysis of the composition and structure of nucleic acids.
  - Significance of gene mutations.

Year 13 Biology

- Bacteria and viruses in action:
  - Characterisation of viruses as acellular structures.
  - Description of the composition, morphology and structure of viruses.
  - Description of viral cycles.
  - Characterisation of the retrotranscription process.
  - Relationship between the viral infection process and human health: examples of viral diseases.
  - Evaluation of some of the social implications of viral diseases.
  - Characterisation of the health-disease binomial.
  - Analysis of the types of immune responses.
  - Explanation and contextualisation of the antigen-antibody reaction.
  - Interpretation of the immune system's action with respect to vaccines.
  - Recognition of the advances of biomedicine in the treatment of infectious diseases and critical evaluation of access to these resources.

Key stage 5 (Science for the contemporary world)

- Science, health and lifestyles:
  - Health and social implications of certain lifestyles, with a particular emphasis on healthy habits.
  - Types of disease, based on typical examples from each group.
  - Scientific, technological and political infrastructure that social concerns represent for health.
  - Key concepts of genetics and its applications.
  - Critical thinking in aspects of science.
  - Molecular and physiological bases of genes.
- Materials, objects and technologies:

- Potential scope of nanotechnology progress in improving health.

TEACHING OBJECTIVES

A list of the learning objectives that students must reach by working through this module is detailed below. The aims are normally broken down into three categories (conceptual, procedural and behavioural). In the case of the objectives corresponding to a particular educational level and/or a specific activity, these are specified in brackets after the objective. These objectives are cross-disciplinary: in other words, they are achieved through various activities from the module, not including any additional reference.

Conceptual objectives

- Distinguish between HIV and AIDS.
- Identify the RNA, capsid, matrix and viral envelope as the main components of an HIV virion (**Key stage 5 only**).
- Sequence the cycle of HIV infection, understanding what takes place at each stage (**Key stage 5 only**).
- Understand the relationship between the reverse transcription process and the high rate of HIV mutation (**Key stage 5 only**).
- Establish the high rate of HIV mutation as the leading cause of resistance to treatment in HIV+ individuals (**Key stage 5 only**).
- List the treatments available for HIV.
- Explain the mechanism of action of existing pharmaceuticals to treat HIV (**Key stage 5 only**).
- Recognise the requirements of a vaccine to eliminate HIV infection.
- Identify the ways in which HIV is transmitted.
- Recognise CD4+ lymphocytes as HIV host cells and the role they play.
- Explain what an opportunistic disease is.
- Explain what antiretroviral pharmaceuticals are and what they do (**Key stage 5 only**).
- Distinguish between HIV controllers and non-controllers on the basis of immune response.
- Identify the main laboratory materials used in virtual experiments to find a vaccine for HIV.
- Describe the mechanism of action of a vaccine on the immune system and for AIDS specifically.
- Identify the problems creating resistance to HIV treatment (**Key stage 5 only**).
- Learn about certain HIV research projects.
- Discuss the main ethical problems affecting the treatment and prevention of HIV infection.

Procedural objectives

- Encourage pair work and group work.
- Interpret and draw conclusions from a chart showing the blood concentration of HIV RNA and CD4+ lymphocytes.
- Write a report on a virtual experiment to find a vaccine for HIV.
- Participate in debates on HIV and its related ethical aspects.

- Participate in a debate on the state of research into vaccines for HIV and pharmaceutical resistance (**Key stage 5 only**).
- Reflect on the importance of collaboration between countries in the search for a HIV vaccine.
- Create a poster to promote HIV prevention.

Behavioural objectives

- Self-assess the learning process of concepts of HIV and AIDS in order to increase student awareness of them.
- Listen to and respect classmates.
- Adopt an attitude of respect and tolerance towards people with HIV.
- Work in pairs and groups on projects such as the creation of a poster to prevent HIV infection.
- Adopt a critical approach to self- and co-evaluations of completed assignments.

COMPETENCES ACQUIRED

This section will cover the competences acquired throughout the module. First of all, we will concentrate on the broad, scientific/technological competences which are acquired as part of compulsory secondary education. We will then look at the general competences required for Key stage 5, as well as the specific competences of the different subjects used in this module: Biology and Science for the contemporary world.

Level Key stage 4

Contributions to Key stage 4 transversal competences

Communication competence

- Watching a range of videos on various aspects of HIV and AIDS and extracting information from them.
- Debates on HIV and related ethical issues, taking into account current scientific knowledge.
- Set of multiple-choice questions with timed answers.
- Production of a written report on a virtual experiment to find a vaccine for HIV.

Digital competence

Students will develop this competence by:

- Watching a range of videos on various aspects of HIV and AIDS.
- Taking part in an interactive virtual game.
- Conducting a virtual experiment to find a vaccine for HIV.

Personal and social competence

This competence is promoted through the following activities:

- Debates on HIV and related ethical issues, taking into account current scientific knowledge.
- Co-evaluation and self-evaluation of a range of assignments completed individually, in pairs or as small groups.
- Creation of a poster to prevent HIV infection.

Contributions to scientific/technological competences

Competence 4. Identify and solve scientific problems suitable for investigation in a school context, involving the design, carrying out and communication of experimental research.

This competence is covered through the following activities:

- Virtual experiment to research an HIV vaccine.
- Report on the virtual experiment to research an HIV vaccine.

Competence 5. Solve everyday problems with scientific reasoning.

Students will develop this competence by:

- Creating a poster to prevent HIV infection.

Competence 12. Adopt preventive measures and healthy habits on an individual and social level based on knowledge regarding the human body's strategies for detecting and responding to disease.

Students will develop this competence with the following activity:

- Creating a poster to prevent HIV infection.

Competence 15. Respond to matters concerning human sexuality and reproduction based on scientific knowledge, evaluating the consequences of risk behaviours.

This competence is promoted through the following activities:

- Debates on HIV and related ethical issues, taking into account current scientific knowledge.
- Creation of a poster to promote safe sexual behaviour in HIV prevention.

Level Key stage 5

Contributions to general Key stage 5 competences

Communication competence

Students will develop this competence by:

- Debates on HIV and related ethical issues, taking into account current scientific knowledge.
- Set of multiple-choice questions with timed answers.
- Production of a written report on a virtual experiment to find a vaccine for HIV.

Competence in managing and dealing with information

Students will develop this competence by:

- Watching a range of videos on various aspects of HIV and AIDS and extracting information from them.
- Creation of a poster to prevent HIV infection.

Digital competence

Students will acquire this competence by:

- Watching a range of videos on various aspects of HIV and AIDS.
- Taking part in an interactive virtual game.
- Conducting a virtual experiment to find a vaccine for HIV.

Research competence

This competence is covered by:

- Conducting a virtual experiment to find a vaccine for HIV.
- Production of a written report on a virtual experiment to find a vaccine for HIV.

Personal and interpersonal competence

Students will develop this competence by:

- Debates on HIV and related ethical issues, taking into account current scientific knowledge.
- Co-evaluation and self-evaluation of a range of assignments completed individually, in pairs or as small groups.
- Creation of a poster to prevent HIV infection.

Competence in knowledge and interaction with the world

This competence will be covered by:

- Creating a poster to promote safe sexual behaviour in HIV prevention.

Contributions to specific subject competences (Biology)

Competence in investigation and experimentation

This competence will be developed by:

- Conducting a virtual experiment to find a vaccine for HIV.
- Production of a written report on a virtual experiment to find a vaccine for HIV.

Contributions to specific subject competences (Science for the contemporary world)

Investigation competence

This competence is covered by:

- Conducting a virtual experiment to find a vaccine for HIV.
- Production of a written report on a virtual experiment to find a vaccine for HIV.

Competence in the social and civic dimension of science and technology

The following activities can be carried out to cover this competence:

- Debates on HIV and related ethical issues, taking into account current scientific knowledge.
- Watching a range of videos on the current state of the research to find a vaccine for HIV.
- Creating a poster to promote safe sexual behaviour in HIV prevention.

Competence in reflecting on the nature of science

Students will develop this competence by taking part in:

- Debates on HIV and related ethical issues, taking into account current scientific knowledge.

PROPOSALS FOR TEACHING SEQUENCE

We will now explain the different teaching activities proposed in the general sequence: they are placed at the relevant level, an accurate description is made of each one, the content covered is listed, different classroom dynamics are proposed (which you will find described in the Appendix 1), as well as control and evaluation activities (see evaluation tools in the section of the same name). Finally, a timing is suggested for each activity.

Main sequence

Session	Activities
Session 1	What is HIV and what is AIDS?; What does HIV involve?
Session 2	How does HIV infect us?
Session 3	Play: Putting the brakes on AIDS
Session 4	HIV, the virus with multiple changing faces
Session 5	What periods follow HIV infection?
Session 6	Virtual experiment
Session 7	HIV vaccine and pharma-ceutical resistance
Session 8	HIV vaccine and pharma-ceutical resistance
Session 9	Session 9 Ethical aspects of HIV
Sessions 10 and 11	Design of a poster

Teaching justification: this sequence covers all the proposed activities of the module. A range of videos and virtual tools are used to discuss diverse concepts of HIV and AIDS. The sequence is organised so that the level of difficulty increases as students work through the activities. Hence, the early sessions work on theoretical concepts while the latter ones (the seventh onwards) focus on current research on the topic. Lastly, the students take part in a structured debate in order to reach a decision on the ethical and social aspects that they have seen.

Proposed alternative sequence 1

Session 1	Session 2	Session 3	Session 4	Session 5	Session 6	Session 7	Sessions 8 and 9
What is HIV and what is AIDS?	HIV, the virus with multiple changing faces	What periods follow HIV infection?	Virtual experiment	HIV vaccine and pharma-ceutical resistance	HIV vaccine and pharma-ceutical resistance	Ethical aspects of HIV	Design of a poster

Teaching justification: this teaching sequence is intended for year 10 students who have not covered genetic material or viruses specifically. Initially, it focuses on how HIV is transmitted and how it can be prevented. It then looks at the current state of research into new treatments for HIV. Lastly, it discusses ethical issues associated with HIV and AIDS.

Proposed alternative sequence 2

Session 1	Session 2	Session 3	Session 4	Sessions 5 and 6
What is HIV and what is AIDS?	HIV, the virus with multiple changing faces	What periods follow HIV infection?	Ethical aspects of HIV	Design of a poster

Teaching justification: this teaching sequence looks at how HIV is transmitted, the infection that causes it, and the ethical issues associated with the virus and its prevention. This sequence does not go into great detail about the virus itself, concentrating instead on the social problems it causes and the fact that it is a sexually transmitted infection (STI). This makes it a good teaching sequence to include in the sexually transmitted diseases topic in year 10.

Proposed alternative sequence 3

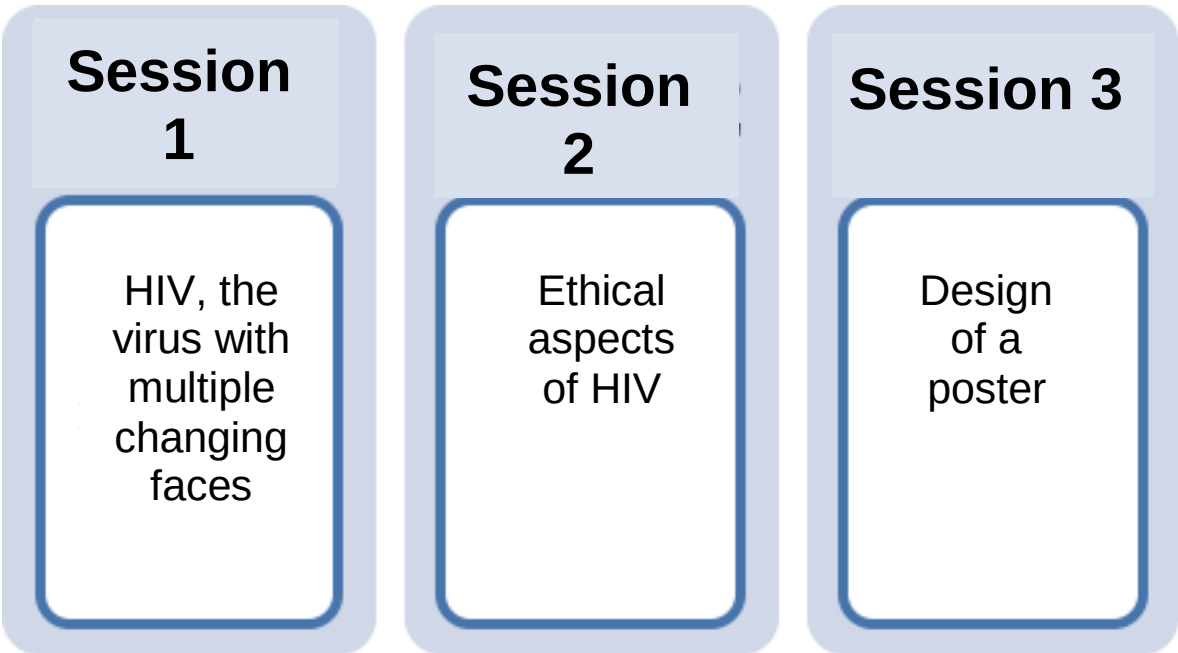

Teaching justification: this teaching sequence is designed for reflecting on HIV infection in the West. It emphasises the social importance of the virus and the ways in which it can be prevented. This teaching sequence puts a strong emphasis on classroom discussion. Given that the other sessions are not used, the teacher will need to introduce the topics of HIV and STIs and may have to clarify concepts that emerge during the discussion. Given the emphasis on ethics and discussion, this could be an interesting teaching sequence for Science for the contemporary world.

TEACHING ACTIVITIES

We will now explain the different teaching activities proposed in the general sequence: they are placed at the relevant level, an accurate description is made of each one, the content covered is listed, different classroom dynamics are proposed (which you will find described in the Appendix 1), as well as control and evaluation activities (see evaluation tools in the section of the same name). Finally, a timing is suggested for each activity. The page indicated below the title of the activity makes reference to the page that is covered in the student dossier.

**LEVEL** Year 10 and	**TITLE:** What is HIV and what is AIDS? | Student worksheet pages 3 and 4			
DESCRIPTION	CONTENT COVERED	PROPOSAL FOR CLASSROOM TEACHING	CONTROL AND EVALUATION	TIMING
**1.** Exploration of preconceptions about HIV and AIDS based on the questions in Play: 1-3.<br><br>**2.** Viewing of the animation *"What is HIV and what is AIDS?"* **(Key stage 5; for Key stage 4 we recommend that the teacher provide an explanation tailored to the level of the class).**<br><br>**3.** Answer to the questions in Play: 4 on the difference between HIV and AIDS, the difficulty treating it, and the meaning of immunodeficiency.	**HIV:** origin and development (phylogenetic tree), what it is, what types there are, geographical distribution, replication capacity.<br><br>**AIDS**: concept and characterisation.<br><br>**Genetic material:** DNA and RNA, variability, random mutations, mutation rate.<br><br>**Immune system**: immune reactions, lymphocytes and opportunistic diseases.	1. To be done individually.<br><br>2. The video can be watched individually or as a group, as appropriate.<br><br>3. 1-2-4 dynamic, selection of a spokesperson and sharing/correction through active listening.<br><br>**In Appendix 1 you will find a description of the teaching methods mentioned in this table.**	The objectives are explained at the start of the lesson and summarised at the end. A self-evaluation table is used to control the learning process.<br><br>If the Play: 4 activity is done as a written exercise, it can be used as a self-evaluation activity.<br><br>**You will find the tools mentioned here in the evaluation section.**	1: 5 min<br><br>2: 15 min<br><br>3: 15 min

www.xplorehealth.eu

LEVEL Key stage 5	TITLE: What does HIV involve? | Student worksheet page 4			
**DESCRIPTION**	**CONTENT COVERED**	**PROPOSAL FOR CLASSROOM TEACHING**	**CONTROL AND EVALUATION**	**TIMING**
**1.** View the animation “*What does HIV involve?*”.<br><br>**2.** Answer the Play: 5 questions on retroviruses and the key components of HIV.	**Genetic material:** DNA and RNA, variability, random mutations, mutation rate.<br><br>**Parts of HIV:** genetic material, capsid, matrix, envelope.	**1.** The video can be watched individually or as a group, as appropriate.<br><br>**2.** 1-2-4 dynamic, selection of a spokesperson and sharing/correction through active listening.<br><br>**In Appendix 1 you will find a description of the teaching methods mentioned in this table.**	The objectives are explained at the start of the lesson and summarised at the end. The self-evaluation table is used to control the learning process.<br><br>If the Play: 4 activity is done as a written exercise, it can be used as a self-evaluation activity.<br>The questionnaire “HIV and its genetic material” in section Play: 5b can be used as an assessment tool and offers a good summary of what has been covered so far.<br><br>**You will find the tools mentioned here in the evaluation section.**	1. 5 min<br><br>2. 15 min

LEVEL Key stage 5	TITLE: How does HIV infect us? | Student worksheet page 5			
**DESCRIPTION**	**CONTENT COVERED**	**PROPOSAL FOR CLASSROOM TEACHING**	**CONTROL AND EVALUATION**	**TIMING**
**1.** View the animation "*How does HIV infect us?*".<br><br>**2**. In Play: 6, complete activity 3 of IrsiCaixa *Cycle of infection* on the stages of the cycle of HIV infection, the transcription, translation and high mutation rate of HIV and resulting pharmaceutical resistance.	**Cycle of HIV infection**: stages, processes and enzymes involved.<br><br>**Pharmaceutical resistance**: relationship between the high mutation rate and pharmaceutical resistance.	**1.** The video can be watched individually or as a group, as appropriate.<br><br>**2.** The *number* dynamic for completing and correcting activity 3 of IrsiCaixa *Cycle of infection*. We suggest selecting different numbers for each exercise in activity 3.<br><br>**In Appendix 1 you will find a description of the teaching methods mentioned in this table.**	The objectives are explained at the start of the lesson and summarised at the end. The *self-evaluation table* is used to control the learning process.<br><br>The group is awarded a mark based on the efforts of a student drawn at random to do the exercise. If a group does not come out, the group mark can be awarded through the *one for all* technique.<br><br>**You will find the tools mentioned here in the evaluation section.**	**1.** 10 min<br><br>**2.** 30 min

**LEVEL** Year 10 and Key stage 5	**TITLE:** Play: Putting the brakes on AIDS | Student worksheet pages 5 and 6			
**DESCRIPTION**	**CONTENT COVERED**	**PROPOSAL FOR CLASSROOM TEACHING**	**CONTROL AND EVALUATION**	**TIMING**
**1.** Play "Play and help put the brakes on HIV" while completing the "Answers that could be improved on" table on page 6 of the student dossier **(Key stage 5). If used for Key stage 4, the teacher must help with the questions on genetic material.**	**HIV**: concept and characterisation, history, routes of transmission, cycle of infection.<br><br>**HIV treatment**: types of pharmaceuticals, cost of treatment, cause of resistance, HIV vaccine.	**1.** Pair work. Given that different questions appear each time students enter the game, we suggest an *active listening* activity to share the "Answers that could be improved on" (table on page 6 of the student dossier).<br><br>**In Appendix 1 you will find a description of the teaching methods mentioned in this table.**	The objectives are explained at the start of the lesson and summarised at the end. The *self-evaluation table* is used to control the learning process.<br><br>**You will find the tools mentioned here in the evaluation section.**	40-50 min

**LEVEL** Year 10 and Key stage 5	**TITLE:** HIV, the virus with multiple changing faces | Student worksheet pages 7, 8 and 9			
**DESCRIPTION**	**CONTENT COVERED**	**PROPOSAL FOR CLASSROOM TEACHING**	**CONTROL AND EVALUATION**	**TIMING**
**1.** Exploration of previous ideas about how HIV is transmitted.<br><br>**2.** Watching of the video “HIV, the virus with multiple changing faces” **(if used for year 10, the video should be paused to clarify concepts, especially after minute 3, when it talks about genetic material and the cycle of infection).**<br><br>**3.** Completion of the exercises Look: 1, 2 on how HIV is transmitted, the IS cells affected, opportunistic diseases and antiretroviral pharmaceuticals **(if used for year 10, the answer to exercise Look: 2c should be simplified).**	**HIV transmission**: routes of transmission, risk behaviours for infection, prevention.<br><br>**Stages of HIV infection:** phases of infection, immune system, opportunistic diseases.<br><br>**Treatments:** antiretroviral pharmaceuticals, side effects, state of research.	**1.** To be done in pairs and then written in a single list on the board as a whole class. The list is created using *active listening*.<br><br>**2**. Watched as a class.<br><br>**3.** *Pencil in the middle* dynamic.<br><br>**In Appendix 1 you will find a description of the teaching methods mentioned in this table.**	The objectives are explained at the start of the lesson and summarised at the end. The *self-evaluation table* is used to control the learning process.<br><br>Individual answers to these questions can be evaluated.<br><br>**You will find the tools mentioned here in the evaluation section.**	**1.** 10 min<br><br>**2**. 7 min<br><br>**3.** 40 min

LEVEL Year 10 and Key stage 5 | **TITLE:** What periods follow HIV infection? | Student worksheet pages 9 and 10

DESCRIPTION	CONTENT COVERED	PROPOSAL FOR CLASSROOM TEACHING	CONTROL AND EVALUATION	TIMING
**1.** Completion of the exercise Look: 3, which requires interpreting a chart on the change in blood concentration of CD4+ lymphocytes and HIV RNA copies/ml of blood in an individual with HIV undergoing pharmaceutical treatment.<br><br>**2.** Watching of the animation “What periods follow HIV infection?”.<br><br>**3.** Completion of the exercise on page 10 of the student dossier requiring the students to fill in a table on the stages of HIV infection, their characteristics, how long they last and the immune response.	**Charts**: variables, interpreting charts, units of concentration (cells/mm^3, RNA copies/ml).<br><br>**Stages of HIV infection:** window period, viral load, latent phase, activated immune system, weakened immune system, AIDS, opportunistic infections, lentivirus.	**1.** *1-2-4 dynamic.*<br><br>**2.** Watch the animation together as a class.<br><br>**3.** Keep in the same groups of 4 as for the first activity in this block and fill in the table following the *pencil in the middle* dynamic.<br><br>**In Appendix 1 you will find a description of the teaching methods mentioned in this table.**	The objectives are explained at the start of the lesson and summarised at the end. The *self-evaluation table* is used to control the learning process.<br><br>Group evaluation of the table using the “*one for all*” technique.<br><br>**You will find the tools mentioned here in the evaluation section.**	**1.** 20 min<br><br>**2.** 5 min<br><br>**3.** 30 min

LEVEL Key stages 4 and 5	TITLE: Virtual Experiment | Student worksheet pages 11 and 12			
**DESCRIPTION**	**CONTENT COVERED**	**PROPOSAL FOR CLASSROOM TEACHING**	**CONTROL AND EVALUATION**	**TIMING**
**1.** Reading of "How is research conducted?" on the virtual experiment website about controllers and non-controllers and the mechanism of action of vaccines.<br><br>**2.** A virtual experiment is conducted to find a vaccine for HIV in "Come and do it in the lab!" on the virtual experiment website **(The experiment can be played as a game even though certain points are not understood. The game should be stopped regularly, particularly for Key stage 4 students, to check that the students have understood it.)**<br><br>**3.** Writing of a report on the completed virtual experiment.	**HIV vaccine:** controller and non-controller patients, effective immune response.<br><br>**Laboratory materials**: pipettes (Pasteur, multi-channel micropipette, serological), centrifuge, tubes, waste containers.<br><br>**Scientific method**: hypothesis, experimentation, results and conclusions.<br><br>**Laboratory techniques**: aseptic techniques, ELISPOT techniques.	**1**. Done as individuals or in pairs (depending on how many computers are available), summarising what the students have learned. The teacher asks questions (what is the difference between a controller and a non-controller? Which individuals have a more efficient immune response?, etc.) and checks that the students have understood using the *thumb-o-meter* technique.<br><br>**2.** Done as individuals or in pairs (depending on how many computers are available).<br><br>**3.** If the teacher would like the students to write the report individually, use the *write and correct in pairs* technique.<br>If the teacher would like the students to write the report in pairs, use the *2 to 4* technique.<br><br>**In Appendix 1 you will find a description of the teaching methods mentioned in this table.**	The objectives are explained at the start of the lesson and summarised at the end. The *self-evaluation table* is used to control the learning process.<br><br>Checking that students understand the explanations given is also a way to control student learning because it gives them the chance to realise when they misinterpret things.<br><br>Regardless of the classroom dynamic used, students and teachers can use tool 3 to evaluate or give feedback on the report.<br><br>**You will find the tools mentioned here in the evaluation section.**	**1.** 10 min<br><br>**2.** 15-20 min<br><br>**3**. 20-30 min

LEVEL Year 10 and Key stage 5	TITLE: HIV vaccine and new ways to combat pharmaceutical resistance. | Student worksheet pages 12 and 13			
**DESCRIPTION**	**CONTENT COVERED**	**PROPOSAL FOR CLASSROOM TEACHING**	**CONTROL AND EVALUATION**	**TIMING**
**1.** Contextualisation of the current situation of HIV and AIDS around the world: 34 million people worldwide infected with HIV, 7,000 new diagnoses every day.<br><br>**2.** Students watch the video "*Let's join forces to find a vaccine for HIV*", which talks about the CUT'HIVAC project and how vaccines work. Discussion of the material and content in the video.<br><br>**3.** Students watch the video "*New ways to combat pharmaceutical resistance*", which talks about the CHAIN project, the points of the cycle of HIV infection at which the different pharmaceutical families act, and resistance to these pharmaceuticals. Discussion of the material and content in the video **(for Key stage 4, certain concepts of the cycle of infection will need to be explained).**<br><br>**4.** Completion of the Experiment: 2	**Research projects**: CUT'HIVAC, CHAIN project, importance of cross-country research projects, preclinical projects, preclinical models, nanoparticles, induction of effective immune responses, CD8 response, HIV vaccine and problems caused by the high mutation rate of HIV.<br><br>**Cycle of HIV infection**: points at which the different pharmaceutical families act.<br><br>**HIV pharmaceutical resistance**: what it is, cause, consequences, how it is investigated.	**1.** Teacher's explanation. A brief discussion can be held on whether the students think a lot of people are infected. The idea is that the teacher should guide the discussion towards the questions "Should it be investigated? Do you think this is happening?".<br><br>**2.** The whole class watches the video and participates in the subsequent discussion.<br><br>**3.** The whole class watches the video and participates in the subsequent discussion.<br><br>**4.** 1-2-4 *dynamic.* Can be corrected using the *number* or *active listening* techniques.<br><br>**5.** The whole class watches the video.<br><br>**In Appendix 1 you will find a**	The objectives are explained at the start of the lesson and summarised at the end. The *self-evaluation table* is used to control the learning process.<br><br>If the short discussion in point 1 is held before watching the video, it will be based on the previous ideas of the students.<br><br>The teacher can assess student participation in the discussions during the session using evaluation tool 4.<br><br>**You will find the tools mentioned here in the evaluation section.**	**1.** 5 min-10 min<br><br>**2.** 20 min<br><br>**3.** 20 min<br><br>**4.** 30 min<br><br>**5.** 15 min

exercises on the content of the video **(for Key stage 4, decide whether exercise Experiment 2f should be included).**<br><br>**5. Optional:** students watch the videos "*Let's join forces to find a vaccine for HIV*" and "*New ways to combat pharmaceutical resistance*" to make sure they fully understand the concepts.		**description of the teaching methods mentioned in this table.**		

**LEVEL** Year 10 and Key stage 5	**TITLE:** Ethical aspects of HIV | Student worksheet page 14			
**DESCRIPTION**	**CONTENT COVERED**	**PROPOSAL FOR CLASSROOM TEACHING**	**CONTROL AND EVALUATION**	**TIMING**
**1.** Teacher explains what the session will cover.<br><br>**2.** Students watch the video "*Ethical aspects of HIV*", which looks at several ethical issues concerning the treatment and prevention of HIV infection.<br><br>**3.** Participation in the *Discussion Continuum* game.	**Ethical and social issues:** intensive treatment (lots of tablets every day), problems with access to medicines, chronic illness, misinformation about HIV and how to prevent it, resistant HIV viruses, high-risk groups.	**1.** If the teacher says that the class is going to have a debate on the ethical aspects of HIV and says that the arguments they can use appear in the video, this may encourage the students to pay more attention when they watch the "*Ethical aspects of HIV*" video.<br><br>**2.** The whole class watches the video.<br><br>**3.** The proposed dynamic is available in Appendix 1 *(Debating game: Discussion Continuum)*. **Attention!** The Discussion Continuum materials must be photocopied and the game cards cut in terms of the number of participating teams.<br><br>**In Appendix 1 you will find a description of the teaching methods mentioned in this table.**	The objectives are explained at the start of the lesson and summarised at the end. The *self-evaluation table* is used to control the learning process.<br><br>If the short discussion in point 1 is held before watching the video, it will be based on the previous ideas of the students.<br><br>Peer evaluation of participation in the game done by the students using tool 5.<br><br>**You will find the tools mentioned here in the evaluation section.**	**1.** 5 min<br><br>**2.** 6 min<br><br>**3.** 40 min

**LEVEL** Year 10 and Key stage 5	**TITLE:** Design of a poster | Student worksheet page 14			
**DESCRIPTION**	**CONTENT COVERED**	**PROPOSAL FOR CLASSROOM TEACHING**	**CONTROL AND EVALUATION**	**TIMING**
**1.** The students are split into groups of 4 to create a poster for HIV/AIDS prevention.<br><br>**2.** Communication or sharing of the end product.	This will differ from group to group, but it should at least contain:<br><br>**HIV**: concept and characterisation, incidence, routes of transmission, methods of prevention.<br><br>**AIDS:** concept, difference from HIV.	**1.** The teacher creates 4 heterogeneous groups and goes through the classroom supervising the work and offering support to the groups.<br><br>**2.** The teacher should indicate where (by giving presentations, organising a sort of scientific congress, giving informative talks, etc.) and how to distribute/communicate the poster: in the classroom, outside the classroom, in other groups of the school, by uploading it on to the school's website, to other organisations in the district not connected to the school, etc.<br><br>**In Appendix 1 you will find a description of the teaching methods mentioned in this table.**	Sharing the teacher's evaluation rubric is one way to control learning. The evaluation rubric for the poster is available in tool 6. It should be shared with the students or agreed on before work starts on the actual creation of the poster.<br><br>Tool 7 is used for co-evaluation and sharing the mark for the assignment with the other members of the group.<br><br>**You will find the tools mentioned here in the evaluation section.**	**1.** One or two sessions.

RECOMMENDATIONS AND TOOLS FOR EVALUATION

General evaluation criteria for the module

This sub-section covers all of the evaluation criteria for the activities in the module. If sequences are carried out incorporating only some of the activities, the corresponding criteria must be chosen from this list.

- Identify the basic components of HIV.
- Explain, in order, the key aspects of the cycle of HIV infection.
- List the treatments available to combat HIV infection.
- Identify the routes of HIV transmission.
- Describe the mechanism of action of a vaccine for a person infected with HIV in terms of their immune response.
- Participate actively in the classroom dynamics, especially in discussions.
- Respect someone's turn when they are speaking and the opinions of other students.
- Actively participate in discussions that are evaluated and co-evaluated.
- Participate in work done in pairs or small groups.
- Correctly interpret charts on the blood concentration of CD4+ lymphocytes and HIV RNA.
- Write a report on an experiment to find a vaccine for HIV that will be co-evaluated in class.
- Design an eye-catching poster to prevent HIV infection.
- Participate in the creation of a poster; this project will be co-evaluated and self-evaluated by the students.
- Promote the distribution and awareness of these posters inside and outside the classroom.

Evaluation tools

This sub-section details the different evaluation tools in regard to the different teaching activities in the module, as well as recommendations for how they should be implemented.

Tool 1. Self-evaluation table

This table is designed to control student learning by raising their awareness of what they have covered in class. The concepts studied are grouped by activity, so if the complete sequence is not followed, the part of the table to be self-assessed can be selected.

Students should put a cross in the box where they are at with regard to their knowledge of the item in question. The table should be checked at the end of each class so that students can put the cross in a different box when they see fit.

	I know…	I do not know	I have an idea, but I don't know how to explain it	I know and I can explain it	Which activity helped me to understand it
What is HIV and what is AIDS?	what a virus is.				
	what HIV is.				
	what AIDS is.				
	the difference between HIV and AIDS.				
	what the immune system is.				
	the meaning of immunodeficiency.				
What does HIV involve?	what retroviruses are.				
	the components of DNA.				
	the difference between DNA and RNA.				
	the main components of HIV.				
How does HIV infect us?	what the stages of the cycle of HIV infection are.				
	what happens at each stage of HIV infection.				
	the relationship between the reverse transcription process and the high rate of HIV mutation.				
	what the causes of resistance to HIV treatments are.				
Play: Putting the brakes on HIV	the different types of pharmaceuticals available to treat HIV.				
	the function of each type of pharmaceuticals available to treat HIV.				
	the requirements that a vaccine must meet to				

	eliminate the HIV infection.				
HIV, the virus with multiple changing faces	what the routes of HIV transmission are.				
	which cells infect HIV and their function.				
	what an opportunistic disease is.				
	what antiretroviral pharmaceuticals are.				
What periods follow HIV infection?	the names of the different stages of HIV infection and how long they last.				
	what the characteristics of each stage of HIV infection are.				
	which immune response takes place at each stage of HIV infection.				
Virtual experiment	the difference between controllers and non-controllers.				
	how vaccines work.				
	the different parts of a report on an experiment.				
	how to produce a report on an experiment.				
	the difference between results and conclusions.				
HIV vaccine and new ways to combat pharmaceutical resistance	what certain HIV research projects involve.				
	what vaccines do once they have been introduced into the body.				
	what we hope an AIDS vaccine will do.				
	methods of administering vaccines without needles.				

	The cause of HIV pharmaceutical resistance.				
	the problems that cause resistance to these pharmaceuticals.				
Ethical aspects of HIV	how to participate in a discussion.				
	the main ethical problems of HIV treatment.				
	the importance of preventing HIV infection.				

Tool 2. One for all

This is a technique that consists of collecting a notebook at random from one of the group members and correcting the exercise in question. The score obtained will be given to all group members. In this case, it is important to pay attention to the contents of the response more than its presentation.

Tool 3. Correction of experiment reports

This tool can be used by students to give feedback and/or for teachers to correct the report. In the first case, it is important to inform students about constructive criticism in a way in which the last table (comments) is the one that will most help their classmates to learn from mistakes and improve their work. In the second case, adding the desired weighting for each point at the discretion of the teacher is necessary. Teachers can also take advantage of the last table to encourage students to correct their reports instead of having teachers make corrections on the paper.

	Aspects to be evaluated	Yes or correct	No or can be improved	Comment: how can it be improved?
General aspects	All parts (introduction, hypothesis, procedure, analysis of results and conclusions) are included.			
	The text follows a logical order.			
	Spelling is correct.			
	The presentation is			

	acceptable.			
Introduction	Contains the experiment's objective.			
Hypothesis	Follows the "If..., then...", "Perhaps..." or a similar structure.			
Procedure: explanation of the what, how and why	Presented by orderly points.			
	Obtaining white blood cells.			
	Adding HIV fragments.			
	Immune response.			
	Adding antibodies.			
Results	Speak about the variation of colours found on the little wells and what changes in colour mean.			
Conclusions	Speaks about controller patients, non-controller patients and people not infected with HIV.			
	Relates it with the initial objective.			

Tool 4. Table for evaluating participation in a debate

Student name	Respects the turns to speak (Yes/No)	Participates (Yes/No)	Makes relevant contributions (Yes/No)	Speaks correctly (1: not at all – 4 very well)
1				
2				
...				

Tool 5: Rubric for evaluating participation in the *Discussion Continuum* game

Aspects to be evaluated	Excellent 4 points	Good 3 points	Correct 2 points	Average 1 point	Points
Involvement from the members of the group	The whole group has been very involved in the game.	Everyone has taken part, but to different extents.	Nearly everyone has taken part, but to different extents.	Some only took part in the first round in placing the cards.	
Discussions to agree on and move cards	The discussions were used to reach agreements and move the cards. Everyone expressed their opinion and has been listened to.	The discussions were used to move the cards, but not everyone has given their opinion or has been listened to.	There were no arguments, but the decision has been imposed by some members of the group.	There were arguments in deciding whether to move some cards or not.	
Respect in taking turns to speak	Everyone has respected their classmates' right to have their say.	In some occasions, more than one person talked at once or one member interrupted another.	Often there was more than one person speaking at the same time or interrupting.	Constantly there was more than one person speaking at the same time or interrupting.	
Co-evaluation	Carried out in a critical and responsible way.	Carried out in a fairly critical and responsible way.	Carried out in a way that is neither critical nor responsible.	Neither done critically nor responsibly or not done at all.	
Total mark for the rubric (total for all points obtained)					
Partial mark for the rubric (total points/16) 10					

Tool 6. Rubric for evaluating posters

Aspects to be evaluated	Excellent 4 points	Good 3 points	Correct 2 points	Average 1 point	Points
Content	The information is excellent: clear, concise and complete.	The information is clear enough but a little incomplete.	The information is unclear and lacks some relevant content.	The information is very simplistic and there is not a lot of it.	
Organisation of content	There is a logical and clear sequence that reflects suitable planning.	The organisation is correct and the content is related to it.	The content is separated into sections, but the transition is not clear.	Confused and without clear direction.	
Presentation	The layout is pleasant: it has a suitable length and uses a suitable font. There are no spelling or grammar mistakes.	The layout is pleasant, but there are some spelling and/or grammar mistakes.	The layout is not pleasant and/or inconsistent, or contains numerous mistakes.	Sentences are unintelligible or contain numerous mistakes. Little care has been taken in the layout of the presentation.	
Creativity	The layout is suitable for the information presented. Unique images are used where necessary and these correctly accompany the information. Relevant ideas have been included that were not found in the sources covered in class.	The images are well chosen and consistent with the text.<br><br>No irrelevant or confusing information or examples.	The images used are standard, but correct. There is some irrelevant information or some confusing examples.	The images do not match the information, contain irrelevant information or confusing examples.	
Total mark for the rubric (total for all points obtained)					
Partial mark for the rubric (total points/16) 10					

Tool 7. Rubric on behaviour and distribution of points awarded.

A document such as the following should be given to each group. The first thing that should be done by students is to fill in the boxes in the table describing actions that they carried out in their group and that reflect the level of cooperation, communication, resolution of issues and contribution that has been made to the work. Following this, teachers should fill in the submission mark (obtained using the previous rubric) and points to award (submission points/number of members in group). Finally, students should share the marks in terms of what individual contributions to the work have been.

How has each member of the group worked? Complete the table noting down the behaviour of each member in every aspect of the group work. Following this, share the marks obtained between the members of the group in a proportionate way based on each group member's contributions to the group work.

	Name:	Name:	Name:	Name:
**Cooperation**<br>Listens to classmates. Helps classmates who have difficulty understanding the content studied. Evaluates the individual contribution of classmates.				
**Communication and resolution of issues**<br>Collaborates in planning out what needs to be done, identifies obstacles and works towards group agreements/solutions.<br>Encourages communication and politeness in the group.				
**Contribution**<br>Takes responsibility for completing the task: starts and finishes the task assigned. Shares ideas and sources of information.				

Assignment mark	Points to share out	FINAL DISTRIBUTION	
		Student name	Points awarded (maximum 10)

APPENDIX 1

1-2-4 structure

A simple cooperative structure where students get into groups of 4. First of all, everybody thinks about and writes down their answers to each question. Then, they get into pairs and share and discuss their answers. Lastly, the whole team decides which answer is the correct one.

Active listening

This dynamic involves listening very carefully to what each classmate says to avoid repeating things. For example, if somebody wants to say something that has already been said, they cannot take part. They can only participate if they want to expand on, contradict or argue against something a classmate has said.

The number

In this dynamic, the students form small groups (4 members is a good size) and the teacher gives each student the same task. Each person has to solve the exercises and can ask the other members of the group for help if they need to. A number is given to each student (for example, this could be their alphabetical position in the register). When the time is up, the teacher selects a student at random to explain the exercise in front of the class. If they get it right, their group gets a reward (a point, to choose the next number, etc.). We recommend that teachers distribute the numbers into different groups. This way, at least one student can be chosen from each group and each group can be evaluated.

Pencil in the middle

Students are grouped into base teams that have as many members as questions in the task they have to do. Each student is responsible for one question and the group decides the order in which they will be answered. Then, each student reads "their question" out loud and all the members discuss it and agree on the correct answer. During the discussion, the pencils of all group members must be placed in the middle of the table. Once everybody is clear about what and how they need to answer, each person picks up their pencil and writes the answer to the question in their exercise book. At this point, they cannot speak, only write. The dynamic is repeated until all the questions have been answered.

Thumb-o-meter

This technique gives the teacher an idea ofthe percentage of students who have understood what was just explained. The teacher must ask a question with two possible answers (yes/no, controller patients/non-controller patients) and tell the students that giving a thumbs up means "Option A", thumbs down means "Option B" and thumbs to the side means "I do not know". (To see if they have understood the dynamic, it can be tested by asking the question "Do you understand what you need to do?" Yes = thumbs up, No = thumbs down and I do not know = to the side. If somebody does not give a thumbs up, one of the students who has understood the dynamic can be asked to explain it to their classmates.) The students should be given 5 seconds to think about the answer. Then the teacher counts to 3 and the students give their answer using their thumbs. If everybody got the answer right, the explanation was understood; if there are some wrong ones, the teacher decides whether one of the students who "did not answer correctly" should explain their point of view or whether one of the students who "answered correctly" should explain the reasons why. Once the explanation has finished, the technique should be used again to check that the "wrong" thumbs are pointing the right way, meaning that it has been understood.

Writing and correcting in pairs

The teacher describes the task and procedure, and keeps an eye on the time. Each student writes their work individually in the time limit set by the teacher. When the time is up, the students swap their work with a partner and make notes on where they differ or points that their partner has missed out, again in the time limit set by the teacher. Finally, each student gets their work back and incorporates the changes they think are relevant.

2 to 4

The teacher gives out a task and sets a time limit. The students work in pairs but are grouped into groups of 4 (A and B, and C and D). Once they have completed the task, A pairs up with D to check the work of A and B, while B pairs up with C to check the work of C and D. The check involves agreeing on results, finding points in common and identifying and discussing points where they differ. The time allocated for the check should be limited and set by the teacher. When the time is up, the students pair up with their original partners and discuss the changes proposed during the check, incorporating the ones they consider appropriate.

Discussion Continuum game

The process is explained in more detail here.

This discussion game contains all the necessary information for students (considered "players") to think about and discuss each issue in detail. The basic instructions for the activity are summarised below:

- The players get into small groups.
- Each player receives an AGREE and DISAGREE token and eight discussion cards.
- The first player reads the first card to the rest of the group.
- Then the first player decides the extent to which they agree with the card. They place the card face up somewhere on a continuous line of discussion cards, nearer to AGREE or DISAGREE depending on what they decide.
- Each player takes turns to read a card, think about it and choose where to position it on the continuous line.
- Once all the cards have been read, understood and placed in a continuous line, the discussion begins. The object is to position the cards between AGREE and DISAGREE in the order agreed by the majority of players. The players have to take a card and discuss whether or not they want to move it.
- At the end of the discussion, each group must have positioned its cards the way that most players agree with.
- If several groups are playing simultaneously, the teacher can share the results of the different groups. Are they similar? Can a member of each group explain their decisions about a particular card?

Xplore Health

AUTHORS OF THIS TEACHING GUIDE:
Rosa Cristina Sayeras Haag

TEACHING CONSULTANTS:
Marcel Costa and Miquel Nistal

COORDINATION:
Rosina Malagrida

DATE:
December 2015

DEVELOPED BY:

Obra Social "la Caixa" eduCaixa IrsiCaixa Institut de Recerca de la Sida

www.xplorehealth.eu

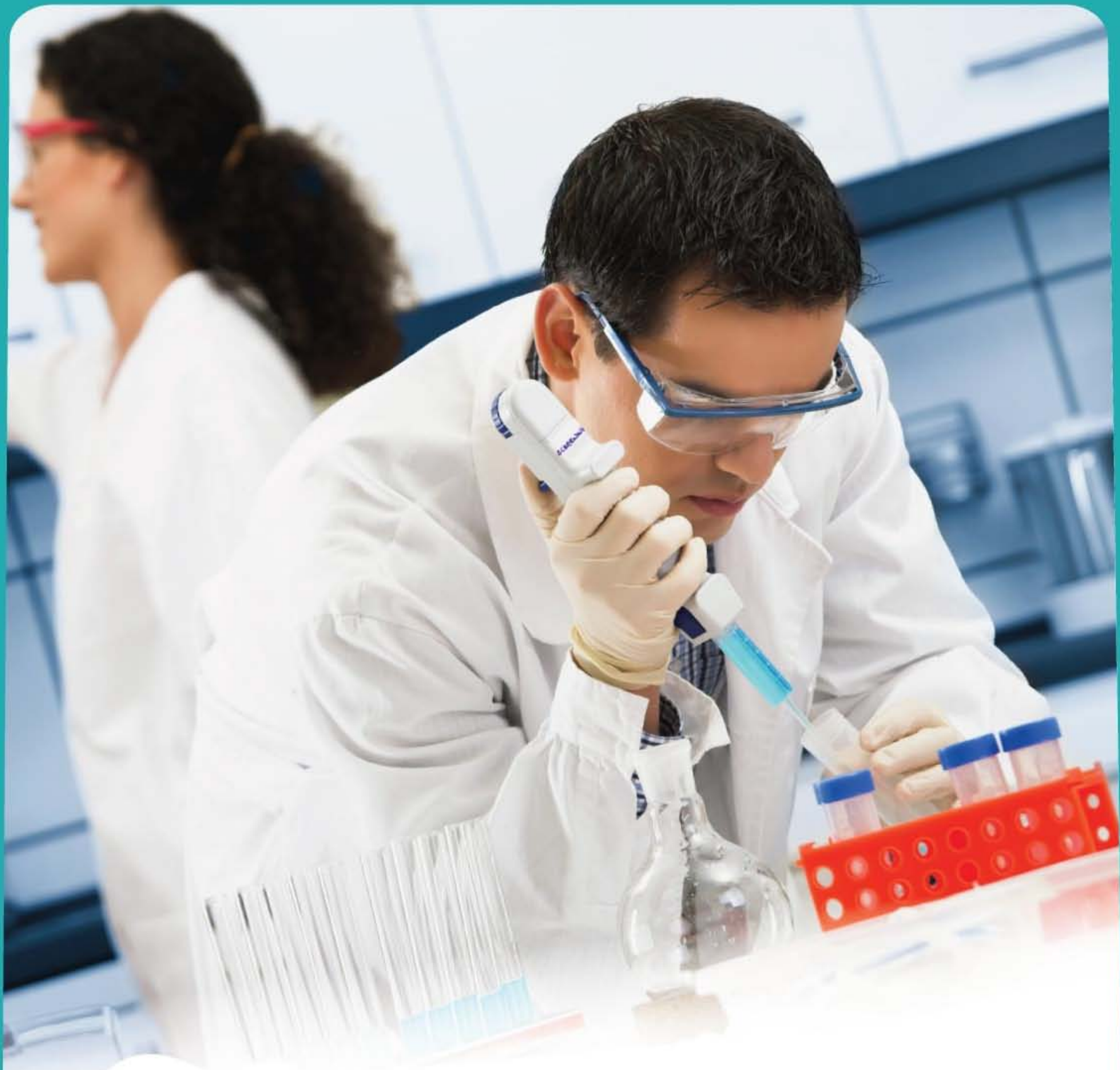

Xplore Health

DISCOVER THE LATEST ON HEALTH RESEARCH

Educators' guide "Towards AIDS eradication"

(Background information)

IrsiCaixa
Institut de Recerca de la Sida

Parc Científic Barcelona

Obra Social "la Caixa"

Centre of the Cell

www.xplorehealth.eu

Xplore Health

Table of contents

1. Introduction	3
2. State of the art	3
2.1. Introduction/ Epidemiology	3
2.2.Transmission	3
2.3. Stages of disease	4
2.4. Prevention	6
2.5. Treatment	7
2.6. Future strategies for elimination HIV	10
2.7. Animal models	11
2.8. Clinical trials	12
3. Ethical, Legal and Social Aspects (ELSA)	13
3.1.Introduction	13
3.2. Prevention	13
3.3. Testing	15
3.4. Treatment	16
3.5. Research ethics	18

1. Introduction

These teacher guidelines will give you information on the Xplore Health module "Towards AIDS eradication". It will first introduce the topic to enable you to prepare your lesson using the different multimedia tools that you will find on the website. The guidelines provide information on the state of the art in this research field and on the ethical, legal and social aspects surrounding this topic.

2. State of the art

2.1. Introduction/Epidemiology

HIV stands for 'human immunodeficiency virus'. At the moment, around 33.3 million people in the world are infected with HIV and 7000 new infections are diagnosed every day.

HIV is a member of the retrovirus family. These viruses live by inserting a copy of their own genome into the DNA in our cells. They are then treated like our own genes and use the machinery of our cells to make new viruses. These new viruses can then infect other cells.

HIV specifically infects the cells of the immune system that we use to destroy infections – mainly CD4+ T cells and macrophages. Infection of these cells impairs or destroys their ability to function normally. This leads to immune deficiency and infected people are more susceptible to a wide range of so called opportunistic infections that the immune system would normally be able to overcome.

Once an HIV infected person develops several symptoms linked to immunodeficiency, their illness is called AIDS, which stands for 'acquired immunodeficiency syndrome'. Most untreated people with HIV eventually develop AIDS, and usually die from opportunistic infections or cancers that occur when the immune system is weakened. Since its discovery in 1981, more than 30 million people have died from AIDS, with the majority in sub-Saharan Africa and a growing number in South and South East Asia.

2.2. Transmission

There are three main transmission routes for HIV:

- sexual
- blood-borne

- mother-to-child

Sexual transmission

The majority of infection is spread through unprotected sexual intercourse. This occurs when virus-containing secretions from the infected person come into contact with genital, oral or rectal mucous membranes of the uninfected partner. Transmission during sexual relations accounts for almost 90% of total infections in the UK, and this is split fairly evenly between those participating in heterosexual sex or sex between men.

Blood-borne transmission

Blood-borne transmission accounts for infection of intravenous (IV) drug users, haemophiliacs and recipients of blood transfusions and can occur when infected blood is directly transferred from one person to another or comes into contact with an open wound. Incidences of transmission via blood products in healthcare situations in the developed world are rare as all blood is screened for HIV before treatment. Infection through IV drug use is still relatively common (5% in the UK) due to the reuse of needles among injecting drug addicts. This type of infection is significantly more common in Eastern Europe (44%)

Mother-to-child transmission

Vertical or 'mother-to-child' transmission can occur during pregnancy, childbirth or via breast feeding. In the developed world, transmission via this route is normally prevented by treating the mother with antiretroviral drugs and performing a Caesarean section during birth. In developing countries however, up to one in three babies born to infected mothers will contract HIV (this makes up to 19% of all new infections).

2.3. Stages of disease

There are three stages of infection recognised in patients infected with HIV. In the first few weeks following exposure to the virus, massive viral replication takes place resulting in high virus levels in the blood. Within weeks of this initial infection, viral replication drops greatly because the immune response to HIV is strong. Then the infected person goes through a stage with no symptoms, which is known as clinical latency. During this time, the number of an important cell type in the immune system, called CD4+ T cells, decreases steadily so that by the time symptoms are noticed, the individual has less than 300 CD4+ T cells/µl (an uninfected person has 500-1000 CD4+ T cells/µl). At the same time, there is an increasing

level of virus in the blood and lymph nodes and this is the beginning of the third, or symptomatic phase, which includes the progression to AIDS.

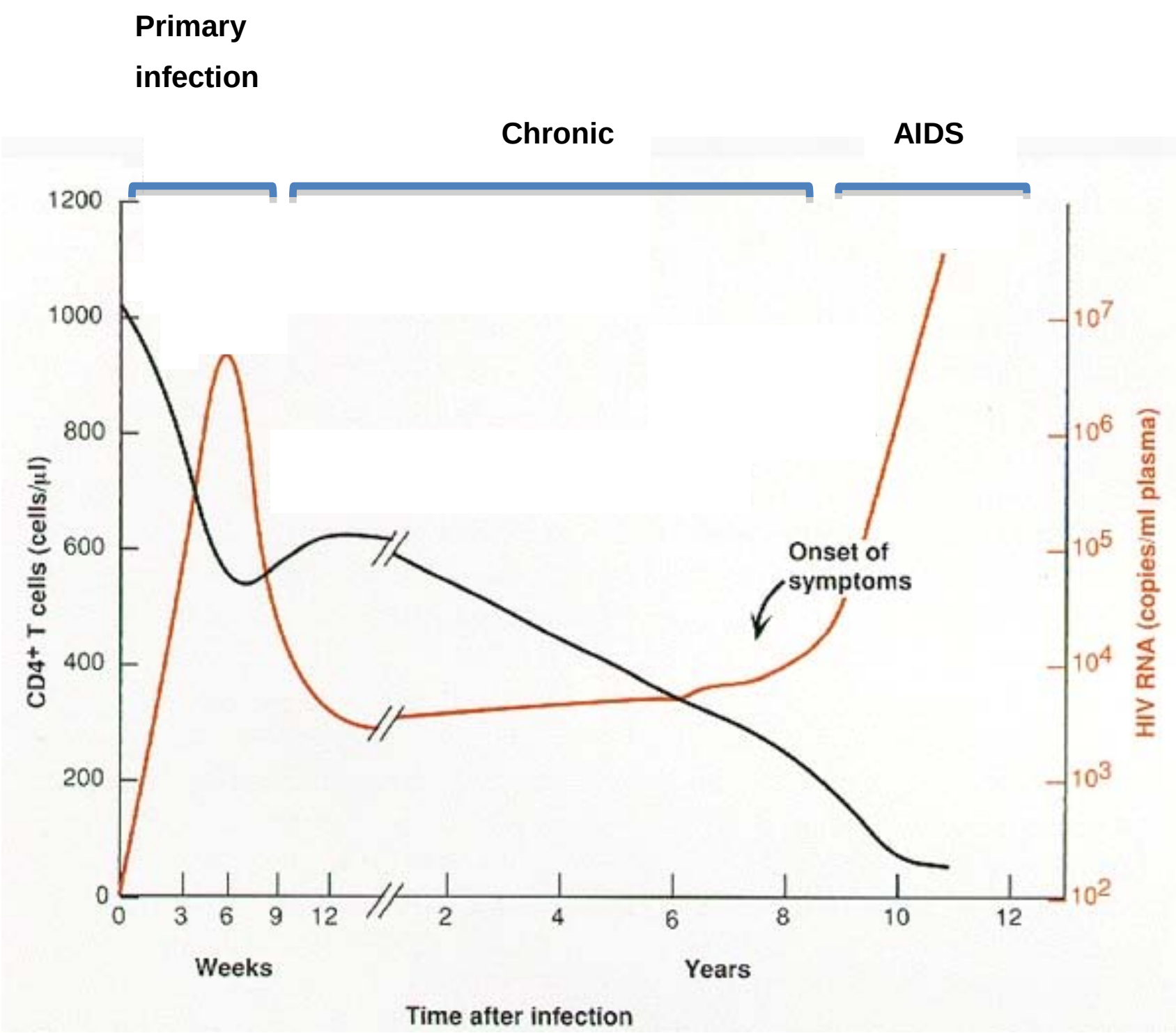

Fig. 1. The disease course of HIV infection. Amount of CD4+ T cells (black) and level of virus in the blood (red) is shown

Initial Infection: Acute Seroconversion Syndrome

For many people, initial infection with HIV may be asymptomatic, but as many as 40-90% of patients in the early stages of HIV infection develop an acute glandular fever-like infection after approximately two to six weeks following exposure. They will often experience fever, rash, joint pains and enlarged lymph nodes.

Seroconversion refers to the development of antibodies to HIV and usually begins between 1 and 12 weeks following infection. The production of these first antibodies is slow, and when they appear, they have only a weak ability to kill the virus. The initial burst of HIV replication is mostly contained by the actions of another cell in the immune system called a CD8+ T cell. The action of the immune response during the early stage of infection is fairly successful, and able to cause a large decrease in the amount of virus in the blood (called a patient's viral load (VL)). However it is not able to stop it completely and HIV replication still occurs in lymph nodes.

Whether or not symptoms are noticed during this period, the high levels of virus in the blood means that individuals are particularly infectious.

Chronic Infection: Clinical Latency

After initial infection, most patients have a period of 'clinical latency' that may last for years. Freedom from symptoms does not mean there is no viral replication. The level of infectious virus in the blood during this time is low and maybe even undetectable. Throughout clinical latency the immune system is gradually getting weaker, in particular, there is an overall loss of CD4+ T cells. As the immune system becomes less effective, the patient is more likely to be infected by other organisms. Once this happens, the doctor will diagnose AIDS.

AIDS

A person is classed as having AIDS when the number of their CD4+ T cells falls below 200/μl of blood and they have at least one of 20 opportunistic infections or HIV-related cancers. The destruction of cell-mediated immune responses means that infection by common, usually harmless environmental organisms (eg. Pneumocystis jirovecii) can be life-threatening. Later in infection, the reactivation of herpesviruses can also cause severe recurrences of shingles, Epstein-Barr virus-induced B-cell lymphomas or Kaposi's sarcoma.

The length of time taken for an HIV infected individual to progress to AIDS varies significantly, and depends on many viral, host and environmental factors. Without treatment, most infected people will be diagnosed with AIDS within 5-10 years. However, since the introduction of antiretroviral therapy (ART) in 1996, the number of people in developed nations that become sick from diseases linked to AIDS has reduced. In the UK, there have been approximately 27,000 diagnoses of AIDS since the beginning of the epidemic in 1981, and almost 20,000 people have died.

2.4. Prevention

An extremely important part of the treatment of HIV infection is prevention. HIV is a very fragile virus and cannot survive for long outside the cell. Transmission by the three modes described above can be readily prevented with appropriate precautions.

New preventive technologies such as microbicides (gels, creams or sprays capable of killing the virus) are continually being tested for their ability to prevent transmission through sexual exposure to HIV. At the moment though, condoms are still the main focus of prevention strategies for HIV and other sexually transmitted infections.

The screening of blood and blood products before treatment and the use of disposable syringes are the most effective ways of reducing infection through contaminated blood.

Infection rates among children born to mothers living with HIV have declined by 26% from 2001 to 2009, due mostly to drugs and the ability of infected mothers to give birth by Caesarean section, along with alternatives to breast feeding for infants.

2.5. Treatment

There is currently no cure for HIV.

At the moment, the only treatment options available are a combination of antiretroviral drugs which act at different steps in the life cycle of HIV. Current antiretrovirals target the following stages of HIV replication (see Fig. 2.):

1) Attachment/Fusion
    - This is the newest class of drugs and it interferes with the binding, fusion or entry of the HIV virus into the cell, either by changing a part of the outside of the virus or blocking the parts of the cell membrane that the virus would normally bind to.
2) Reverse transcription
    - These drugs were the first to be developed and act against the viral reverse transcriptase (RT) enzyme. They work either by physically binding to and inhibiting the enzyme's activity or interfering with the production of new viral DNA by forcing it to stop before it is finished.
3) Integration
    - Drugs that act on the viral enzyme integrase prevent the virus from inserting its genome into the DNA of the cell. This means that no new viruses can be produced.
4) Maturation
    - Inhibitors that stop the function of the viral enzyme protease only let non-infectious viruses be produced.

HIV multiplies at a very fast rate, and is able to change itself to become resistant to any drug that it comes across. Using these drugs in a combination of at least 3 different types at once makes it harder for the virus to adapt and become resistant, as it then needs to make multiple changes which may make it less able to infect. So for patients who stick carefully to

their treatment regime, combination ART means that there should be a longer period of time before the virus can become resistant to the drugs, and therefore the virus levels in their blood may be undetectable for many years. For these patients, progression to AIDS is slowed down and a newly diagnosed patient on ART can now experience an improved quality of life with an average life expectancy of 20-50 years depending on the age at which they were infected.

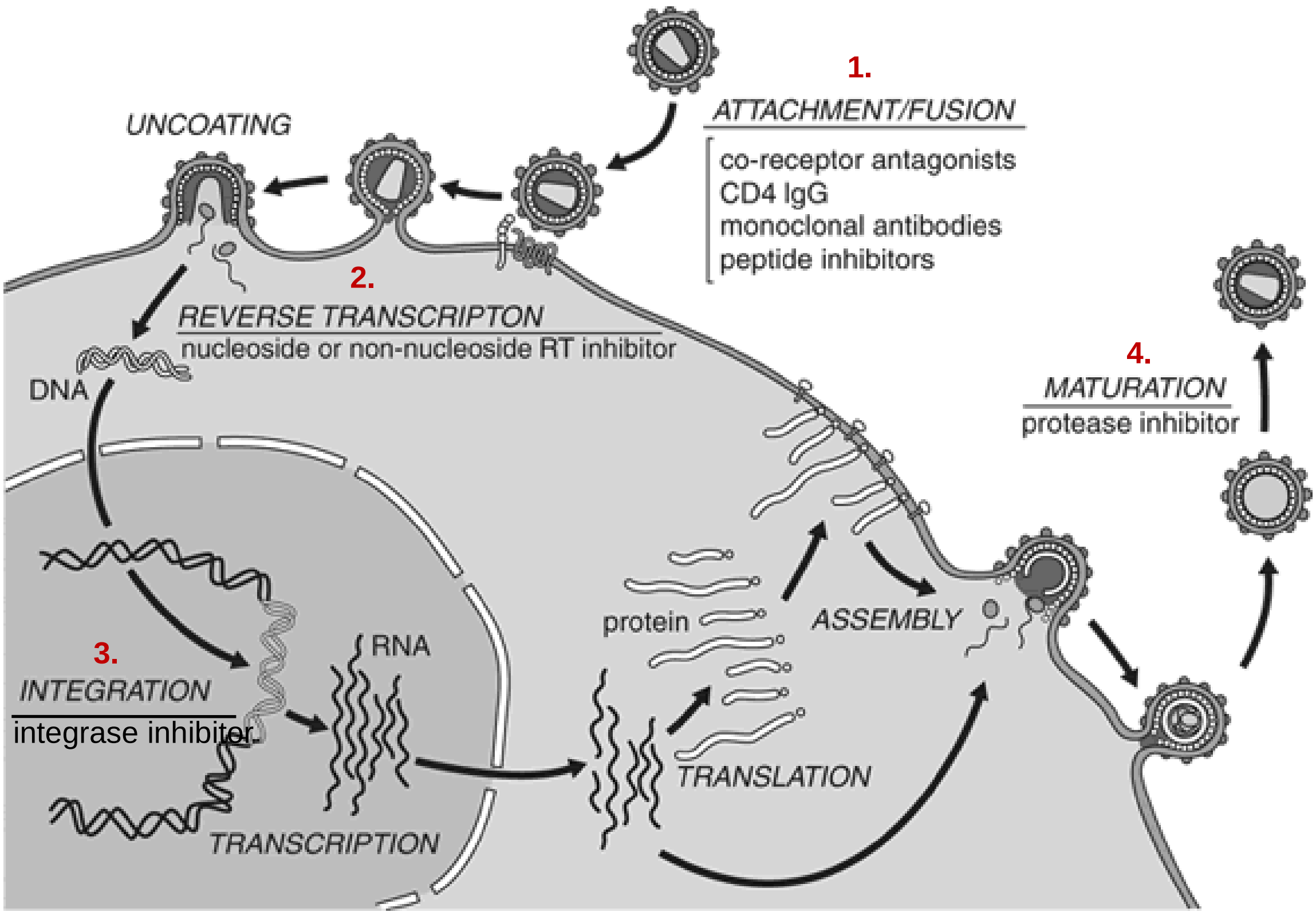

Fig. 2. The life cycle of HIV, showing the stages targeted by antiretroviral drugs.

So why don't drugs eliminate HIV from the body?

Early on, the success of combination ART in reducing HIV replication to undetectable levels led many to believe that a cure for HIV infection had been found. However in reality it seems there are at least three major barriers to curing HIV.

- Long-lived latently infected cells
    - These are infected cells in which the virus is silent, replicating only occasionally and at low levels. These cells can persist for long periods of time and are unaffected by ART or host immune responses.

- Anatomical reservoirs
    - This refers to cells in locations that are physically protected from the effect of ART (eg. virus in brain cells escape from drugs that can not cross the blood-brain barrier).
- Residual viral replication
    - This is due to the inability of ART to 100% inhibit the virus, which results in a continuous low level of replication.

A cure for HIV

There are two possible ways of achieving a cure for HIV.

- Sterilising cure

This refers to a situation where HIV is treated and then disappears altogether. This would mean that all infected cells were eliminated and the patient would have a VL of less than one virus/ml.

- Elimination of HIV following bone marrow transplantation

The only example of this type of cure is the recent report of a patient from Berlin with acute myeloid leukaemia (a cancer of white blood cells) who received a bone marrow transplant from a donor who had a deletion in the CCR5 gene (a human cell needs to have CCR5 on its surface before it can be infected with HIV). After the transplantation, this patient stopped taking ART and has had a VL of less than one virus copy/ml of blood for the three years. Bone marrow transplantation may not be a realistic cure for everybody with HIV infection, but understanding how this patient's 'new' immune system was able to respond positively and completely against infection will be useful in developing treatments available to everyone.

Making cells resistant to HIV

These studies aim to copy the elimination of virus that was seen in the Berlin patient after bone marrow transplantation. He was transplanted with bone marrow cells from a person who had a genetic trait (CCR5 gene deletion) that protects some people from HIV infection. In one example, patients had their own T cells removed which were then treated so that the CCR5 protein (which assists in HIV entry into cells) no longer worked in these cells. These cells were then put back into the patient and the researchers saw that the cells were able to carry out their usual functions for many months. While clinical use of this method of treatment

is many years away, the results show that we may one day be able to change any cells in an individual to be better able to eliminate the virus.

Functional cure

This alternative approach is similar to the idea of 'remission' in cancer patients, where a patient has long term good health without treatment, in the best case with a VL of less than 50 virus copies/ml of blood.

Elite controllers

Elite controllers are a unique group of HIV infected patients who do not have any symptoms and do not need to take drugs to keep their blood VL at an undetectable level. Multiple studies have looked at ways an elite controller's genetic makeup, infecting virus strain and immune response could be interacting to allow them to stay so healthy. In many cases, it seems that a set of genes responsible for alerting the immune system to which cells in the body are infected (called MHC class I) is linked with an elite controller's ability to naturally control of HIV infection. Several components of the immune system, such as a type of T cell called 'natural killer' (NK) cells that are able to kill virus infected cells, also seem to be more active in many of these patients.

2.6. Future strategies for elimination HIV

Several options to eliminate any virus that remains after a patient has been using ART are currently being investigated.

Treatment intensification

This involves changing a patient's drug regime to include drugs from more categories than they would normally be prescribed. The results from these studies so far have been disappointing, and no increased destruction of virus is seen.

Early treatment

In these studies, patients are given ART before their CD4+ T cell counts fall below 200/µl in the hope that less infected cells will end up in locations or situations where the drugs have no effect. Some promising results have been seen, but further investigation is required.

Elimination of latently infected T cells

As virus in the latently infected cells is not usually replicating it cannot be seen by the immune system and so avoids destruction. One idea is to encourage this silent virus to become active and replicate again, so that ART and the immune system can kill the infected cells. Several ways of 'waking up' latently infected cells are being looked into, such as treating with the cytokine IL-7 or activating NF-κB with a drug called prostatin. These molecules are normally produced by cells and can increase the activity of infected cells, making the virus they produce visible to the immune system. Alternatively, drugs such as the histone deacetylase inhibitor Vorinostat or DNA methylation inhibitor decitabine are able to artificially 'switch on' the expression of genes, including the integrated HIV DNA, forcing the virus to make itself visible to the immune system. Some promising results have been seen in patients testing these therapies; however the side effects can be harmful. More research needs to be done to make these treatments safe for clinical use.

Therapeutic vaccination

In this case, vaccination is used to try and boost the ability of the immune system to fight off HIV infection. So far there has been no success with this approach, however recent results from a monkey model of HIV disease were encouraging, suggesting that it may be worth continuing to investigate this method.

HIV vaccine

One of the ultimate goals of HIV research is to develop a vaccine that prevents people from becoming infected. This would be of particular benefit to underdeveloped countries who cannot afford long term drug treatment.

So far, several vaccine candidates have been produced, and have been halted at various stages of the validation process. Generation of a suitable vaccine to HIV is more difficult than for other infectious agents for two main reasons:

- HIV is able to change itself very rapidly, so that the immune system constantly has to readjust its counterattack. This means that some virus will always escape detection and is free to continue to replicate and infect new cells.
- There are multiple different strains of HIV, which while similar, appear as different invaders to the immune response that would be generated by a vaccine. So any vaccine would have to produce an immune response that recognised viruses of all different types.

2.7. Animal models

HIV vaccines are usually tested first in monkeys, normally in a species called macaque. Macaques have their own version of HIV (called simian immunodeficiency virus (SIV)) which causes a similar disease in them to HIV in humans. The immune response observed in the macaque after vaccination is not exactly the same as in humans, which makes this model less than ideal for seeing if vaccines are effective against infection. Recently however, a mouse model has been described which shows a very similar pattern of immune response to that seen in humans. Put together, results from both these animal models will be helpful in working out which vaccine candidates should go into human clinical trials.

2.8. Clinical trials

Most initial approaches in making an HIV vaccine have targeted the envelope of the virus, in particular a viral protein called gp120. At least 13 different vaccines against this part of the virus have been tested in early stage clinical trials, with very little success.

A promising approach to vaccination is now thought to be one that stimulates a much broader immune response – producing HIV-specific antibodies as well as CD8+ T cells that are capable of killing HIV infected cells.

The largest AIDS vaccine trial so far was begun in Thailand in 2003 with a regimen called RV 144. Over 16,000 Thai volunteers were vaccinated and then asked to be tested for HIV every six months for three years, as well as receiving education on how to avoid being infected with HIV. The vaccination program was stopped in 2006 and the results released in September 2009. HIV infection rates were reduced by 31.2% in the vaccinated group compared with those who did not receive the vaccine. While these results showed there was only a limited amount protection to HIV after this 6-injection vaccine, a number of positive aspects were observed.

The RV 144 vaccination strategy made use of the two different sides of the immune system. The first injection, called the ‘primer’, was made up of components that activated the ‘cell-mediated’ immune response, which stimulates CD8+ T cells that are able to kill HIV infected cells. The following five injections, called ‘boosters’, caused the production of HIV-specific antibodies able to kill the virus. This is part of what is known as the ‘humoral’ immune response.

Both vaccine components were tested individually and failed to give any protection from HIV infection. So the results of the RV 144 clinical trial show that the combination of the different

parts of the immune system may be useful for a successful vaccine. This is an important step in the right direction in the fight against HIV and gives us hope that the answer may be just around the corner.

3. Ethical, Legal and Social Aspects (ELSA)

In this section you will find a number of opinions and incentives for discussion in class on ethical, legal and social aspects (ELSA) related to HIV.

3.1. Introduction

HIV/AIDS is a globally significant disease, with significant burdens of disease in all countries, especially in the developing world. It cannot be cured, though with the right treatment people can live with minimal symptoms (though some side effects of treatment) for many years after infection. In much of the world treatment is hard to come by and expensive, in part due to the roles of the pharmaceutical industry and the intellectual property system. In addition, because two of the main routes of transmission of HIV (sexual intercourse and injecting drug use) are topics which draw intense public debate and frequent moralism, the topic of prevention is often fraught with controversy. Finally, even where effective prevention and effective treatment is available, the psychology of risk, and social vulnerability, may lead people to become exposed to HIV unnecessarily.

3.2. Prevention

There are two broad strategies for preventing HIV transmission. The first is to take steps to protect oneself from being exposed to HIV. Since the chances of becoming exposed to HIV other than through a recognised "risk behaviour" are now known to be virtually nil, prevention efforts then focus on (a) minimising high risk behaviours and (b) making high risk behaviours safer. In settings where the main mode of viral transmission is through sexual intercourse, prevention efforts have emphasised variously the use of barrier methods of contraception (condoms, in particular), sexual abstinence, and sexual fidelity within relationships. The precise balance of emphasis given to these is controversial both in terms of advice and health promotion "on the ground" and within the circles of international aid and health policy.

The ethics of health promotion and public health are inevitably entangled to a greater or lesser extent with sexual morality, culture, attitudes to sexuality, gender (in)equality, and the degree to which people feel comfortable and empowered to discuss sexuality and sexual behaviours with their partners and others. Similarly, where the main mode of transmission is

via injecting drug use, the main prevention methods (in particular the use of safe needle disposal, free distribution of clean needles, and the discouraging of needle sharing, which can all be highly effective in reducing transmission rates, run up against the ethics and politics of drug use and “The War on Drugs”. In both these debates around safe sex and around safer injecting drug use there is a tension between a pragmatic utilitarian approach which focuses on “harm reduction” and a more principled, and sometimes highly “moralistic”, approach which links safety to reduction of behaviours considered wrong for some other reason (drug use or sex outside of marriage, for instance).

The other main preventive strategy is to limit the probability that the HIV infected person can transmit HIV to an uninfected person. Some of the methods mentioned above apply just as much here (safer sex practices, not sharing needles in drug use). Another method, which has received a lot of attention in the medical literature from the mid-2000s onward, is the use of antiretroviral treatment to minimise the HIV+ person’s viral load, thus minimising the chance that they can pass on the virus. Although the main reason to treat the HIV+ person with drugs is for their own health, this has been an important development as it gives a further reason to expand treatment, especially in the developing world: treatment has a significant public health benefit as well as significant individual benefits. (This argument also bypasses the negative attitude sadly often found towards HIV+ people – that HIV infection should be considered “their fault” and thus “they don’t deserve treatment”.)

Mention of this negative attitude to people who are HIV+ draws our attention to some other methods which are occasionally discussed and indeed practiced in the name of HIV prevention. People have been dismissed from their jobs in many countries because they are thought to pose a risk to their clients, patients or workmates – although the risk of transmission may be practically nil, even in jobs involving significant contact with blood such as surgery, provided sensible protective measures are used. In some countries, HIV has been made into a notifiable disease, and people have been required to live in supervised accommodation (quarantine, in effect). And in many countries, the criminal law has been used to punish people found criminally responsible for knowingly or recklessly infecting other people. This can be considered a prevention strategy; but it may have as much to do with stigmatising and marginalising HIV+ people. Several of these methods have been strongly criticised on human rights grounds, as well as on the more pragmatic ground that these aggressive responses are ineffective and discourage people from ascertaining their HIV status. On the other hand, it can be argued that people who are HIV+ have a responsibility to

prevent themselves from infecting others, and that deliberate infection is a serious harm which should attract criminal punishment.

An important method for preventing the transmission of HIV is the use of antiretroviral treatment in pregnant women and nursing mothers, to prevent the transmission of the virus to their unborn children or children who are breast-feeding. This is known to be safe and effective, but has attracted controversy in two different ways. First, to offer treatment to a woman only during her pregnancy and the infancy of her children may be considered unfair to her and reinforcing of an image of women as only important insofar as they are (actual or expecting) mothers. Second, there is a tension between those who would insist that breast feeding is the best approach to early child nutrition, and who would insist on this treatment-based approach as a way to support that; and those who insist that breast feeding poses an avoidable risk to the child and HIV+ women should be discouraged from breast feeding.

3.3. Testing

A persistent issue in the debates about the ethics of HIV prevention and treatment concerns the ethics of HIV testing. A consistent theme throughout the HIV era, since the first identification of the virus, has been the importance of getting tested for HIV, for three main reasons: first, to know one's HIV status so that one's health can be appropriately monitored and supported and so that one can take appropriate preventative measures to protect others; second, so that the development of the epidemic can be monitored for public health reasons; and, more recently, so that one can access treatment. However, at every stage there have been countervailing factors which discourage people from seeking testing. Especially in countries which have take a more punitive public response to HIV, but everywhere where HIV+ people are subject to stigma, discrimination, fear, and, not infrequently, hate crimes, there is a serious disincentive to finding out one's HIV status. Even under ideal conditions, people may fear the disclosure of their diagnosis by health professionals or others, in breach of confidentiality. The guidance to professionals on when they may or should breach confidentiality to disclose to a partner or other interested party that the HIV+ person is HIV+ (or indeed that they have been tested) is frequently obscure and may in any case be disregarded. Particular concerns arise in the context of disclosure to insurance companies or employers; on occasion insurers have been known to refuse to insure, or insure only at higher premia, people who have disclosed that they have been tested for HIV, even if the test proved negative.

In part this stigma around testing itself, rather than HIV status as such, is explained by the linkage, in the public mind, of HIV to socially disapproved behaviours or membership of marginalised groups. A patient may not wish to disclose his or her status to his or her partner if this discloses that they caught the virus from someone else; someone else may be discriminated against if it becomes known that they've been tested (though negative) if it is assumed that this means they must be gay; and so on. Sometimes there is an association between a measure which may be sound public health policy and a socially stigmatising attitude: thus, there remains controversy in many countries whether sexually active Men Who Have Sex With Men should be allowed to donate blood, as they are considered automatically to be at higher risk of carrying blood borne viruses such as HIV, whether or not this bears any relationship to their actual viral status or sexual behaviours.

This debate relates back to an earlier debate where in several countries major public health disasters occurred because blood products contaminated with the HIV virus were put into clinical use (blood for transfusion, Factor VIII and IX for the treatment of haemophilia). While it is now generally thought that what went wrong in these cases had more to do with inadequacies in the safe management of blood banks and blood product safety than to do with the behaviour of donors as such, it is still often thought that it is sound and ethical policy to restrict who may donate as part of ensuring the biosafety of blood products.

A current issue concerns whether some groups of people should be compulsorily tested (for instance, pregnant women), especially in high prevalence countries. A slightly less intrusive approach is to "mainstream" HIV testing (as with testing for some other common diseases) in primary care, and to make HIV testing offered to all on an opt-out basis as part of routine infectious disease screening in primary care. Here there is a tricky debate between those who maintain that we have a duty to know our HIV status in order to protect people who are not infected from the risk of infection, and those who maintain that even today an HIV diagnosis is distressing and life-changing so people should actively consent to HIV testing. It might be argued that people have a right not to know their HIV status (as has been argued frequently with genetic testing for inherited disorders). But the better view is that people have a responsibility to know their status, but that this must be balanced with a clinical interest in sensitive and consensual testing, and a social justice interest in preventing discrimination and stigma.

3.4. Treatment

HIV may now be considered a treatable chronic illness, thanks to the development of the combination therapies from the mid-1990s onwards. However, treatment poses certain ethical issues, which can be broken down into clinical issues; public health issues; and global justice issues.

Clinical issues

Because HIV evolves so rapidly in each patient, and because a driver of such evolution is the virus's response to the drugs used to treat it, the use of drug therapies requires careful clinical management and regular, long-term contact with patients. Starting and stopping therapies ad hoc, non-adherence to drug therapy regimes, failing to adjust the precise mix of drugs used when treatment appears to be failing, can all influence the effectiveness of treatment and the evolution of the virus with serious consequences for the health of the patient – and, potentially, to public health (see below). There are clinical debates about at what stage in the development of HIV illness in the patient to commence drug treatment which are both about the clinical success of treatment (or otherwise) and about cost-effectiveness in the use of the drugs.

Many of these issues are particularly challenging in developing world settings, where the ability to monitor treatment may be limited by scarcity of money, clinical facilities and staff. Similarly, if treatment is only available to a limited extent, we may face the clinically important issue that a woman who is given antiretroviral therapy to prevent transmission of the virus to her unborn child or to the children she is nursing may have treatment stopped at a certain point, which will affect how she is treated (if at all) thereafter.

Public Health Issues

Treatment does not benefit solely the person treated. Treatment of HIV+ people has an important role in preventing the spread of the infection as noted above. But it also has a huge benefit in terms of protecting economic productivity, social order, reducing the number of AIDS orphans, preserving family structures, and promoting hope. There are excellent public health reasons for expanding treatment which go well beyond the purely medical. On the other hand, incautious or inappropriate use of treatment plays a part in the development of resistant strains of the virus. This is especially evident when one considers the way that HIV, through its role in depressing the immune system, makes the HIV+ person significantly more

vulnerable to other infectious diseases, notably tuberculosis, which is in its turn developing increasingly dangerous treatment-resistant strains.

Global Justice Issues

A heated issue continues to be access to treatment. Three major barriers to treatment can be noted. First, treatment requires testing, and in societies where there is strong stigma against HIV positive people, people may be unwilling to come forward for testing. This relates to a wider issue concerning the "culture" of HIV: HIV infection, testing and treatment form part of a complex web of social practices and attitudes to illness, sexuality, family life, religion, healthcare… in every society. Appropriate intervention to prevent and treat HIV will always require some level of engagement with this culture, and there are subtle and problematic issues involved in such engagement especially cross-culturally. Much important research in HIV treatment is actually social science research, including research into the interrelationships between human rights, health, and ethics.

A second major barrier to treatment is the lack of sufficient healthcare infrastructure in many parts of the world most affected by HIV. "Rolling out" treatment in resource-poor or widely scattered communities poses serious difficulties. This has led some public health specialists to argue that HIV is a disease of poverty. Treating people with limited resources leads to difficult choices of fair distribution and priority setting, which are entwined with very pragmatic questions of public health strategy. For example, should we concentrate on treating those people who are easiest to get to? Is this an ethical criterion? Or merely one of convenience?

A third major barrier to treatment is suggested by the second: scarcity in the short term is almost always explicable by structural factors and incentives which could be changed, but which seem like "facts of life" at close up. For instance – primary care in rural settings may be thinly spread, and in part that is down to sheer geography. But it is also down to decisions upstream about how many healthcare workers to train; how attractive it may be to healthcare workers to leave to work overseas once trained (the "brain drain"); economic decision-making about healthcare financing; and so on. Much of the debate has focussed on one particular structure: the use of the intellectual property rights system to entrench (albeit temporarily) the right to price antiretroviral drugs at high profit margins and to prevent the manufacture of cheap "generic" copies while the patent lasts. Drugs which may be affordable in the developed world may be quite unaffordable save to a very few in the developing world.

3.5. Research ethics

This issue of affordable access to treatment spills over into debates about the ethics of clinical trials, as much as about the ethics of treatment pricing itself. Trials designed to test cheaper, more sustainable treatment approaches have been tried in various countries, but there are difficult ethical controversies about whether people who are in such trials should be entitled to continue treatment once their time “on study” is over, about whether using dummy (“placebo”) control groups is acceptable when in the developed world the control group would receive standard active drug treatment, and (in vaccine or microbicide trials) whether people who become infected while “on study” should be given treatment.

Authors:

The State of the Art document was drafted by Kelly Cheney Post Doctoral Research Assistant at the Centre for Immunology and Infectious Disease, Barts and The London School of Medicine and Dentistry in London. The ELSA document was drafted by **Richard Ashcroft**, Professor of Bioethics at Queen Mary, University of London.

References – ELSA aspects

- Barnett, T and Whiteside, A *AIDS in the Twenty-First Century* London: Palgrave MacMillan, 2006 (second edition)
- The World Bank *Legal Aspects of HIV/AIDS: A Guide for Policy and Law Reform* Washington, DC: The World Bank, 2007

www.xplorehealth.eu

Xplore Health

DISCOVER THE LATEST ON HEALTH RESEARCH

DEVELOPED BY:

IrsiCaixa Institut de Recerca de la Sida

Parc Científic Barcelona

Obra Social "la Caixa"

Centre of the Cell

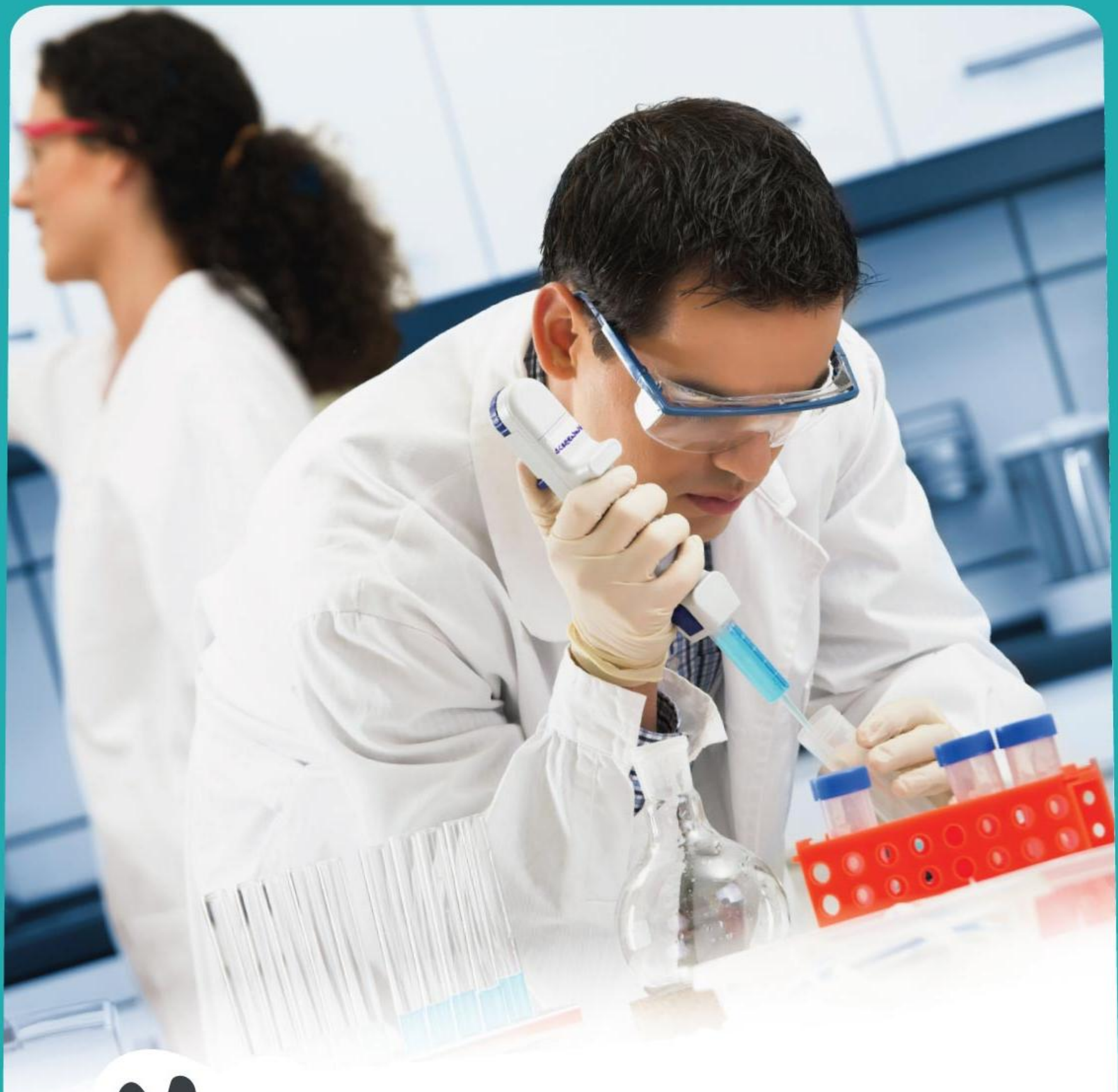

Towards AIDS eradication

Student worksheet

DEVELOPED BY:

IrsiCaixa Institut de Recerca de la Sida

Obra Social "la Caixa"

www.xplorehealth.eu

Student worksheet

TOWARDS AIDS ERADICATION

The following series of activities will help you explore what the scientific community is doing to beat AIDS, a pandemic currently affecting more than 34 million people around the world. You'll find out more about HIV, the virus that causes AIDS and you'll carry out a virtual experiment which simulates designing a vaccine so that the immune system responds efficiently to infection. Finally, you'll take part in a debate, in which you'll have to defend your opinion with good arguments, and set up a prevention campaign.

STOP HIV. Stop AIDS

Play

In the multimedia game you'll play in a minute, you'll be asked to help cure Jan, who is infected with HIV. But before we make a start, answer the following questions:

1. What do you know about HIV?

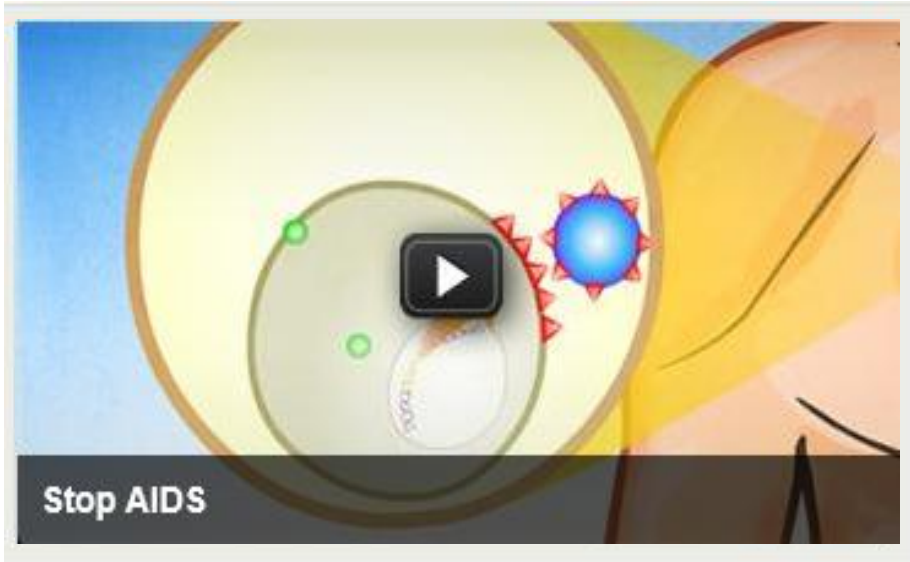

2. The name of the game is: "Stop AIDS." What do you think it means to stop AIDS? How do you think it can be done?

3. Do you think that you need more information in order to answer these questions properly and to well understand the subject matter?

Finding out more:

4. Go to IrsiCaixa's website: http://divulgacio.irsicaixa.es/en and watch the animated film entitled *What do we know about HIV and AIDS?* (http://divulgacio.irsicaixa.es/en/what-do-we-know-about-hiv-and-aids)

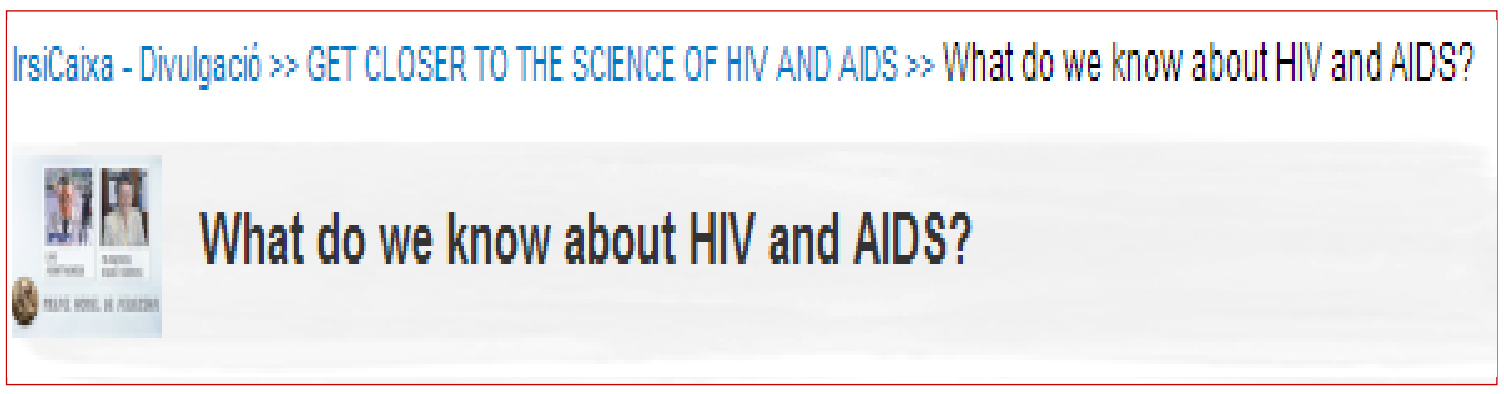

4a. What's the difference between HIV and AIDS? Try to explain it in your own words.

4b. Write a few words about why you think it is so difficult to treat and eradicate.

4c. What does *immunodeficiency* mean? Don't look it up in the dictionary! Try to explain it in your own words.

5. Now watch the IrsiCaixa animation *What is HIV like?* (http://divulgacio.irsicaixa.es/en/what-hiv).

5a. HIV is a so-called retrovirus; what does that mean exactly?

5b. Indicate which components of the virus are the most relevant (working with the worksheet from IrsiCaixa teaching unit 2).

http://divulgacio.irsicaixa.es/sites/default/files/unit_2_eng.pdf

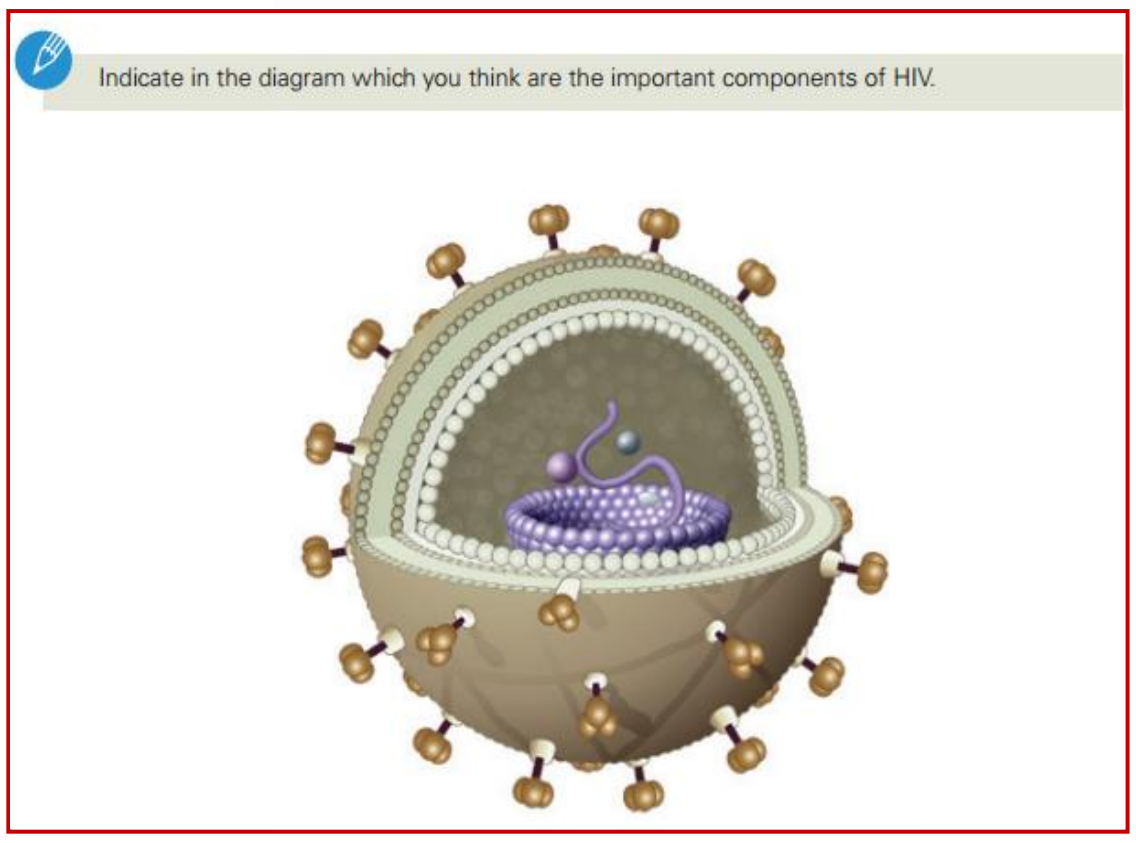

6. Then watch the animation you will find in the section entitled "How does HIV infect us?" called *Stages of HIV infection* (http://divulgacio.irsicaixa.es/en/stages-hiv-infection).

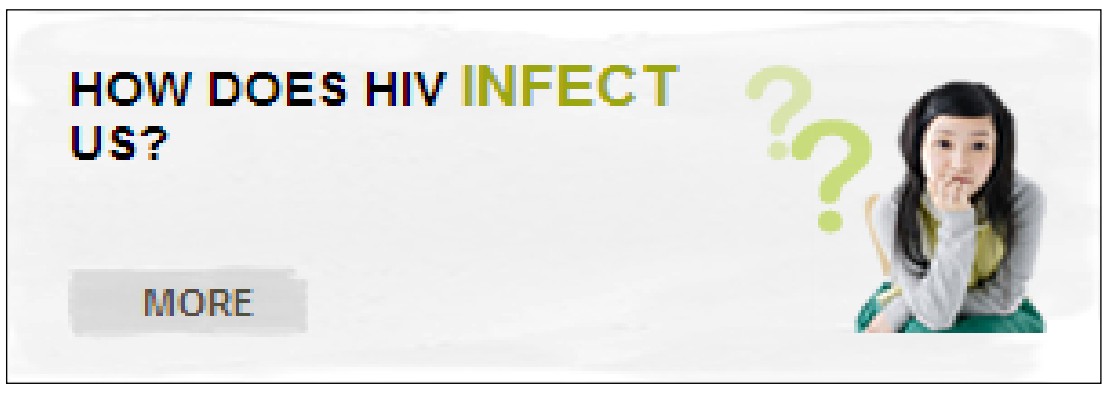

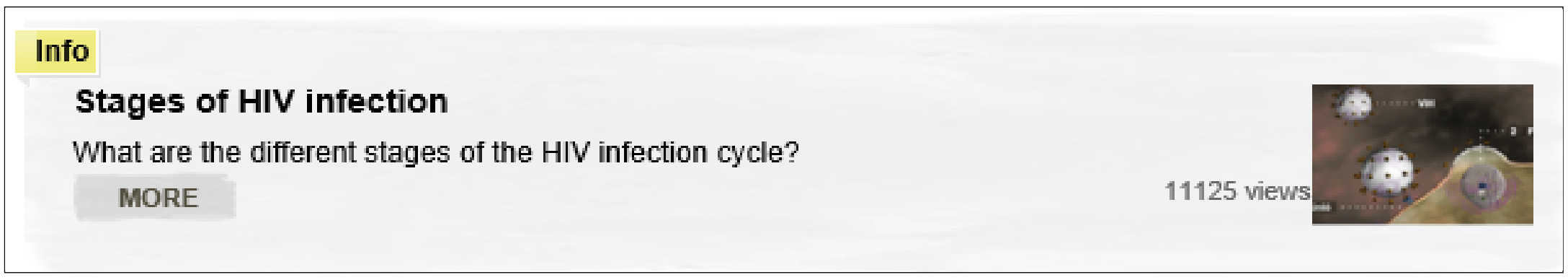

Answer the questions you'll find on IrsiCaixa activity sheet 3: *HIV infection cycle:*

http://divulgacio.irsicaixa.es/sites/default/files/unit_3_eng.pdf

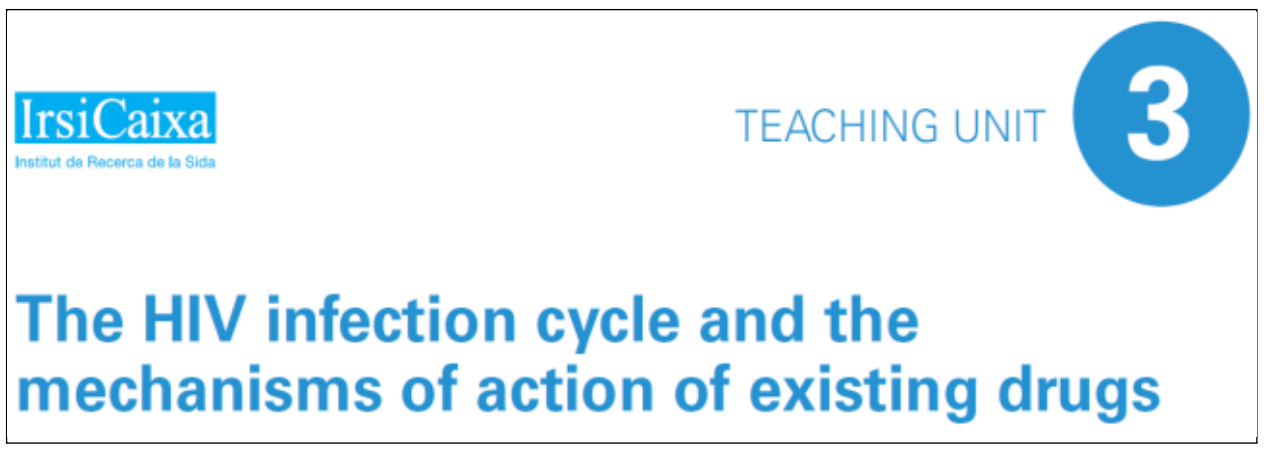

7. It's now time to play the game we mentioned earlier on the Xplore Health website. **Choose the answers you think are correct and then check your results with the self-evaluation**. Play the game in small groups and start by clicking here:

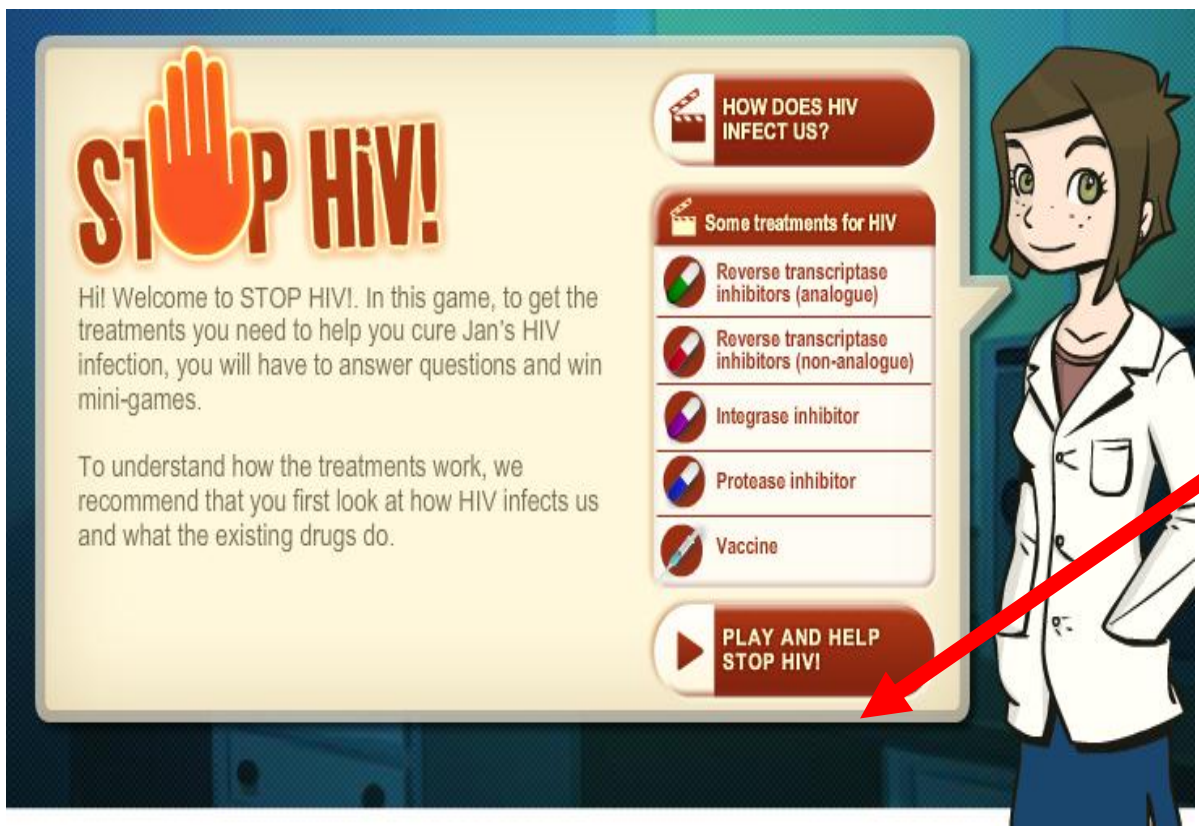

Play...

http://www.xplorehealth.eu/en/media/stop-aids

Answers needing improvement	
**What we answered...**	**What we should have answered...**

Paste a screenshot of your score here.

Watch

HIV, the virus with multiple changing faces

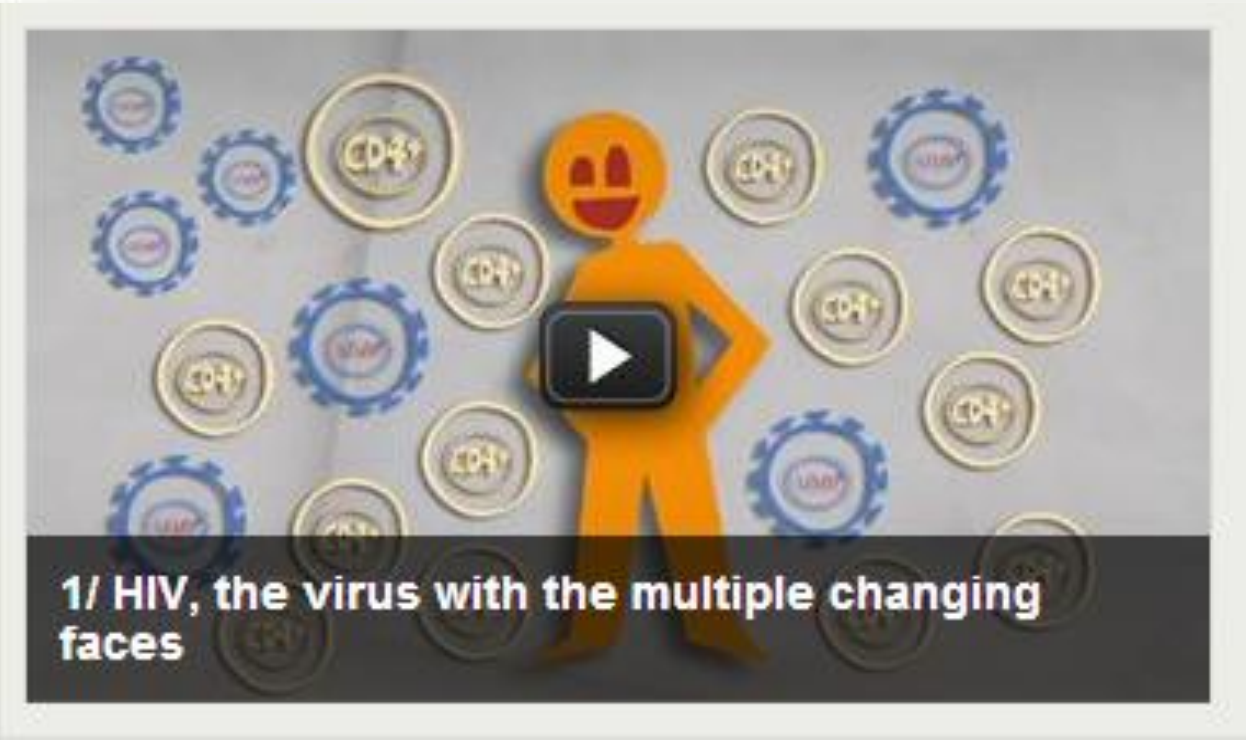

Before watching the video:

Write down all the different ways you know HIV can be transmitted. Check out your answers with everyone else in the group.

Now make one comprehensive list, either in your group or as a whole class.

Now watch the video and check your answers against what they say (http://www.xplorehealth.eu/en/media/hiv-virus-multiple-changing-faces):

1. Fill out the following table:

CAN be transmitted by...	Why?	CANNOT be transmitted by...	Why NOT?

2. In the video, they talk about:

2a. What kind of cells are infected by the virus? What function do they have in the organism?

2b. Describe in your own words what an “opportunistic infection” is.

2c. What are antiretroviral drugs and what do they do?

3. If a patient does not undergo treatment, the HIV infection follows a set development pattern which is summarised and shown in the graph below.

Look at the graph and try to interpret the information in it:

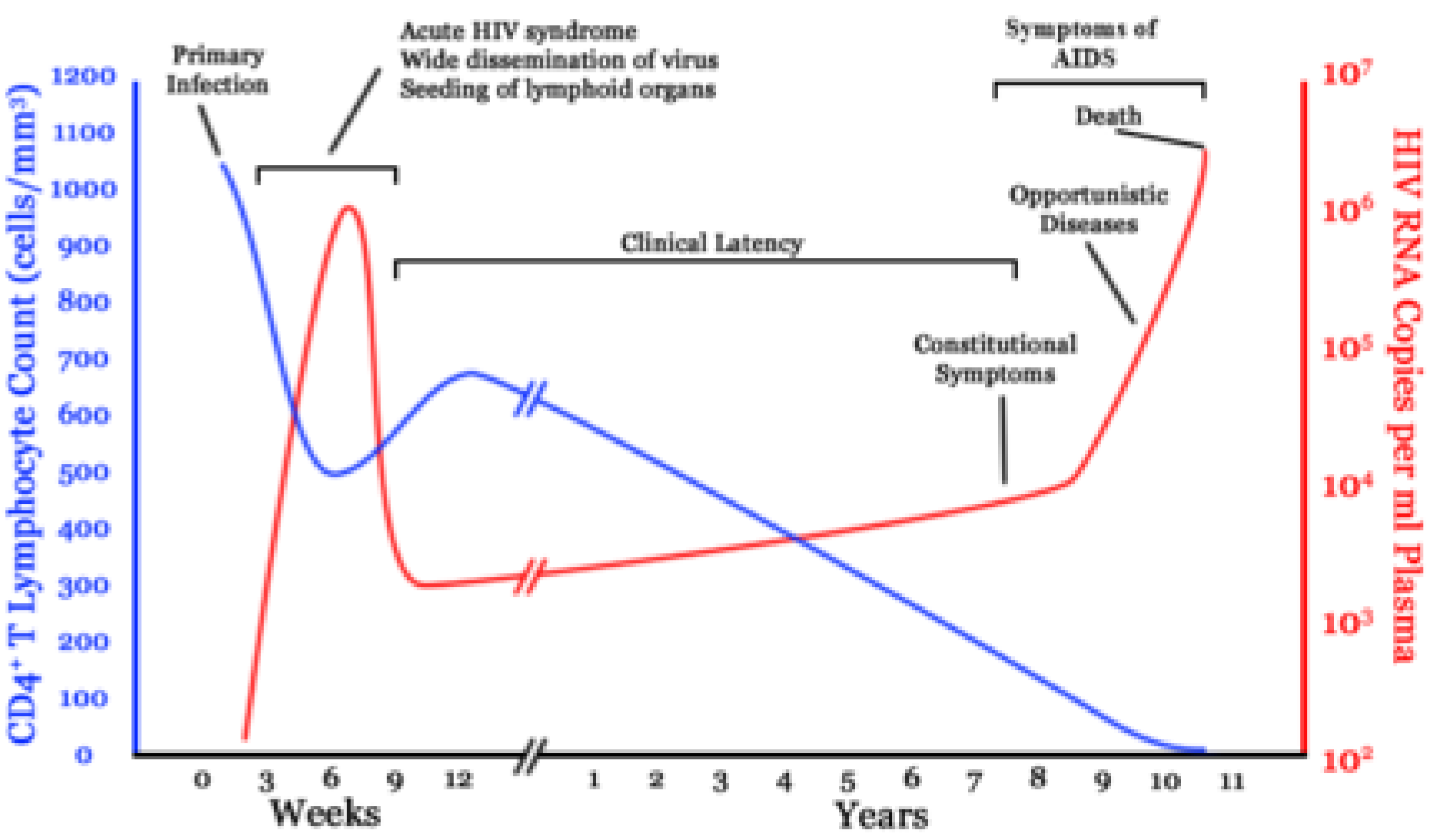

Source: http://en.wikipedia.org/wiki/HIV

Now watch the IrsiCaixa animation entitled *What periods follow HIV infections?* to check you've understood the graph correctly.

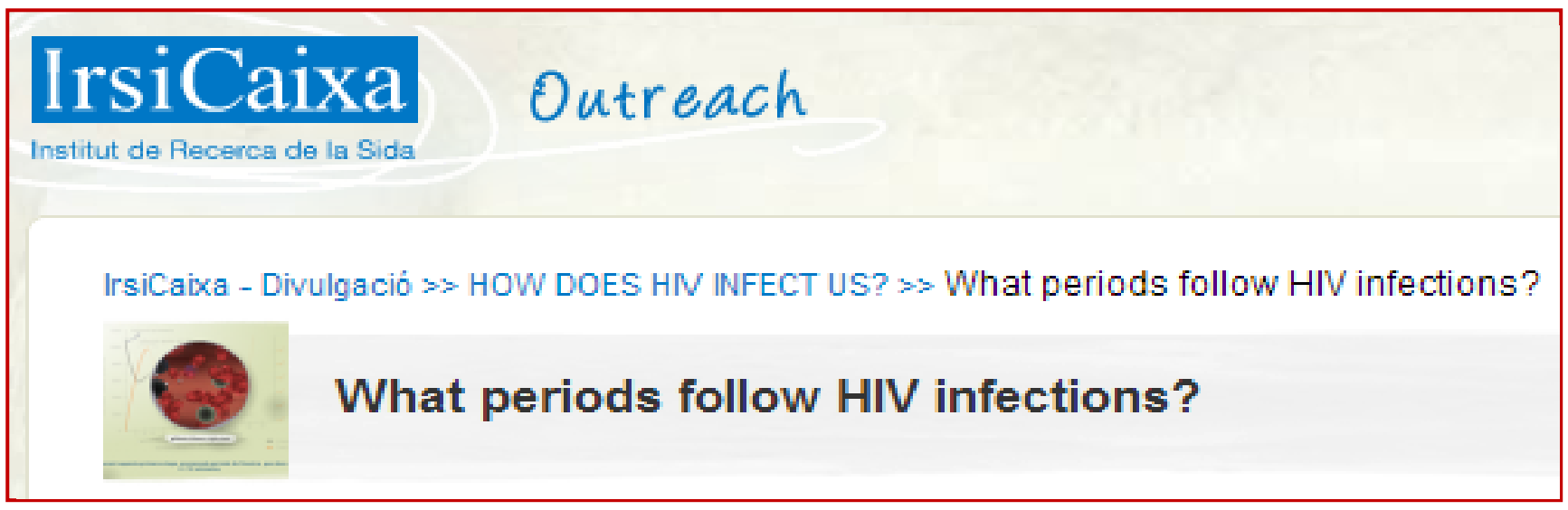

http://divulgacio.irsicaixa.es/en/what-periods-follow-hiv-infections

Fill out the following table:

Phase	Characterised by	Length	Immune response

If you need to, go back and reinterpret the graph.

Experiment

Participate in the research to find a vaccine for HIV.

1. Now activate the virtual experiment:

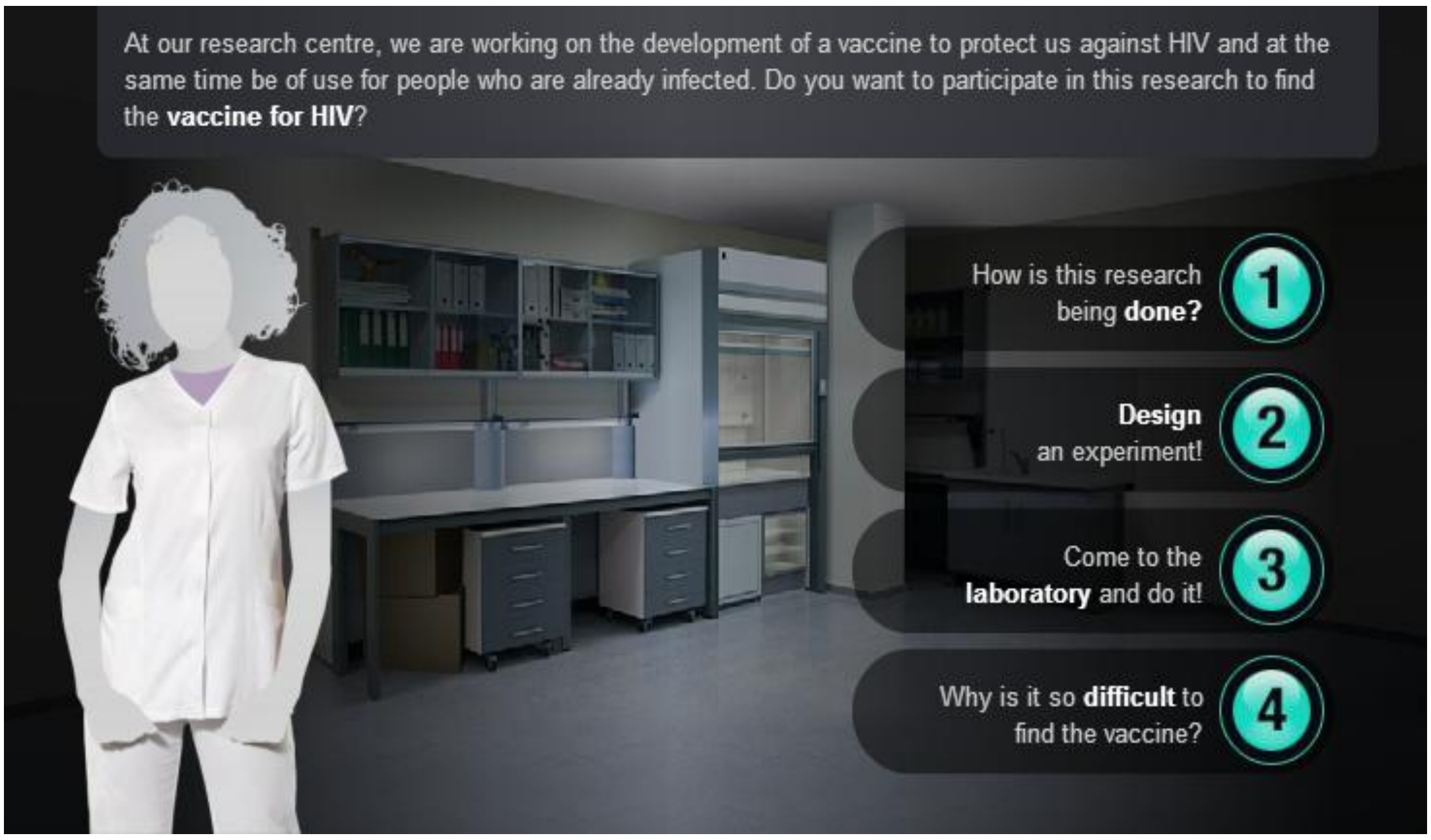

http://www.xplorehealth.eu/en/media/participate-research-find-vaccine-hiv

- Go to part 1, "How is this research being done?" and read all the information. You'll need it to understand the experiment you're about to do.
- Then go to part 3 and follow the instructions to carry out the experiment.
- Once you've finished the experiment, you'll need to write a report. This report will contain your results and conclusions, and should include the following steps:
    - an introduction (brief summary of what the experiment aims to achieve);
    - a working hypothesis (to be formulated as an "if... then..." phrase);
    - the procedure (explain what you're doing in each step and why);
    - an analysis of the results obtained as part of this experiment;
    - conclusions (relate them to your initial aim).

- Now you can write the report:

2. Watch the following videos showing different research projects and answer the questions below:

Joining efforts to find the HIV vaccine

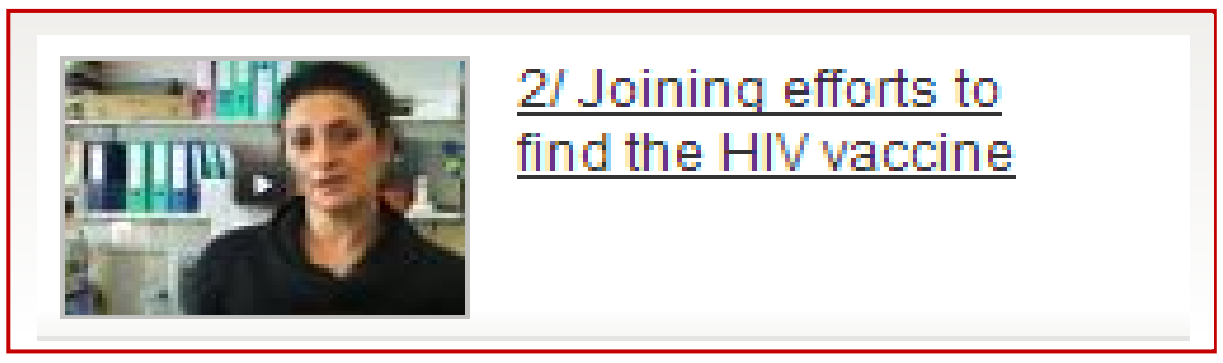

http://www.xplorehealth.eu/en/media/joining-efforts-find-hiv-vaccine

2a. What does a vaccine do once it's introduced into the organism? What do we hope will happen in the case of AIDS?

2b. What does Christian investigate? What project is he working on? What do you think are the advantages of the project?

2c. The investigator uses the example of a pen to explain how a vaccine works. What does the lid represent? And the rest of the pen? What does the red lid that won't cover the pen represent? And what about when the lid *does* cover the pen?

2d. The investigators appearing in this video participate in a Europe-wide project which brings together research from different institutions all across Europe. Why do you think this is important for society as a whole?

New paths against drug resistance

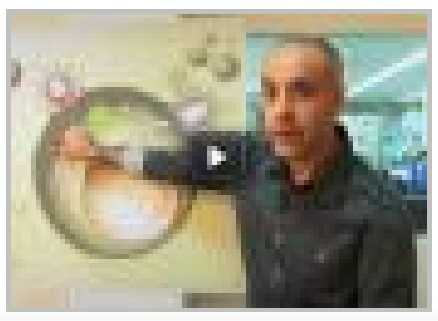

3/ New paths against drug resistance

http://www.xplorehealth.eu/en/media/new-paths-against-drug-resistance

2e. What is the aim of the project which the investigators in this video are working on?

2f. Why have some medicines become less useful due to resistance? What kinds of problems does resistance cause?

Xplore Health

Decide DECIDE

1. Watch the video carefully and pay attention to the information given in it as you will need it to play the *discussion continuum* game basing your answers on scientific fact.

http://www.xplorehealth.eu/en/media/hiv-ethical-issues

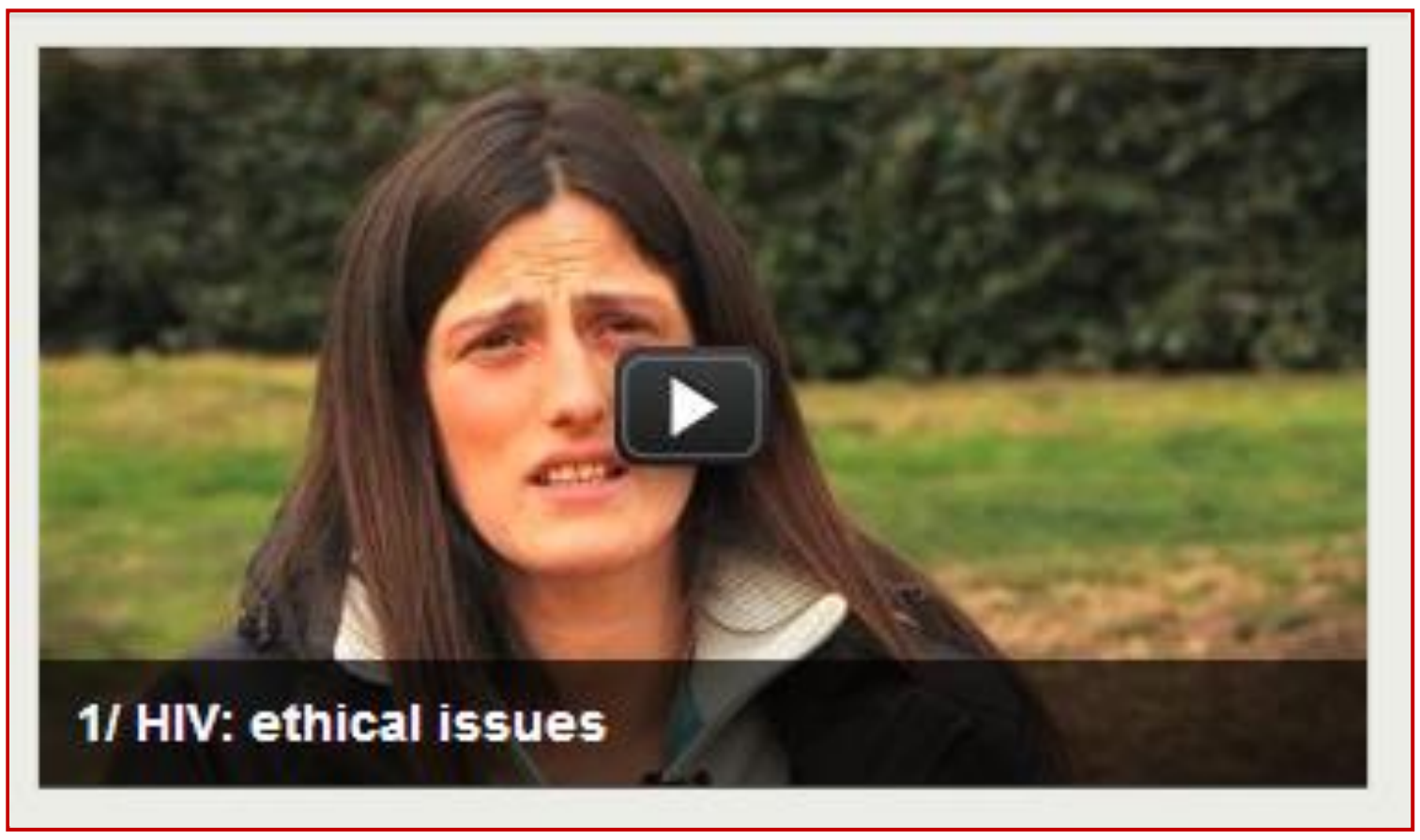

http://www.xplorehealth.eu/sites/default/files/Xplore%20Health%20Discussion%20Continuum%20Game%20HIV%20EN_0.pdf

Discussion Continuum

COMMUNICATE

To finish off, once you've completed your discussion, visit the Xplore Health blog (http://www.xplorehealth.eu/en/xploreblog) and publish the conclusions from your debate.

Afterwards, as part of a prevention plan, design a poster (one per group) which contains useful information on preventing HIV/AIDS infection for the rest of the students in your centre.

www.xplorehealth.eu

Xplore Health

DISCOVER THE LATEST ON HEALTH RESEARCH

eduCaixa

Obra Social "la Caixa"

Authors:
Montse Cabello and Mariona Domènech

Investigate the vaccine for HIV

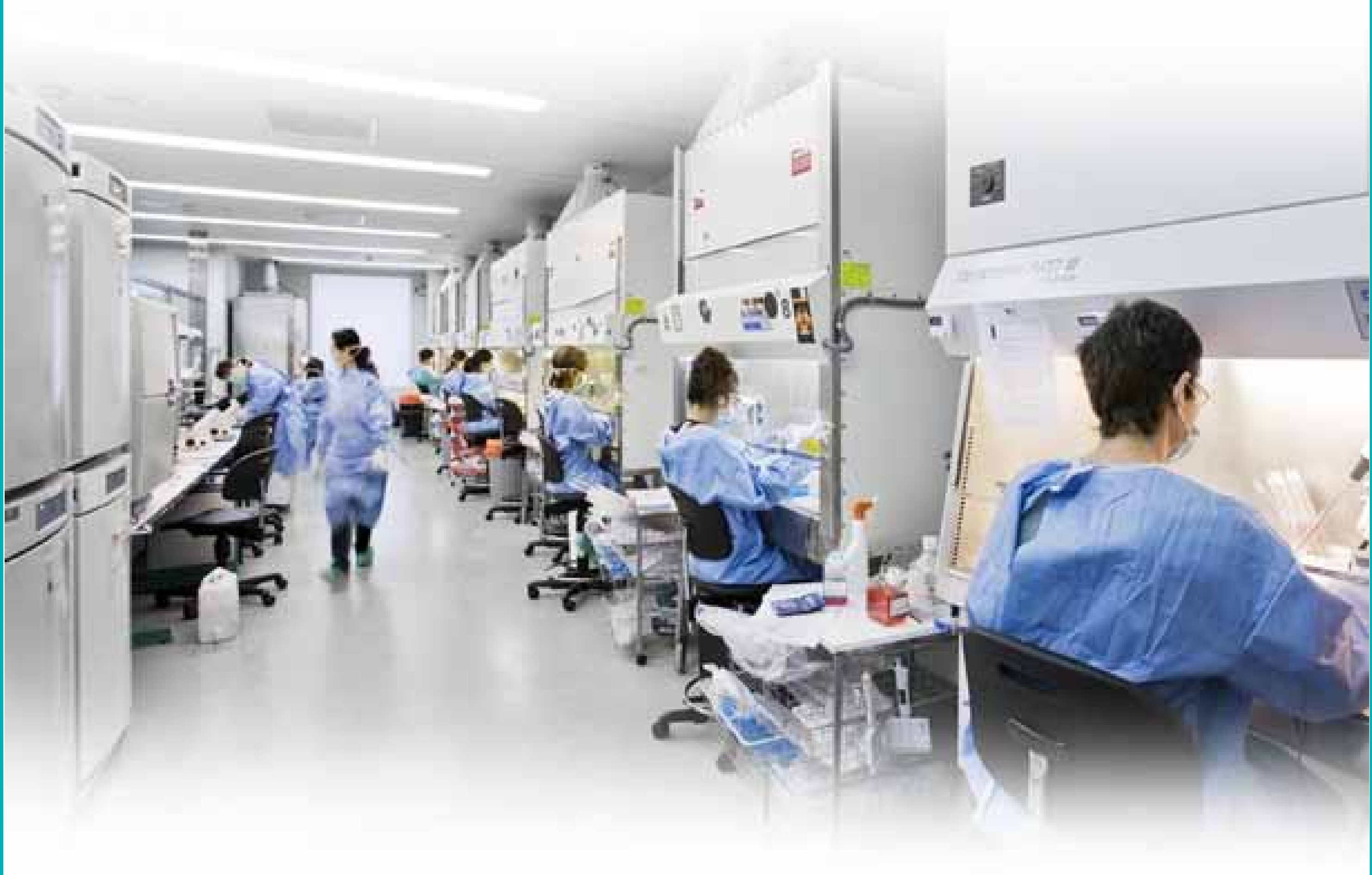

Experimental workshop
PROTOCOL

IrsiCaixa
Institut de Recerca de la Sida

Parc Científic de Barcelona

Obra Social "la Caixa"

Xplore Health

www.xplorehealth.eu

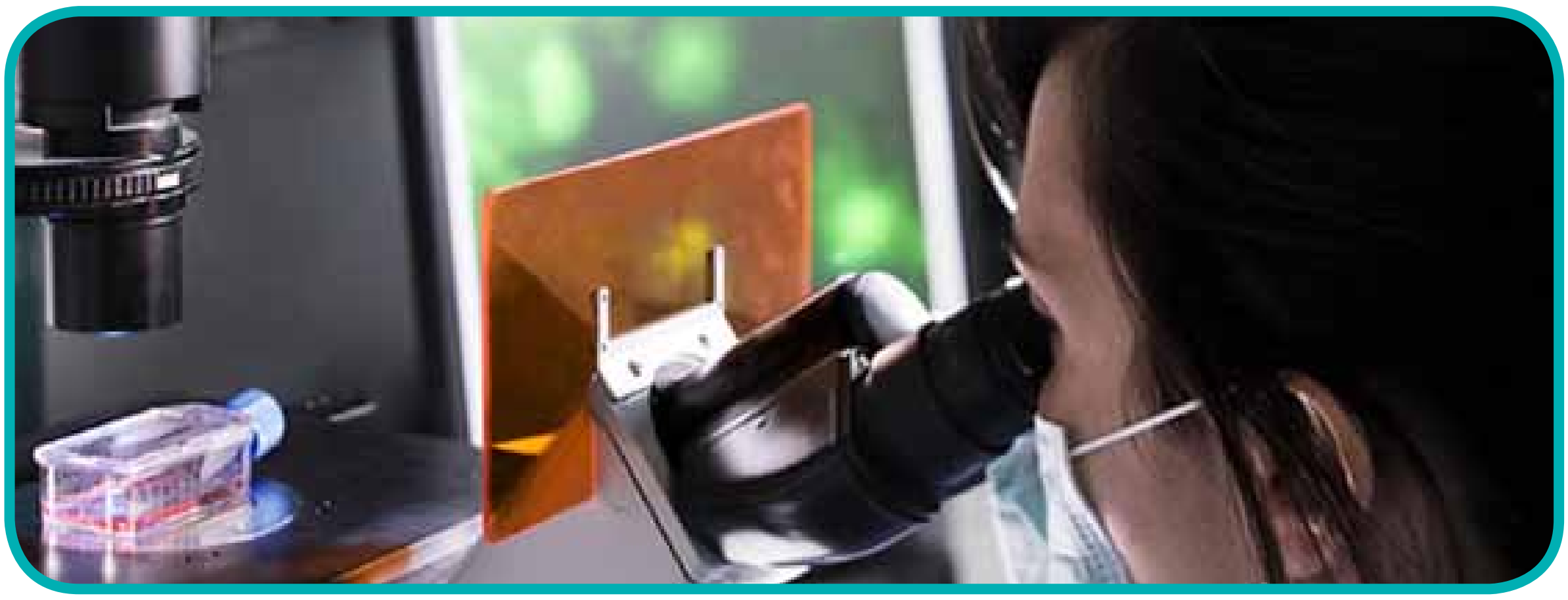

Abstract

The IrsiCaixa Institute for AIDS Research is investigating a vaccine to protect people against the AIDS virus (HIV) and has currently identified a candidate vaccine, which is under development. One of the main difficulties of vaccine development is to overcome the immense variability of the virus in different regions of the world.
In this workshop, you will investigate whether the HIV vaccine developed by IrsiCaixa could be effective in protecting people from different continents.

Introduction

Over 33 million people worldwide are infected with the AIDS virus, or HIV, and every minute six more people become infected. At present, we already have drugs to prolong and improve the quality of life of those infected, but there is no definitive cure and we do not have a vaccine either.

The IrsiCaixa Institute for AIDS Research has been researching for more than 15 years to develop a vaccine and make steps towards improving existing treatments. Meanwhile, the best tool to fight the pandemic is prevention.

A vaccine is a substance that 'teaches' the immune system to recognise viruses or bacteria that cause diseases and defend against them. When we are vaccinated, our immune system recognises the vaccine agents as foreign and triggers a response against them. That way, when the germ infects us, our immune system is ready to respond.

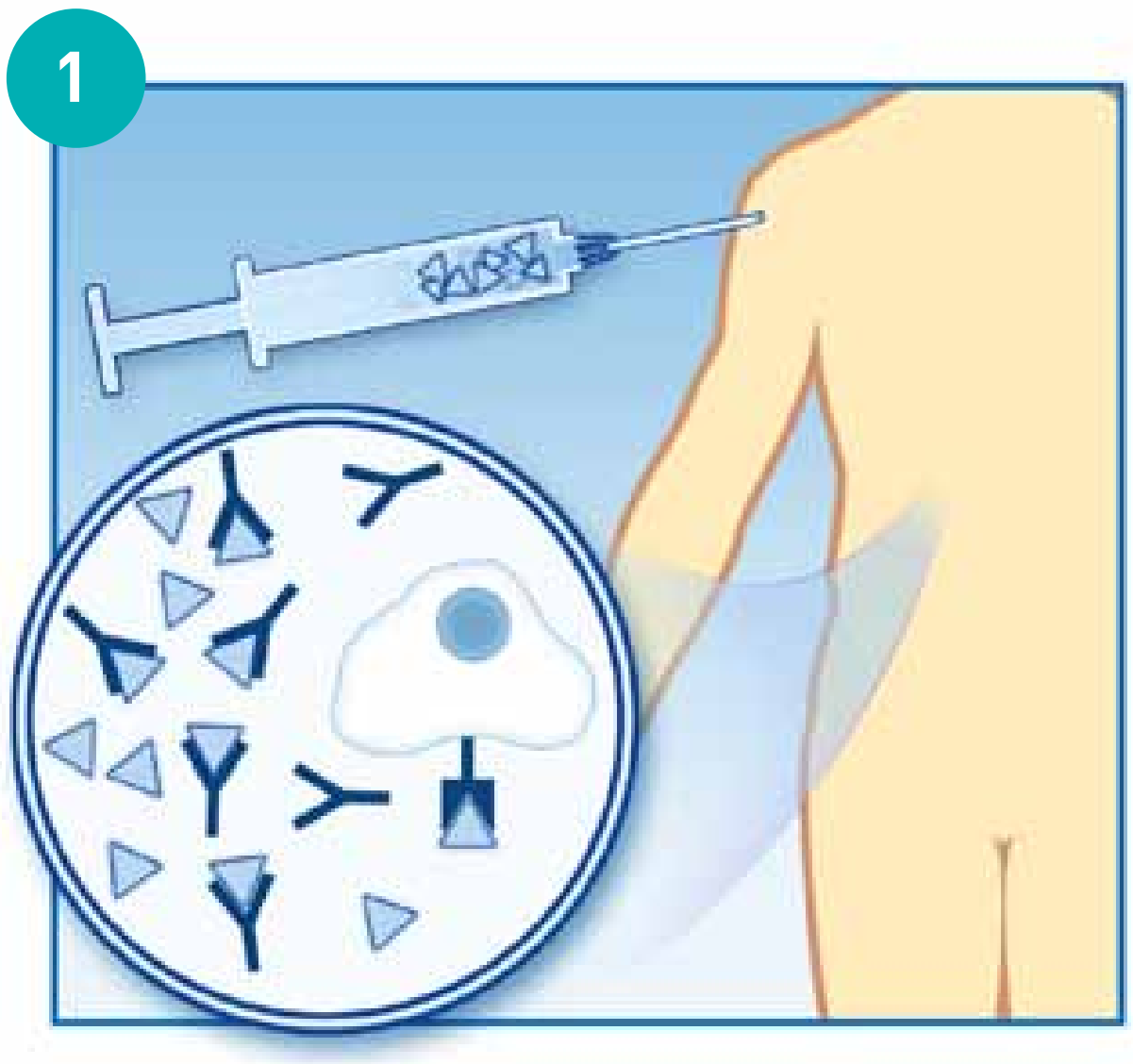

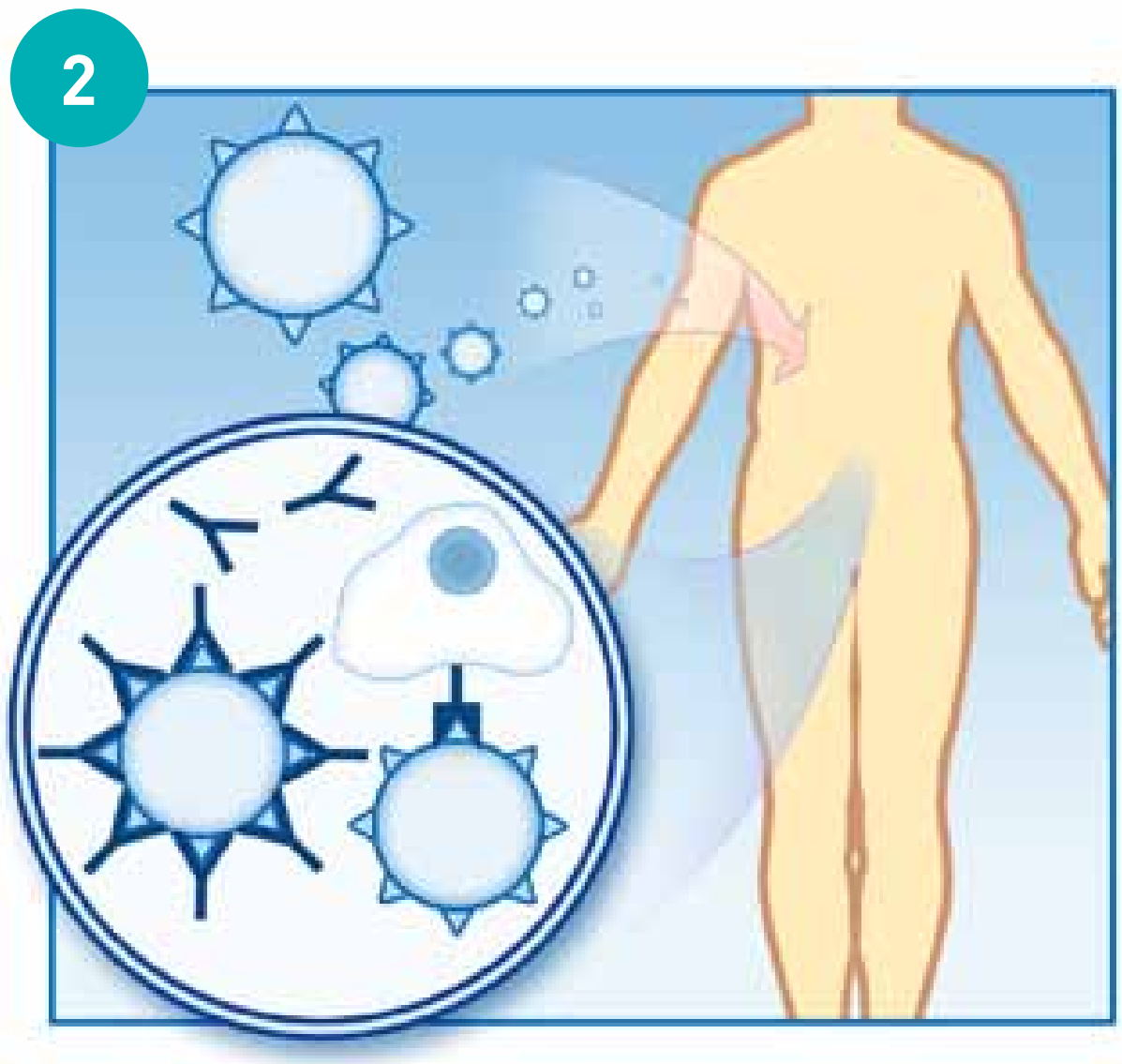

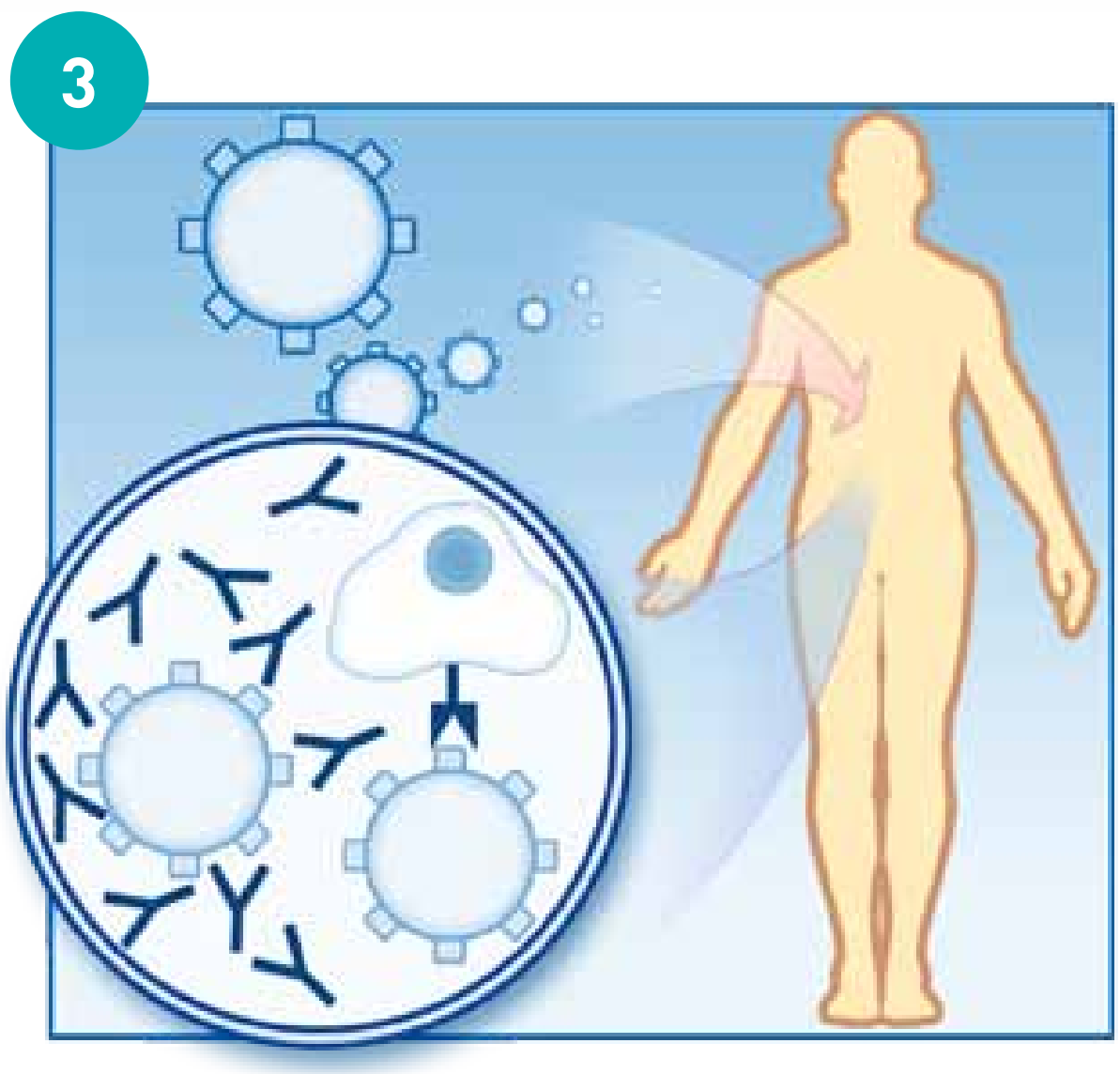

1. The vaccine candidate protein stimulates the immune system to produce antibodies against it

2. If one day the vaccinated person becomes infected, they will have antibodies to fight HIV

3. However, if one day the vaccinated person becomes infected with HIV that has a different protein, the antibodies generated by the vaccine will not be useful in fighting the infection

The difficulty in developing an HIV vaccine is to find a vaccine that is capable of activating the immune system to completely eliminate the infection caused by the millions of types of HIV that exist around the world.

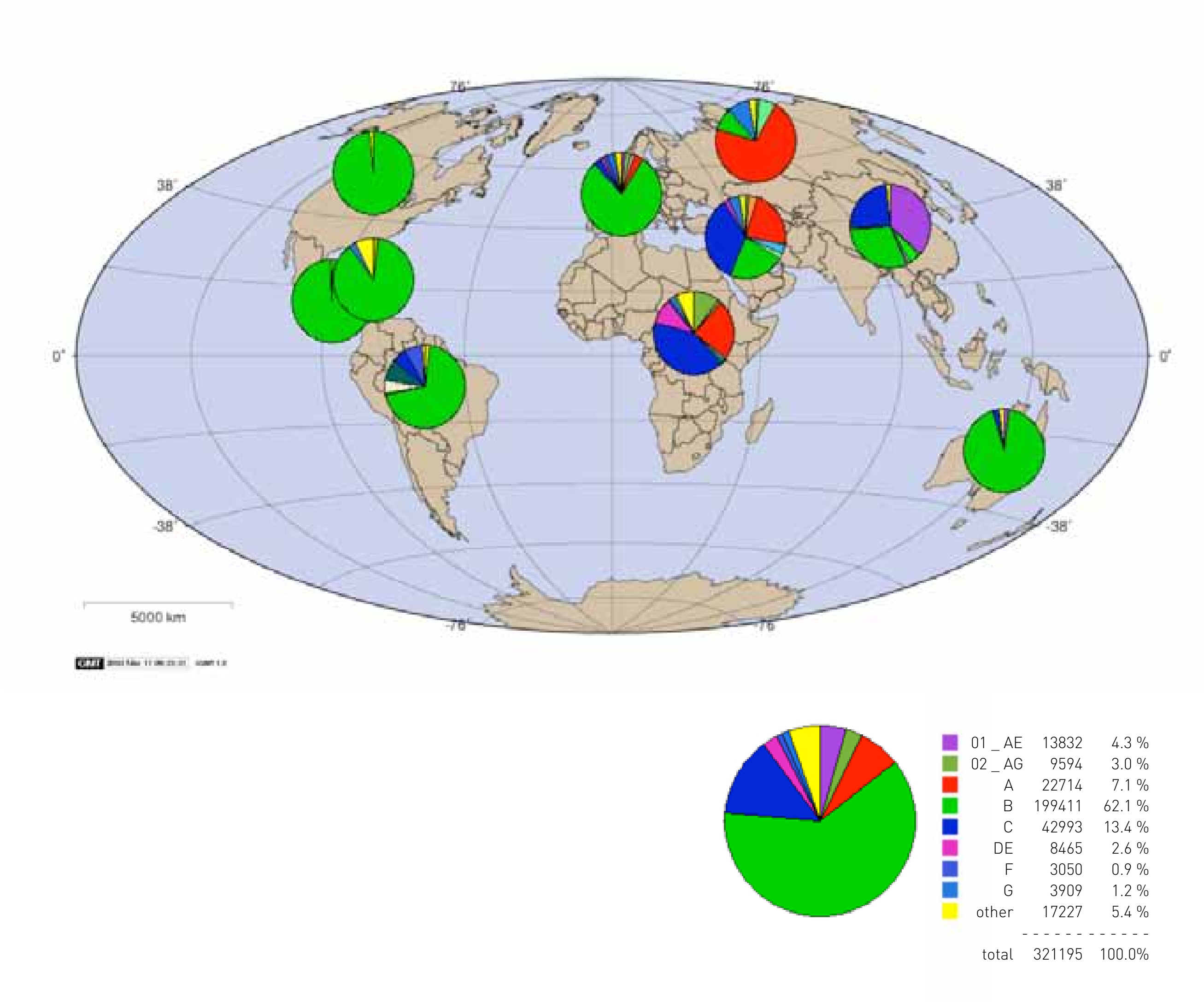

Distribution of the different variants of HIV-1 worldwide. (Los Alamos HIV sequence Database, www.hiv.lanl.gov)

How can you participate in the search for an HIV vaccine?

In this workshop you will be able to investigate an HIV protein that IrsiCaixa has identified as a possible component of a vaccine to protect people against this virus. It has been shown that in vitro this protein stimulates the immune response.

Currently, IrsiCaixa is conducting studies in vitro to assess its effectiveness and, if all goes well, more advanced stages of research will begin afterwards, culminating in tests with patients.

The aim of the workshop will be to determine whether the IrsiCaixa candidate vaccine will be effective in protecting people against the great variety of types of HIV in different regions of the planet. Will we have to develop different vaccines for different parts of the world?

To investigate this, you will have to analyse whether this protein is present in HIV variants from Africa, Asia and Europe.

If present, the vaccine could be effective in preventing infection with these HIV variants, as it would stimulate an immune response against them. Therefore, it might be effective in protecting the population of these continents.

But if any of the variants does not contain the candidate protein, then the vaccine would not be effective against this strain of HIV and would have to be modified.

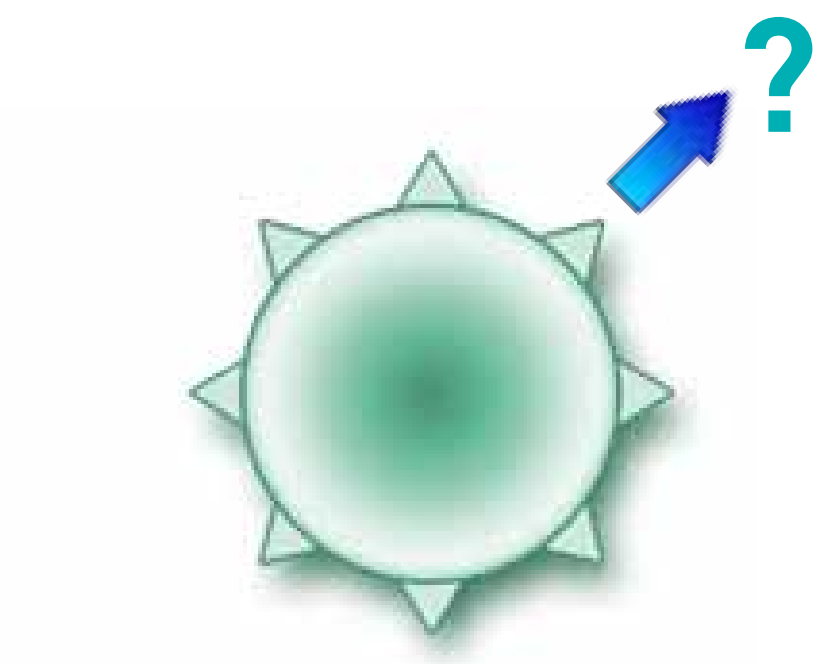

African virus

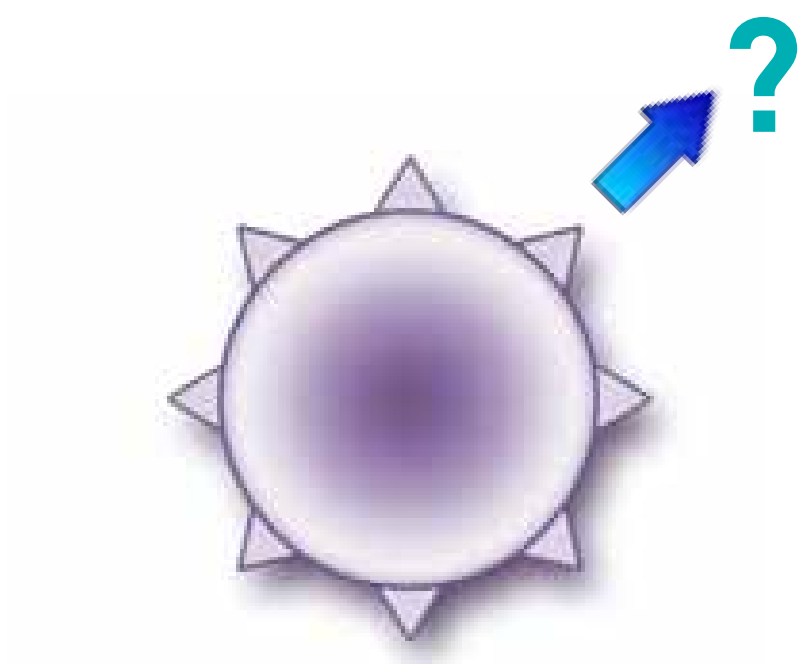

Asian virus

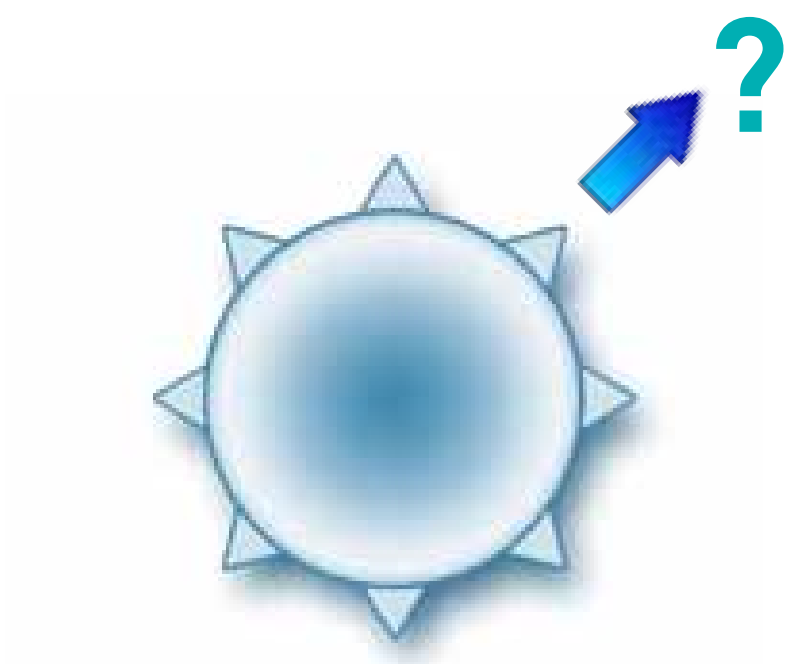

European virus

Objectives

- To participate in the development of the IrsiCaixa AIDS vaccine, following the steps of the scientific method.
- To use laboratory techniques used in this research field, such as the separation of DNA fragments by electrophoresis or DNA cleavage using restriction enzymes.
- To investigate and become familiar with the immense variability of the AIDS virus.
- To check whether the protein IrsiCaixa has identified as a possible vaccine candidate could be useful in stimulating the immune system of people exposed to the majority of HIV variants from different continents: Africa, Asia and Europe.

Hypothesis

The protein that is a candidate to become part of a vaccine identified by IrsiCaixa is not modified in the vast majority of HIV variants that exist all over the world. Therefore, a vaccine with this protein should be equally effective against the majority of the HIV variants that exist.

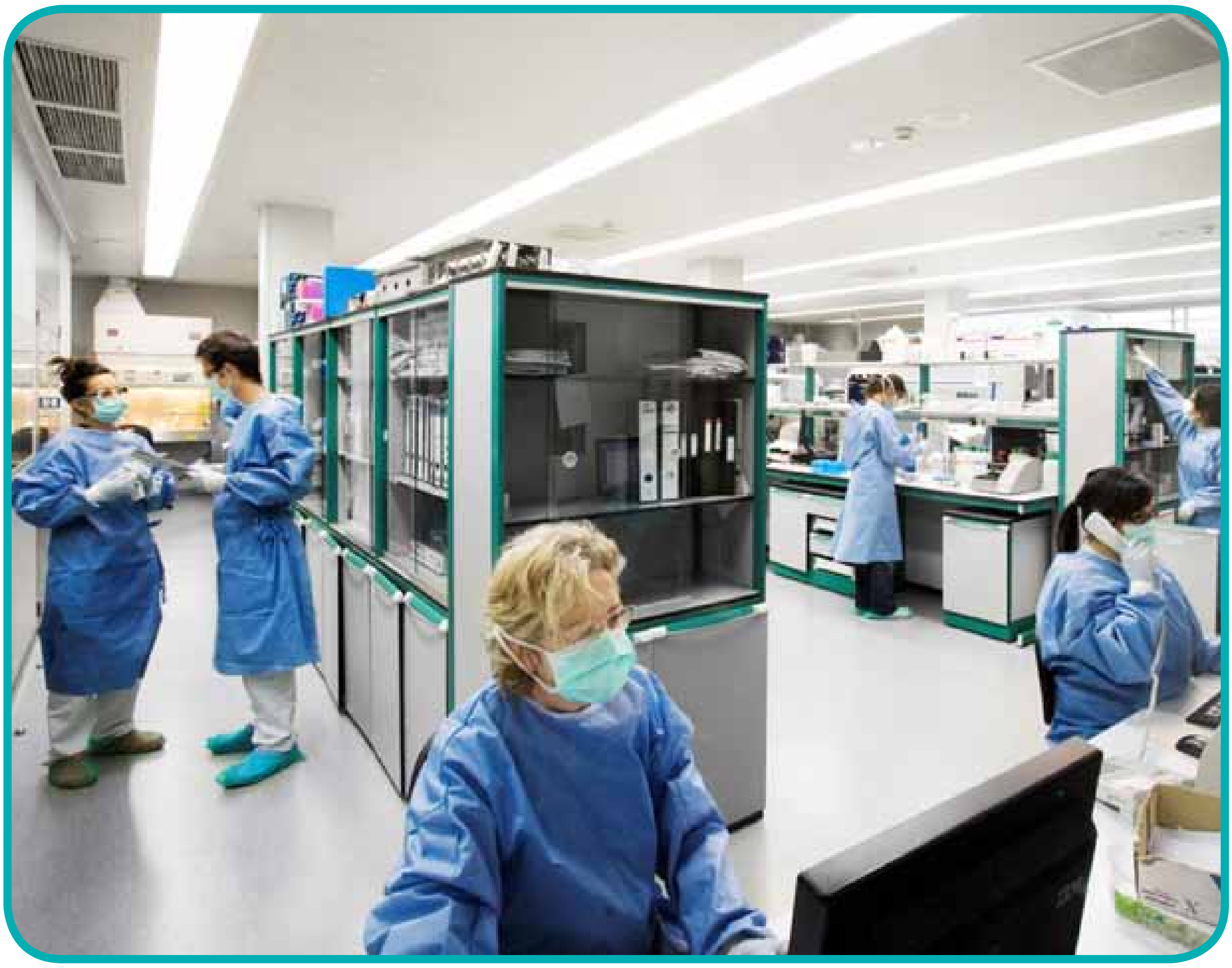

Methodology

We will work with DNA samples from the three most common HIV variants found in three continents: Africa, Asia and Europe.

We will also have a DNA sample containing the information needed to produce the IrsiCaixa vaccine candidate protein. With these samples you will investigate whether the different HIV variants contain the candidate DNA sequence.

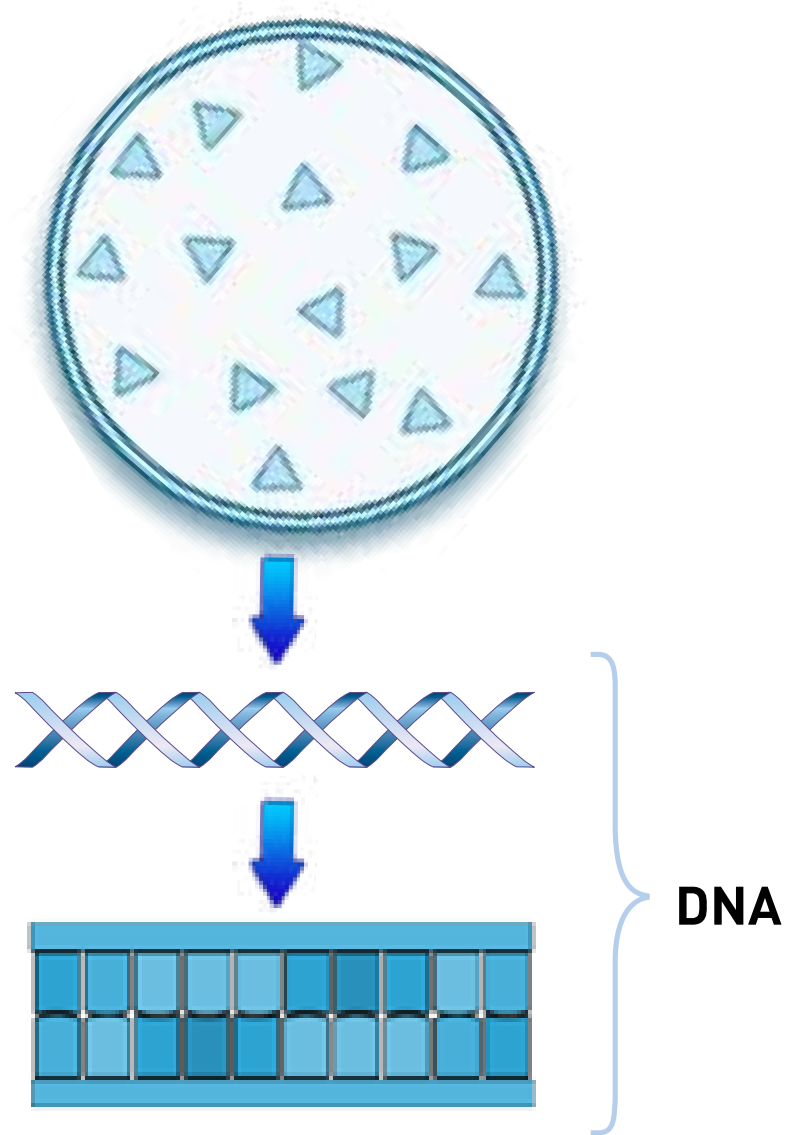

Candidate protein

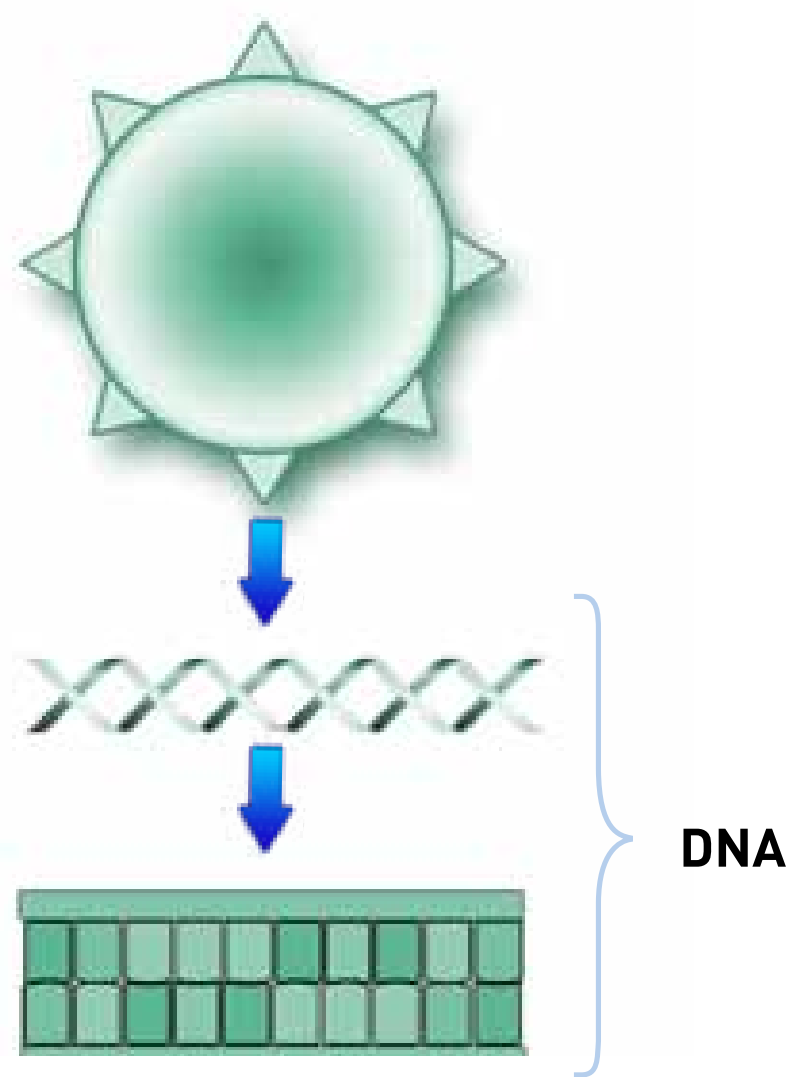

Most common European HIV variant

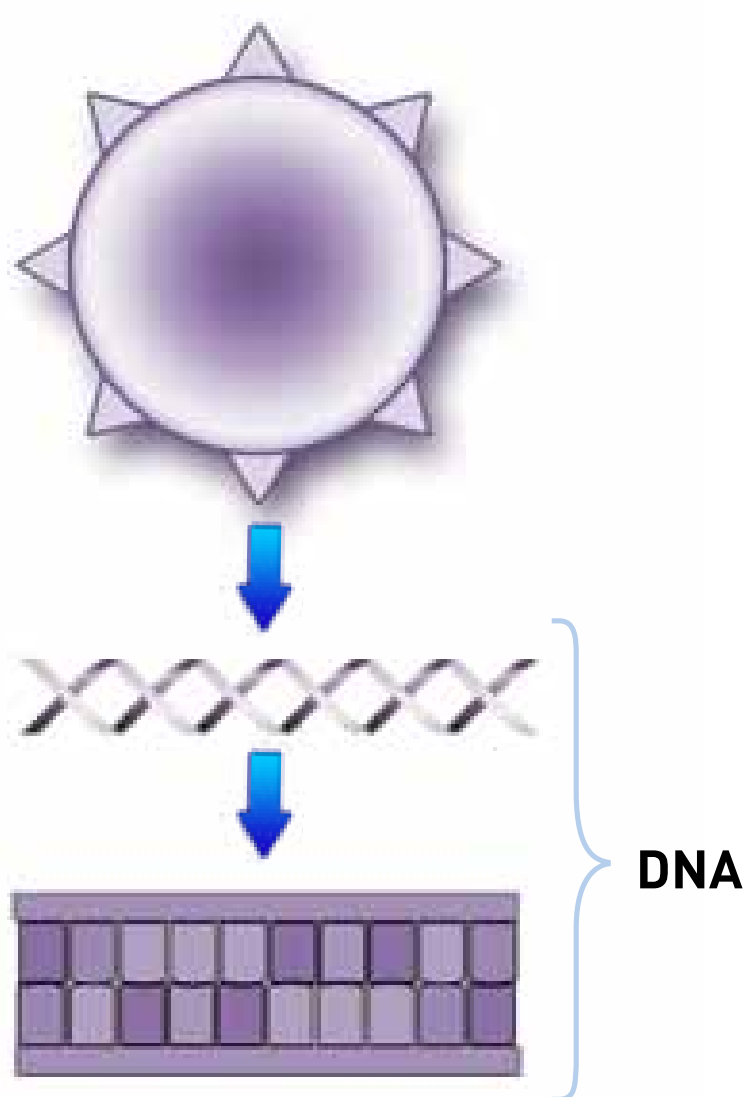

Most common African HIV variant

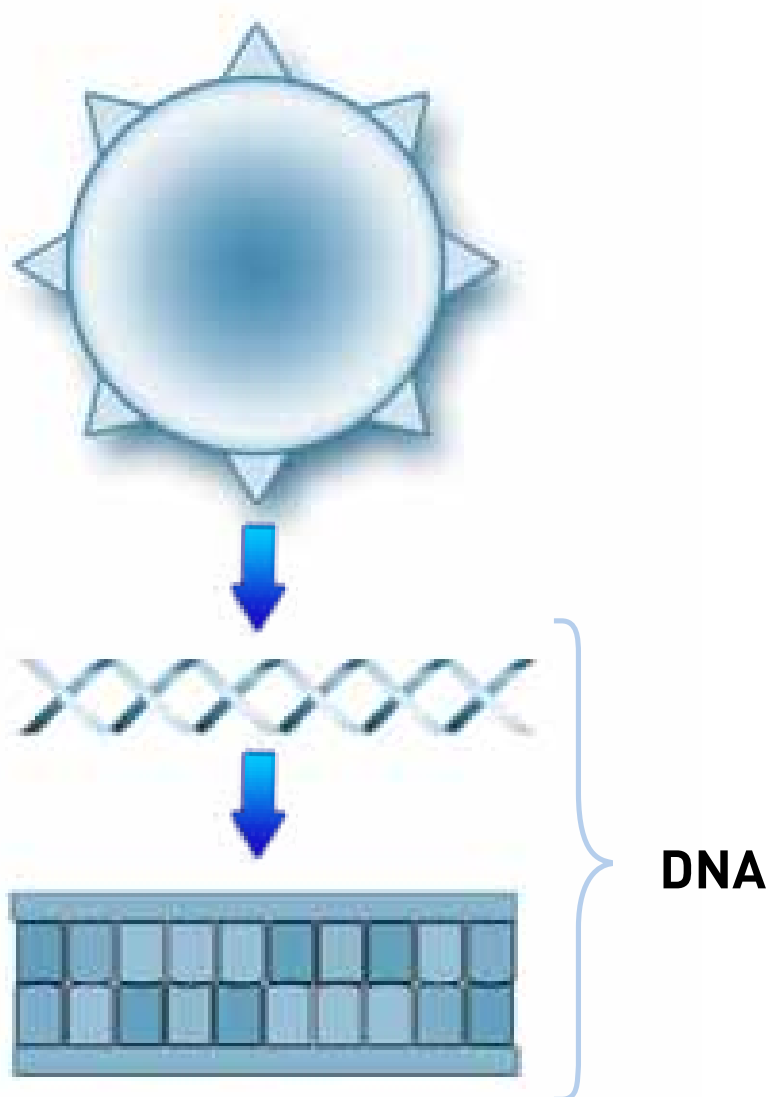

Most common Asian HIV variant

To carry out this research we will perform the following tasks:

(A) We cut the DNA strands of the viruses from the three continents: To do this, we will use restriction enzymes, proteins known to researchers for their ability to cut the DNA at specific sites. The enzyme which we will use specifically cuts the gene responsible for synthesising the candidate protein, but does not cut it if it shows some variation in the DNA region being studied. Therefore, DNA samples are only cut if this gene is conserved.

(B) We separate the DNA fragments: With the aid of a technique called electrophoresis, we separate the DNA fragments resulting from digestion by the enzymes.

(C) We visualise the DNA fragments: We visualise what DNA samples are cut and, therefore, which variants have the candidate protein.

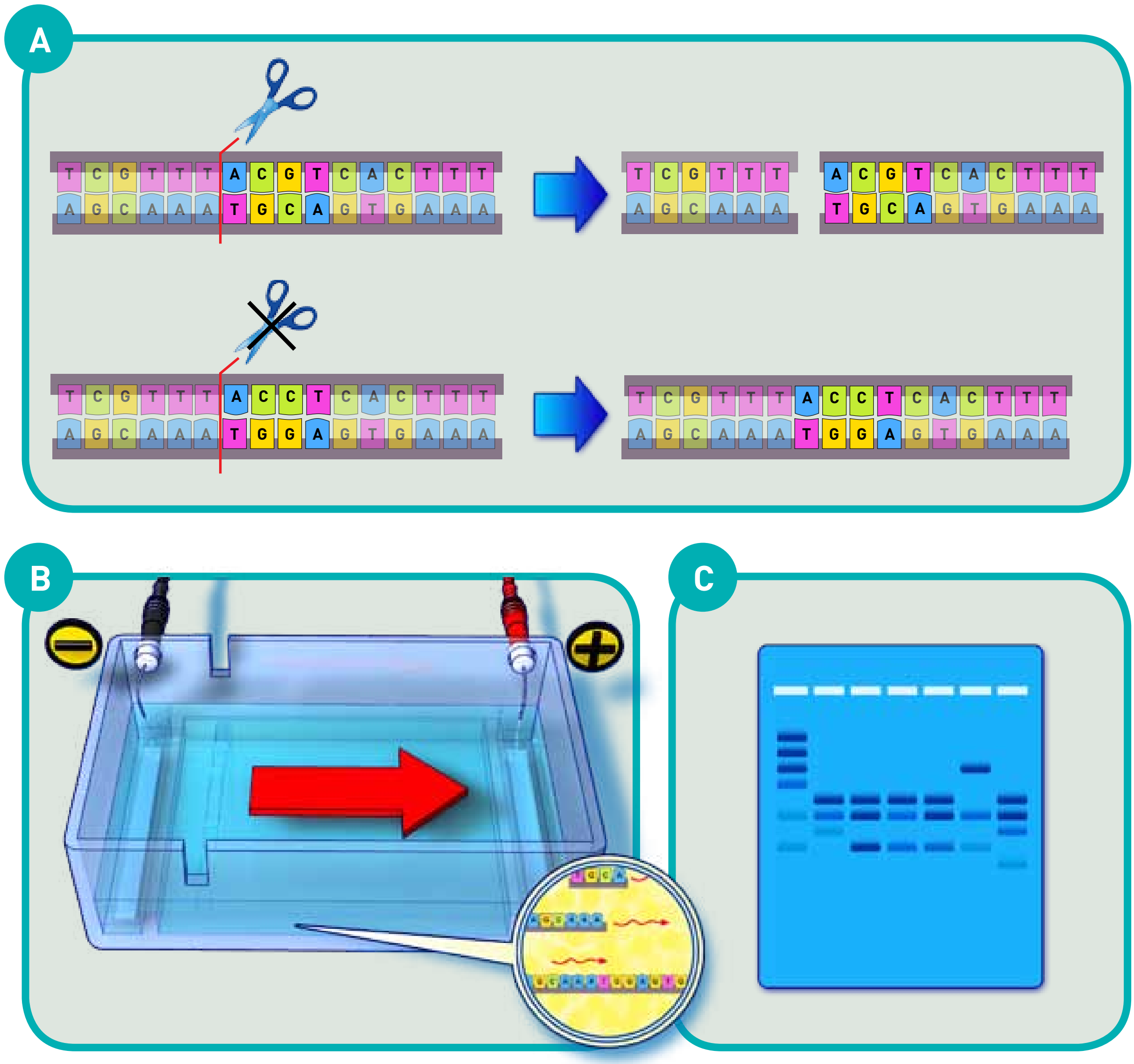

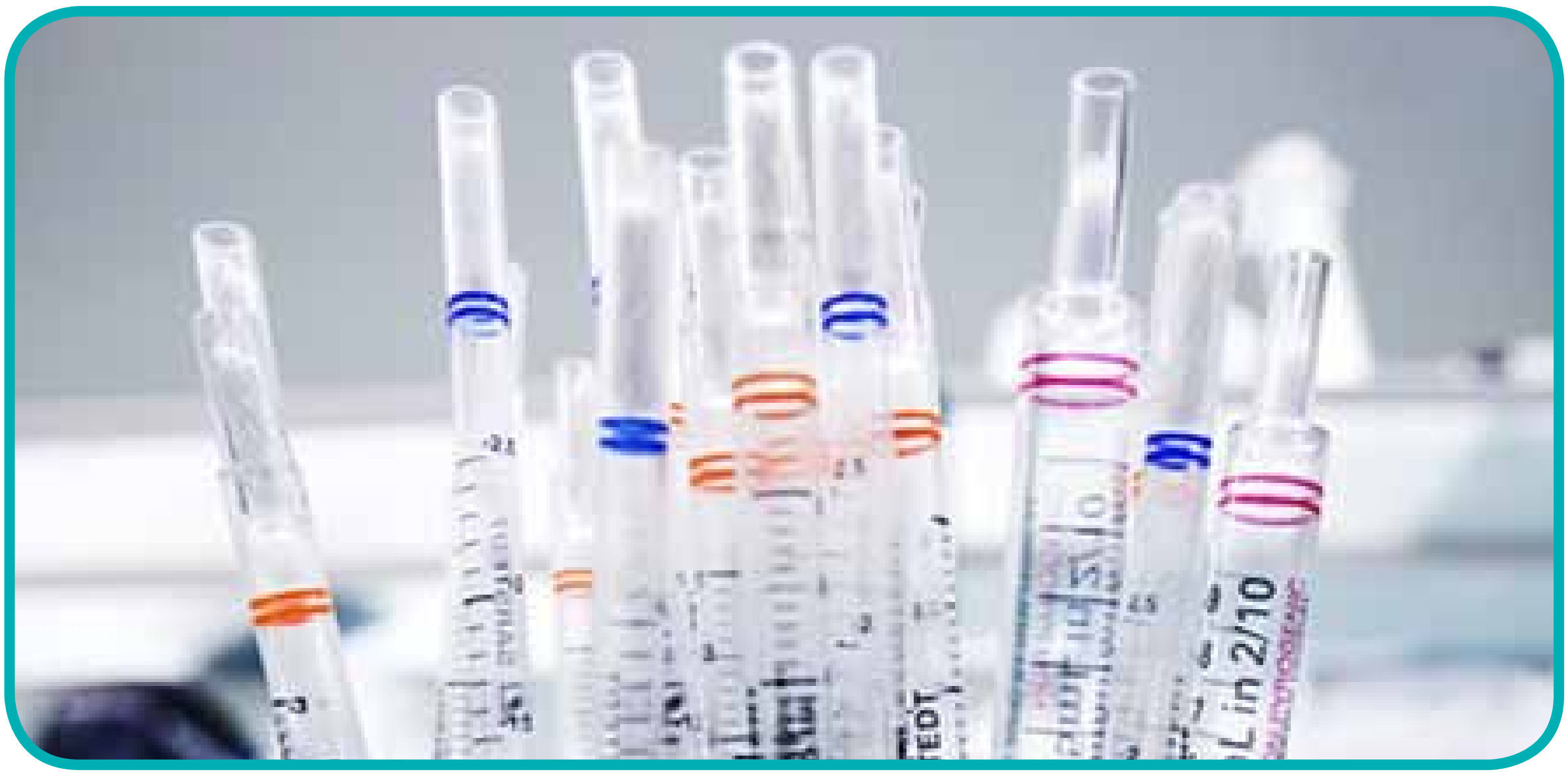

Results and conclusions

We will compare the genetic sequences of the different HIV variants, analysing the effect of the restriction enzyme, and thus identifying the differences in their DNA. With this information, we will be able to reflect on the immense variability of HIV.

With the results obtained we can conclude whether the IrsiCaixa candidate protein might be useful for protecting people from different continents, or whether different vaccines will need to be developed, each tailored to the sequence of the virus present in each geographic area.

Instruments and materials required

Laboratory instruments and material

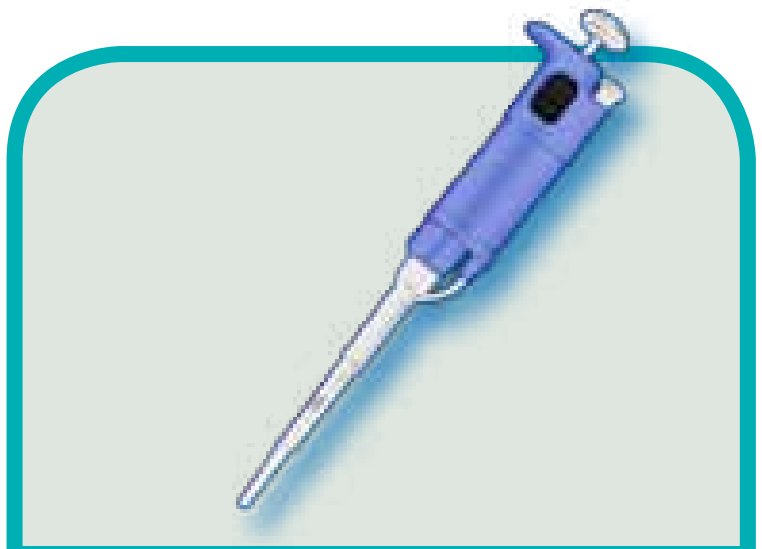

10 and 20 µl pipettes

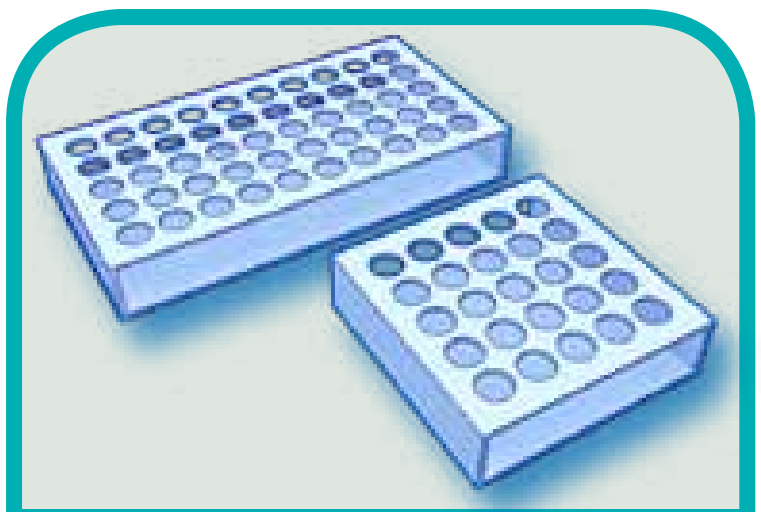

Racks

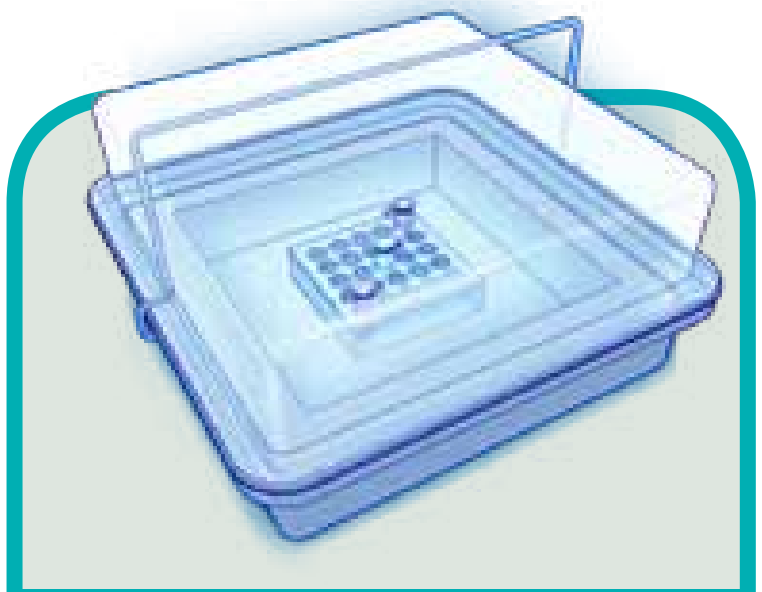

Cooler

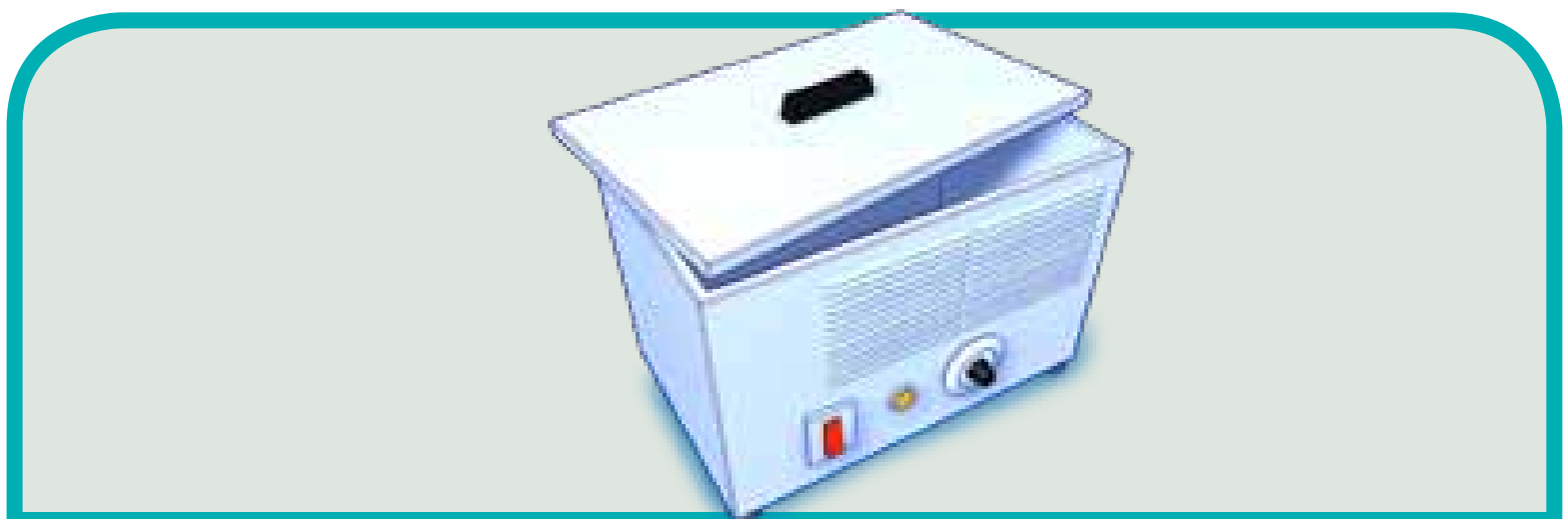

Water bath and support for the tubes

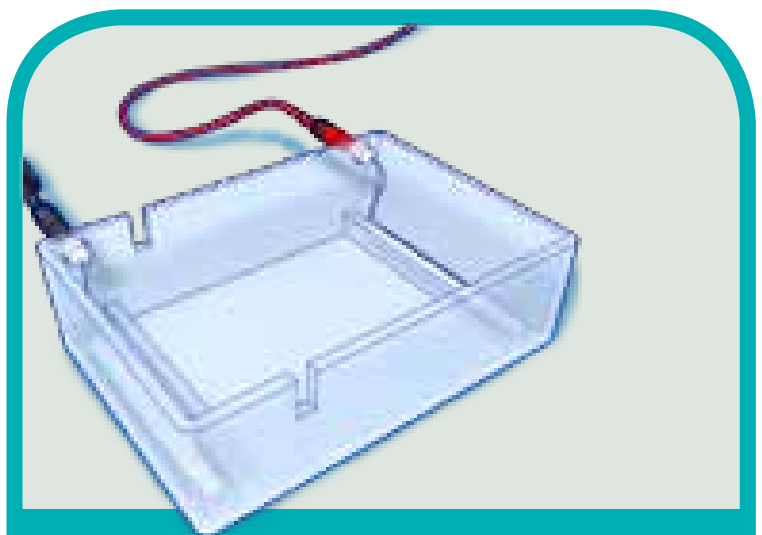

Electrophoresis chamber

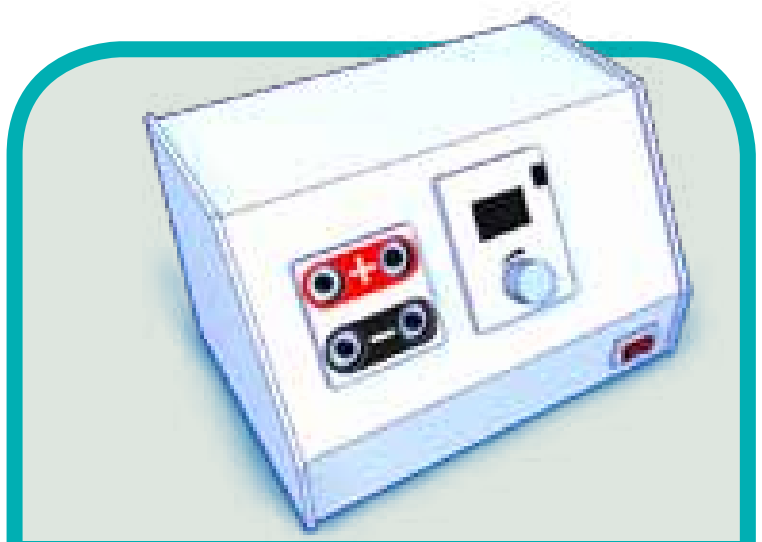

Power supply

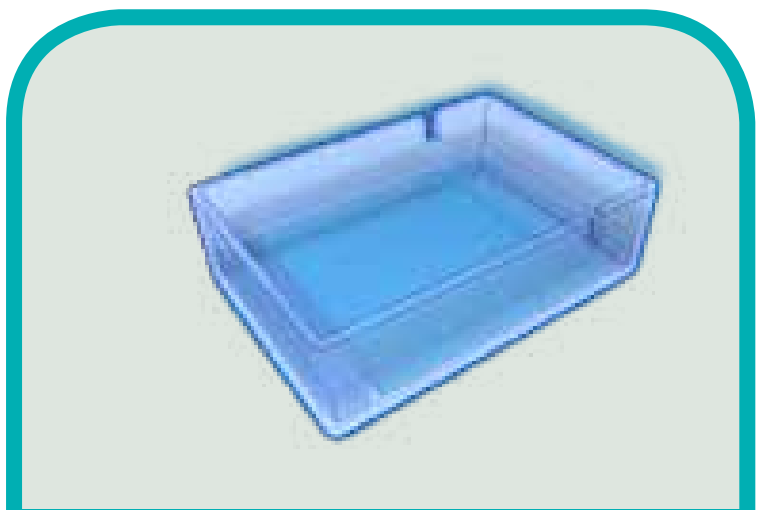

Agarose gel

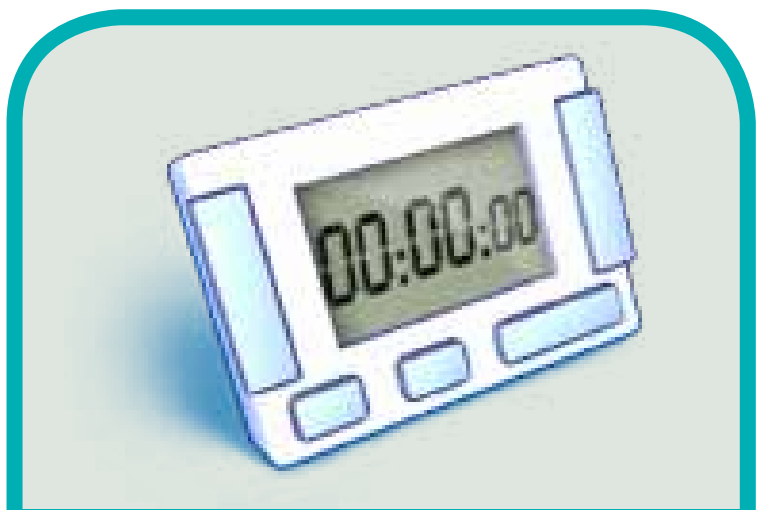

Timer

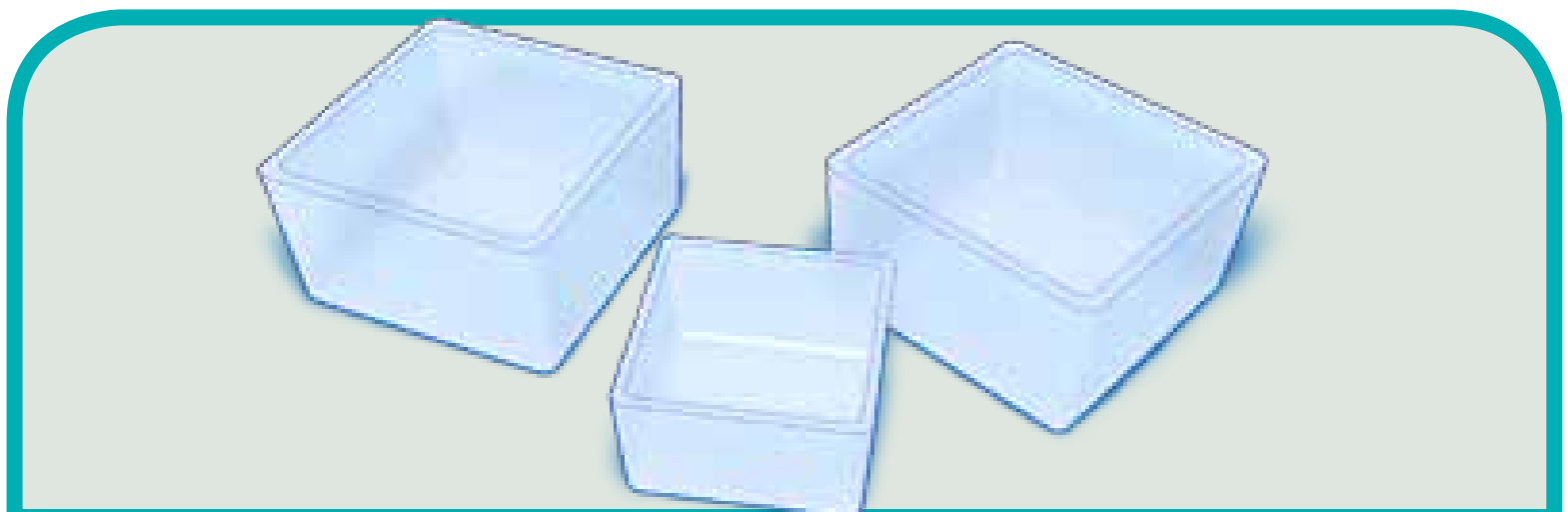

3 containers (to stain and clean the gel)

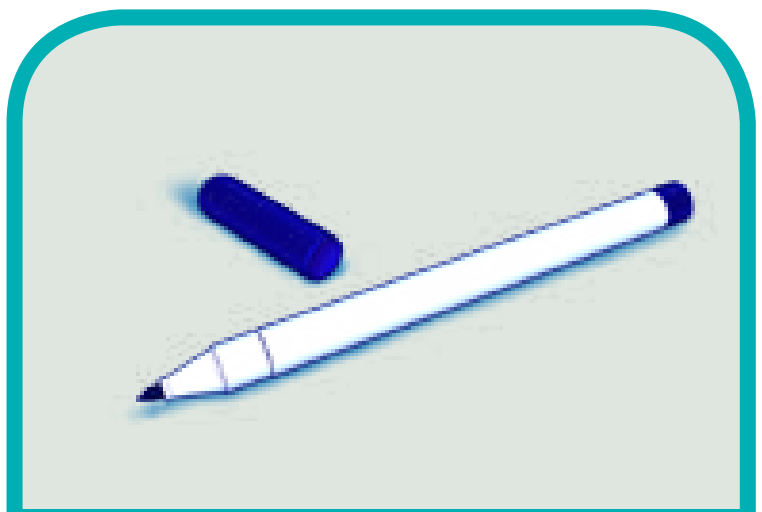

Glass marker

Consumables

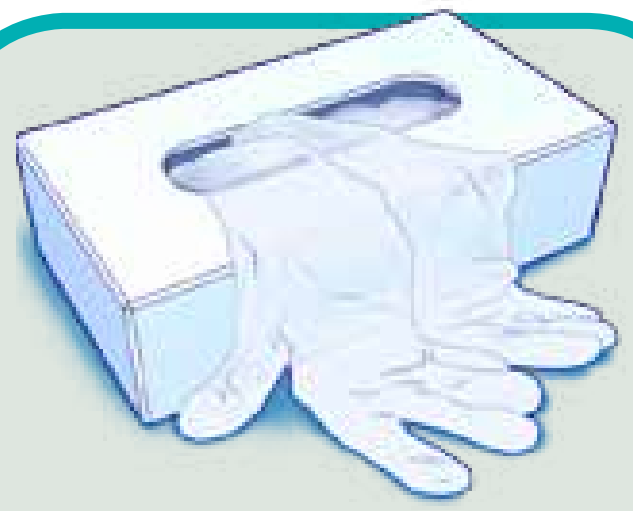

Gloves and lab coats

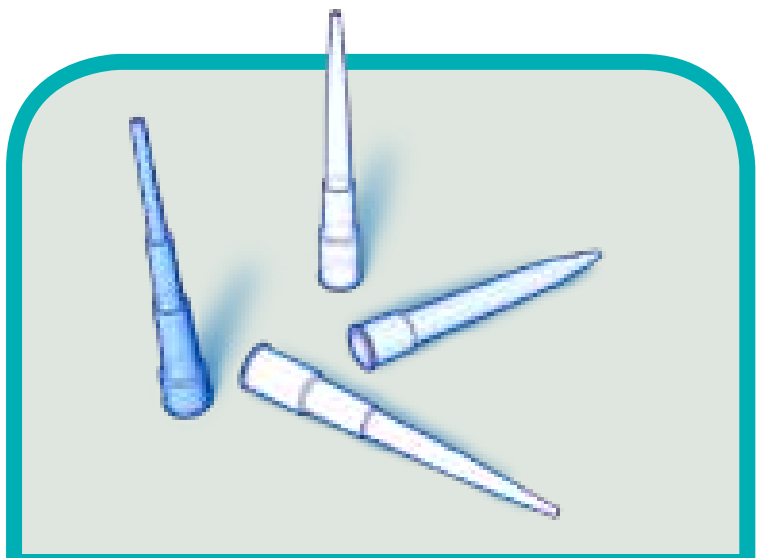

Pipette tips

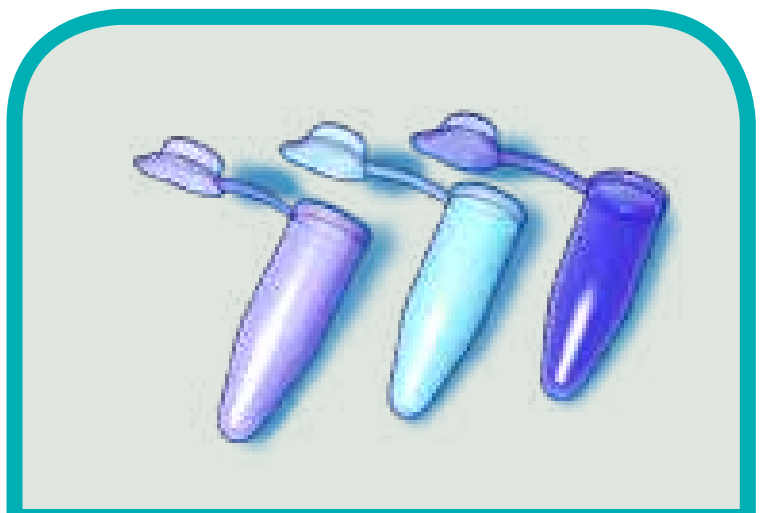

1.5 ml and/or 0.5 ml tubes

Reagents and solvents *

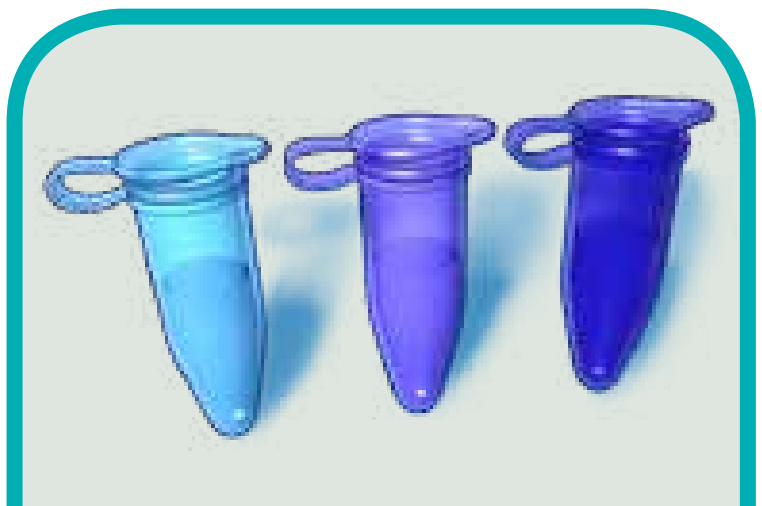

Virus DNA from different sources

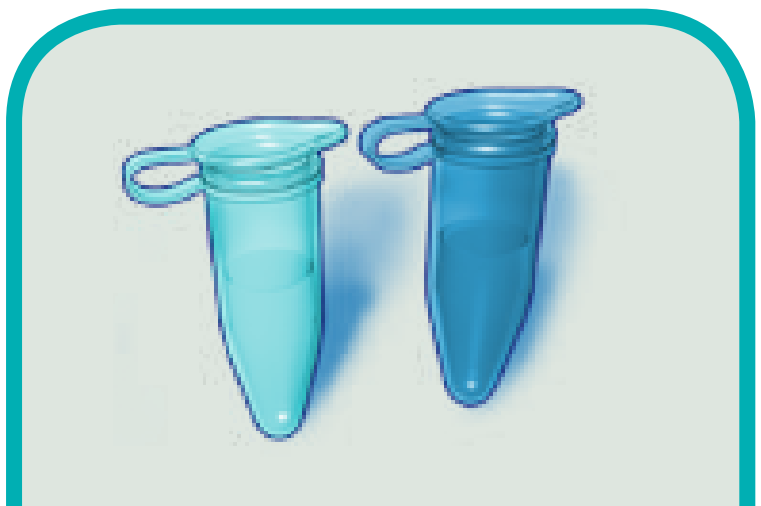

NheI restriction enzyme + buffer (1)

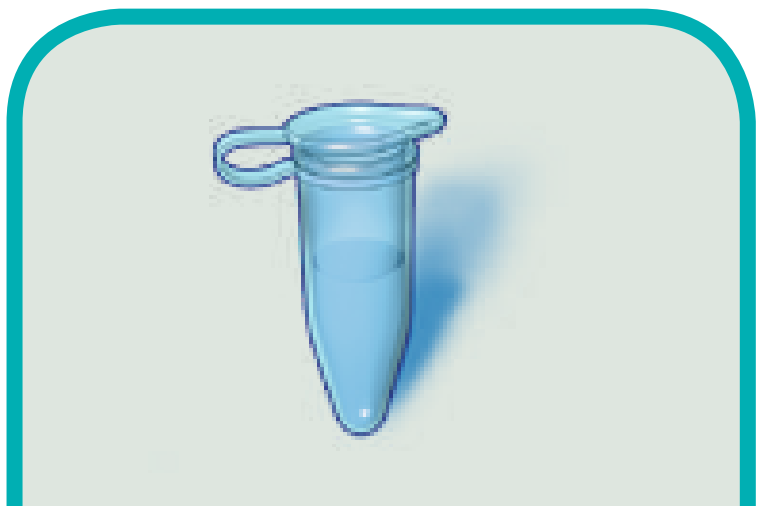

Molecular weight marker (2)

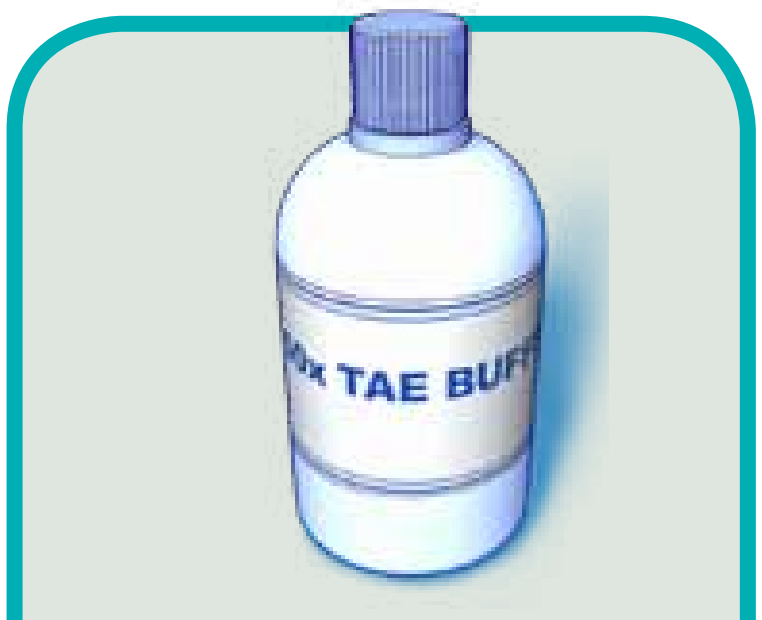

TAE buffer
500 ml of 0.5x TAE (3)

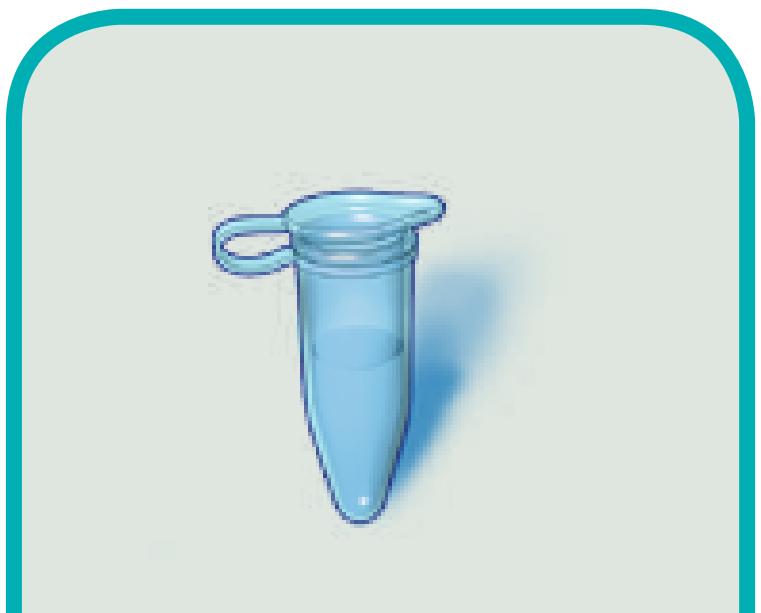

Loading buffer (4)

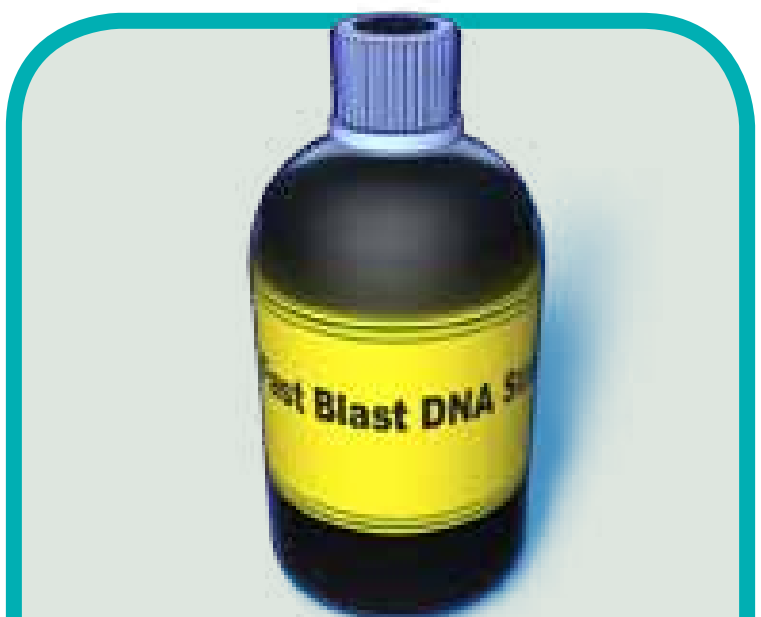

Liquid to stain the DNA (Stain Fast 100x)

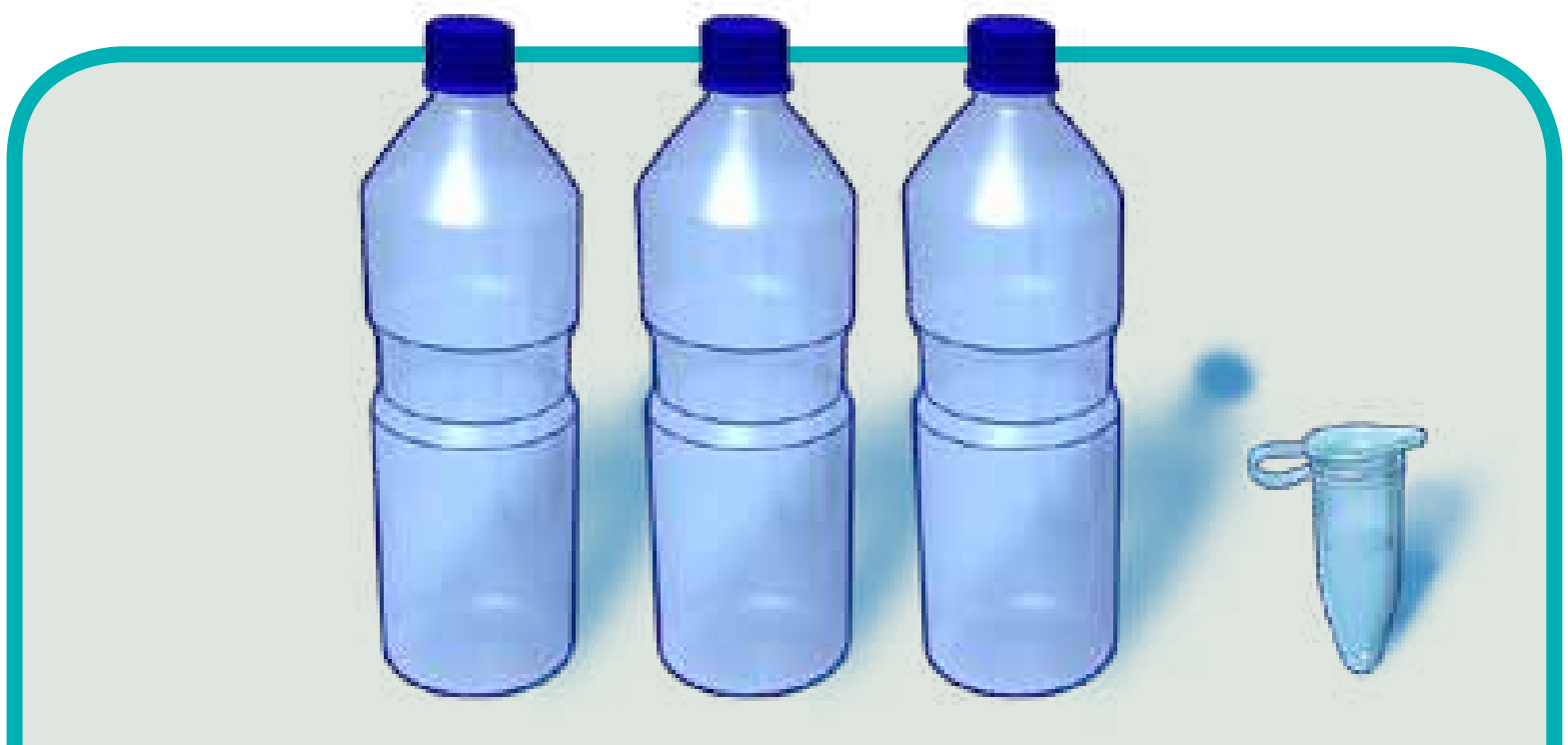

Distilled water: 3 x 500 ml glass flasks and one 1.5 ml tube.

(1) The buffer is used to obtain a suitable medium for the enzyme to act.

(2) The marker has a label which states: measures of the different fragments of between 100 and 3,000 base pairs.

(3) To maintain the pH, it also contains salts to facilitate the passage of the electrical current (in 0.5x concentration to fill the chamber).

(4) To give colour and density to the samples and therefore make them easier to load.

*If you want to do this experiment in your school or museum, get in touch with divulgacio@irsicaixa.es so that they can provide you with the necessary reagents.

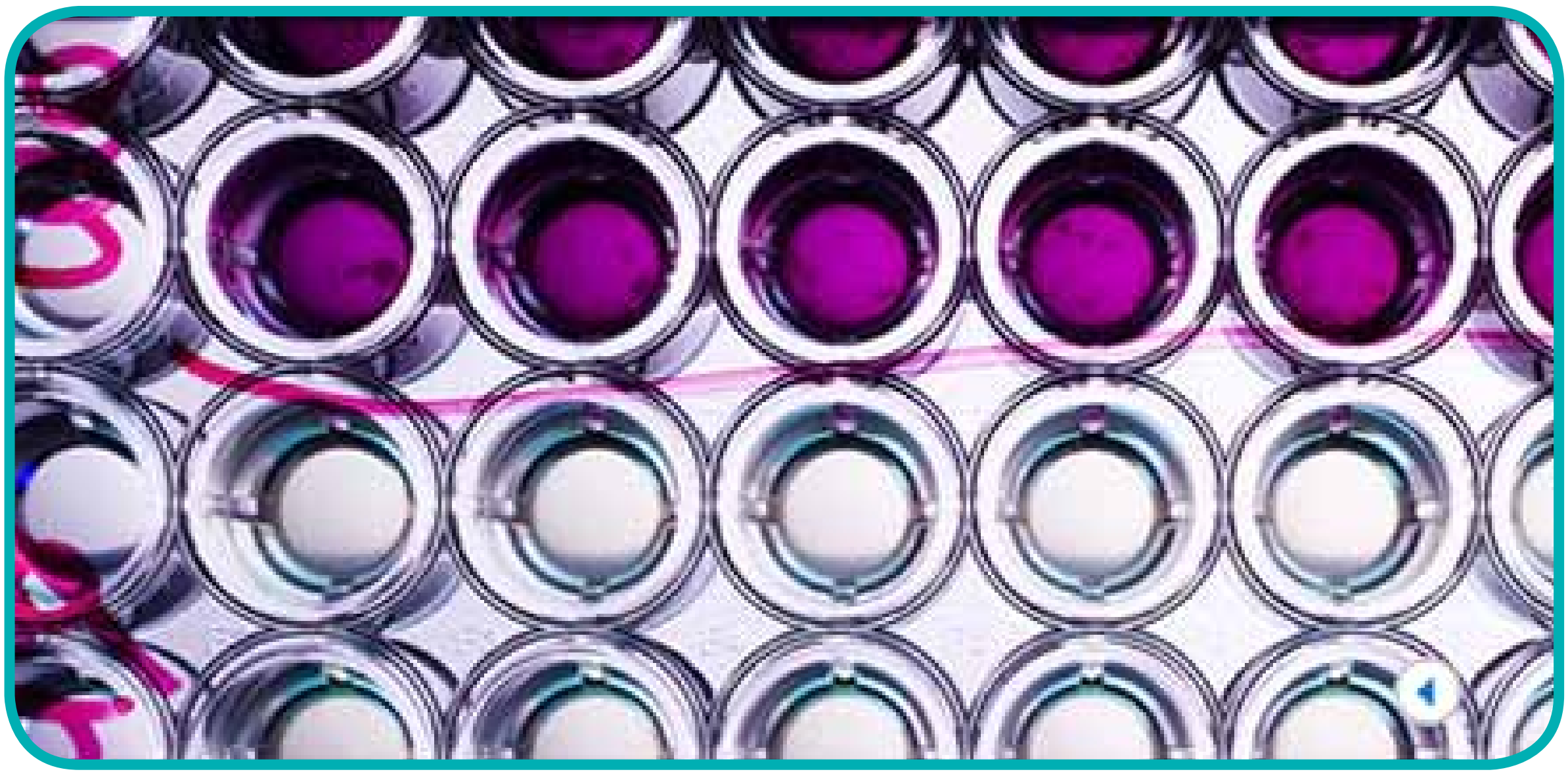

Procedure

A We 'cut' the DNA strands of the viruses from the three continents +

INTRODUCTION

We will cut the DNA of the three variants of HIV using the restriction enzymes, proteins known to researchers for their ability to cut DNA at specific sites. The enzyme we will use specifically cuts the gene responsible for synthesising the candidate protein, but does not cut it if it shows some variation in the DNA region being studied. Therefore, DNA samples are only cut if this gene is conserved.

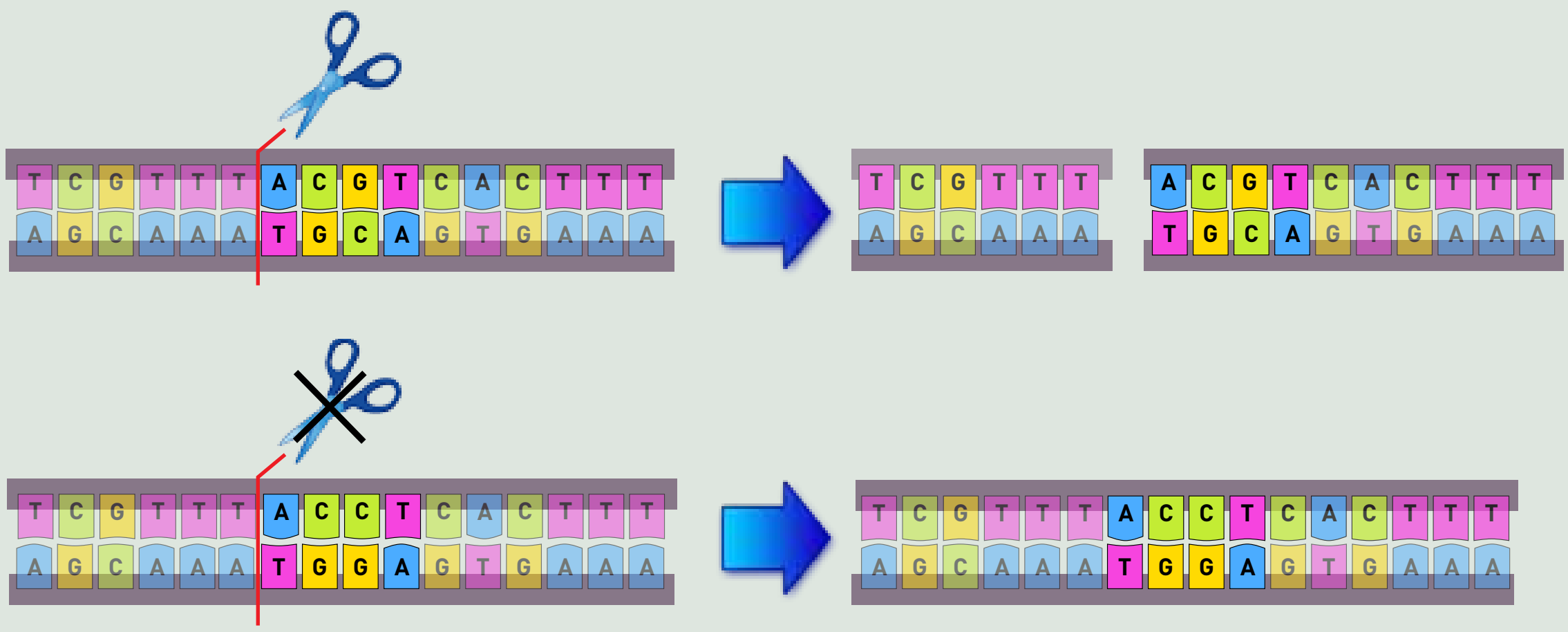

PROCEDURE A

1

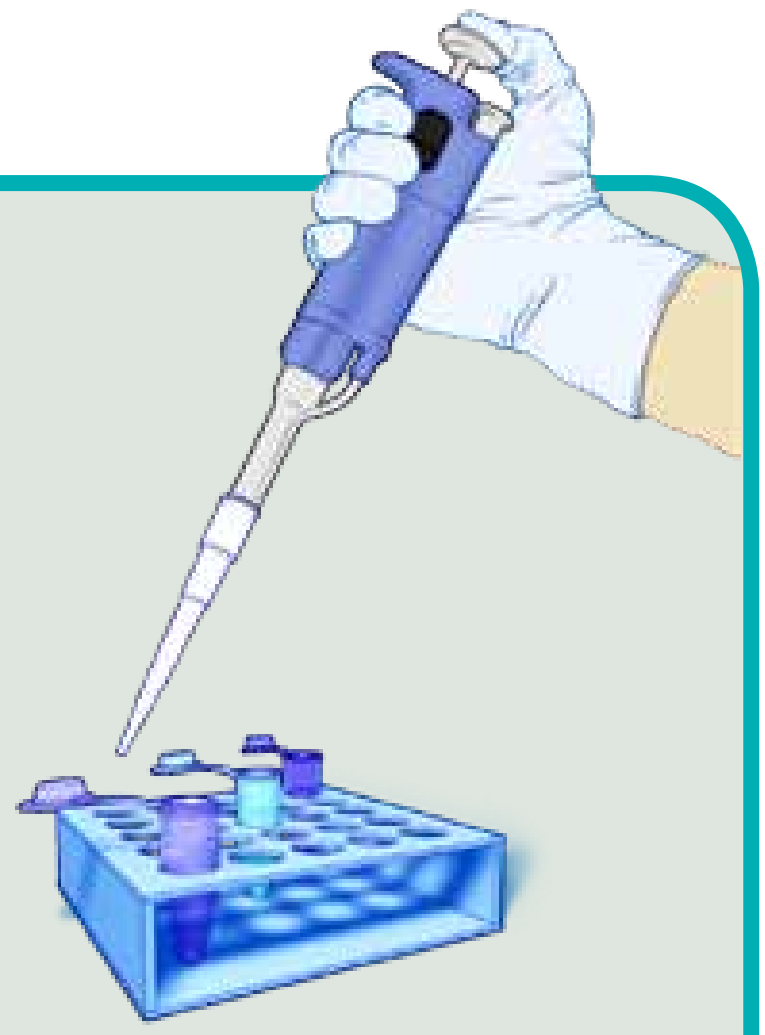

We prepare with the pipette the components of the digestion reaction in four properly labelled 1.5 ml tubes.
We add to each tube:

- Distilled water 16 µl
- Buffer to optimise the performance of the restriction enzyme (green) 3 µl
- Restriction enzyme (cold) 1 µl
- One of the four DNA samples (one of the candidate protein and 3 of each of the HIV variants) 10 µl

2

We cover the tube and tap gently with our finger

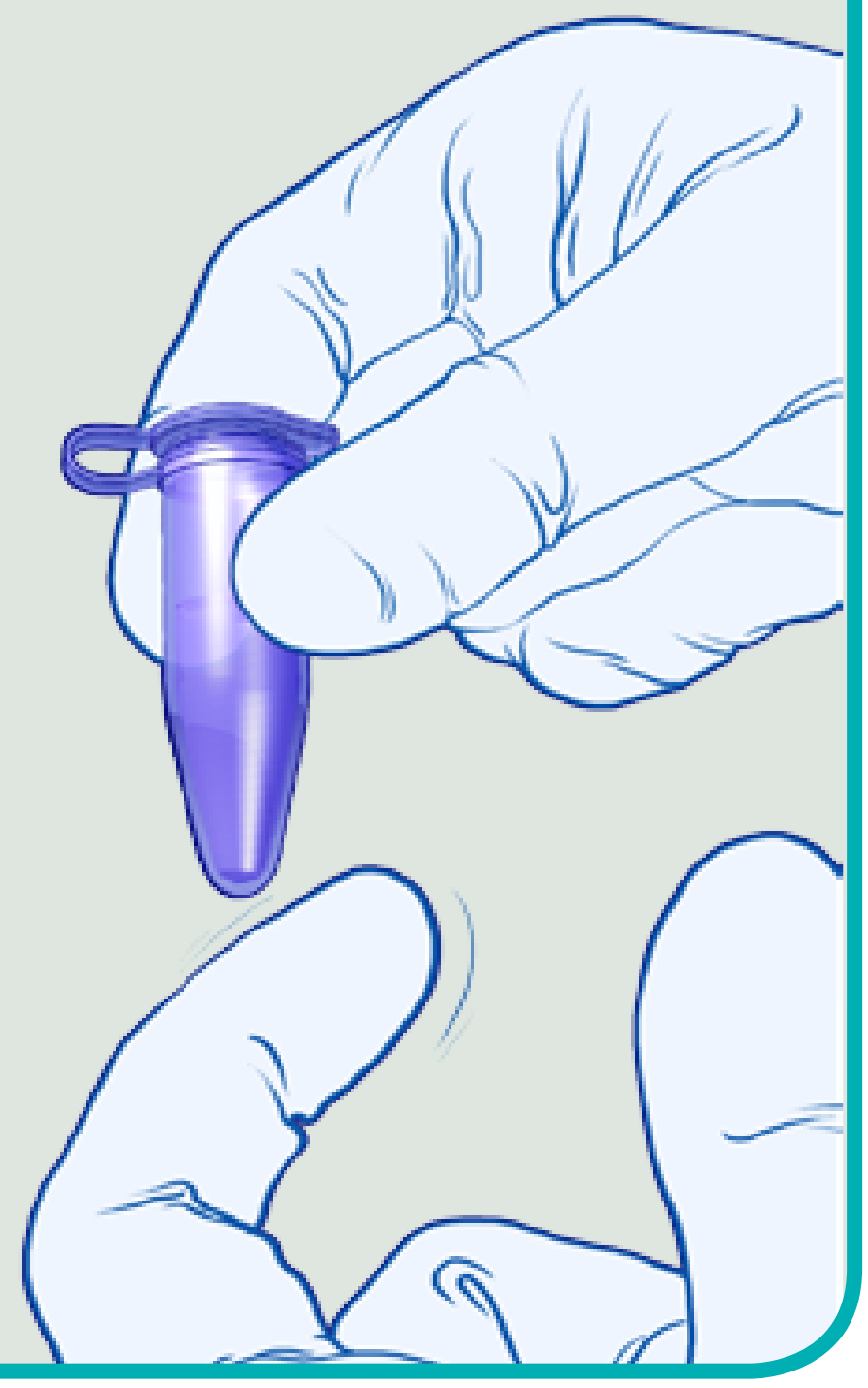

3

We incubate the tube at 37 °C for 5 minutes for the restriction enzyme to cut the DNA

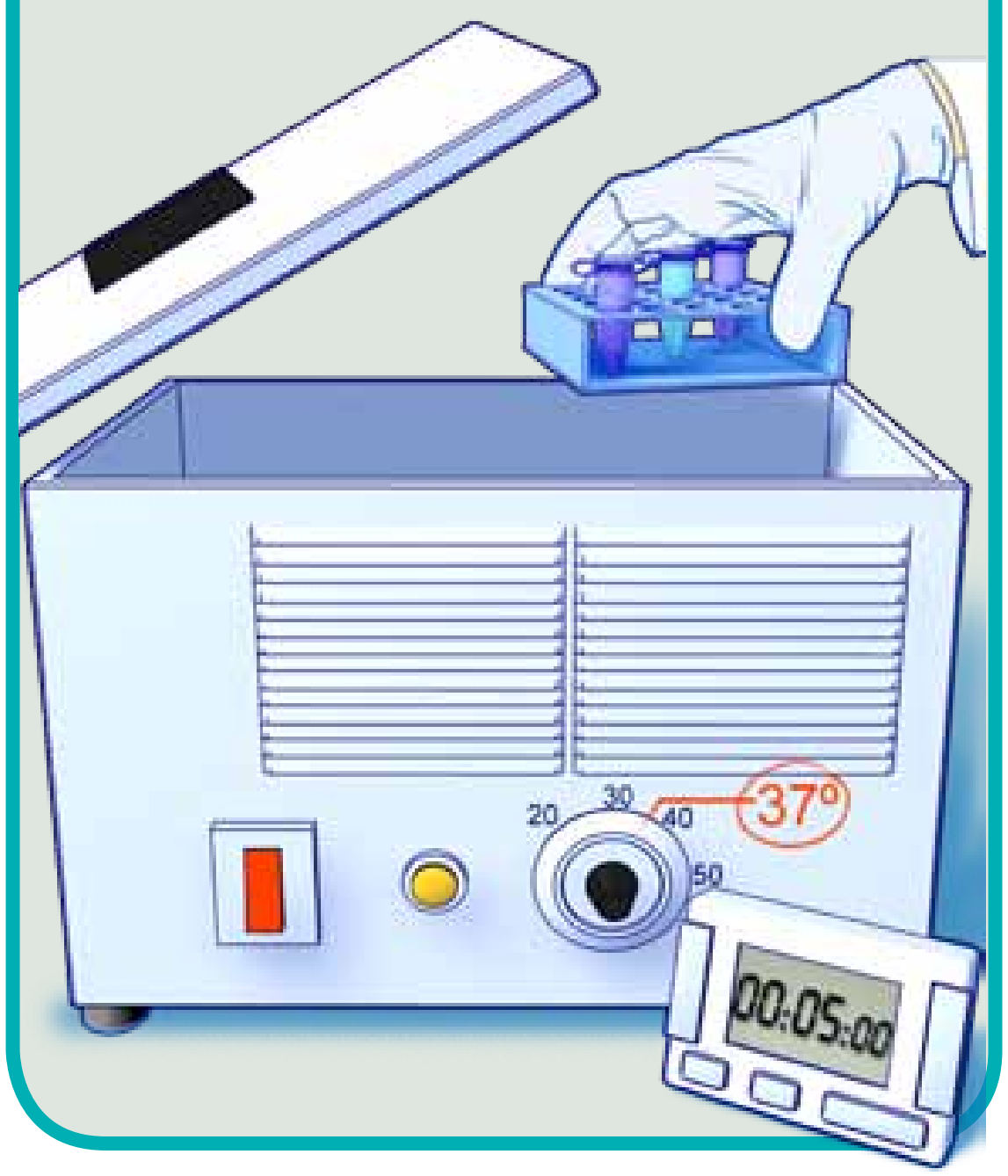

4

After 5 minutes, we place the tube in the cooler to stop the digestion reaction

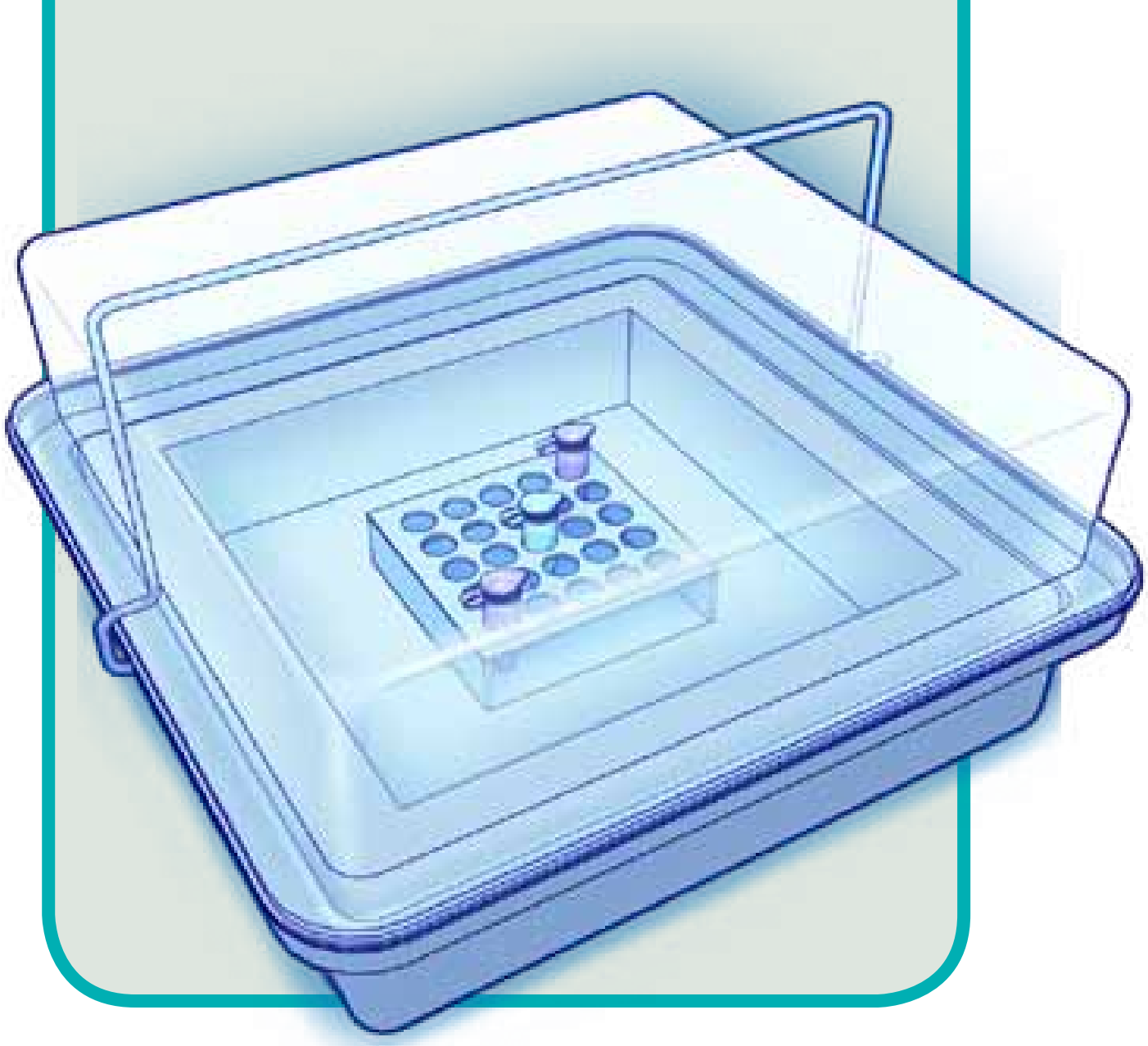

B We separate the cut DNA fragments

INTRODUCTION

To separate the DNA fragments, we will use a technique called electrophoresis, which is widely used in the laboratory and which allows us to separate the DNA fragments according to length.

To do this, DNA samples are placed on a porous gel made with gelatine extracted from seaweed. To facilitate the placement of the samples and see how they move, a 'loading buffer' is added which gives them density and colour. The gel is also loaded with a 'DNA marker', which contains DNA fragments of known measurements in order to work out the length of the fragments of the samples being studied.

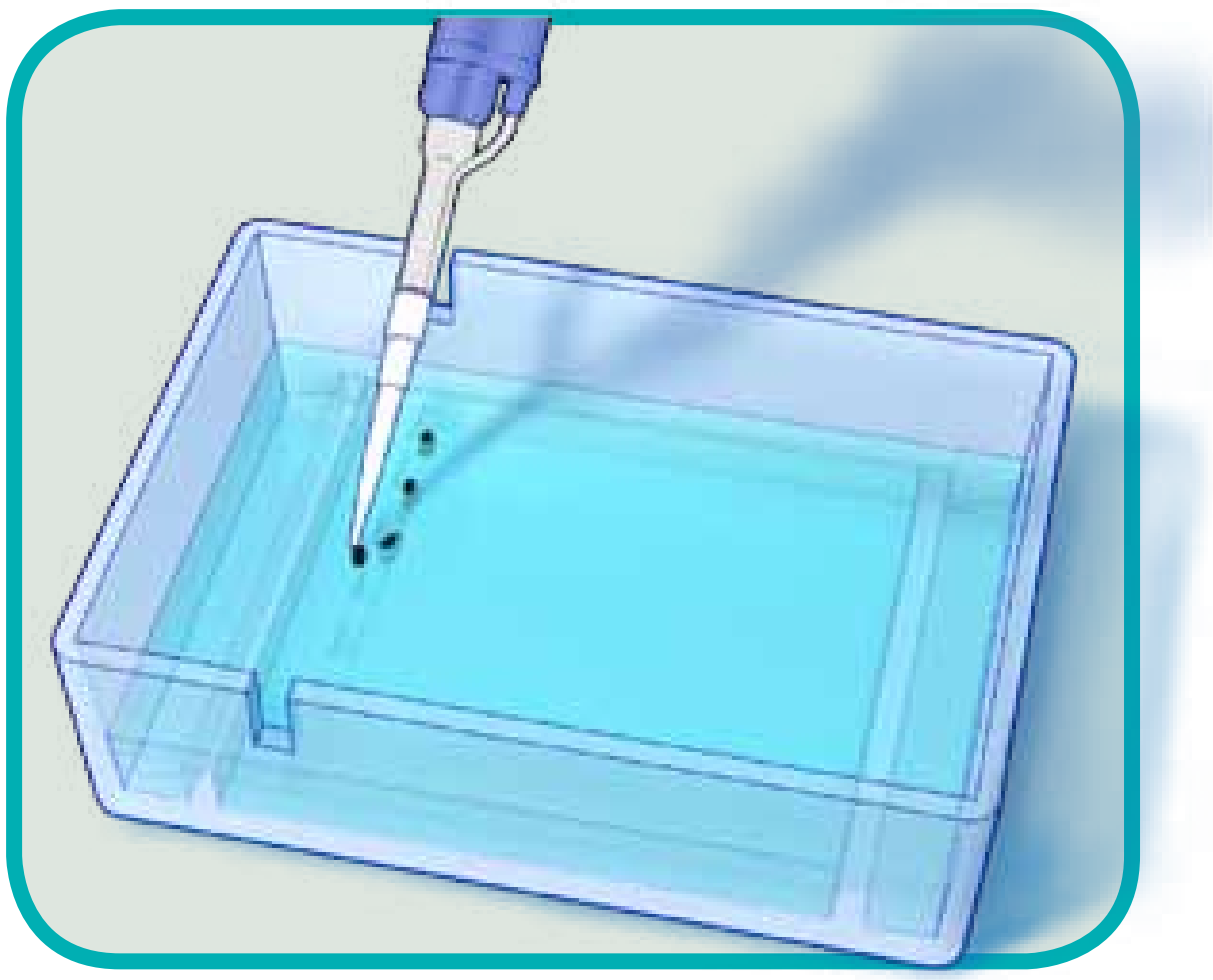

An electric current is applied to the gel, so that the negative charge is concentrated at one end while the positive charge is at the other. As the DNA fragments naturally have a negative charge, when we place them at one end of the gel they are attracted by the positive charge and move. The distance that the fragments travel through the gel is proportional to their length so that the smaller fragments may move more than the larger fragments, which will have more difficulty passing through the pores of the gel.

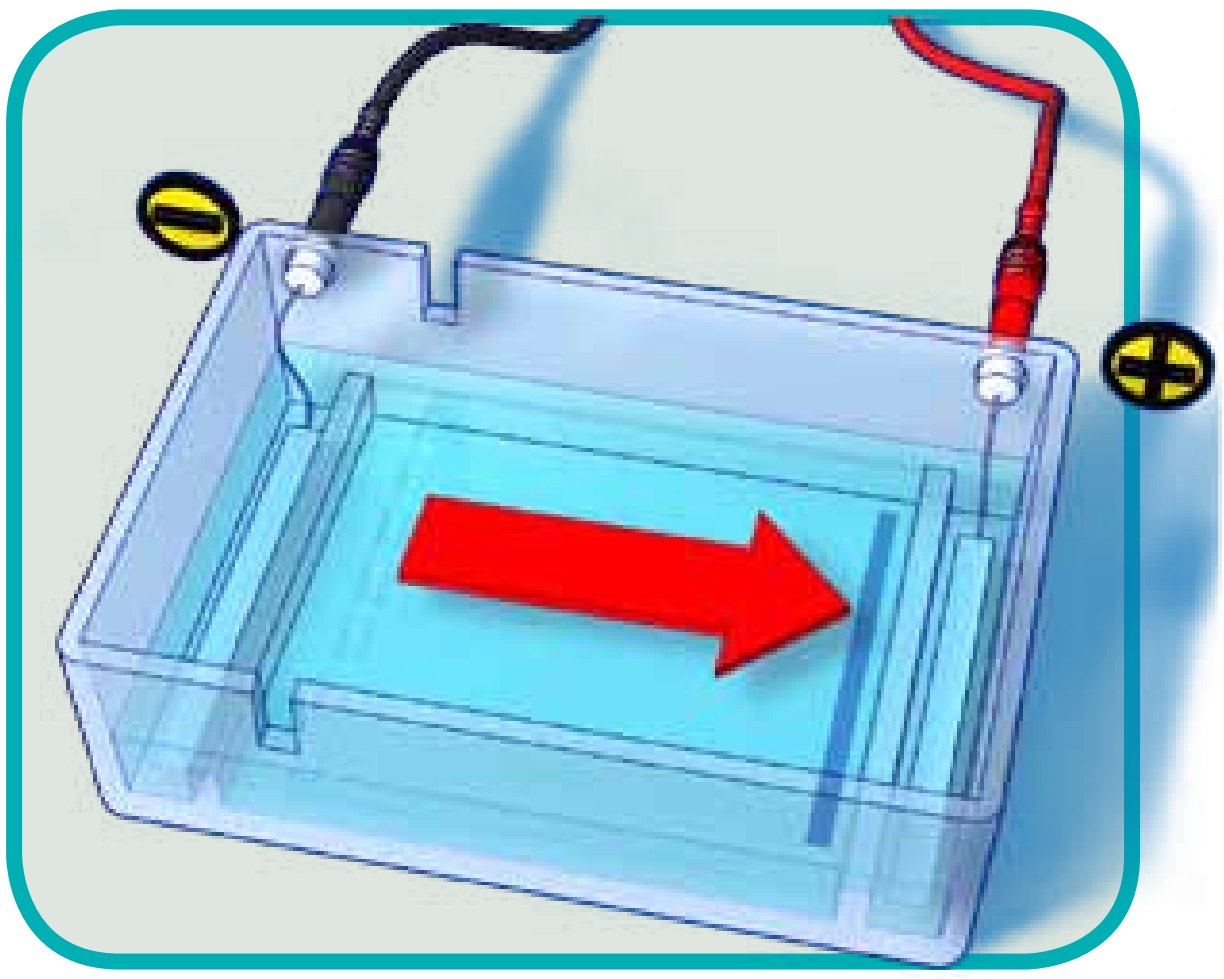

PROCEDURE B

1

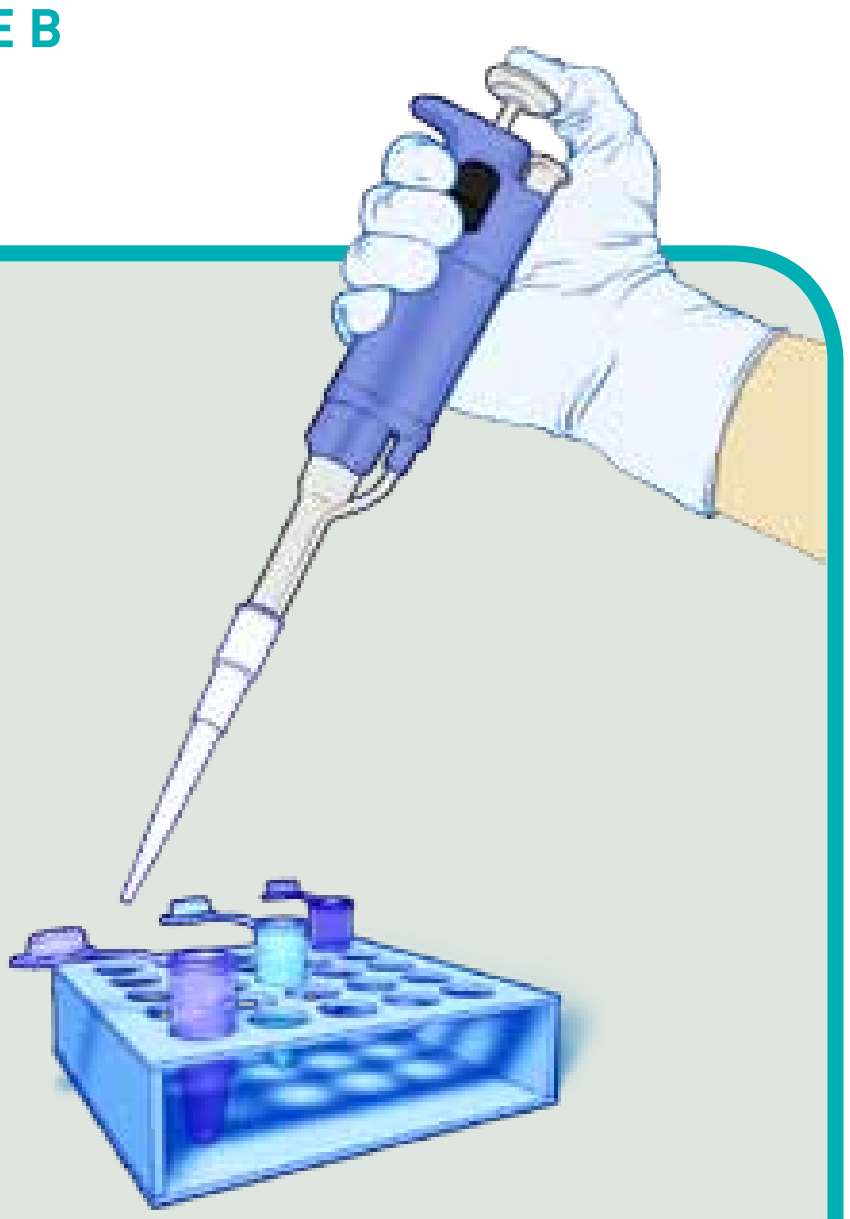

We prepare the DNA samples with a loading buffer: it will not be necessary to add a loading buffer to the samples digested with the restriction enzyme because the enzyme buffer that we added already included a green loading buffer. We therefore take 3 x 0.5 ml tubes and use the pipette to add 2 µl of the green loading buffer and 20 µl of the original undigested sample to them. These samples will serve as a control.

2

We place the gel in the middle of the electrophoresis chamber and with the wells at the negative end. Then we fill the chamber with 0.5× TAE buffer, pouring it down the sides so as not to break the gel, until completely covered.

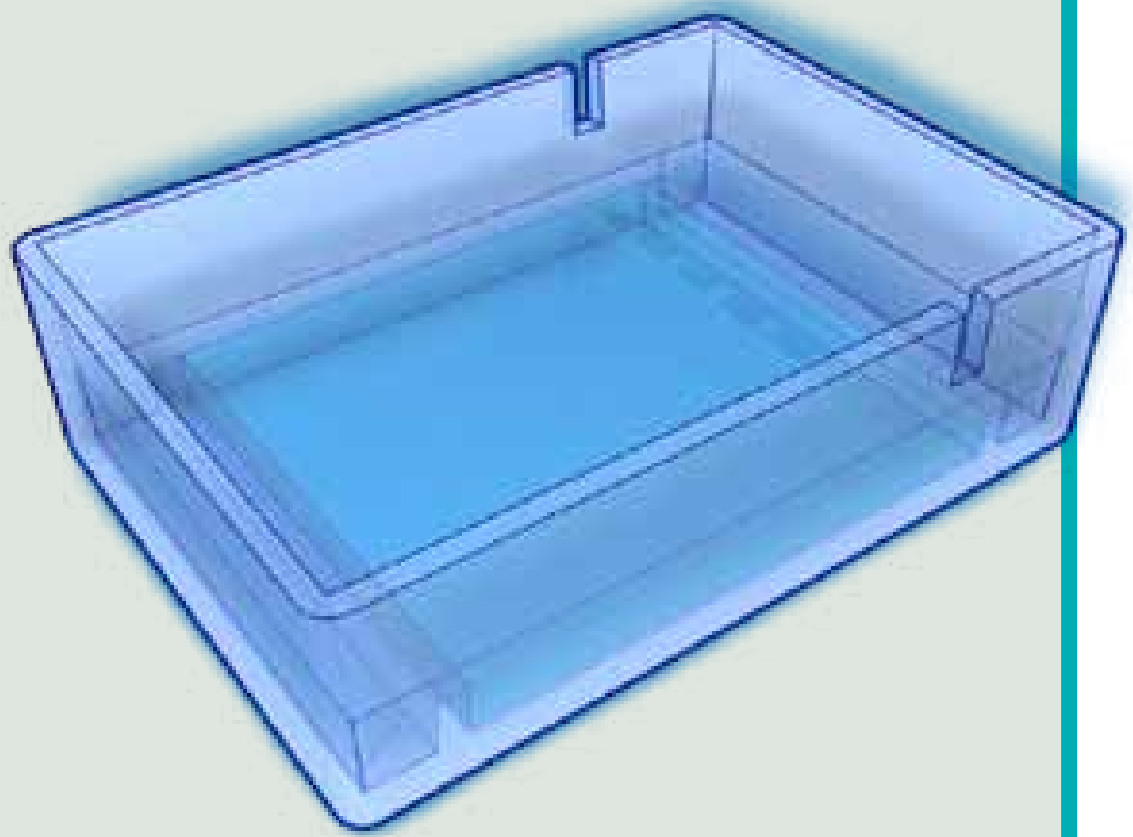

3

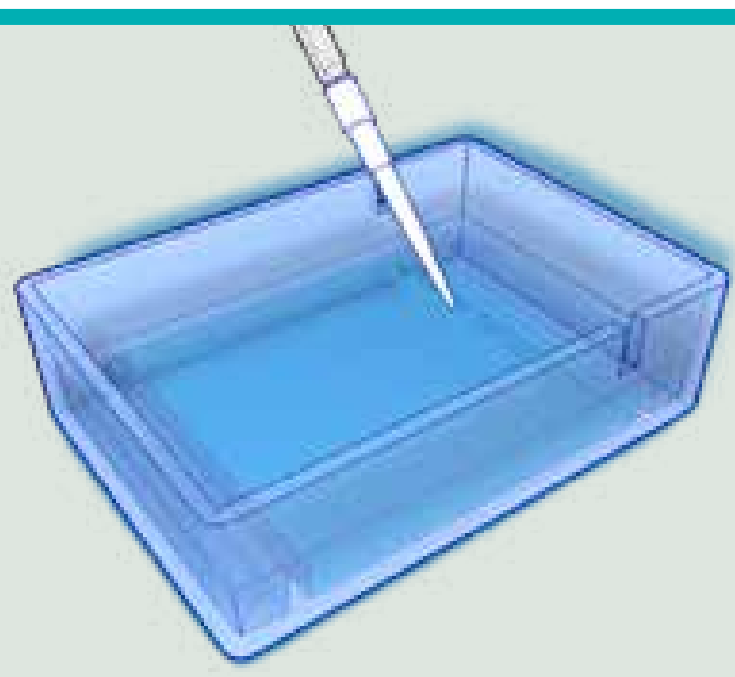

We load the samples into the wells with the pipette, in the following order:

- the first well for (25 µl) of DNA marker
- the following three for DNA samples of the undigested variants (20 µl)
- and the next four for the digested samples (first, the DNA from the candidate, and then the samples of the 3 HIV variants) (25 µl)

4

We cover the chamber and connect it to the power supply at 130 V and 200 mA for approximately 20 minutes.

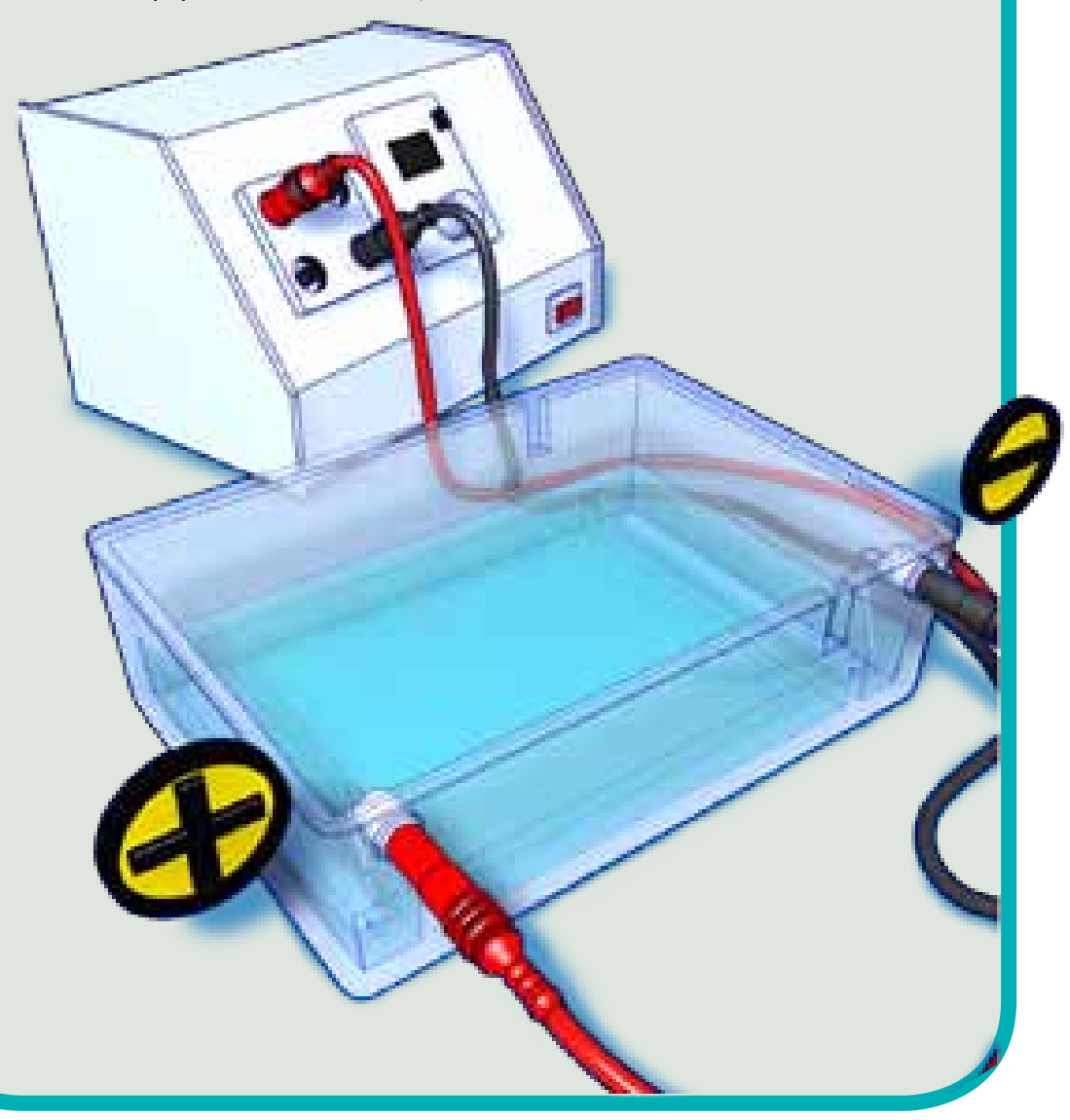

As the gel runs, practise: restriction map

1. Identify the target sequence(s) of the NheI restriction enzyme within the complete DNA sequence of the candidate protein

Complete DNA sequence of the vaccine candidate:

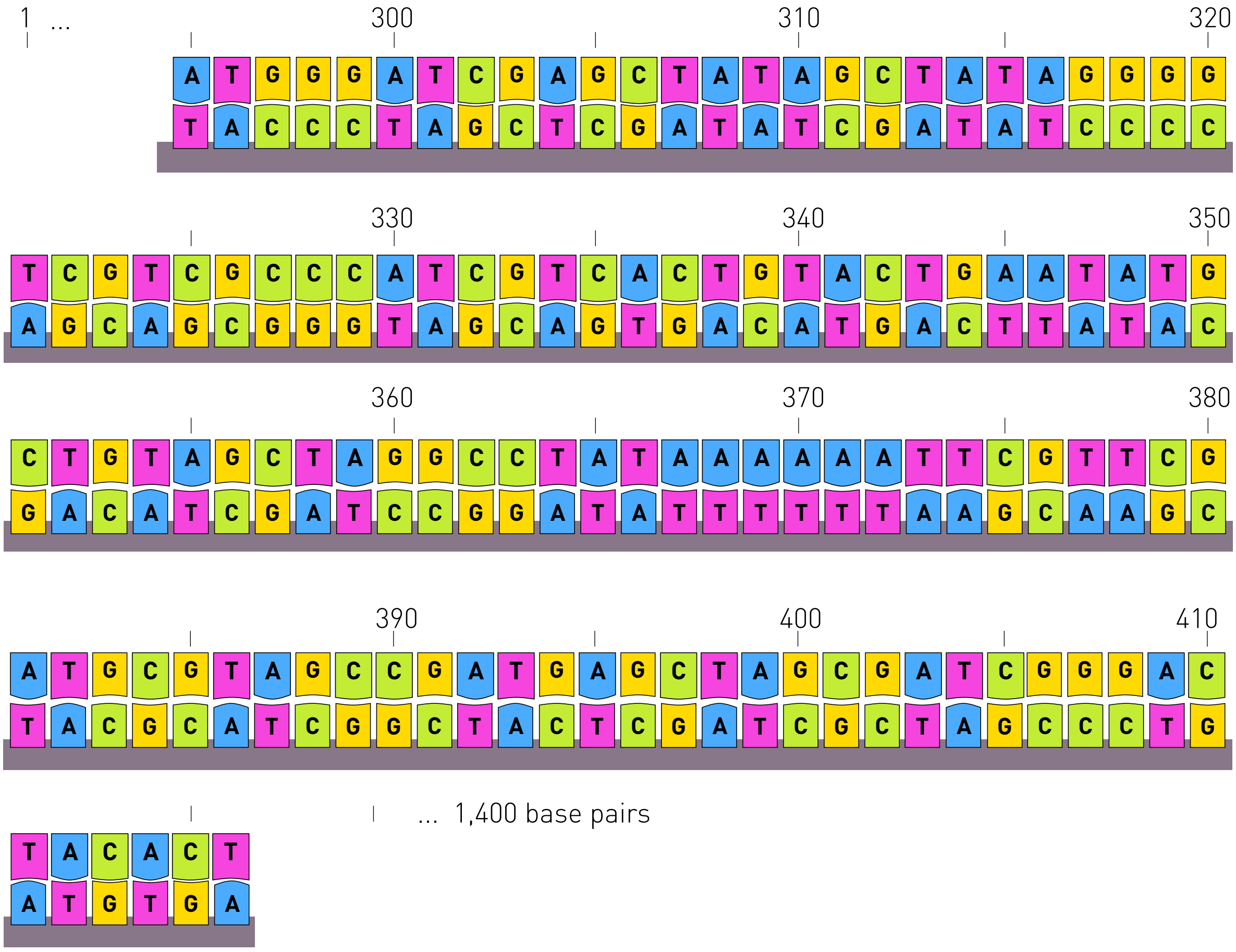

Target sequence of the restriction enzyme:

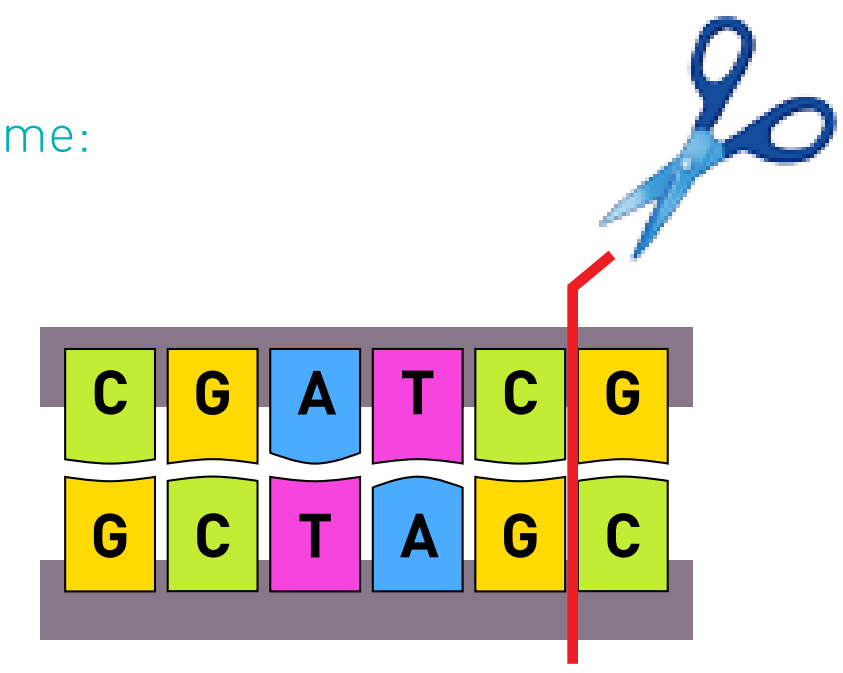

2. How many DNA fragments will we get if the enzyme finds its target and cuts the DNA? Of what size?

3. How many DNA fragments will we get if the enzyme does NOT find its target and does NOT cut the DNA? Of what size?

4. How do you think the DNA marker will help you identify the measurements of the fragments you get?

5. Identify the target sequence(s) of the EcoRV restriction enzyme within the complete DNA sequence of the candidate protein:

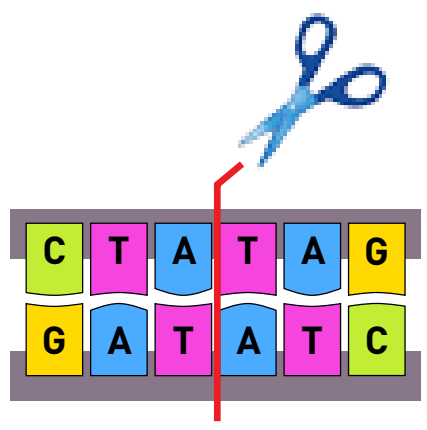

How many DNA fragments would we get if we had used this other enzyme? Of what size?

(In the experiment we used the NheI enzyme because the site where it cuts is the one that we are interested in studying, since it is more effective in stimulating the immune system)

Practise: see how the gel electrophoresis is running

7. Why separate the fragments? How do you separate the fragments from the agarose gel?

8. What charge does the DNA have? What would have happened if you had connected the electrodes the other way round?

9. What do you visualise? Why have you added the loading buffer?

C We visualise the DNA fragments

INTRODUCTION

At this stage we stain the DNA fragments so that we can visualise them and observe whether they have been cut. Only those which have been cut will have the gene responsible for synthesising the IrsiCaixa candidate protein with the original sequence, i.e. no mutation.

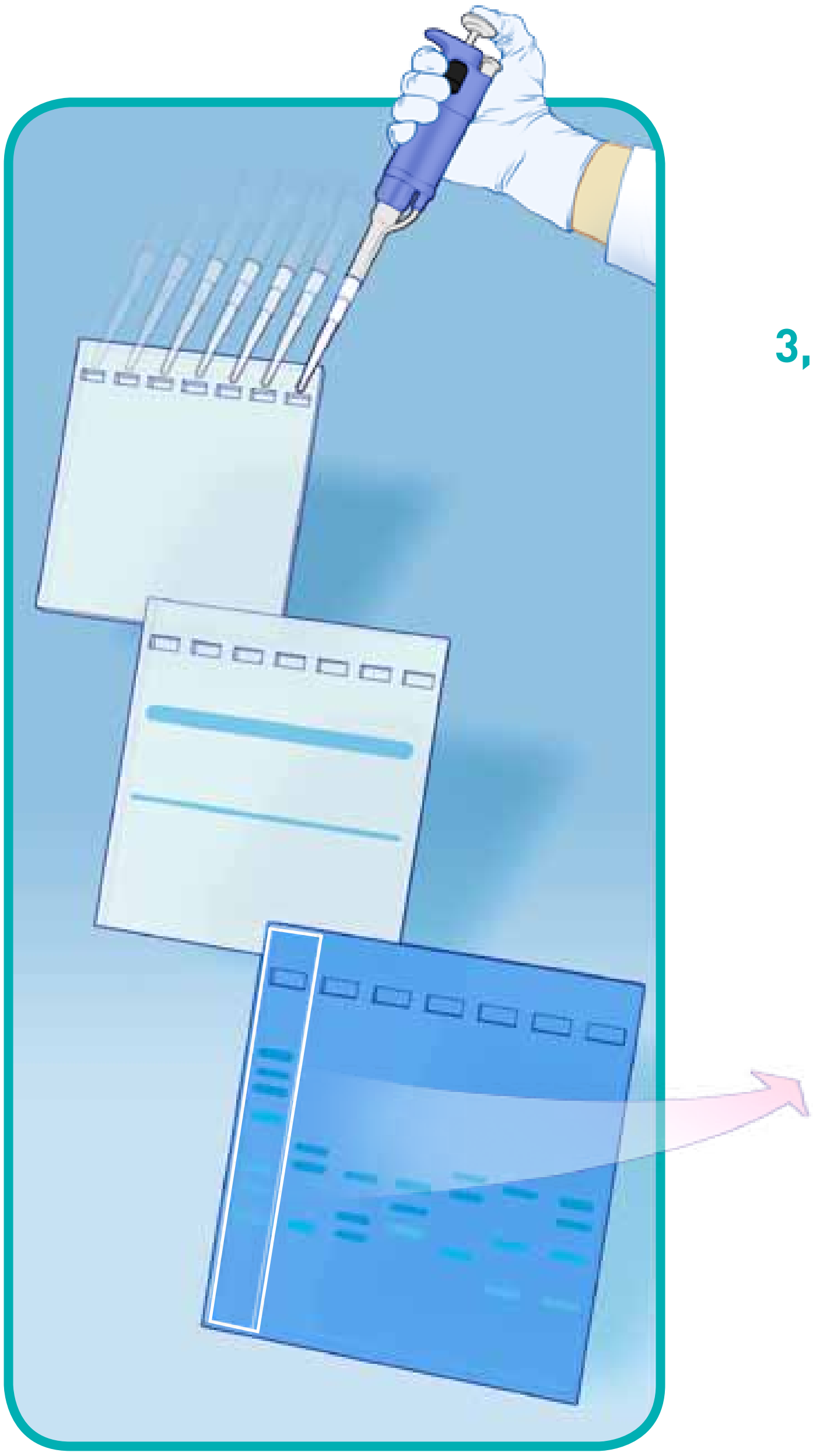

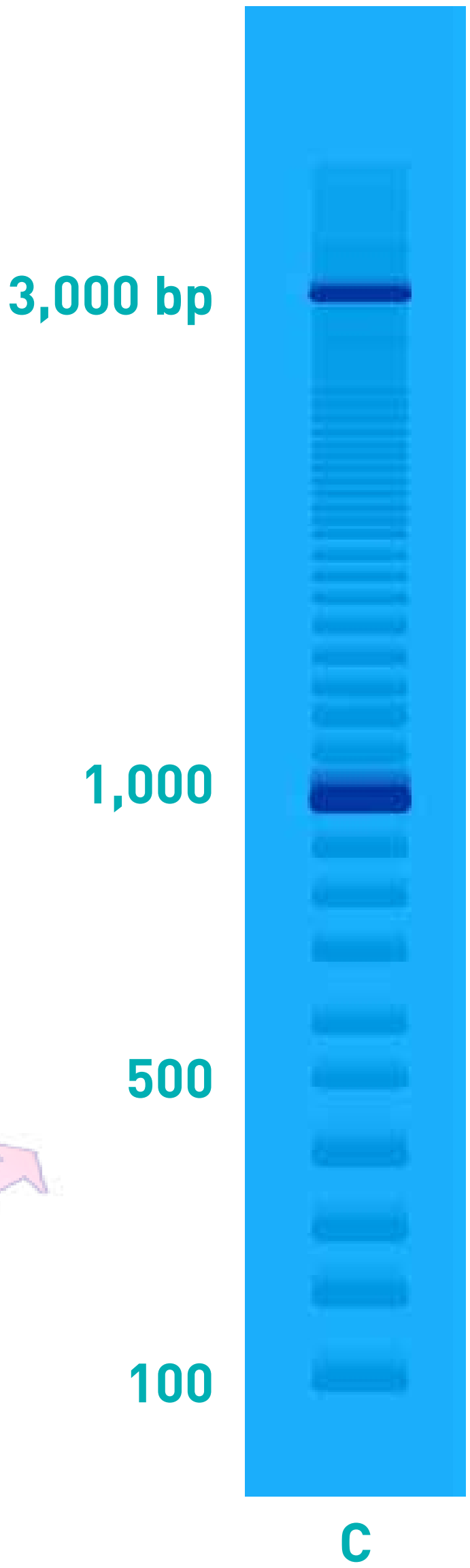

PROCEDURE C

1

We remove the gel from its supports and immerse it in a solution to stain it (Stain Fast Blast) in an appropriate size 'container' for about 2 minutes.

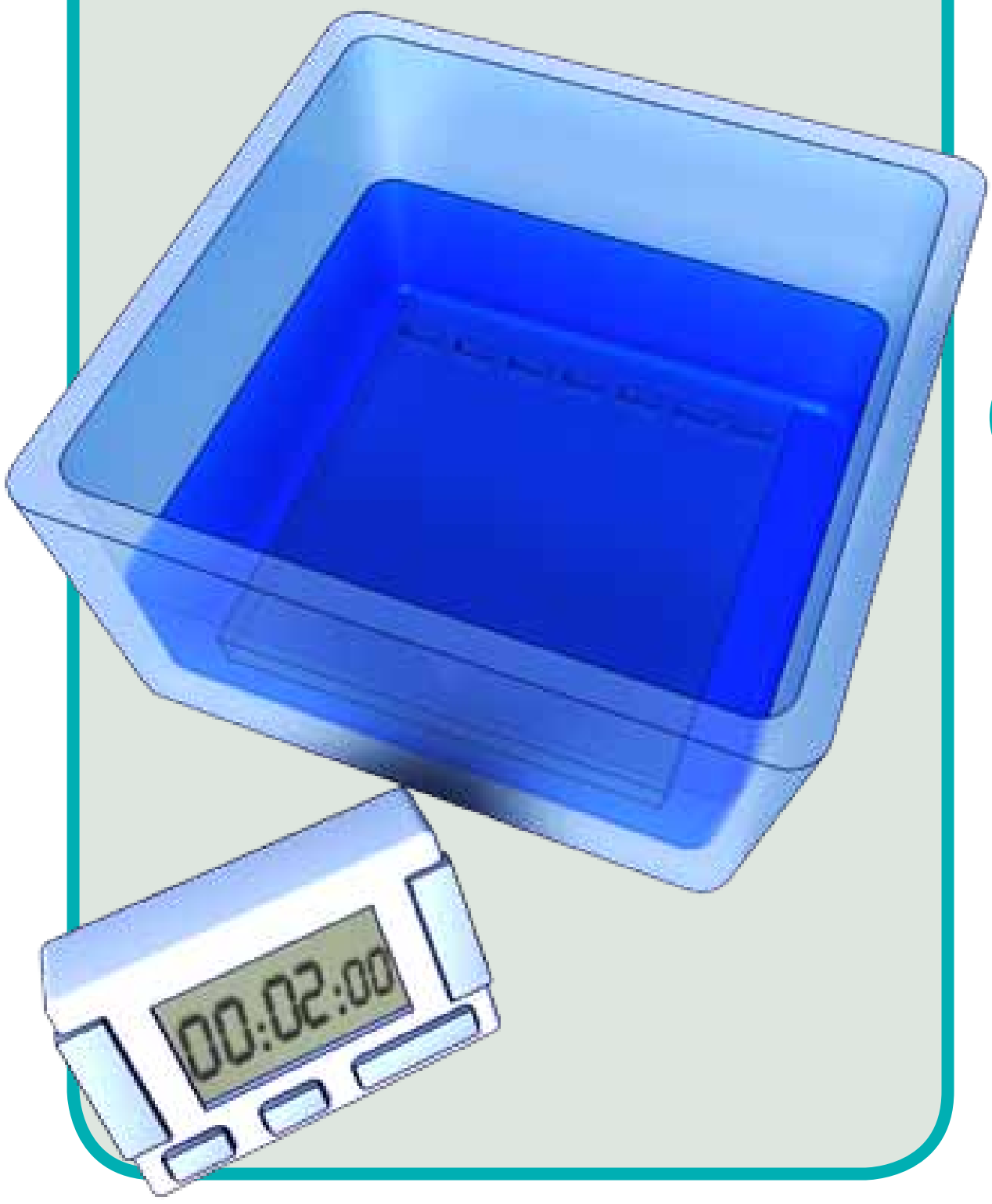

2

We transfer the gel to a bigger 'container' with 500 ml of water. Carefully, we move the gel for around 10 minutes to 'rinse it'.

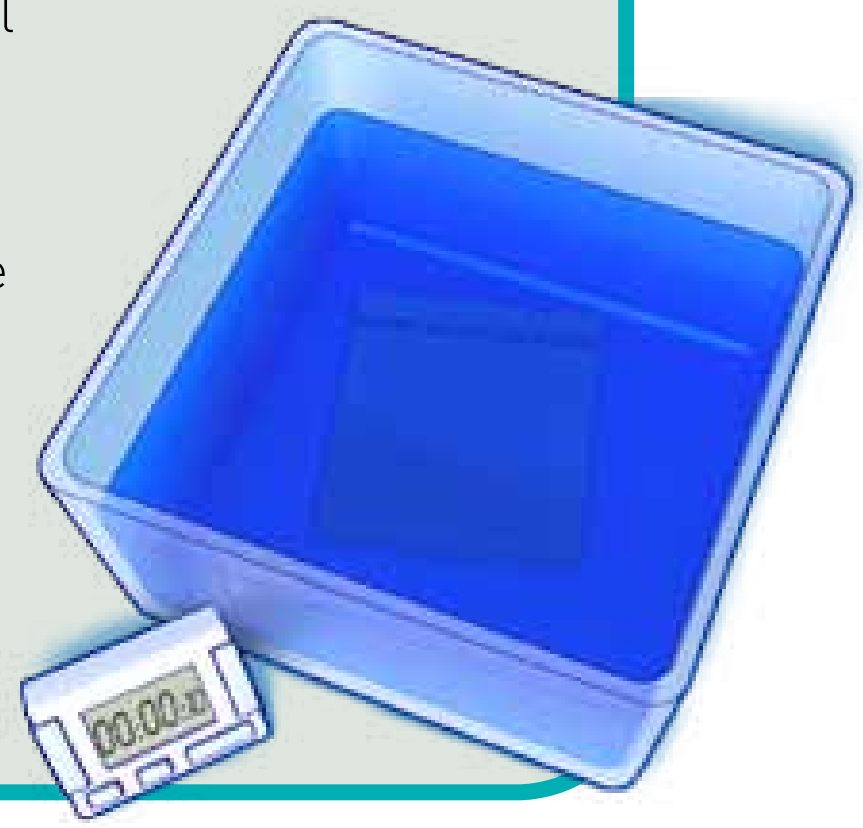

3

Now we need to wash the gel: We transfer the gel to a third 'container' with 500 ml of clean water and gently move the gel once every minute, for a total of 5 minutes.

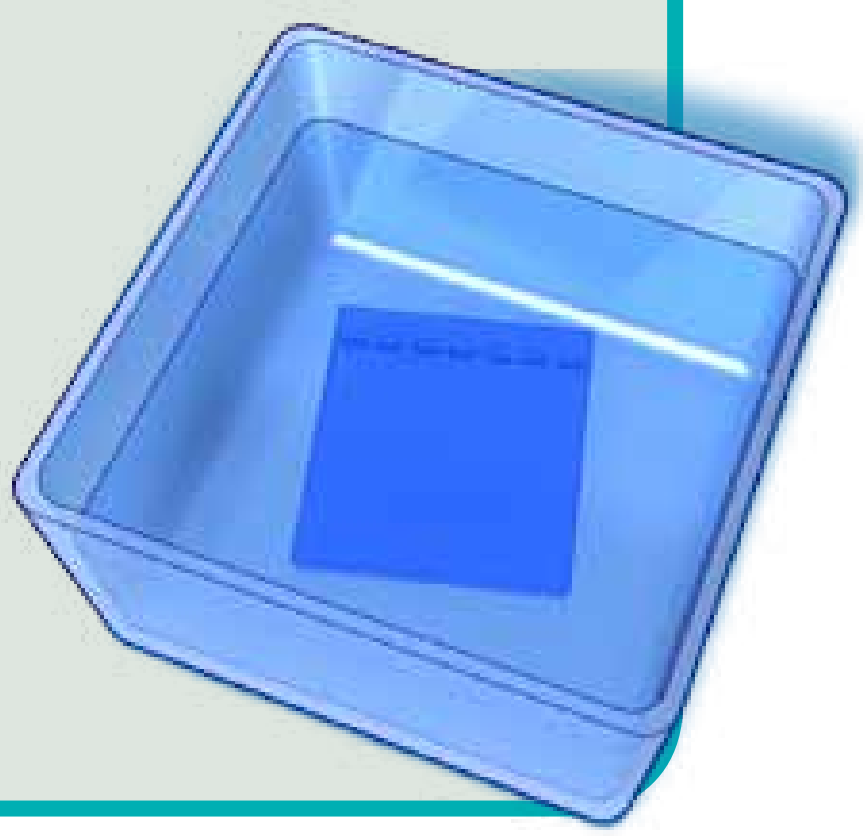

4

We perform a second wash as described in the previous section.

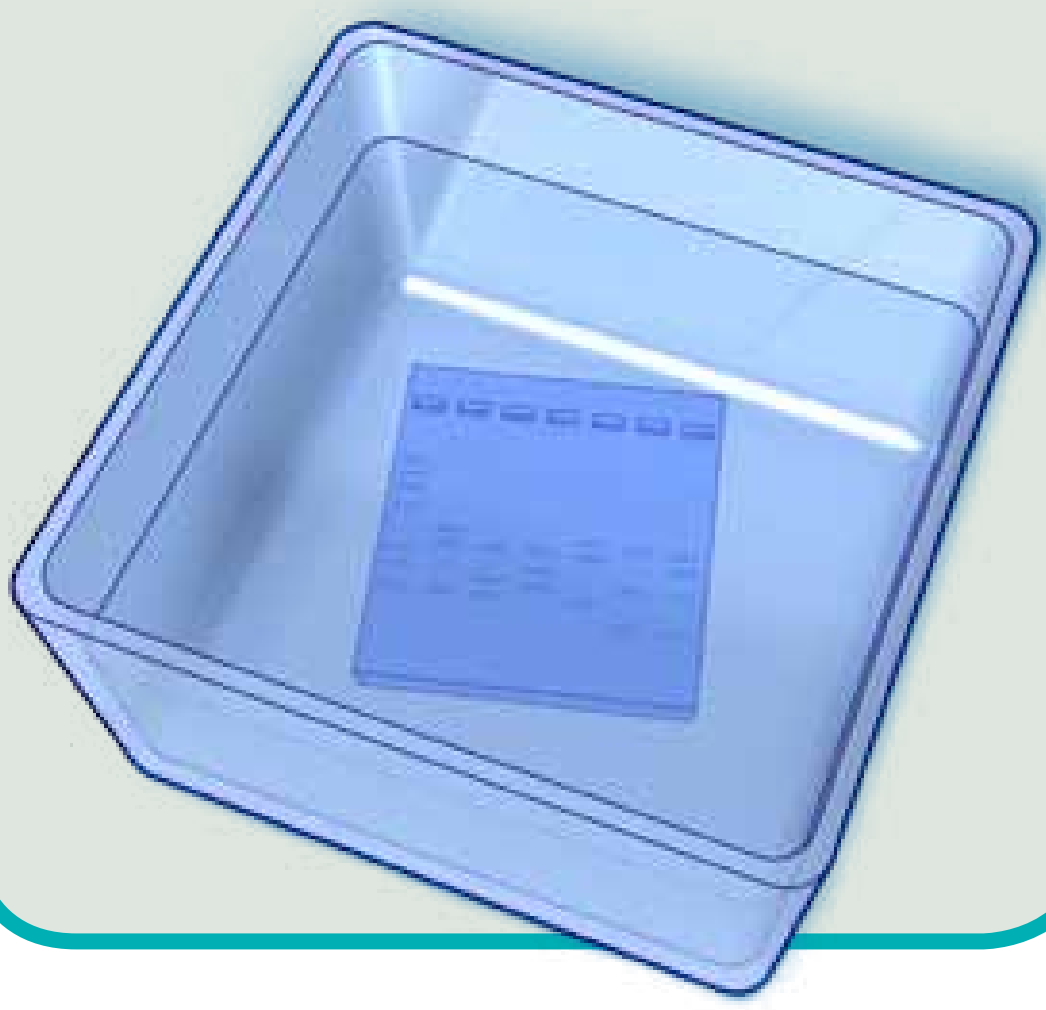

5

The DNA bands should already appear in the gel, although they will be rather blurred. If we wait for 5 to 15 minutes, they will be seen more clearly. *

*For better contrast, more washes with water will be needed.

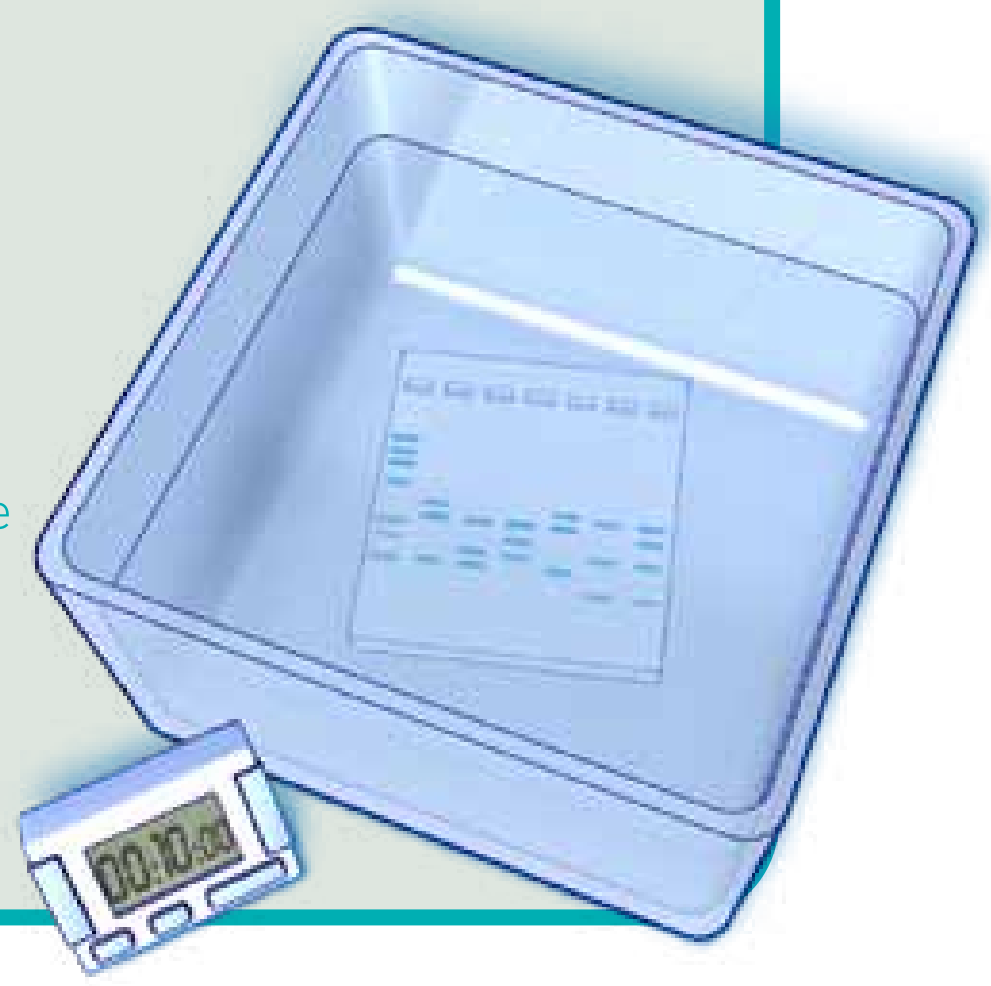

Results and conclusions

10. Look at the DNA bands that you got with each sample of each variant and draw the results below. Also indicate the length of the segments of the DNA marker.

11. What effect have the restriction enzymes had on each of the variants?

African virus	European virus	Asian virus

12. Is there any HIV variant in which the enzyme did not act?

13. So what is the conclusion of your research? (please refer to the initial hypothesis).

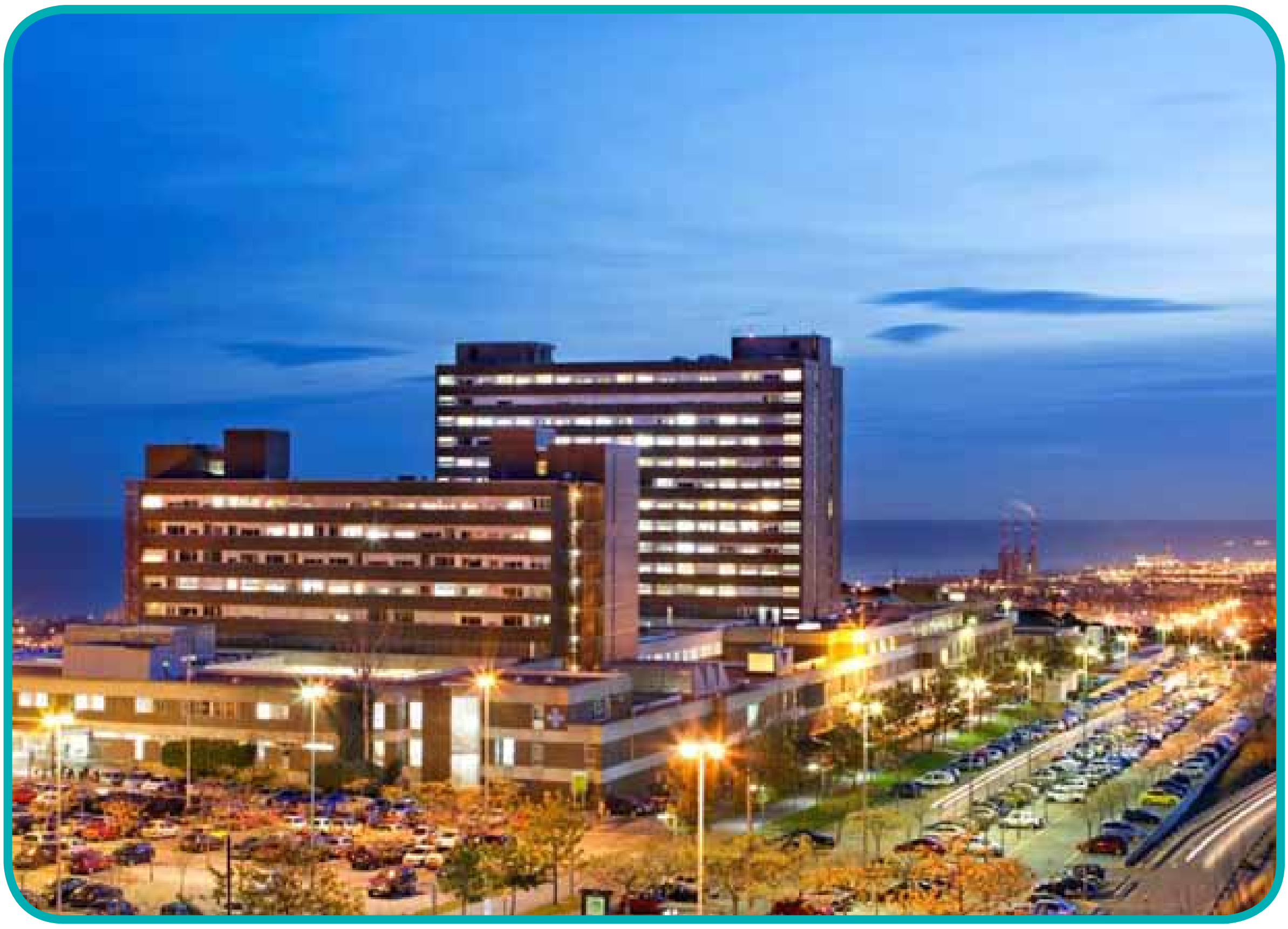

About Irsicaixa

What is Irsicaixa?

The IrsiCaixa Institute for AIDS Research is an international benchmark institute that aims to help improve knowledge, prevention and treatment of HIV infection and AIDS, with the ultimate aim of eradicating the epidemic.

Promoted by "la Caixa" Foundation and the Department of Health of the Government of Catalonia, IrsiCaixa was established in 1995 as a private non-profit organisation and is located at Hospital Universitario Germans Trias i Pujol in Badalona.

Led by Dr Bonaventura Clotet, IrsiCaixa brings together more than fifty researchers who are mainly engaged in basic research to understand the mechanisms of HIV infection and thus develop new therapies and vaccines. At the same time, IrsiCaixa participates in clinical trials to evaluate new therapeutic strategies and cooperates with developing countries in combating the global pandemic.

The scientific research of the IrsiCaixa Institute for AIDS Research is carried out in coordination with the most renowned international research centres, and the resulting publications are among those with the highest impact factors in this field.

Annex

Safety precautions

BE INFORMED
Find out where the safety equipment is located in the laboratory or the place where you are experimenting (fire extinguishers, showers or baths, exits, etc.). Read the instructions carefully before doing an experiment. Do not forget to read the safety labelling for reagents and equipment.

WEAR APPROPRIATE CLOTHING
Gloves, lab coat and goggles.

GENERAL RULES
It is forbidden to smoke, eat or drink in the laboratory or area where you are experimenting. Work in a neat and orderly fashion without hurrying. If any product should spill, clean it up immediately. Always leave materials clean and orderly. Never use equipment or apparatuses without perfectly understanding how they work. Wash your hands before leaving the laboratory.

HANDLING GLASS
Protect your hands when handling materials made of glass and pay attention to their temperature, as hot glass is indistinguishable from cold glass. Do not use cracked glass items.

CHEMICAL PRODUCTS
Do not use unlabelled or not properly labelled containers of reagents. Do not sniff, inhale, taste or touch chemical products. Never pipette by mouth. Wear gloves and wash your hands frequently if you use toxic or corrosive products. In case of contact with eyes, wash them immediately with water. Do not place reagent containers near a flame. Do not heat inflammable liquids. When carrying bottles, hold them from beneath, never by the neck.

WASTE DISPOSAL
Deposit solid and liquid waste that requires it in special, appropriately labelled containers. If you have a question, ask the instructor. Never dispose of solid waste down the sink.

REMEMBER
In case of accident, notify the instructor immediately.

SPECIFIC PRECAUTIONS FOR THIS WORKSHOP

In this workshop you will be handling chemical reagents, solvents and instruments that present the following degrees of hazard:

TAE buffer: Toxic if swallowed and can cause severe eye irritation. It needs to be disposed of as hazardous waste.

Agarose gel: It needs to be disposed of as hazardous waste. Because it contains TAE buffer, follow the instructions specified above.

DNA Stain Fast Blast: Toxic if swallowed. There is no need to dispose of it as hazardous waste, but it must be diluted with plenty of water.

Electrophoresis: We must pay special attention to electrical hazards.

Xplore Health

DISCOVER THE LATEST ON HEALTH RESEARCH

Keep on researching at Xplore Health!

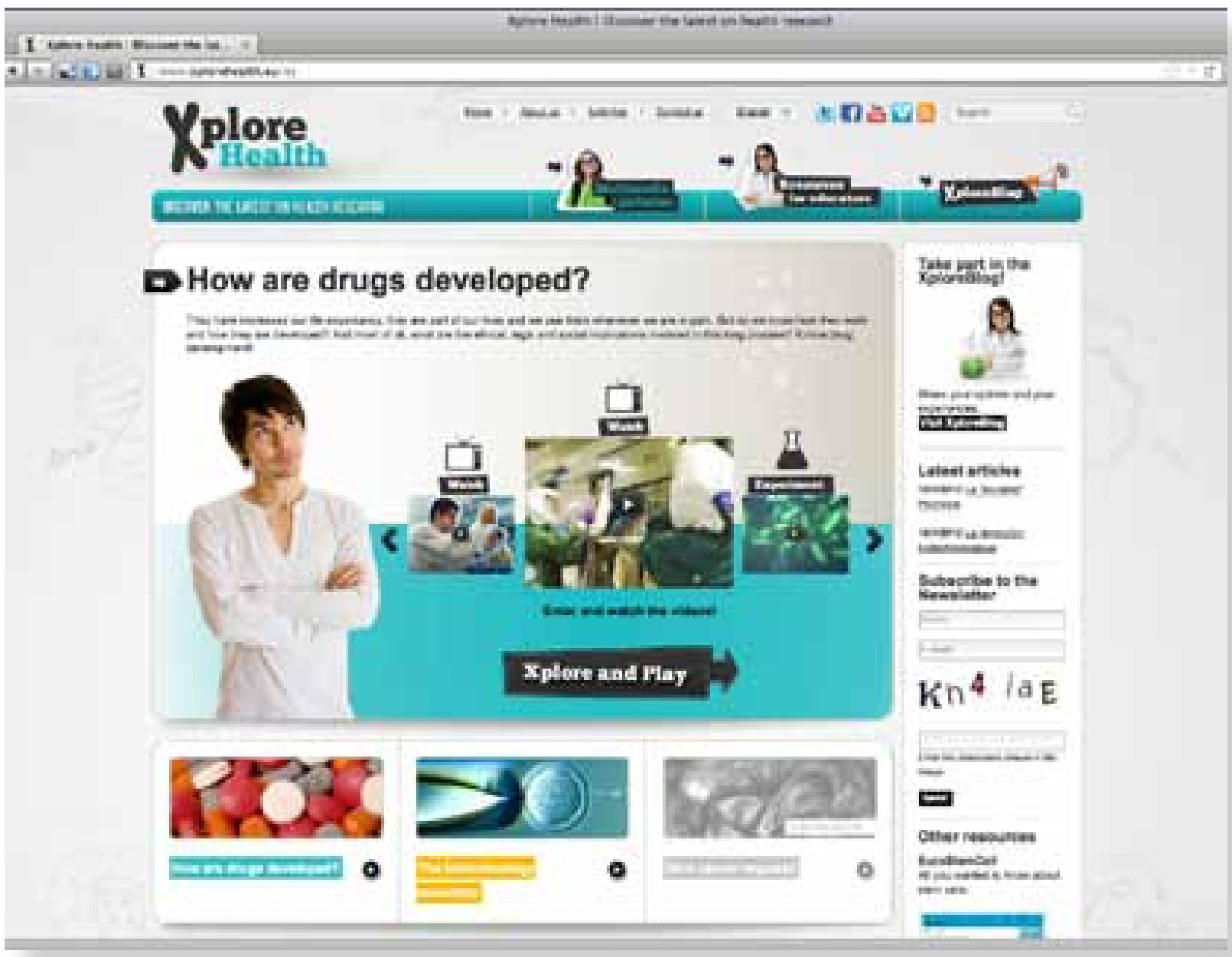

www.xplorehealth.eu

Investigators who have contributed content: Marta Curriu, investigator for IrsiCaixa.

DEVELOPED BY

IrsiCaixa Institut de Recerca de la Sida · Parc Científic de Barcelona · Obra Social "la Caixa"

This work has been included with an Attribution-NonCommercial-NoDerivs 3.0 Unported Creative Commons licence. To see a copy of this licence, visit http://creativecommons.org/licenses/by-nc-nd/3.0/

HIV and Aids

Key Terms

- **HIV**: Human Immunodeficiency Virus, a virus that causes an incurable infection transmitted by sexual activity, contaminated needles, infected blood or from mother to child, which attacks the body's immune system.
- **AIDS:** Acquired Immunodeficiency Syndrome, an advanced stage of HIV infection where the immune system is greatly weakened and other infections appear because of this.
- **Immune system:** The body's natural defense system against disease.

What's all the fuss about?

HIV/Aids cannot be cured, though with the right treatment people can live for many years after infection, with few symptoms and side effects of the treatment. The main way of transmitting HIV is through sexual relations.

The question of how to prevent HIV is often controversial. In much of the world, treatment is hard to come by and expensive. Even where effective prevention and treatment are available, people may become exposed to HIV because they don't understand the risk, or they are socially vulnerable. Finally, stigma and discrimination of people living with HIV is still widespread, as often they are marked out as being different and are blamed for that difference.

Statistics[1]

- **34.2 million:** The number of people in the world living with HIV in 2011.
- **7000:** the number of new infections diagnosed every day around the world.
- **30 million:** The number of people who have died from AIDS since its discovery in 1981.

---

[1] Sources: WHO http://www.who.int/mediacentre/factsheets/fs360/en/index.html and www.unaids.org/documents/20101123_FS_nawe_em_en.pdf

Science Q&A

What is HIV?

HIV, or Human Immunodeficiency Virus, is a virus that attacks the body's immune system.

It replicates by getting into cells in the patient's body and inserting chunks of its own genetic code into the DNA in those cells, in order to instruct them to make new viruses. The new viruses can then infect other cells.

HIV infection through sex

90% of HIV infections in the UK are from sex, of which around half are between a man and a woman, and around half are between two men.

In the case of HIV, the main type of cells which get infected are cells which help the body fight against disease. This leads to the patient having a weakened immune system. If HIV is left untreated, AIDS will usually develop. This is an advanced state of the HIV infection where the patient can die of other infections or cancers because the immune system is not strong enough to fight them off.

How do people get infected with HIV?

There are three main ways people get infected with HIV:

HIV infection through drug use

In the UK, 5% of HIV infections are from drug users sharing needles to inject drugs. In Eastern Europe, this figure rises to 44%.

Sexual transmission: In Europe, HIV is most often spread through unprotected sex. Bodily fluids like semen, blood and vaginal fluid can transmit the HIV virus into the bloodstream of a sexual partner.

Transmission through the blood: If the HIV virus is present in the blood it can be transmitted when infected blood enters into the uninfected bloodstream. This can be from drug users sharing needles, a blood transfusion using infected blood, a tattoo or piercing where the needle is contaminated or when infected blood comes into contact with an open wound.

Mother to child: A woman with HIV who gets pregnant can transmit the virus to her child during pregnancy or during the delivery itself.

How do people live with HIV?

> ***HIV transmission between men***
>
> *Data from 23 European countries show that the annual number of HIV diagnoses among men who have sex with men rose by 86% between 2000 and 2006.*

There is no cure. At the moment, the only treatment is a combination of anti-HIV drugs. These drugs slow down the development of the disease by targeting the virus while it replicates, limiting various stages of its life cycle.

HIV multiplies very quickly, and can also become resistant to drugs. This is why we use a combination of drugs, to make it harder for the virus to replicate. Then new, resistant variants of the virus are less likely to appear. If patients stick carefully to their treatment, they can keep the virus levels in their blood so low that they may be undetectable for years.

People living with HIV take a number of tablets every day. They are encouraged to exercise, eat healthily, stop smoking and have regular vaccinations to minimise the risk of getting serious illnesses.

How can HIV transmission be prevented?

In Europe, public awareness campaigns about HIV usually focus on preventing sexual transmission by using condoms where the HIV status of the partner is unknown, and getting people tested for HIV so they know their status. Screening blood for HIV before blood transfusions and using disposable syringes are two ways to prevent transmission into the bloodstream.

Many HIV prevention methods use anti-HIV treatment to decrease the chance of HIV transmission:

- **Lowering the viral load:** People living with HIV often take treatment for their own health. Anti-HIV treatment decreases the amount of the virus in a person's bodily fluids, known as the 'viral load', thereby making them less likely to get AIDS-related illnesses. An HIV-positive person's viral load is the single biggest risk factor in the transmission of HIV. So taking treatment has the additional benefit of significantly decreasing this risk. The idea of 'treatment as prevention' is to use treatment as a prevention strategy that people could use to protect their sexual partners, or, on a large scale, to reduce HIV transmission among a population.
- **Prevention of mother-to-child transmission**: Across the world, HIV-positive pregnant women take anti-HIV drugs to reduce the chances of transmitting the virus to their babies. Without intervention there is a 20-45 percent chance that a baby born to an HIV-infected mother will become infected. Treatment for the mother during pregnancy significantly reduces this risk.

- **Post exposure prophylaxis**: After a risk of exposure to HIV, through sex or through contact with HIV-infected blood, patients can receive an emergency anti-HIV treatment called PEP. This is usually a 4-week course of anti-HIV drugs. It must begin within 72 hours of the risk of infection. PEP reduces the chances of infection by around 80%, although it is expensive as a treatment and can have side effects.

What research is being conducted into prevention and treatment?

Prevention:

- New technologies such as **microbicides** (gels, creams or sprays capable of killing the virus) are continually being tested to see how well they prevent HIV being passed on through sex.
- Researchers are also working on a **vaccine** which could prevent people from becoming infected.
- Another possible prevention technique being researched is **pre-exposure prophylaxis**. This involves providing people who are not infected with HIV with anti-HIV drugs before possible exposure to the virus, to stop them from becoming infected. It has the potential to be useful for couples where only one partner is living with HIV. But it could also be used as a prevention method for people at high risk.

Treatment: Researchers are looking for ways to either treat HIV infection so that it disappears all together, or to minimize the infection. The most promising strategies being investigated are:

- **Gene therapy** – modifying patients' immune systems to make them resistant to HIV infection.
- **Treatment optimization and intensification** – stopping the virus replicating any further.
- **Immune-based therapies and reversal of HIV latency** – stimulating the body's immune system and making dormant HIV-infected cells detectable to the immune system.
- **Therapeutic vaccination** – improving the patient's immune response and allowing control over HIV at low levels.

Discussion Continuum

This activity is designed to facilitate dialogue about the ethical, legal and social aspects of research into HIV and Aids. Groups of 4-12 students discuss the issues raised by each statement and choose where each card should go between 'agree' and 'disagree'.

Contents:

- An AGREE and a DISAGREE card
- 8 Discussion Cards, which include a statement on some aspect of HIV and Aids

Gameplay:

1. Players form small groups, between 4 and 12 per group. Each group is given an AGREE and DISAGREE card and 8 discussion cards.
2. Within each group, the AGREE card and DISAGREE card are placed on the floor/table about one metre apart, to represent the two extremes of the continuum. The space in between is where the discussion cards will be placed.
3. The first player reads the first discussion card to the rest of the group. The player should check everyone understands the card, and use information from the introduction where necessary to ensure the group understands the statement.
4. The first player then decides to what extent they agree with the first card. They place the card face up, anywhere on the discussion continuum, closer to AGREE or DISAGREE as they choose. This is entirely the choice of the individual player, and is not discussed by the group. The player can give a reason, if they wish.
5. Each player in turn then reads a card, checks that everyone understands, and chooses individually where to place it on the continuum in a similar way.
6. When all the cards have been read, understood and placed on the continuum, the discussion begins. The aim is to place the cards between AGREE and DISAGREE in an order that most of the players agree on. Players should pick a card for discussion, and discuss whether to move it.
7. At the end of the discussion, each group should have a continuum which they mostly agree with.
8. If several groups are playing at the same time, the facilitator may wish to bring the different groups' results together. Are they similar? Can someone from each group explain their choices on particular cards?

Adapting the game:

Time limitations? Don't hesitate to reduce the number of cards, or simply use the cards as starting points for discussion.

Discussion continuum developed by Ecsite, in collaboration with Barcelona Science Park, in the context of the Xplore Health project. Thanks to At-Bristol for the development of the discussion continuum format: www.at-bristol.org.uk

Agree

Disagree

Discussion Card 1

"Men who have sex with men should **not be allowed to donate blood**, to minimise the risk of HIV transmission."

Discussion Card 2

"Public health campaigns should **spread the message that condoms are not the only way to decrease the risk of HIV transmission:** reducing the viral load in people living with HIV also reduces risk, for example."

Discussion Card 3

"When applying for a job as a doctor or nurse, **no-one should be obliged to disclose their HIV status**, even if their work involves a risk of transmission."

Discussion Card 4

"If an effective vaccine is developed against HIV, **as much money as possible should be spent ensuring it gets to people most in need,** even if this means that fewer people can be vaccinated."

Discussion Card 5

"Doctors should **recommend abstinence** (not having sex) as a good way of preventing HIV transmission among young people."

Discussion Card 6

"**Giving out free, clean syringes** to known drug users is a good way for health authorities to reduce the transmission of HIV by sharing needles."

Discussion Card 7

"**Doctors should be allowed to disclose a patient's HIV+ status** to their HIV- sexual partner if it is clear that there is a risk the partner could become infected."

Discussion Card 8

"**Pregnant women should be obliged to have an HIV test** to ensure doctors can prevent the baby being infected."

Discussion Card 9

"**Emergency PEP treatment for HIV infection should not be widely advertised** to the public, because it may encourage people to take risks, knowing they can start the treatment the day after."
