# Supplementary material for: A systemic, multistakeholder participatory approach to research, innovation and action agenda setting for more integrated and effective prevention of HIV and other STIs: the case of Catalonia, Spain
Source: Front Public Health. 2026 Sep 23;14:1783030. doi: 10.3389/fpubh.2026.1783030 (PMC13644724; doi:10.3389/fpubh.2026.1783030)
Supplement: Supplementary file 2 [file Supplementary_file_1.docx]

Supplementary Material

## 1 Supplementary Figure

Supplementary Figure 1. Covariate-adjusted Actor-Partner Interdependence Model (APIM) controlling for patient cancer stage and comorbidity burden.

**Supplementary Figure 1.** **Covariate-adjusted Actor-Partner Interdependence Model (APIM) controlling for patient cancer stage and comorbidity burden.**

Notes: Standardized path coefficients are presented.**p* < 0.05. Solid lines indicate statistically significant paths (*p* < 0.05), and dashed lines indicate non-significant paths. The model was adjusted for patient cancer stage and comorbidity burden.
