# Supplementary material for: A systemic, multistakeholder participatory approach to research, innovation and action agenda setting for more integrated and effective prevention of HIV and other STIs: the case of Catalonia, Spain
Source: Front Public Health. 2026 Sep 23;14:1783030. doi: 10.3389/fpubh.2026.1783030 (PMC13644724; doi:10.3389/fpubh.2026.1783030)
Supplement: Supplementary file 3 [file Supplementary_file_2.docx]

Additional file 2. Facilitation guidelines

**Focus Grups del projecte Co-ResponsaVIHlitat
Exploració de necessitats, barreres i solucions per a la prevenció del VIH i altres ITSs**

**(guia per als facilitadors)**

**Què és Co-ResponsaVIHlitat?**

Co-ResponsaVIHlitat és un projecte impulsat pel Living Lab de Salut d’IrsiCaixa, amb el suport de projectes europeus, que té com a objectiu elaborar una Agenda Participativa per a la prevenció del VIH i altres ITSs. Compta amb la participació d’estudiants de secundària i professionals de diferents àmbits (administració, recerca i tercer sector).

Per elaborar l’agenda, els estudiants i professionals participen en grups focals. Posteriorment, els resultats seran integrats pel Living Lab, a posteriori, els resultats se us enviaran perquè els pugueu validar i establir prioritats.

Finalment, s’organitzarà una reunió de diàleg on només se sol·licitarà la presència d’alguns dels diferents actors.

**Objectiu del Grup Focal:** Explorar necessitats i barreres, per una banda, i solucions, per una altra, en forma de preguntes de recerca i línies d’actuació en el camp de la prevenció del VIH i altres ITSs per elaborar conjuntament una Agenda de R&I en Prevenció del VIH i altres ITSs.

**Programa:**

Sessió 1- Introducció al projecte Co-ResonsaVIHlitat

Sessió 2 – Exploració de necessitats i barreres

Sessió 3- Integració dels resultats en categories

Sessió 4- Recomanacions de solucions en forma de preguntes de recerca i línies d’actuació

**Descripció:**

**Sessió 1. Introducció al projecte Co-ResponsaVIHlitat**

El/la docent dóna la benvinguda. Explica el projecte, l’objectiu del grup focal, el programa i les actuacions que es duran a terme a posteriori. Informa que anirà prenent nota de les aportacions.

S’explica que els investigadors fan diferents tipus de recerca entorn de la prevenció del VIH i altres ITs, i es presenta el llistat d’àrees de recerca (Annex 1- see additional file 1).

Es convida als estudiants, organitzats en grups, a fer una cerca a internet sobre quins tipus de continguts es treballen en cadascuna d’aquestes àrees de recerca. Al final de la sessió cada grup presenta els resultats de la seva cerca.

**Sessió 2. Exploració de necessitats i barreres**

**Objectiu:** identificar necessitatsi barreres que dificulten la prevenció del VIH i altres ITSs.

**Reflexió individual (10min):** Cadascú agafa **6 post its vermells** i ha d’escriure una necessitat/barrera en cadascun dels post its amb un retolador gruixut (resumit en 2-3 paraules clau). El facilitador recorda que pot ser d’utilitat pensar en les diferents línies de recerca entorn de les quals els investigadors estan treballant, i que es van treballar a la sessió anterior).

**El facilitador/a demana a un participant que**

1) Col·loqui **un post it** en un flipchart o pissarra i l’expliqui.
2) A continuació es demana a la resta de participants si n’hi ha **més de similars**. En cas afirmatiu es col·loquen propers al post it inicial i els participants els tornen a presentar. En cas que sigui repetit **s’elimina**.
3) En un post it d’un altre color **s’identifica un clúster** que aglutina tots els d’una sola temàtica.
4) **Reflexió** entorn a aquest problema: per què és un problema? Com és que és un problema? Quina és la conseqüència?

El facilitador/a demana a un altre participant d’afegir un altre post it en una altra zona del flipchart. Es repeteix el mateix mètode fins que s’han afegit tots els post its i s’han agrupat en clústers. Per acabar de crear clústers, es demana als participants que es posin d’acord amb com el volen anomenar, i s’escriu el nom del clúster al damunt dels post its.

(Els facilitadors copien els clústers de necessitats i barreres en nous post its que enganxen en diferents categories tal i com s’indica a l’annex 2. Com a resultat s’obté un flipchart amb els clústers distribuïts en les categories, i el deixen preparat fins la propera sessió).

**Sessió 3- Integració dels resultats en categories**

El facilitador/a mostra el flipchart amb diferents categories on hi ha col·locat tots els clústers i demana als participants de validar la classificació que ha fet el facilitador i d’acabar de completar la taula.

El Facilitador:

1) Explica les **categories** i demana als participants si poden reconèixer els clústers, si estan d’acord en com estan ubicats
2) Demana als participants que s’apropin al flipchart i que donin feedback de la distribució de post its i si creuen que en manca algun.
3) Modera una reflexió: hi ha zones que han quedat sense post its, com és? Es demana que en **post its grocs** afegeixin noves necessitats i barreres a les zones que estan més buides de post its. El facilitador recorda que pot ser d’utilitat pensar en les línies de recerca que s’han treballat.
4) Recull els post its, els col·loca al flipchart dins les categories i es comenten un per un.
5) Facilita una discussió dels resultats amb el grup.

**Sessió 4- Recomanacions de solucions en forma de preguntes de recerca i línies d’actuació**

El facilitador convida als participants a elaborar recomanacions de solucions en forma de preguntes de recerca i línies d’actuació.

El facilitador/a:

1) Explica que hi ha diferents tipus de recerca que podrien contribuir amb coneixement que podria ajudar a donar resposta a les necessitats i barreres. De fet, la solució no serà sempre una manca de coneixement, i per tant no sempre es requerirà recerca. Però en molts casos la recerca pot aportar nous punts de vista que poden ser d’utilitat.
2) Convida els assistents a elaborar les recomanacions de solucions. En la mateixa taula amb les categories i clústers, afegeixen noves tarjes verdes amb solucions. L’exercici es duu a terme a nivell de grup.
3) Es facilita una discussió dels resultats.
4) Dóna les gràcies als participants pel seu input.
5) Se’ls recorda que s’enviarà un document amb els resultats i se’ls demanaran comentaris i prioritzacions i que alguns se’ls demanarà si poden contribuir a una darrera sessió de diàleg amb els diferents actors.

**Annex 2: Categories per ubicar en un flipchart horitzontal.**

Ajuden a fer un anàlisis global del sistema on s’aplica la prevenció del VIH i altres ITSs, tenint en compte molts diversos aspectes i actors. Serviran per distribuir els post its on s’identifiquen necessitats i barreres recollides durant la sessió 2, i també solucions recollides durant la sessió 4 tal i com es descriu a la guia.

**Categories:** Quines necessitats i barreres tenim per a la prevenció en VIH i altres ITSs amb...: (*Ubicar en les diferents columnes els noms dels clústers obtinguts durant la sessió 2 i les solucions desenvolupades durant la sessió 4)*

| **Funding model**  for research and innovation and for prevention interventions | **Access to knowledge and research on HIV and other STIs**  including access to dissemination of knowledge and access for stakeholders to participate in research processe | **Physical or virtual environment**  including school facilities and facilities for night, sports, leisure and healthcare and virtual spaces such as computers, tablets and social networks | **Legislation and regulation**  both inside and outside schools | **Values, culture and climate**  in the schools and within families, affecting relationships with teachers and among students and families | **Spaces for interaction among actors**  such as teachers, students, psychologists, CSOs or healthcare providers, and including both time and space for reflection and training |
| --- | --- | --- | --- | --- | --- |
|  |  |  |  |  |  |
|  |  |  |  |  |  |
|  |  |  |  |  |  |
|  |  |  |  |  |  |
|  |  |  |  |  |  |

Principio del formulario

¡Gracias!

Tu contribución se utilizará para mejorar la calidad de la traducción y podría mostrarse a los usuarios de forma anónima.

Enviar

Cerrar

Mogelijke thematische indeling exercitie 1 (zie ook causaalanalyse) Psychosociaal: o Interpersoonlijke interacties en relaties o Attitudes van mensen in omgeving/maatschappij / ondersteuning o Psychosociale zorg (voorzieningen / lotgenotencontact) o Angst o Schuld o Schaamte o Vrouwenzaken o Acceptatie door brandwondenslachtoffer zelf o Herbeleving Lichamelijk: o Pijn o Jeuk o Bewegingsbeperking / immobiliteit o Beperkte orgaanfuncties o Lichamelijke zorg Zorg / behandeling: o EHBO o Acute zorg o Wondbehandeling o Littekenbehandeling o Plastische chirurgie o Psychosociale zorg o Revalidatie o Zelfzorg Belangenbehartiging o Financieel (o.a. arbeidsongeschiktheid) o Juridisch (o.a. schadeclaims) Preventie

Definiciones de Mogelijke thematische indeling exercitie 1 (zie ook causaalanalyse) Psychosociaal: o Interpersoonlijke interacties en relaties o Attitudes van mensen in omgeving/maatschappij / ondersteuning o Psychosociale zorg (voorzieningen / lotgenotencontact) o Angst o Schuld o Schaamte o Vrouwenzaken o Acceptatie door brandwondenslachtoffer zelf o Herbeleving Lichamelijk: o Pijn o Jeuk o Bewegingsbeperking / immobiliteit o Beperkte orgaanfuncties o Lichamelijke zorg Zorg / behandeling: o EHBO o Acute zorg o Wondbehandeling o Littekenbehandeling o Plastische chirurgie o Psychosociale zorg o Revalidatie o Zelfzorg Belangenbehartiging o Financieel (o.a. arbeidsongeschiktheid) o Juridisch (o.a. schadeclaims) Preventie

Sinónimos de Mogelijke thematische indeling exercitie 1 (zie ook causaalanalyse) Psychosociaal: o Interpersoonlijke interacties en relaties o Attitudes van mensen in omgeving/maatschappij / ondersteuning o Psychosociale zorg (voorzieningen / lotgenotencontact) o Angst o Schuld o Schaamte o Vrouwenzaken o Acceptatie door brandwondenslachtoffer zelf o Herbeleving Lichamelijk: o Pijn o Jeuk o Bewegingsbeperking / immobiliteit o Beperkte orgaanfuncties o Lichamelijke zorg Zorg / behandeling: o EHBO o Acute zorg o Wondbehandeling o Littekenbehandeling o Plastische chirurgie o Psychosociale zorg o Revalidatie o Zelfzorg Belangenbehartiging o Financieel (o.a. arbeidsongeschiktheid) o Juridisch (o.a. schadeclaims) Preventie

Ejemplos de Mogelijke thematische indeling exercitie 1 (zie ook causaalanalyse) Psychosociaal: o Interpersoonlijke interacties en relaties o Attitudes van mensen in omgeving/maatschappij / ondersteuning o Psychosociale zorg (voorzieningen / lotgenotencontact) o Angst o Schuld o Schaamte o Vrouwenzaken o Acceptatie door brandwondenslachtoffer zelf o Herbeleving Lichamelijk: o Pijn o Jeuk o Bewegingsbeperking / immobiliteit o Beperkte orgaanfuncties o Lichamelijke zorg Zorg / behandeling: o EHBO o Acute zorg o Wondbehandeling o Littekenbehandeling o Plastische chirurgie o Psychosociale zorg o Revalidatie o Zelfzorg Belangenbehartiging o Financieel (o.a. arbeidsongeschiktheid) o Juridisch (o.a. schadeclaims) Preventie

Ver también

Traducciones de Mogelijke thematische indeling exercitie 1 (zie ook causaalanalyse) Psychosociaal: o Interpersoonlijke interacties en relaties o Attitudes van mensen in omgeving/maatschappij / ondersteuning o Psychosociale zorg (voorzieningen / lotgenotencontact) o Angst o Schuld o Schaamte o Vrouwenzaken o Acceptatie door brandwondenslachtoffer zelf o Herbeleving Lichamelijk: o Pijn o Jeuk o Bewegingsbeperking / immobiliteit o Beperkte orgaanfuncties o Lichamelijke zorg Zorg / behandeling: o EHBO o Acute zorg o Wondbehandeling o Littekenbehandeling o Plastische chirurgie o Psychosociale zorg o Revalidatie o Zelfzorg Belangenbehartiging o Financieel (o.a. arbeidsongeschiktheid) o Juridisch (o.a. schadeclaims) Preventie
