# Supplementary material for: A systemic, multistakeholder participatory approach to research, innovation and action agenda setting for more integrated and effective prevention of HIV and other STIs: the case of Catalonia, Spain
Source: Front Public Health. 2026 Sep 23;14:1783030. doi: 10.3389/fpubh.2026.1783030 (PMC13644724; doi:10.3389/fpubh.2026.1783030)
Supplement: Supplementary file 4 [file Supplementary_file_4.docx]

***Additional file 4. Data analysis: from raw data to the final agenda (items and codes of needs and barriers, items and codes of solutions, themes of 1^st^ thematic analysis and themes of 2^nd^ thematic analysis, all organized in different missions)***

**Color coding of items according to the FG in which they were collected (original language: Catalan):** *FGs with students****,*** FGs with civil society organisations, FGs with experts in behavioural and structural aspects, FGs with experts in communication and education, FGs with experts in biomedical prevention and barrier methods.

| **Number** | **Missions** -underlined text identifies the content that comes from these Themes of barriers, needs and solutions | **Themes** | **Codes of needs and barriers** | **Items of needs and barriers- students** | **Items of needs and barriers- professionals** | **Codes of solutions** | **Items of solutions-**  **students** | **Items of solutions-**  **professionals** | **Themes of R&I topics and actions**  **in the final agenda – 2^nd^ thematic analysis** |
| --- | --- | --- | --- | --- | --- | --- | --- | --- | --- |
| 1 | Resources for Research and Innovation (R&I), prevention interventions and health inequalities | Lack of funding for the implementation of HIV and other STIs prevention interventions  REFINMENT TO:  Lack of funding for the implementation of HIV and other STIs prevention interventions (lack of human, structural and economic resources) | More resources for the prevention of HIV and other STIs  Lack of economic and human resources  Lack of structural resources | Recursos de salut publica  Ajuts econòmics per a proves i tractaments  Financiar els mètodes de prevenció perquè siguin encara més econòmics i accesibles.  Incrementar el finançament per a la prevenció | Manca de finançament  Manca de recursos per a les ITSs i farmàcs (econòmics, tècnics, etc.)  Recursos limitats  Manca de recursos  Manca de recursos (econòmics, recursos humans) | More resources for the prevention of HIV and other STIs | Es poden desenvolupar mètodes de prevenció més còmodes i més cost-efectius per evitar la transmissió del VIH i altres ITSs?  Test de diagnostic gratuït cada mes  Millorar el preu i la distribució de condons  Per què no es redueix el preu dels condons? I dels farmacs?  Quants diners s’han invertit? Perquè encar no tenim més mètodes preventius? | Cercar més recursos per a la prevenció del VIH i altres ITSs involucrant als diferents actors socials  Més inversió en prevenció | 1.Better balance between biomedical and socio-educational models with more human, structural and economic resources for communication and educational interventions, for research and rigorous evaluation (qualitative and longitudinal), for early detection and control of STIs and for complementary services, such as social services, especially focused on vulnerable communities |
|  |  | Lack of resources for R&I | Lack of resources for R&I | El marc econòmic actual provoca manca de recursos per a la recerca i hauria de ser una prioritat per a la nostra societat.  Necessitat d’incrementar la inversió per a la recerca en prevenció de l’VIH, en concret de la vacuna  Millorar la línia d’investigació en persones autoimmunes.  La manca o davallada de recursos econòmics, i més en temps de crisi, en investigació obstaculitza avançar en tots els aspectes del VIH, inclosa la prevenció.  Ni governs ni empreses privades aporten prou en el marc de la recerca.  Falta més inversió de diners i més dedicació a les diferents línies de recerca.  Quants diners s'han invertit?  I per què no hi ha més mètodes de prevenció?  Recursos per investigació i  Recursos públics per a la investigació  Ajudar amb subvencions a la recerca  Incrementar el pressupost per millorar els medicaments, evitant efectes secundaris i pèrdua d’eficàcia.  Estandaritzar l’ús de medicaments d’absorció sistèmica i descartar els de teràpia tòpica.  Manca de voluntaris per a la recerca  Tractaments incomodes i llargs |  | Lack of resources for R&I | Anàlisis comparatiu de la inversió en recerca i en prevenció del VIH i altres ITS i dels costos econòmics i socials respecte a d’altres malalties a nivell regional, nacional i internacional  Hi ha prou inversió en l’estudi de la vacuna preventiva? I en l’estudi de la PrEP? I en la millora dels fàrmacs existents per a que siguin capaços de curar?  Com es podria millorar la inversió en recerca per a la prevenció del VIH i altres ITS?  Una proposta factible donada la poca inversió per la investigació seria afegir una casella a la “declaració de renda”. |  |  |
|  |  | Lack of resources for communication interventions  REFINMENT TO: communication and educational interventions  REFINMENT TO: as priority is given to medicalized interventions that respond to industry interests (pharmaceutical, alcohol, drugs, etc.) | Lack of resources for communication interventions | Més campanyes oficials en prevenció del VIH. | Diners condicionen els espais per a poder treballar la prevenció VIH/ITS  Interessos econòmics | Resources for communication and educational interventions  Priority is given to medicalized interventions that respond to industry interests (pharmaceutical, alcohol, drugs, etc.) |  | Invertir en més campanyes i programes educatius adreçats a diferents comunitats ja que es prioritzen les intervencions medicalitzades que responen als interessos de la indústria (farmacèutica, alcohol, drogues...) |  |
|  |  | Lack of resources for early detection and control of STIs and for social services | Resources for early detection |  | Manca de recursos pel diagnòstic precoç (test, EC), per la prevenció i control i per als serveis socials. | Resources for early detection, control of STIs and for social services |  | Més inversió en prevenció, control, serveis socials, educació, test... |  |
|  |  | Lack of resources for rigorous evaluations (longitudinal)  REFINEMENT TO: (qualitative and longitudinal) | Lack of resources for longitudinal studies |  | Manca recursos per estudis longitudinals d’impacte | Resources for rigorous evaluations (qualitative) |  | Finançament per a l’avaluació dels programes que s’estan duent a terme (qualitatius) |  |
|  |  | Lack of non-precarious resources to change inequalities, with political support for the most vulnerable sectors | Lack of non-precarious resources to change inequalities, with political support for the most vulnerable sectors |  | Poc accés a recursos que no siguin precaris per a canviar les desigualtats  Suport polític per als més vulnerables |  |  |  |  |
|  |  | Urgent need to review the financing model for interventions, which must be co-defined between administrations and third sector entities | Need to review the funding model for internvatnions |  | Necessitat de revisar el model de finançament de les intervencions | Redesign the funding model for interventions which must be co-defined between administrations and third sector entities |  | Redesign of the funding model for interventions using a participatory process that includes administration and the third sector | 2. Redesign of the funding model for interventions using a participatory process that includes administration and the third sector |
| 2 | Governance, regulation and human rights | Need to consider sexual and reproductive rights in a transversal way in all prevention actions | Need to consider sexual and reproductive rights in a transversal way in all prevention actions  Legislation is discriminatory, sometimes influenced by religions i.e. in France men who have sex with men (MSM) cannot donate blood  Strategies should be based on human rights | Cal evitar que les lleis discriminin, com és el cas de la prohició als homosexuals de donar sang a França.  Estratègies basades en els drets humans  Lleis retrogrades basades en creences religioses.  Per què els homosexuals no poden fer transfusions? (França) | Necessitat de tenir en compte els drets sexuals i reproductius de manera transversal en tota actuació de prevenció | Legislation to protect people living with HIV from social, public services and workplace discrimination  REFINEMENT TO:  Raise awareness and facilitate open discussions on sexual and reproductive rights and other human rights to avoid controversies regarding whether it is necessary to define new specific legislations for people living with STIs  Laws against bullying and cyberbullying to avoid stigmatization | Fer lleis que protegeixen a les persones seropositives per no ser discriminades en els treballs i en la societat en general.  Deurien haver lleis contra el bullying i el cyberbullying per evitar l’estigmatització.  Actualitzar la legislació en la consideració de la SIDA com una malaltia altament contagiosa que limita l’accés a determinats serveis en igualtat de condicions | Anàlisis de com es fomenten o bé es vulneren els drets sexuals i reproductius en les diferents actuacions de recerca i de foment de la prevenció del VIH i altres ITSs  Polítiques de foment dels DSIR en els projectes de recerca i en les intervencions | 3.Raise awareness and facilitate open discussions on sexual and reproductive rights and other human rights to improve their coverage in all prevention actions and research projects, to avoid controversies regarding whether it is necessary to define new specific legislations for people living with STIs and to clarify when health professionals can bypass the right to confidentiality because of HIV transmission risk |
|  |  | Need to disseminate peoples’ rights and duties regarding topics related to HIV and other STIs and the reasons why it is not necessary to define new specific legislation | Regulate duties of people living with HIV  REFINEMENT TO: Need to disseminate peoples’ rights and dutes regarding topics related with HIV and other STIs and the reason why it is not necessary to define new specific legislation | Regulació de la obligatorietat d’informar de l’estat serològic i de les implicacions legals en cas de contagi  La gent té dret a saber si la seva parella pateix el VIH. Les lleis haurien de contemplar-ho, així com les implicacions legals en cas de contagi. | Falta de capacitació legal i apoderament per desconstruir l’estigma | Research on how to regulate the rights and duties of PLHIV and the legal implications and the risks and benefits of this regulation while simultaneously safeguarding individual freedom and the right to privacy  REFINEMENT TO: Need to disseminate peoples’ rights and dutes regarding topics related with HIV and other STIs and the reason why it is not necessary to define new specific legislation | Com s’haurien de regular els drets i deures de les persones que conviuen amb el VIH i quines implicacions legals hi hauria d’haver en cas de transmissió?  Quins són els riscs i els beneficis de crear o no una llei específica?  Com normalitzar l’existència de malalties infeccioses, protegint al mateix temps la llibertat individual i el dret a la pròpia intimitat? |  |  |
|  |  | Clarify the rights and duties of health professionals when they bypass the right to confidentiality in order to comprehensively address an HIV transmission risk | Need to respect confientiality and also minimize the transmission risk  REFINEMENT TO: Clarify the rights and duties of professionals when they bypass the right to confidentiality in order to comprehensively address and HIV transmission risk |  | Necessitat de respectar la confidencialitat i minimitzar el risc de transmissió | Rights and duties of health professionals described in an existing protocol |  | Protocol per a professionals de la salut d’estudi de contactes (publicat recentment pel Departament de Salut)  Promoure intervencions d’estudis de contactes |  |
|  |  | There is a lack of effort to preserve ethical aspects in research projects, especially regarding the rights of volunteers | Lack of effort to preserve ethical aspects in research projects, especially regarding the rights of volunteers |  | Manquen esorços per preservar es aspectes ètics en els projectes de recerca, especialment pel què fa als voluntaris |  |  |  |  |
|  |  | Prevention model isolated from the cascade of health services for people living with HIV | Prevention model isolated from the cascade of health services for people living with HIV |  | Model de prevenció aïllat de la cascada de serveis de salut per al les persones que viuen amb el VIH | Redesign the prevention model |  | Com es pot integrar la prevenció en la cascada de serveis de salut que s’ofereixen a les persones que viuen amb el VIH?  Polítiques científiques que integrin el tractament de la infecció pel VIH amb les intervencions de prevenció | 4. Redesign the prevention model to make it more focused on health promotion rather than disease prevention, more integrated (i.e., with other health problems and healthcare services, with social services from different geographical areas) and more focused on ethics, human rights and social aspects, such as inequalities. The governance of the new model should be based on co-responsibility in decision-making (e.g., on R&I priorities, priority target groups, design and implementation of interventions) including the participation of diverse stakeholders (i.e. policy makers, healthcare professionals, researchers from different disciplines, social services and the social community), should use innovative relational dynamics with a special focus on dealing with power relations |
|  |  | Prevention model based on biomedical interventions without sufficiently addressing ethical issues, human rights and social aspects, such as inequalities | Prevention model based on biomedical interventions with low priority for social sciences and public health |  | Polítiques públiques tenyides per la biomedicina  Menys valoració de les ciències socials vs les ciències biomèdiques  David (salut pública) i Goliat (assistència)  Segrest del relat de la prevenció pels lobbies | Lack of balance between biomedical interventions and interventions addressing social aspects |  | Model predominant i impacte: Anàlisis del model de prevenció que predomina en les actuacions en aquest camp i sobre l’impacte que té sobre la prevenció  Anàlisis del balanç entre inversió en recerca i intervencions biomèdiques i en recerca i intervencions entorn a aspectes socials  Integració dels dos models: Com vèncer la disparitat de llenguatges i mirades i com facilitar projectes transdisciplinaris que contemplin els dos models i alhora la participació de la comunitat  Polítiques que fomentin que el model quedi equilibrat per assegurar que assolim la prevenció mitjançant intervencions biomèdiques i alhora i en paral·lel amb intervencions socials. |  |
|  |  | Prevention model conditioned by a non-collaborative economic model | Prevention model conditioned by a non-collaborative economic model |  | Model econòmic vigent vs Economia col·laborativa ètica | Current economic model influences prevention |  | Com influencia el model econòmic vigent en el model de prevenció i com aquest es podria transformar en un model més col·laboratiu i menys discriminatori?  Anàlisis comparatiu de com les intervencions fomenten models inspirats pel model econòmic vigent o per models col·laboratius |  |
|  |  | Co-responsibility model in decision-making (e.g., shared decision-making for research and innovation priorities and priority target groups): It is necessary to change the model of interaction between different actors in the biomedical community (healthcare professionals, researchers from different specialities), the social services, the social community and others using innovative relational dynamics currently based on a communication model, to evolve towards a co-responsibility model in which prevention is a shared project | Lack of consensus among different stakeholders  Lack of shared responsibility  Transformation of the current model towards shared responsibility | Consensuar i posar ordre en els valors i opinions de les persones, per a determinar una resposta, i que els juristes puguin marcar quina línia ha de seguir un país. Estratègies basades en .... el respecte mutu, la inclusió, la responsabilitat i l’esforç comú per tal de reduir la transmissió de VIH. | Necessitat d’un nou model de relació entre comunitat biomèdica i la comunitat cap a un model de responsabilitat compartida (tant a nivell de relació individual metge-pacient, com a nivell de sistema)  Diferències entre comunitat biomèdica i comunitat  Les decisions no es basen en un consens  Falta de coneixement real de les necessitats dels diferents grups poblacionals: el model no respon a l’actual canvi en les dinàmiques relacionals entre la comunitat, la societat civil i altres actors | Transformation of the current model towards shared responsibility  Need to define R&I and interventions with participatory approaches  Need to change the model of interaction between different actors in the biomedical community (healthcare professionals, researchers from different specialities), the social services, the social community and others using innovative relational dynamics currently based on a communication model, to evolve towards a co-responsibility model in which prevention is a shared project  Research on methodologies for participatory research agenda setting  Set up a participatory research agenda in Catalonia | Com es podria facilitar la participació dels diferents actors socials (professionals de la salut, investigadors, indústria, entitats de la societat civil, gestors de polítiques públiques, comunitat educativa, ciutadania) en la presa de decisions en matèria de prevenció tant a nivell individual com col·lectiu?  Com es defineixen les prioritats en recerca i intervencions de prevenció del VIH i altres ITSs? (ex. Baixa inversió en microbicides)  En decisions sobre missatges preventius: com assegurar que els actors involucrats en la prevenció tinguin accés a la informació i puguin participar en la presa de decisions sobre com prioritzar missatges preventius i com facilitar que en certes circumstàncies sigui la ciutadania qui pugui decidir comptant amb tota la informació-ex. No difondre el missatge de que les persones que estan indetectables no poden transmetre el VIH, o davant brots d’Hepatitis C no facilitar la informació per no crear alarma)  Quin és l’impacte de determinades mesures participatives (com ara el projecte Co-ResponsaVIHlitat) en la presa de decisions?  Política internacional: Millorar la cooperació intergovernamental per eradicar el VIH i altres ITS a nivell global | Com es podrien identificar necessitats no cobertes i prioritzar millor la recerca i la innovació que es duu a terme? Qui hi participa? Com es podria millorar?  Mètodes per prioritzar les línies de recerca i innovació amb participació (agenda de recerca integral): Anàlisis de processos de priorització coordinada entre diferents actors socials. Com implementar una agenda de recerca més integral, que sigui més interdisciplinària i alhora integri les persones afectades?  Promoure la co-definició d’una Agenda d’R&I en promoció de la salut i en especial de la salut sexual a Catalunya o d’un Pla Estratègic de Catalunya: per co-definir i co-implementar una estratègia o Agenda d’R&I en prevenció amb la col·laboració dels diferents actors socials.  Desenvolupar una agenda compartida que promogui la presa de decisions participativa i basada en evidència (i.e. polítiques de priorització coordinada entre diferents actors socials)  Crear una plataforma transdisciplinària per a la presa de decisions en prevenció perquè estigui més alineada amb les necessitats dels diferents col·lectius)  Els processos per decidir la població diana no es duen a terme de manera participativa (i.e. intervencions universals vs universalisme proporcional, per a qui? Qui decideix?: Cal co-decidir si volem continuar deixant de banda alguns col·lectius que tot i tenir menys risc també tenen dret a intervencions. La co-decisió podria ajudar a trobar un millor balanç de priorització de públics diana. |  |
|  |  | Need for a model of shared responsibility for the design and implementation of actions that are least fragmented, have a better relationship between policy, research and application, and include greater participation of the social community | Prevention programmes do not respond to the real needs of different communities |  | Els fonamentalismes condicionen la nostra pràctica i polítiques  Programes de prevenció mal dissenyats | Need for a model of shared responsibility  Need for greater participation of the social community to avoid the current fragmentation between policies, research and application  Research on participatory and systemic approaches to design interventions  Research on how to promote a more inclusive governance | Com es pot millorar la coordinació dels diferents actors que participen en les diferents línies d’actuació per a la prevenció del VIH i altres ITS? | Grau de participació: Quin és el grau de co-responsabilitat en la prevenció de les ITSs?  Anàlisis del grau de participació de la comunitat: qualitat de la participació i condicions (i.e. si es tracta de voluntarisme, si existeixen plans de formació i de reconeixement amb honoraris)  Mètodes per co-dissenyar campanyes i intervencions amb sinergies entre la comunitat científica, els experts en màrqueting i comunicació i les entitats de la societat civil.  Metodologies de co-creació  Promoure intervencions no compartimentades: que es promoguin des de tots els actors socials i no només des d’Agències de Salut Pública.  Coordinació de la participació: Promoure la participació dels diferents actors amb el lideratge o coordinació per part d’un actor que reculli les bases d’actuació de tots els actors en forma d’estratègies, plans....  Invertir en polítiques que fomentin més el treball col·laboratiu en el disseny: Implicar els diferents actors socials en el disseny de les campanyes i intervencions (incloent els destinataris) basades en evidència científica  Mètodes de governança: Com facilitar un sistema d’R&I més obert i inclusiu, que compti amb més participació de la comunitat en les diferents etapes del procés de prevenció del VIH i altres ITSs.  Promoure el disseny de campanyes i intervencions en col·laboració entre els diferents actors en espais de co-creació, i.e. amb la comunitat, la comunitat científica i els experts en màrqueting i en comunicació. Promoure també la comunicació i interacció entre les diferents fonts d’informació i els diferents emissors de missatges (i.e. FLS i CEEISCAT...).  Espais de co-creació: Fomentar espais de coordinació/comunicació/co-creació de diferents actors socials per tal de facilitar sinergies i d’optimitzar recursos tant de recerca com de serveis assistencials i intervencions |  |
|  |  | Difficulty for some groups to participate due to power relations | Difficulty for some groups to participate due to power relations  Difficulty to participate during decision making and implementation | Podríem gastar recursos en altres malalties? El VIH s'ha transformat en crònica als països rics.  Podríem transformar en cròniques altres malalties.  Perquè es dediquen tants recursos al VIH i no a altres malalties pitjors com el envelliment? | Dificultat de participar d’alguns col·lectius: tant en la presa de decisions com en la implementació d’intervencions i recerca, donat que hi ha comunitats que lideren i monopolitzen el relat i duen a una manca de reconeixement de les entitats de la societat civil  Manca de reconeixement de les entitats del tercer sector | Difficulties to facilitate participatory approaches  Need to recognize volunteers from the community  Research on pariticipatory approaches  Recognition of successful participatory practicies | Grau de participació de les entitats de la societat civil: Quins és el grau de participació de les entitats de la societat civil en la presa de decisions en les polítiques de prevenció del VIH i altres ITSs? | Anàlisis dels actors representats en la presa de decisions en prevenció i dels que queden poc representats  Recerca entorn a metodologies per involucrar els afectats en la recerca  Anàlisis de bones pràctiques per fer participar els no representats i per fer intervencions on siguem proactius i anem a buscar el públic objectiu  Anàlisis de com es prenen les decisions en les prioritats en prevenció. Estudi del cas de la PrEP: transparència, interessos dels diferents actors, punts de vista, necessitat de promoure més el preservatiu vs aplicació de la PrEP etc.  Reconeixement de la participació: Desenvolupar plans de reconeixement professionalitzat dels voluntaris de la comunitat |  |
|  |  | Interventions are fragmented among certain health problems, diseases and geographical areas and focus on diseases rather than health promotion | Interventions are fragmented among certain health problems, diseases and geographical areas  Interventions focus on diseases rather than health promotion |  | Manca de coordinació i d’atenció i prevenció integral. Optimització de recursos  Les intervencions estan compartimentades en determinats problemes de salut o malalties: drogues, alcohol, tabac, VIH, ITS... són poc globals i es focalitzen en malalties i no en la promoció de la salut |  |  |  |  |
|  |  | Prevention model too influenced by HIV | HIV is socially perceived as a boring topic |  | El “rollo” del VIH | Prevention model influenced by negative perception of HIV and other STIs |  | Com es diferencia la percepció del VIH de la d’altres ITSs i com influencia la percepció negativa del VIH en la prevenció de les altres ITSs? |  |
|  |  | Need to improve the doctor-patient relationship: the patient perceives the doctor as someone who is paternalistic, solves problems, does not empathize, blames them and does not understand the new paradigm of sexuality | Need to improve the doctor-patient relationship  The patient perceives the doctor as someone who is paternalistic, solves problems, does not empathize, blames them and does not understand the new paradigm of sexuality |  | Manca formació professionals  Col·laboració professionals i pacients: els professionals es perceben com algú que alliçona, resol problemes sense empatitzar, jutja i no entén en nou paradigma de la sexualitat | Need for a relationship where decisions are shared respecting the sexual rights and avoiding too much focus on risks |  | Com canviar el model de relació metge pacient per un model biogràfic que acompanya en la presa de decisions en un marc dels drets sexuals i no tant focalitzat en els riscs?  Elaborar un directori de recursos i XXSS perquè els professionals sanitaris puguin facilitar informació als pacients per a l’acompanyament | 5.Improve the doctor-patient relationship: the patient currently perceives the doctor as someone who is paternalistic, solves problems, does not empathize, blames them and does not understand the new paradigm of sexuality |
| 3 | Information, education and communication | Low knowledge on HIV and STIs as well as on transmission routes, preventive methods, including alternative methods such as PrEP*, the consequences of infections and the need to take care of one’s health, including one’s sexual health | Low knowledge on HIV and STIs  Low knowledge on transmission routes, preventive methods, including alternative methods such as PrEP*  Low knowledge on the consequences of infections and the need to take care of one’s health, including one’s sexual health | Com em puc infectar? Què cal evitar?  Es possible la infecció prenent anticonceptius?  Existeixen tractaments per eliminar el VIH?  Hi ha tractaments post-infecció disponibles?  Hi ha altres mesures preventives a part del preservatiu?  Baix coneixement sobre el tema  Hi ha gent afectada que encara no en té coneixement.  Des del punt de vista psicològic, caldria que la gent conegués els símptomes de la malaltia.  Poc coneixement específic sobre vies de prevenció  Quants infectats hi ha a Barcelona?  Per què els fàrmacs no són efectius?  Per què no s’ha trobat encara una cura? Quan podria tardar?  Fa falta millorar els detectors de VIH i els efectes secundaris del tractament?  No saber que estàs infectat per la manca de símptomes  S’ha de tenir autoconeixement i cuidar-se un mateix. És, del tot necessari, establir mecanismes que incrementin el coneixement, per part de la població general i a nivell mundial.  Cal incidir de manera important en la prevenció per relacions sexuals sense protecció  On es fa recerca del VIH?  Per què no fan més reportatges sobre el VIH?  Com es transmet el VIH?  Sempre que mantens relacions sexuals amb un infectat, et pots contagiar?  Com puc evitar el contagi? En el cas de tenir el VIH, què puc fer?  Quin percentatge de població s’infecta en un any? I cada deu persones? Quina és l’esperança de vida d’una persona amb VIH? | -Manca de coneixements sobre les ITS/VIH i de les complicacions  - Baixa percepció de gravetat de les malalties (educació en salut general)  - Falta d’informació (rutes, TASP, transmissió...)  - Falta informació de les persones usuàries (VIH/ITS). Nul·la educació sexual.  - Baixa disponibilitat de literatura científica  - Desinformació poblacional i dels decisors | More quantity and quality of information and education that make use of different channels and target different audiences with personalized appraoaches | Anàlisis del grau de coneixement: Quin coneixement tenen els diferents públics sobre el VIH/SIDA i les ITS?  Més quantitat i qualitat de campanyes informatives i educatives sobre VIH i altres ITS: en que es treballin continguts relacionats amb les diferents categories d’aquesta agenda i on es faci ús de diferents canals, que s’imparteixin per part de diferents actors i que es dirigeixin a diferents públics i entorns (famílies i joves, col·lectius de risc, etc)  Programes per treballar la sexualitat en família: Educació sobre com tractar la sexualitat en la família.  Campanyes i programes educatius sobre l’ús del preservatiu i altres mètodes: Facilitar formació sobre l’ús correcte del preservatiu i altres mètodes preventius incloent-ne per exemple mètodes com la PrEP | Quin coneixement tenen els diferents públics sobre el VIH i altres ITSs? Quin és el grau de coneixement sobre prevenció de ITSs entre la població?  Quin coneixement tenen els diferents públics sobre les vies de transmissió a través de diferents pràctiques sexuals? Quin coneixement tenen sobre les conseqüències de la infecció pel VIH i altres ITSs i què significa conviure amb el VIH? Quin coneixement tenen sobre la necessitat de cuidar de la nostra salut?  Quin és el coneixement social de mètodes de prevenció alternatius al preservatiu com ara la PrEP?  Incorporar missatges preventius en mitjans de comunicació  Campanyes i programes educatius sobre l’ús del preservatiu I altres mètodes.  Programes amb formació sobre vies de transmissió mitjançant diferents pràctiques sexuals: Incloure informació sobre les vies de transmissió del VIH i altres ITSs mitjançant diferents pràctiques sexuals i sobre les seves conseqüències i la necessitat de cuidar la nostra salut.  Informar els comunicadors: i líders d’opinió de la necessitat de promoure missatges de sexe segur | 6.Increase literacy on STIs with quantity and quality of information and with (non-) formal education interventions that use a salutogenesis approach, make use of different channels, facilitate reflection spaces, bring research and education closer together and target both different audiences with personalized approaches and the general public to increase:  - Knowledge on transmission routes, preventive methods (including alternative methods such as PrEP*), the consequences of infections and the need to take care of one’s health (including but not limited to one’s sexual health, wrong information that acts as barriers to getting tested, sexuality with a good balance between the perception of risk and the focus on pleasure)   - Competencies of researchers to increase application of results, of professionals to promote affective-sexual education, and of all stakeholders to distinguish between non-rigorous information and rigorous and contrasted information   7.Facilitate training for professionals on how to educate about affective-sexual aspects (currently, they mostly focus on biological aspects) |
|  |  | Perception and wrong information about HIV and other STIs as a barrier to getting tested | Wrong information that acts as barriers to getting tested | Informació errònia o escassa - Falses creences sobre el VIH i del sida com a una malaltia del passat i que es pot curar.  Falsos mites sobre el VIH  Idees preconcebudes(religio, ,tradició...)  Confusió a causa d’internet. Informació manipulada o poc fiable.  La informació pot no ser sempre fiable  Perill de que la informació per Internet no sigui verídica | Percepció de risc i estigma |  |  |  |  |
|  |  | Need to improve the quantity and quality of information and education interventions: making use of different channels, bringing research and education closer together, aiming at different audiences with personalized approaches and, combined with interventions, targeting more general audiences and focusing less on preventing risk and more on promoting pleasure | Need to improve the quantity and quality of information and education interventions  Need to make use of different channels  Need to bring research and education closer together  Need to target different audiences with personalized approaches and, combined with interventions, targeting more general audiences  Need to focus less on preventing risk and more on promoting pleasure | Les campanyes no són efectives i hi ha molt poques oportunitats de que els alumnes s’impliquin realment en el tema.  Per a què la informació realment influeixi en la presa de decisions dels joves, cal que es transmeti d’una manera molt propera a ells, per exemple, parlant amb pacients d’edat i context social similar.  Poc impacte de les campanyes de publicitat.  Existeix informació de qualitat suficient per a l’alumnat però no tothom ha accedit o sap que existeix.  Manca d’efectivitat de les campanyes publicitàries  Falta Coneixement de com fer dissenyar estratègies efectives  Fomentar l’ús de les xarxes socials per disseminar l’estratègia preventiva  Apropar el tema de la sexualitat i les campanyes informatives als joves  Si estàs de festa nocturna no estàs pendent per rebre informació del VIH.  Influència dels media (pelis, líders en XXSS).  És bo disposar d’Internet  Per internet, jocs, anunci, web informatives.  Per què no rebem prou informació pels medis de comunicació? Per què no es fa publicitat informativa del VIH?  Interacció adolescent científic  Fer projectes com l’actual.  Dificultat d’accés a la recerca per part dels adolescents (aïllament dels científics)  Treball de recerca dels alumnes, que arribin als científics i que puguin tenir un impacte.  Podria ser molt útil que en pàgines web sobre el tema, es pogués preguntar directament a professionals.  Existeixen campanyes orientatives a les escoles, campanyes de promoció i organitzacions com XploreHealht que ajuden als alumnes a ser part del procés de recerca.  Realització del projecte Co-ResponsaVIHilat  La ignorància o el desconeixement de les fases de la malaltia podrien millorar-se amb la interacció amb científics. | No parlem de sexe a nivell de societat – Manca de campanyes comunitàries  Consens entre informació, missatges...  Si no ets grup de risc no surts al mapa  No som capaços d’oferir materials innovadors: “Stop folletos”  Dissenyar campanyes “personalitzades” i no tant generals  Arribar a certs públics  Adaptació dels missatges segons la població  Ser capaços de dissenyar campanyes personalitzades basant-nos en perfils XS (amb CI)  Massa contingut però poc aterrat  Xarxes socials positives o negatives?  Responsabilitat a l’hora de comunicar: no pecar de positivisme perquè no sembli que ja no hi ha problema  Els joves parlen molt de sexe, però parlen de sexe segur? No tabú no vol dir més prevenció | Research needs on reasons why there is a lack of information and on communication coverage and deficits  Research on coverage of prevention measures in communication channels | Quins són els motius pels quals no es facilita més informació sobre la prevenció del VIH/sida i altres ITS a nivell global i amb major abast?  Quina és la presència d’informació sobre prevenció del VIH als espais públics i a d’altres canals? Quins canals utilitzen per informar-se? Quins canals serien els més adequats per fer-los arribar informació? | Anàlisis de la informació, desinformació i preocupacions a les XXSS i a altres entorns.  Anàlisis de la informació que reben els diferents públics (continguts, mètodes i canals) i percentatge que arriba a través de canals com ara els de pornografia entre d’altres on no es fomenta la prevenció del VIH i altres ITSs.  Anàlisis de la presència de missatges de sexe segur en peces de comunicació sobre sexualitat: Anàlisis de la presència de missatges sobre sexe segur en entorns de comunicació que tracten temàtiques de sexualitat (i.e. el sexe està deixant de ser tabú però en quina mesura es combina amb missatges de sexe segur?).  Grau d’informació que arriba a través de canals que no promouen la prevenció |  |
|  |  | Low level of social reflection on sexuality | Low level of social reflection on sexuality |  | No parlem de sexe a nivell de societat – Manca de campanyes comunitàries  Més fàcil tenir relacions sexuals que parlar de sexe  Com més tabú és, menys educació sexual es fa en joves  Els joves parlen molt de sexe, però parlen de sexe segur? No tabú no vol dir més prevenció | Facilitate training for professionals on how to educate about affective-sexual aspects  Facilitate reflection spaces | Crear espais de reflexió/formació amb professionals investigadors i alumnes facilita i millora el coneixement i ajuda a resoldre els dubtes. | Tabú: Com es podria facilitar la reflexió entorn a les relacions sexuals perquè no sigui un tema tabú?  Facilitar recursos per a fomentar la reflexió entorn a les relacions sexuals |  |
|  |  | Lack of awareness of the need to take care of one’s health, including one’s sexual health and of how to prevent HIV and other STIs; low effectiveness of the current pathogenic model | Lack of awareness on need to take care of one’s health  Low effectiveness of the current pathogenic model | Cal més comunicació sobre VIH i ITS no només l’ús del preservatiu per a evitar embarassos.  Problemes per canviar conductes preestablertes | Menysvaloració de les ciències socials vs les ciències biomèdiques  Coneixement molt teòric, desvinculat a l’entorn proper  Relaxació  Ha baixat la guàrdia de la prevenció: com s’augmenta la consciència sense alarmar i sense sensacionalisme?  Responsabilitat a l’hora de comunicar: no pecar de positivisme perquè no sembli que ja no hi ha problema  Adaptació del llenguatge al públic diana | Need to facilitate spaces to promote prevention  Research on perception of need for prevention  Need for interdisciplinary approaches | Afavorir espais i materials per fomentar els hàbits saludables als centres educatius que incloguin la prevenció de les ITS i també d’altres malalties. | Percepció sobre la necessitat de cuidar la salut i prevenir les ITSs: Quina és la percepció de la necessitat de prevenir les ITSs i com hi influencia la comunicació (i.e. el balanç entre notícies positives i negatives que es difonen, la quantitat d’informació, els tipus de continguts....)?  Promoció interdisciplinària de la salut a l’educació formal i no formal: Millorar l’educació en sexualitat saludable i en salut en general a l’educació formal i no formal (presencial i online) de manera que contempli continguts interdisciplinaris que incloguin coneixement en biomedicina, ciències socials, i d’altres disciplines però alhora també competències com ara en el camp de les emocions.  Promoció de la salut entre els joves mitjançant Centres Joves de Salut: Millorar l’educació no formal en sexualitat saludable i en salut en general mitjançant la creació de Centres Joves de Salut (ja existents a Nova York i un a Barcelona i un a Girona). Aquests han de donar una atenció global a la persona per tal d’adaptar-se a les seves necessitats i que cobreixin el buit existent entre pediatria i els 40-50. Cal també que s’apropin allà on ja van aquests públics, com ara centres cívics. |  |
|  |  | Lack of affective-sexual education in formal education, which currently has a very biological approach | Lack of affective-sexual education in formal education  Formal education has a very biological approach  Knowledge on transmission routes, preventive methods, with a good balance between the perception of risk and the focus on pleasure | Important que el professorat sàpiga del tema  Ha ajudat molt fer aquest programa pilot  Facilitar intervencions escolars  Educar en prevenció  Els alumnes de diferents itineraris formatius haurien de tenir tots ells una base formativa mínima per a la prevenció en VIH  -Introduir xerrades obligatòries de prevenció del VIH a les escoles a nivell del 3r d’ESO  Calen més coneixements sobre el VIH i ITS a les escoles.  Que el estudi del SIDA sigui obligatori en els estudis de secundaria .  Incorporació tardana al cuirriculum (ESO3)  Facilitats: Podem modificar el currículum d’ensenyament de forma que integri el millor coneixement de la malaltia.  Obstacles: El temps de dedicació curricular a aquesta malaltia seria igual d’important que la dedicació a altres per fer-la efectiva, i això implica una reducció del currículum habitual establert.  Xerrades de gent infectada.  Que totes les escoles treballin el tema de prevenció del VIH amb els alumnes. També amb les famílies.  Profes poc preparats, valents per tractar del tema amb llibertat. Sobretot a partir de 3r d’ESO.  Que els professors informin sobre vies de transmissió, ja que els alumnes els senten com a persones properes.  Tractar el tema com una assignatura o dins d’una assignatura de malalties de transmissió sexual.  Començar a parlar de manera més general a partir de 1r d’ESO.  Visualitzar vídeos divulgatius.  Buscar temps per parlar del tema com estem fent actualment, per tenir coneixement fiable.  Si s’inclogués al currículum estaria bé, ja que es podria tractar amb el professorat de biología  Falten xerrades de divulgació.  Més iniciatives dins l’horari lectiu com la que estem desenvolupant a través d’aquest projecte. Els currículums extensos, i un sistema massa acotat, no actualitzat, no afavoreix “sortir-se del guió” amb iniciatives com aquesta. Grups classe amb gran nombre d’alumnat. Caldria poder treballar en petits grups per augmentar la implicació de tothom.  Hi ha poc espai temporal que fomenti la prevenció del VIH. Per exemple en l’ús del preservatiu | Manca d’educació sexual  Educació sexual: qui la fa? Com? On?  Manca formació professionals  Manca educació afectivo-sexual  Manca de formació des de la infància primerenca  Manca d’educació  Falta formació de professionals de la salut i educació  Manca d’educació sexual al currículum  Coneixement molt teòric, desvinculat a l’entorn proper, amb poc balanç entre percepció de risc i focus en plaer  Model educatiu poc participatiu: Missatges unidireccionals enlloc de diàlegs/debats  Alfabetització en salut | Need to innovate in sexual education | Quins són els punts de vista dels diferents actors socials en quant a com cobreix el currículum continguts entorn a aquesta malaltia? Anàlisis comparatiu a diferents contextos. Quins són els motius pels quals no es comença l’educació per a la prevenció del VIH i d’altres ITS des de la primera infància?  Nova proposta de currículum desenvolupat amb participació  Desenvolupar protocols, estratègies i materials que facilitin la co-creació i la implementació de les activitats educatives afectiva-sexuals adaptats a cada edat  Educació sexual obligatòria des de la primera infància  Promoure l’educació sexual a les escoles i també dirigida a les famílies  Definir-la amb la participació de diferents actors de diferents nivells educatius  Promoure metodologies didàctiques innovadores que fomentin el treball en grup, l’esperit crític i la participació | Suport social per fomentar l’educació afectiva-sexual: Quines són les percepcions dels diferents actors socials en quant a la necessitat de fomentar l’educació afectiva-sexual?  Quines són les percepcions dels diferents actors socials en quant a com s’hauria de fomentar l’educació afectiva-sexual?  Educació sexual que respon a les necessitats de diferents col·lectius: Com es podria fomentar una educació afectiva-sexual als centres educatius que respongui a les necessitats dels diferents actors? Com podrien ajudar metodologies de participació dels actors en la definició dels programes?  Com podria haver canviat la resposta de les famílies a la decisió del Govern d’instal·lar màquines expenedores de preservatius a les escoles si la decisió s’hagués dut a terme amb mètodes de co-decisió que deixessin més autonomia als centres?  Promoure l’educació afectiva-sexual al currículum  Afegir la salut i l’educació afectiva-sexual al currículum de les escoles començant des d’edats primerenques  Promoure que s’incorporin perspectives de diversitat sexual.  Col·laboració entre la comunitat educativa (departament d’educació, professorat i alumnat) i la comunitat científica (agència de salut pública) i també amb la comunitat. Separar l’educació sexual de les assignatures de ciències i fer-la més transversal al currículum  Estratègia pedagògica innovadora: Educació sexual més interdisciplinària i amb mètodes educatius més innovadors i participatius i continguts més vinculats a l’entorn dels destinataris.  Promoure l’educació afectiva-sexual flexible  Facilitar espais de co-creació en les pràctiques docents de sexualitat per assegurar que s’adapti a les seves necessitats.  Fomentar un bon lideratge polític que faciliti una educació en sexualitat que pugui ser flexible de manera que respongui a les necessitats dels diferents actors mitjançant la seva involucració (i.e. famílies, escola, professorat...)  Formació de professorat  Incloure una Assignatura de salut a primària |  |
|  |  | Lack of affective-sexual education in non-formal education targeting different stakeholders, such as diverse professionals and citizens; it currently has a strong focus on biological education | Lack of affective-sexual education in non-formal education  Non formal education targeting different stakeholders, such as diverse professionals and citizens;  Non-formal education currently has a strong focus on biological education  Lack of training for professionals  Facilitate reflection spaces | Fer més activitats de divulgació científica  Organitzar xerrades  Manca de campanyes de publicitat per conscienciar a la població  Fer arribar també als pares dels alumnes i altres actors socials aquests valors.  Educació de temes sexuals en la família.  Facilitats: La nostre escola és vicenciana i educa en valors als més necessitats i desfavorits. Tenim centres de Obra Social com el de Sta Lluïsa de Marillac on es fa rehabilitació i seguiment a persones sense llar, drogoaddictes etc.-Que els adolescents tinguin clar que hi ha centres on informar-se i fer preguntes.  A tot arreu hauria d’haver un servei d’informació als adolescents, com al centre cívic de Begues  Facilitats: Espais d’interacció (casals de joventut, casal d’estiu...).  Falten punts d’informació per als joves. Hi ha una manca de motivació per instruir als joves.  Fer presentacions per parts dels alumnes oberts a famílies i societat en general.  Buscar espais de xerrades-col·loqui i tallers d’orientació.  Espais d’interacció en hi ha , i es creen tant dins com fora de l’escola: convivències, DOMUND, APS, intercanvi amb alumnes de Guinea Equatorial etc.  Programa que s’aproximés al màxim als adolescents ja des dels 12 o 13 anys, garantint que poguessin demanar ajuda, exposar preocupacions, dubtes…  Programes per a la prevenció simultània de SIDA i MTS. | Manca d’educació sexual  Educació sexual: qui la fa? Com? On?  Manca formació professionals  Manca educació afectivo-sexual  Manca de formació des de la infància primerenca  Manca d’educació  Falta formació de professionals de la salut i educació  Alfabetització en salut, actualment massa focalitzada en aspectes biològics | Training for professionals  Non-formal education | Facilitate reflection spaces  Interventions designed with shared responsibility | Promoure l’educació afectiva-sexual a l’educació informal  Promoure educación no formal perquè pugui ser implementada durant totes les franges d’edat  Formació: per a professionals que treballen amb població diana (i.e. personal assistencial, professionals d’entitats socials, treballadors de lleure, etc.)  Implementar intervencions educatives de salut i salut sexual d’acord amb el Pla Estratègic citat anteriorment  Facilitar unitats des d’on es pugui donar un seguiment a persones que acudeixen al sistema de salut amb algun problema de salut sexual, en especial per a adolescents.  Facilitar espais específics d’atenció a la salut sexual per a adolescents i joves  Facilitar espais que es desenvolupin en col·laboració entre diferents actors socials i iniciatives existents desenvolupades des de la comunitat i des d’altres actors del sistema de salut. |  |
|  |  | Difficulty in transferring knowledge from the scientific community to the media and community agents | Difficulty in transferring knowledge from the scientific community to the media and community agents  Competencies of researchers to increase application of results |  | Manca de comunicació investigadors, tècnics, població...  Dificultats en la transferència de coneixement des dels “experts” als agents comunitaris  Baixa disponibilitat de literatura científica | Bring research and education closer together | Mètodes per apropar la recerca a la societat i en especial a l’educació: Com es podria apropar millor la recerca en prevenció del VIH i d’altres ITS a la ciutadania i en especial al sector educatiu?  Programes de prevenció vinculats amb recerca actual: Facilitar més projectes que fomentin l’apropament de la recerca a la comunitat educativa i a d’altres entorns socials | Adequació dels missatges preventius a les evidències sorgides de la recerca empírica (EX: no totes les pràctiques sexuals tenen el mateix risc però en moltes intervencions es posen al mateix sac) |  |
|  |  | Difficulty for citizens to distinguish between non-rigorous and rigorous information | Difficulty for citizens to distinguish between non-rigorous and rigorous information |  | Baixa disponibilitat de literatura científica  Sensacionalisme  Sensacionalisme és la font de les dades  Interès periodístic vs rigor científic  Adaptació del llenguatge als públics diana |  |  |  |  |
|  |  | Low perception of the risk of contracting HIV and other STIs and their consequences: HIV/AIDS is not fashionable | Low perception of the risk of contracting HIV and other STIs and their consequences: HIV/AIDS is not fashionable | La percepció de la gent sobre el seu coneixement respecte el VIH, no s’ajusta a la realitat: la gran majoria consideren que estan ben informats, però afirmen que l’única via de contagi és la sexual. Per tant, poden mantenir conductes no segures, sense saber-ho. S’estima que la població que te un nivell de coneixement correcte sobre el VIH és d’un 46%. Això fa que la percepció del risc sigui molt baixa en la possibilitat d’adquirir una MTS, a nivell general de la població i en adolescents particularment (alguns abans dels 15 anys ja tenen relacions sexuals i no són conscients dels riscos que hi han davant les MTS i VIH ja que no es perceben com un grup de risc)..  Falsa percepció del risc de contagi  Falta de coneixements i de consciència i  No utilitzar preservatiu o altres mesures preventives a causa de la poca consciència de la seva importància de cara a prevenir malalties  Existeix en la població una percepció baixa de risc davant el contagi caldria fer visible en diferents espais.  Mala percepció del fet d’estar infectat, pel malalt i per l’entorn  Sensibilització sobre la sensació de risc pensant en benefici dels altres  Treballar la percepció de risc.  S’ha minimitzat el risc de contagi de les MTS, i en especial VIH  Manca de consciència de la població. | Baixa percepció de risc en els actes sexuals  - Manca de percepció de risc en adolescents (ITSs)  Baixa percepció de risc en adults (ITSs)  Baixa percepció de gravetat de les malalties (educació en salut general)  Baixa de percepció de la gravetat i de les conseqüències  Conductes de risc  Baixa percepció de risc  Manca de sensació de risc (“per una vegada no passa res”)  El VIH/sida ja no està de moda (en relació a altres causes) | Research on causes of lack of perception of risk | Perquè la gent no pensa en els riscos de contraure el VIH quan manté relacions sexuals sense protecció? |  |  |
|  |  | Communication and education for people living with HIV: lack of knowledge on how to communicate during sexual relationships |  |  | Comunicació en el moment del sexe (VIH+)  REFINEMENT TO: HIV and other STIs |  |  | Guia amb recomanacions sobre com comunicar-se entorn a temàtiques vinculades amb el VIH i altres ITSs en el moment de tenir relacions sexuals i sobre mesures de prevenció i risc | 8.Offer communication and education interventions for people living with STIs on how to communicate during sexual relationships and how to avoid transmitting the infection, especially for high risk communities |
|  |  | Low or no perception of risk by some infected people who do not always take precautions, sometimes because they do not mind transmitting the infection | Some infected people do not mind transmitting the infection  REFINEMENT TO: Low or no perception of risk by some infected people who do not always take precautions, sometimes because they do not mind transmitting the infection |  | Els infectats que no els importa infectar | Interventions to improve perception of risk  Research on risk perception and how to change it | Intervencions per millorar la percepció | Percepció del risc i sobreprotecció dels joves: Com influencia la sobreprotecció que exerceixen les famílies sobre els joves en la percepció del risc?  Quins mecanismes són més efectius per millorar la percepció del risc de contraure ITS i de les seves conseqüències, així com per incrementar la co-responsabilitat en adults / i en adolescents?  Efectivitat d’intervencions persistents i perllongades en el temps  Com es pot millorar la identificació de persones amb conductes de risc repetitives per poder preveure-les? (com obtenir feedback)  Com treballar al voltant d’una sexualitat responsable a nivell individual i col·lectiu |  |
| 4 | Access to prevention, diagnosis, treatment and services | Lack of universal access and use of diagnostic and prevention methods (i.e., tests, condoms, PrEP and PEP*, treatment as prevention, syringes, a vaccine if it becomes effective, etc.) | Lack of universal access and use of access to a vaccine if it becomes effective  Lack of universal access and use of condoms  Lack of universal access and use of syringes | Administrar (de forma general i pública) vacunes preventives si aquestes arriben a ser factibles.  Prohibir la compra d’antiretrovirals per internet, perquè la seva seguretat no està garantida.  Accés a la medicació pertinent.  Accés més fàcil als preservatius.  Distribucíó preservatius.  Fàcil accés al preservatius  Caldria facilitar encara més l’accés als preservatius, tenint en compte especialment els punts de trobada dels adolescents i d’altres grups amb conductes de risc.  Una de les vies de transmissió del VIH és la reutilització d’agulles entre toxicòmans que s’injecten drogues. Per tant, també caldria garantir que això no passés, facilitant la disponibilitat d’agulles en els espais que freqüenten.  La transmissió per transfussions sanguínies i la transmissió vertical (de mare a fill) en els països avançats és pràcticament inexistent a hores d’ara. | Manca d’accés universal  Dificultat d’accés dels joves a recursos de prevenció | Research on determinants for not using prevention methods and tools | Anàlisis dels determinats que duen a la infecció: Quins són els determinants pels quals no s’evita la transmissió?  Anàlisis de l’accés: Com es distribueixen els mètodes de diagnòstic i prevenció? Quin accés hi tenen els diferents públics? Com es podria millorar i garantir l’accés per a tothom? Quines són les perspectives dels diferents actors socials?  Millorar l’accés i fomentar més l’ús dels mètodes de diagnòstic i de prevenció especialment als punts de trobada de grups amb conductes de risc (per exemple espais d’oci i centres educatius, centres cívics, de salut...) i a les poblacions més vulnerables.  Accessibilitat gratuïta i propera: Destinar recursos per a la disponibilitat gratuïta dels diferents mètodes preventius i facilitar-los en espais d’oci i a d’altres espais educatius junt amb informació sobre la diversitat, els avantatges i les habilitats necessàries per fer-los servir | Anàlisis de l’accessibilitat dels mètodes preventius i anticonceptius i recomanacions de millora  Polítiques de millora de l’accessibilitat a la diagnosi  Polítiques de millora de l’accessibilitat als mètodes preventius  Accessibilitat anònima: Facilitar l’accessibilitat anònima al preservatiu per vèncer problemes com la vergonya d’anar a comprar preservatius | 9.Strengthen and improve accessibility to diagnostic and prevention methods in combination with other interventions, both pharmaceutical and non-pharmaceutical (e.g., tests, condoms, PrEP and PEP*, treatment as prevention, syringes, a vaccine if it becomes effective). Accessibility should be improved by: (1) using personalized approaches for people at risk, (2) investing in research and innovation with initiatives such as facilitating spaces for face-to-face and online interactions between different actors (i.e., counsellors, psychologists, teachers, families, young people, citizens, from different social communities and communities at high risk), e.g. by using online tools, such as sexual networks, and (3) focusing on social equity and protection factors for certain vulnerable groups (vulnerability due to factors such as intra-family and institutional violence or disadvantaged socioeconomic situations) in Spain and in countries with low per capita incomes. |
|  |  | Need to improve access through sexual networks that can facilitate the spread of infections but can also be a good tool for prevention | Facilitate interventions in sexual networks | L’aparició del cibersexe constitueix un repte en quant a la prevenció però també una oportunitat doncs permet accedir a la informació d’una forma còmoda i anònima. | Tractaments no arriben  Xarxes socials positives o negatives?  XXSS: Eines que faciliten contactes  Estructura de la xarxa sexual | Research on how to intervene in sexual networks |  | Xarxes sexuals: Quins mecanismes poden ser més efectius per identificar les dinàmiques de les xarxes sexuals per millorar les intervencions?  Com es poden identificar les dinàmiques de les xarxes sexuals de diferents col·lectius?  Intervencions que tinguin en compte les dinàmiques de les xarxes sexuals, i que en alguns casos es duguin a terme a través de les Xarxes Sexuals  Intervencions a les xarxes sexuals |  |
|  |  | Lack of perspective about social equity and protection factors in research projects and prevention interventions | Lack of perspective about social equity and protection factors in research projects and prevention interventions |  | Manca de perspectiva que tingui en compte la diversitat, les desigualtats en salut... Manca de factors protectors  Manca intervencions en poblacions vulnerables  Escassa mirada de gènere i altres eixos d’equitat  Manca de perspectiva d’equitat | Research on how to address inequalities in prevention interventions |  | Metodologies per vèncer desigualtats: Com es poden dissenyar intervencions per a col·lectius vulnerables que siguin constructives i enfocades a l’apoderament i a fomentar factors protectors que minimitzin les connotacions negatives?  Model de prevenció focalitzat en l’equitat social i factors de protecció  Aplicar aquest model en tots els projectes finançats des de l’administració com un requisit previ |  |
|  |  | High cost of preventive methods | High cost of preventive methods  High cost of PEP | Preu dels preservatius  Hi ha baixa disponibilitat de la PEP en la nostra societat per motius econòmics? |  | Research on cost-effectivity decreasing costs of prevention tools | Per què no es redueix el preu dels condons?  Per què els preservatius són tant cars? Per què per nadal no és fa una "cesta económica"de condons?  Per què no es redueix el preu dels fàrmacs?  Quant valen les proves de detecció? Per que no son molt barates  Cost i cobertura per part del sistema de salut: Quins són els motius pels quals l’administració pública del nostre país no cobreix la despesa sanitària de preservatius i altres mètodes com la PrEP i la PeP a nivell ampli (tot i que la PEP es facilita des dels serveis d'urgències, no se li dóna difusió a nivell ampli, i és desconeguda per alguns professionals sanitaris i comunitaris, així com per part de la ciutadania)? Quin balanç hi ha entre cost i efectivitat d’aquestes mesures de prevenció? Com podria ajudar l’accés gratuït a aquests i d’altres mètodes de prevenció?  Quin seria el balanç entre cost i efectivitat de facilitar accés al diagnòstic i als tractaments a nivell global?  Revisar els costos de les alternatives al preservatiu | Baixar el preu del preservatiu |  |
|  |  | Need to strengthen promotion of HIV testing | Lack of universal access and use of diagnostic tests | Hi ha fàcil accés a les proves de detecció?  Més proves de detecció.  Fer que els tests del VIH formin part de les analítiques rutinàries  Accés a salut bàsica per a la realització de les proves de detecció.  S’hauria de regular que la població clau es fes la prova per tal d’ajudar-los  Prova diagnòstica gratuïta mensual.  Podem millorar l’accés a la prova del VHI de forma general i pública, i …  L’altre obstacle seria modificar la salut pública de forma que es fes de manera general i gratuïta la prova del VIH.  Fa falta poder detectar abans el VIH per poder tractar més persones.  Hauria de ser obligatori realitzar les proves del VIH a persones que realitzen pràctiques de risc? (Homosexuals, drogadictes..)  Hauria de ser obligatori realitzar les proves del VIH? (a TOTS) Sí.  Com garantir eines suficientment ràpides per proves i …  Incloure tests en seguiments periòdics  Per què la gent es neguen a fer-se les proves? Proves gratuïtes de detecció del VIH en farmàcies.  Fomentar l’increment de proves entre la població  Incrementar el finançament per fer accessibles les proves | La detecció de les ITS com a oportunitats de prevenció.  Falta d’accés a recursos de detecció en joves  Estudis d’efectivitat de les alertes als metges d’atenció primària perquè fomentin la prova | Research on how to promote universal access  Implementation science to support existing tested approaches | Com es podria normalitzar perquè tothom hi tingui lliure accés? Com s’hauria de distribuir?  Quins serien els punts de vista de diferents actors socials envers a fer la prova en anàlisis rutinàries?  Com es podria conscienciar els diferents grups de persones amb alta probabilitat de contraure el VIH perquè se la fessin més rutinàriament en funció del risc al què s’exposen?  Avaluació d’intervencions per promoure la prova:  Quin és el grau d’efectivitat dels programes que fomenten la prova i els efectes en evitar noves transmissions?  Accés a les proves (i.e. a totes les farmàcies)  També amb les anàlisis rutinàries i en funció del risc al què s’exposen incrementar la periodicitat:  Cost: Proves gratuïtes de detecció del VIH i d’altres ITS | Lliure accés a la prova: Pels casos que no es fan la prova del VIH per manca de privacitat i confidencialitat, o per manca de percepció del risc, com es podria millorar l’accés a través d’internet, com es pot apropar a la comunitat i com millorar els serveis sanitaris per facilitar el diagnòstic amb el màxim de confidencialitat (minimitzar el nombre de visites, repensar el lloc de la visita...-linkage to care)  Prova en anàlisis rutinàries  Cost: Quin hauria de ser el preu d’aquesta prova i en quines condicions hauria d’estar coberta per la sanitat pública?  Estudis d’efectivitat de les alertes als metges d’atenció primària perquè fomentin la prova  Polítiques de promoció de la prova: Està proporcionat l’esforç en la promoció del TASP, els missatges preventius i l’accessibilitat a les proves?  Polítiques: Polítiques de promoció de les proves del VIH i altres ITSs  Intervencions per facilitar l’accés a les proves ràpides de VIH  Promoure l’auto-test i el linkage to care fora dels centres assistencials, a prop de la comunitat (i.e. el test de càrrega viral a domicili i a través d’internet).  Establir protocols perquè es facin alertes al metge d’atenció primària perquè fomenti la prova |  |
|  |  | Need to promote alternative methods to condoms for certain communities, such as PrEP, in combination with non-pharmaceutical interventions | Lack of universal access and use of alternative methods to condoms for certain communities, such as PrEP in combination with non-pharmaceutical interventions | Facilitar l’accés a la prep  Extensió dels tractaments alternatius pre i post exposición per fer-los més accessibles | La PrEP no cobreix la prevenció d’altres ITSs  Cost elevat PrEP  Controvèrsia de si la PrEP ha de ser només per a determinats col·lectius  Reticència de la població general a cobrir les despeses de la PrEP  Demanda de PrEP i on aconseguir trobar-la  Qüestionament social davant de la PrEP: Qui ha de pagar això?  Falten mesures complementàries ja que s’ha estancat el nombre d’infeccions, s’ha arribat al “sostre”.  PreP i altres intervencions educatives: Com complementar la PrEP amb altres intervencions preventives educatives?  Població diana de la PrEP: Com es decideix a qui es facilita la PrEP? Com ens assegurem que arribi als més vulnerables que actualment no estan accedint?  Beneficis afegits de la PrEP: Pot la PrEP ser un ham per accedir al sistema de salut? | Need for personalized approaches to promote access to PrEP for vulnerable communities |  | PrEP i altres intervencions educatives: Com complementar la PrEP amb altres intervencions preventives educatives?  Població diana de la PrEP: Com es decideix a qui es facilita la PrEP? Com ens assegurem que arribi als més vulnerables que actualment no estan accedint?  Beneficis afegits de la PrEP: Pot la PrEP ser un ham per accedir al sistema de salut?  Cost i seguretat de la PrEP: Com es pot facilitar l’accés a tractaments per a la PrEP amb garanties de seguretat i amb preus accessibles?  Controvèrsia entorn al finançament públic per a la PrEP  Quines són els perspectives de la PrEP per part de diferents actors socials?  Com influencien valors de certes religions com ara la catòlica en el suport social a la promoció de la PrEP des del sector públic? (i.e. no hi ha dubte de finançar el tractament del càncer de pulmó però si que es dubta de si finançar PrEP perquè hi ha judici de valor?)  Alternatives a la PrEP: Anàlisis dels obstacles i les oportunitats de la PrEP i d’alternatives a la PrEP com ara intervencions de promoció del preservatiu  Facilitar l’accés a la PEP  Implementació de la PrEP  Promoció de la PrEP i d’intervencions no medicalitzades: Promoure la PrEP en determinats col·lectius en paral·lel amb intervencions comunitàries no medicalitzades*  Definició del públic diana: Promoure processos de participació per decidir qui ha de ser el públic diana  *en vistes a l’efectivitat demostrada en diferents publicacions científiques. L’evidència existent en altres contextos com ara el de l’ús de drogues i la intervenció amb metadona, també demostra que en certs col·lectius cal reconduir la demanda per reduir els danys. |  |
|  |  | Lack of access to prevention for certain vulnerable groups due to various factors (intra-family and institutional violence, disadvantaged socioeconomic situations, etc.) in Spain and in countries with low per capita incomes. | Lack of access to prevention for certain vulnerable groups due to various factors (intra-family and institutional violence, disadvantaged  Focus on social equity and protection factors for certain vulnerable groups in Spain and in countries with low per capita incomes | Molt variable, segons famílies, centres educatius i entorn social.  Per a què una persona prengui decisions  correctes, ha de tenir informació i alhora seguretat en ella mateixa. Persones amb dificultats en el seu entorn, com maltractaments, dificultats econòmiques, etc, poden ser més propenses a descuidar la prevenció.  No tots els països tenen les mateixes oportunitats.  Per què amb l'atur que hi ha, no treballen a l'Àfrica subministrant medicaments?  Hi ha grups socials amb més risc d’infecció?  Un altre obstacle pot ser l’arribada massiva d’una immigració amb pocs recursos.  La legislació i les lluita sanitària existent en relació a la prevenció del VIH al 1r món en clarament diferent a la del 3r món.  Pocs recursos per a la detecció en països pobres  Dificultat per fer arribar els tractaments a la gent sense recursos  La realitat en la nostra societat de 1r mon és molt diferent de la realitat de la resta del món. Per exemple l’accés als tractaments.  En tots els països hauria d’haver el mateix accés a la informació i a la medicació.  Facilitats:- Totes les noves tecnologies faciliten el coneixement, la implicació, la recerca de qüestions i dubtes. Cosa que molts països no disposen. Tampoc disposen d’un entorn social on hi hagi un mínim de garanties de salut, (farmàcies, fàrmacs, hospitals, etc) i hàbits socials saludables. | Manca d’accés a la prevenció per part de certs col·lectius vulnerables per diversos factors (violència intrafamiliar i institucional, situacions socioeconòmiques desafavorides, ...), com ara els joves de centres d’acollida de la Generalitat o els dels Centres de Justícia Juvenils | . | Research on equity in prevention | Joves de centres d’acollida: Quina és la prevalença del VIH entre joves de centres d’acollida de la Generalitat?  Implementar intervencions dirigides a joves de centres d’acollida, als de centres de justífica juvenils i a d’altres col·lectius vulnerables |  |
|  |  | Low use of prevention methods; risks associated with the use of certain contraceptive methods; and early detection for people at risk | Low use of prevention methods | Els joves poc a poc deixen d’utilitzar preservatius. | Baix ús del preservatiu i desconeixement de les raons  Percepció (o no) que el preservatiu disminueix el plaer  Infeccions durant l’embaràs o alletament (transmissió vertical)  Els joves parlen molt de sexe, però parlen de sexe segur? No tabú no vol dir més prevenció | Research on use of prevention methods and the causes | Anàlisis general de l’ús: Quin ús fan els diferents públics dels mètodes de diagnòstic i prevenció? Com evoluciona l’ús dels mètodes preventius en les diferents edats d’una persona?  Ús del preservatiu: Quins són els determinants del baix ús del preservatiu entre diferents col·lectius? Quins mètodes alternatius al preservatiu podrien ajudar a millorar la prevenció del VIH i d’altres ITS?  Ús d’anticonceptius i ITSs: Com influencia l’ús de mètodes anticonceptius que no serveixen com a mesura de prevenció d’ITS en la propagació d’aquestes infeccions? | Anàlisis dels determinants per no usar el preservatiu  Efectivitat de les intervencions de promoció del preservatiu en col·lectius d’alt risc i de risc baix i moderat. Anàlisis dels motius de la baixa inversió en la promoció del preservatiu  Quina és la percepció de que l’ús del preservatiu disminueix el plaer? Quins són els factors que influencien en el plaer sexual i en quina proporció influencia el preservatiu?  Detecció precoç de persones en risc de no fer ús dels mètodes: Com es pot millorar la detecció precoç de les persones en risc (per exemple per evitar transmissió per exemple durant l’embaràs i alletament)? |  |
|  |  | Few spaces for face-to-face and online interaction between different stakeholders (i.e., psychologists, teachers, families, young people, citizens from different social communities and communities with high risk, etc.) | Facilitate spaces for face-to-face and online interactions between different actors (i.e., counsellors, psychologists, teachers, young people) | Treball combinat entre tots els agents educatius (psicòlegs, orientadors  Pocs espais d’interacció amb professionals.  Els professionals de la salut també tenen temps limitat per a accions de divulgació i col·laboració.  S'hauria de facilitar la comunicació alumne-orientador/psicòleg/professor ja que per a compartir una informació tan delicada s'ha de tenir confiança, i això evitaria futurs estigmes. |  | REFINEMENT TO: Facilitate spaces for face-to-face and online interactions between different actors (i.e., counsellors, psychologists, teachers, families, young people, citizens, from different social communities and communities at high risk), e.g. by using online tools, such as sexual networks | Interacció ciutadania-professionals: Quin és el grau d’interacció entre professionals i persones en situació de risc de contraure el VIH?  Efectivitat dels actuals canals d’interacció entre professionals (i.e. professionals de la salut, professorat, orientadors, psicòlegs,...) i ciutadans (persones amb alta probabilitat d’adquirir el VIH, familiars, etc) i entre diferents col·lectius de la ciutadania  Mètodes: Com es podria fomentar una millor interacció?  Foment d’espais d’interacció: Creació i major difusió d’espais d’interacció entre diferents actors socials (professionals, col·lectius de risc, familiars, indústria, investigadors, polítics, comunitat educativa, espais de lleure, entitats societat civil), amb possibilitat de mantenir l’anonimat. | Explorar com facilitar espais col·laboratius entre els professionals sanitaris especialistes en VIH i els d’altres àmbits, amb la comunitat i amb els serveis socials per dur a terme aquestes intervencions. |  |
|  |  | Rejection or lack of adherence to antiretroviral treatment and its impact on transmission risk | Strenghten programmes tarteted at people who reject or do not adhere to antiretroviral treatment |  | Estudi de contactes | REFINMENT TO:  Improve and strengthen programmes targeted at people who reject or do not adhere to antiretroviral treatment, highlighting the impact on the risk of HIV transmission  Disseminate recommendations for professionals on how to intervene in front of people who reject or do not adhere to antiretroviral treatment | Per què la gent no vol seguir el tractament i …?  Percepció de les conseqüències de no medicar-se: Quins són els obstacles i els facilitadors perquè les persones diagnosticades s’adhereixin al tractament correctament? Quins són els determinants per deixar d’acudir als serveis sanitaris?  Mètodes per arribar als que no acudeixen als serveis sanitaris: Com podem tenir un bon control i seguiment d’aquestes persones quan deixen d’acudir als serveis sanitaris? | Intervencions per millorar l’adherència al tractament TAR en persones que viuen amb el VIH: Com donar suport a les persones que viuen amb el VIH per evitar el rebuig i millorar l’adherència i per tant obtenir tant beneficis a nivell individual com per reduir la probabilitat de transmissió del VIH?.  Difusió entre professionals de les recomanacions ja existents sobre com intervenir en casos de persones que conviuen amb el VIH i que no prenen medicació ni precaucions per evitar la transmissió (recollides en algunes publicacions), i reforçar-ho amb recomanacions de fomentar intervencions de pars (també recollides en [algunes publicacions](https://drive.google.com/file/d/0B4x7CNvuMYRUQ2UyVWtoOHY0XzA/view)), i de com dur-les a terme de manera coordinada entre professionals de la salut, de la comunitat i de serveis socials.  Fomentar l’aplicació dels protocols d’estudi de contactes sempre i que es preservin els drets de les persones implicades. | 10.Improve and strengthen programmes targeted at people who reject or do not adherence to antiretroviral treatment, highlighting the impact on the risk of HIV transmission |
| 5 | Psycho-social aspects related to culture, values, stigma and discrimination | Lack of a gender perspective in research projects and prevention interventions | Discrimination for gender  Lack of gender perspective in prevention and research | Treballar des dels centres la igualtat i la no discriminació per visualitzar la diferències de gènere i desestigmatitzar així les conductes sexuals  Desigualtat de sexes i rols predeterminats  La societat sexista (masclista) no ajuda  Les dones tenen una situació desfavorable en la societat per temes d’assetjament sexual.  Tot i l’avanç en la igualtat de gènere, les dones han encara de guanyar poder de decisió en les relacions sexuals, deixant definitivament el rol de satisfer l’home i garantint la seva seguretat envers la transmissió de malalties. | La recerca de gènere no contempla la perspectiva de gènere  Manca de perspectiva de gènere (incloent perspectiva LGTBI) de les polítiques preventives  Desigualtats de gènere  Masculinitats  Escassa mirada de gènere i altres eixos d’equitat | Design and implement policies to address gender inequalities in research and interventions  Analyse how gender and LGTBI inequalities are addressed in research and in prevention interventions  Training |  | Anàlisis sobre perspectiva de gènere  Dissenyar una agenda política per a fer front a les desigualtats socials en els diferents rols de gènere  Dissenyar polítiques per regular que les desigualtats es tinguin en compte en els projectes de recerca i en les intervencions  Promoure programes de formació per eliminar les activitats masclistes  Donar visibilitat a la importància i transcendència de les desigualtats de gènere en les intervencions preventives i de promoció de la salut sexual  Anàlisis de com es té en compte la perspectiva de gènere en els projectes de recerca i en les intervencions de prevenció (incloent també la perspectiva de LGTBI) | 11.Design and implement policies to ensure that all prevention research and interventions address the fight against stigma, discrimination*, violence and taboos while balancing this with the promotion of pleasure, as it is key for effectiveness |
|  |  | Need to consider the prevention of sexual violence in STIs prevention interventions | Need to consider the prevention of sexual violence in STIs prevention interventions |  | Manca tenir en compte la violència LGTBI, sexual i masclista | Analyse inspiring practices that address sexual violence in prevention interventions and how it is being covered  Policies to promote prevention of sexual violence |  | Anàlisis de la prevenció de la violència masclista en les intervencions de prevenció del VIH i altres ITSs i de bones pràctiques d’integració  Polítiques de promoció de la integració de la prevenció de la violència masclista en les intervencions  Polítiques per promoure la prevenció de la violencia masclista des de l’educació formal |  |
|  |  | The high level of stigma and discrimination against people according to their sexual orientation and serological status makes prevention difficult | The high level of stigma and discrimination against people according to their sexual orientation and serological status makes prevention difficult | Normalitzar el poder parlar obertament del tema, que no sigui una cosa especial.  Treure la por sobre el VIH als adolescents i tenir informació real  Exclusió de gent que es considera que pateix conductes de risc (homosexuals i prostitutes).  Prejudicis sobre les persones seropositives  Crear un clima de confiança i acceptació a les persones seropositives  Estigmatitzar les persones portadores no contribueix a la prevenció. Només perjudica als propis afectats.  Exclusió social a qui pateix la malaltia.  En persones ja afectades, l’entorn és un factors important pel que fa a evitar estigmes i fins i tot bulllying. Tractar la malaltia amb normalitat, no implica descuidar la prevenció, sinó el contrari, perquè la gent la considerarà més propera.  Una persona estigmatitzada evitarà parlar-ne i, fins i tot, potser, rebre tractament. Aquesta situació, interfereix clarament en la prevenció. | Homofòbia  Discriminació  Orientació sexual i estigma  Serofòbia  Desigualtats de gènere i masculinitats  Estigmatització  Estigma (col·lectius concrets) | Analyse the determinants and prevalence of stigma and how it affects prevention  Policy innovation and creation of new structures to fight against stigma  Gender perspective | Quin és el grau d’estigma envers les persones que viuen amb el VIH?  Com perjudica les persones afectades i com varia la influència que té en diferents situacions? Com dificulta la prevenció?  Legislació per a la no discriminació de les persones que viuen amb el VIH  Anàlisis de com es té en compte la perspectiva de gènere en els projectes de recerca i en les intervencions de prevenció (incloent també la perspectiva de LGTBI) | Com i quin és el grau de discriminació de les persones segons la seva orientació sexual o estat serològic enfront al VIH o nivell d’addicció a les drogues?  Anàlisis de com afecten les fòbies (serofòbia, LFGTBI fòbia, ...) i la seva interiorització en les estratègies de prevenció del VIH/ITSs  Quins són els determinants de l’estigmatització enfront les persones amb VIH i altres ITSs i com afecta la prevenció?  Desenvolupar polítiques públiques contra l’estigma i la discriminació de les persones que viuen amb el VIH  Facilitar la creació d’estructures dedicades a la lluita contra l’estigma |  |
|  |  | The fight against stigma and discrimination** is not part of the prevention model in a transversal way | Lack of structural interventions to address stigma and discrimination based on serophobia and sexual orientation | Caldria més xerrades a instituts sobre això (estigma).  Treballar sobre l’estigma perquè és normalitzi la vida de la població afectada.  Falta d'implicació pels riscos.  Com afecta la infecció a la vida d’una persona? | Percepció de risc i estigma  A part de les ONGs, falten estratègies per desconstruir l’estigma i apoderar  Manca d’intervencions estructurals (estigma, desigualtats)  Orientació sexual  Homofòbia  Serofòbia  Desigualtats socials  Accessibilitat | Lack of structural interventions to address stigma and discrimination based on serophobia and sexual orientation  REFINEMENT TO: based on sexual orientation or LGBTI phobia, serophobia, social inequalities, accessibility to the health system, gender inequalities and masculinities. | Com es podria evitar l’estigma relacionat amb les persones que conviuen amb el VIH?  Campanyes contra l’estigma i la discriminació | Anàlisi de les estratègies existents per eliminar l’estigma  Com es pot fomentar una sexualitat lliure, oberta i madura que no aporti judici de valor a les persones que contrauen ITSs?  Anàlisis de com l’estigma influencia la percepció del risc  Estratègia per eliminar l’estigma de manera transversal  Campanyes i intervencions estructurals contra l’estigma i les desigualtats socials: Promoure intervencions estructurals per fer front a problemes com ara l’estigma, la discriminació segons l’orientació sexual, la serofòbia, les desigualtats socials, accessibilitat al sistema de salut, la LGTBI fòbia, les desigualtats de gènere i les masculinitats  Desenvolupar polítiques públiques contra l’estigma i la discriminació de les persones que viuen amb el VIH. . Concretar i implementar el Pacte Social contra l’Estigma i la Discriminació contemplat en l’Acord Nacional per fer front al VIH i contra l’estigma relacionat signat per tots els grups polítics en el Parlament de Catalunya l’any 2014 |  |
|  |  | Difficult balance between promoting pleasure and prevention while dealing with stigma | Difficult balance between promoting pleasure and prevention while dealing with stigma |  | Banalització i mercantilització de l’addicció (drogues, sexe, altres...): Decisions poc reflexives en un moment en què els valors socials basats en l’hedonisme, on s’identifica el bé amb el plaer, duen a una mercantilització de recursos de foment del plaer als que costa renunciar, i en què la sexualitat s’ha convertit en un tret identitari |  |  |  |  |
|  |  | Paradox between trivialization and stigmatization: prevention is trivialized, but once the person is addicted to drugs (such as those used in chemsex) or infected, they are discriminated against and stigmatized | Paradox between trivialization and stigmatization: prevention is trivialized, but once the person is addicted to drugs (such as those used in chemsex) or infected, they are discriminated against and stigmatized |  | Estigmatitzar el concepte “prevenció”  Banalització i mercantilització de l’addició (drogues, sexe, altres...) |  |  | Anàlisis de la paradoxa entre la banalització de la prevenció i la discriminació de les persones un cop ja cauen en l’addicció o contrauen la infecció |  |
|  |  | Sex is taboo, there are prejudices and feelings of shame and discrimination, which make prevention difficult | Sex is taboo, there are prejudices and feelings of shame and discrimination, which make prevention difficult | Vergonya a comprar preservatius  Vergonya compra preservatius  Encara és un tema molt tabú  Treballar la Vergonya que fa parlar del tema o fer preguntes.  Tractar els temes tabú i els prejudicis  Hi ha “vergonya generacional” | Dificultat d’accés dels joves a recursos de prevenció degut a l’estigma  Tabú | Analysis of the determinants that make sex a taboo  Research on how taboos affect prevention and how to address it | Sexe tabú, vergonya i dificultat a l’accés: És la sexualitat un tema tabú entre els joves, els adults i els professionals que l’haurien de tractar? Quins són els determinants que duen aquests actors a sentir vergonya per parlar sobre sexualitat? Com influencia la vergonya a la capacitat de demanar accés a mètodes preventius? Com es podria normalitzar la reflexió entorn a la sexualitat? | Barreres i facilitadors perquè els i les joves se sentin còmodes per demanar ajuda o parlar sobre salut sexual? Com fer un canvi transformatiu del model assistencial actual per afavorir-ho? |  |
|  |  | Lack of knowledge about sexual, functional and cultural diversity and its incorporation into research projects and prevention interventions |  |  | Diversitats | Lack of knowledge about sexual, functional and cultural diversity and its incorporation into research projects and prevention interventions  Policies to promote cultural, sexual and functional diversity in research and interventions  Training for professionals |  | Anàlisis de com es fomenta la diversitat en els projectes de recerca i en les intervencions de prevenció (inclusió de dones, persones amb diferents orientacions sexuals, cultures, menors d’edat, persones amb diversitat funcional, persones en risc d’exclusió social, persones amb diferents nivells de recursos econòmics, diferents ètnies, persones que pateixen o estan en risc de violència sexual, masclista etc.)  Anàlisis de la diversitat en els públics objectius: Anàlisis dels grups de població no representats en les polítiques de prevenció  Polítiques de foment de la diversitat sexual en els projectes de recerca i en les intervencions  Polítiques de foment de la diversitat, funcional, sexual i cultural en les intervencions  Formació entorn a diversitat funcional, cultural i sexual per als responsables del disseny d’intervencions  Formació per als professionals de la salut  Polítiques de salut pública dirigides a altres grups de població | 12.Increase literacy about sexual, functional and cultural diversity and how to incorporate them in prevention research and interventions |
|  |  | Stigma can influence changes in values and how sexuality is conceived |  |  | Estigmatitzar el concepte “prevenció” | Stigma can influence changes in values and how sexuality is conceived  Design and implement policies to ensure all prevention research and interventions address changes in values and how sexuality is conceived |  | Estigma i sexualitat: Com ha influenciat l’estigma als canvis de la manera de concebre la sexualitat?  Promoure l’educació i la comunicació que fomentin la reflexió entorn a estigma i valors entorn a la sexualitat  Aplicar el requisit de promoure la reflexió en valors i estigma en tots els projectes finançats des de l’administració com un requisit previ | 13.Design and implement policies to ensure that all prevention research and interventions address cultural aspects, values (e.g., when people get infected it is their own fault due to their immoral behaviour) and the current paradigm shift, including how these values are conditioned by stigma and religions and how they influence prevention plans, conceptions of sexuality, and discrimination against people for sexual, cultural and functional diversities |
|  |  | Rejection of people who become infected, they are considered guilty because of the values associated with certain sexual behaviours conditioned by religions |  | Cultura (religiosa, països del Sud) | Rebuig de les persones que s’infecten per ser considerades culpables pels valors associats a les conductes que les han dut a les infeccions  Model de prevenció condicionat per les religions | Design and implement policies to ensure all prevention research and interventions address changes in values and how sexuality is conceived, including how these values are conditioned by stigma and religions and how they influence prevention plans, conceptions of sexuality, and discrimination against people for sexual, cultural and functional diversities  Policies that promote prevention models not influenced by religions |  | Paradoxa entre un model de sexualitat cada cop més obert i la discriminació que després pateixen les persones amb VIH  Culpabilitat, religions i prevenció: Recerca entorn als valors de culpabilitat de certes conductes sexuals que provenen d’algunes religions.  Com influencien les religions en el model de prevenció  Metodologies: Recerca sobre com promoure una reflexió per evitar l’estigma derivat del valor de culpabilitat.  Com es podria millorar el model de prevenció per fer front als problemes derivats de les influències dels valors promoguts per les religions?  Polítiques que promoguin i defensin explícitament models de prevenció no influenciats per la moralitat i no basats en la culpabilitat i l’aïllament social. Posicionament polític clar i contundent en contra d’aquells models influenciats per les religions (també NO finançament!) |  |
|  |  | Paradigm shift in sexuality regarding culture and values that influences prevention | Paradigm shift in sexuality regarding culture and values that influences prevention | La responsabilitat de les persones, com a valor social, és un factor important per a la prevenció.  La cultura de la població és important, en aquest cas | Increment activitat sexual (amb més parelles i amb ús de drogues)  Conceptualizació del sexe “cultural”  Canvi en el tipus de relacions sexuals  Cultura i ITSs: canvis socials (població immigrant)  Canvi de paradigma de la sexualitat  Els joves parlen molt de sexe, però parlen de sexe segur? No tabú no vol dir més prevenció  Noves tendències en pràctiques sexuals | Analysis of how sexuality is conceived  Training for professionals |  | Com perceben els diferents actors socials i les diferents generacions la sexualitat? Com està canviant aquesta percepció? Quins factors influencien aquests canvis?  Diferencies en comportaments entorn a la prevenció del VIH i altres ITSs en diferents cultures  Diferències en comportaments entorn a la prevenció del VIH i altres ITSs en funció de la concepció de la sexualitat  Formació per a professionals que imparteixen educació sexual i per a professionals de la salut sobre concepció de la sexualitat i canvis en tipus de relacions  (“l’educació sexual ha de contemplar la realitat) |  |
|  |  | Lack of training on values and how they affect informed and thoughtful decision-making for prevention | Lack of training on values |  | Auto-responsabilitat  “Normalitzar” la formació en valors | Lack of training on values and how they affect informed and thoughtful decision-making for prevention | Valors i presa de decisions: Quins són els valors i les opinions de les persones a l’hora de prendre decisions que afecten la prevenció del VIH? Quines reflexions duen a formular aquestes opinions?  Com podríem donar més valor a la responsabilitat personal per millorar la prevenció en la nostra societat?  Actuacions per donar més valor a la responsabilitat a nivell social i a la formació en valors.  Normalitzar la reflexió entorn a la sexualitat amb un balanç equitatiu entre diferents punts de vista  Promoure programes educatius no tant focalitzats en la informació sinó més en la formació en valors |  | 14.Offer training in values involved in informed and thoughtful decision-making for the prevention of HIV and other STIs |
| 6 | Use of psychotropic substances and night leisure | Perceptions about the nightlife model and controversy about how to regulate accessibility to alcohol and other drugs | Controversy about how to regulate accessibility to alcohol and other drugs | Més control sobre l’accés a l’alcohol i estupefaents.  Cal implementar polítiques per a dificultar l’accés a les drogues, especialment a l’alcohol, per la seva  perillositat i bona acceptació social.  Legalitzar les drogues per a poder-hi aplicar major control de seguretat, social i econòmic. | Banalització i mercantilització de l’addició (drogues, sexe, altres...) | Generate evidence on the perception of drug consumption and its association with unsafe sex and group sexual practices  Generate evidence on how to regulate alcohol and other drug consumption | Regulació alcohol i estupefaents: Com s’hauria de regular l’accés i el consum d’alcohol i estupefaents? Podria ser una bona opció repercutir el cost que genera el consum d’alcohol al consumidor? Quins són els riscs i els beneficis de les diferents opcions? Com es perceben per part dels diferents actors? Com es podria fer arribar informació rigorosa d’aquestes opcions?  Com es podria capgirar aquesta bona acceptació social del consum d’alcohol en moltes situacions quotidianes? | Alcohol banalitzat | 15.Promote a participatory process to reflect on nightlife leisure activities and the increased perception of their association with sex, group sexual practices and drug use, including chemsex, the controversy surrounding the regulation of access to alcohol and other drugs, and the group pressure that makes it difficult to make informed and reflective decisions in this leisure environment |
|  |  | The nightlife leisure model and group pressure makes it difficult to make informed and reflective decisions in this leisure environment | Generate evidence on the group pressure that makes it difficult to make informed and reflective decisions in this leisure environment | Hi ha molta informació arreu (internet, campanyes publicitàries en altres mitjans, xerrades a les escoles …). Per tant l’accés al coneixement per a poder prevenir, a priori, hi és. El que obstaculitza als adolescents en la presa de decisions és sobretot la pressió d’altres; per no quedar aïllats del grup, es segueix el corrent.  Pressió social sobre el sexe.  Pressió del grup |  |  |  |  |  |
|  |  | Increase in the perception of a nightlife model associated with sex and group sexual practices and with the use of drugs (i.e., chemsex) | Nightlife model associated with unsafe sex and group sexual practices and with the use of drugs (i.e., chemsex) | Drogues i alcohol, dificulten l’ús del preservatiu.  Efectes indirectes de l’alcohol i les drogues (ITSs)  Millorar sensibilització dels joves en els efectes colaterals de l’alcohol i drogues.  Les xarxes socials són vehicle de molts missatges que inciten el consum d’alcohol i altres substàncies.  Cal augmentar l’esperit crític dels joves i animar-los a què prenguin un paper més actiu mostrant que altres conductes són possibles i millors.  Model d’oci nocturn  També sovint es deixen portar per la impulsivitat i la inconsciència, sobretot si s’ha fet us de substàncies psicotròpiques. | Drogues i ITS (alcohol i cànnabis)  Chemsex  Ús de drogues (legals i il·legals) augmenta el risc  Increment activitat sexual (amb més parelles i amb ús de drogues)  Consum de drogues i HIV/Sida  Chemsex  Drogues  Conductes de risc  Dificultat per abordar nous temes: chemsex – falta de resposta ràpida al problema  Chemsex  Diferències entre consum de drogues problemàtics i no problemàtics | Generate evidence on the perception of drug consumption and its association with unsafe sex and group sexual practices  Training for professionals  Pilot programmes for chemsex | Implementar el programa pilot: Implementació del programa pilot d’atenció integral per al Chemsex | Drogues i prevenció: Anàlisis de la influència del consum de drogues sobre la prevenció del VIH i altres ITSs  Estudiar consum de drogues I com influencia la prevenció diferenciant entorns d’alt, moderat i baix risc  Estudis qualitatius i quantitatius per conèixer bé el chemsex  Com intervenir en persones que deixen de practicar chemsex perquè tornin a tenir relacions sexuals  Facilitar la co-creació i la co-responsabilitat davant problemes emergents com el Chemsex: Facilitar processos de co-creació i co-responsabilitat davant a noves problemàtiques emergents com ara el Chemsex que permetin al sistema de salut donar resposta ràpida i contrastada entre diferents actors socials  Formació sobre ChemSex: Formació per a professionals que imparteixen educació sexual i professionals de la salut  Programa pilot per al Chemsex: Programa pilot d’atenció integral per al Chemsex  Programa pilot per al chemsex amb recomanacions per a experts que imparteixen intervencions de prevenció del VIH i altres ITSs  Programa pilot per al chemsex |  |
| 7 | Assessment of stakeholders’ needs and interventions | Lack of needs analysis to design innovative solutions more aligned with the needs of different communities and more personalized | Lack of needs analysis to design innovative solutions more aligned with the needs of different communities and more personalized |  | Manca d’anàlisis de necessitats per dissenyar solucions  Solucions innovadores més alineades amb les necessitats dels destinataris més, personalitzades | Needs analysis to design innovative solutions more aligned with the needs of different communities and more personalized | Anàlisis de les necessitats de diferents grups poblacionals per al disseny d’intervencions més ajustades a les seves necessitats (per ex. que contemplin que els usuaris de drogues fan pràctiques sexuals) | Anàlisis de necessitats de determinats col·lectius  Difusió de les necessitats: Difusió i formació per a responsables de dissenyar intervencions i professionals de la salut sobre les necessitats de determinats col·lectius  Dissenyar estratègies de prevenció que responguin a les necessitats dels diferents col·lectius (que, per exemple, tenen diferents visions de la sexualitat, diferents percepcions del risc...)  Promoure campanyes de salut personalitzades i adaptades a cada persona (i.e. missatges adaptats a cada persona, amb geolocalització s’envia informació rellevant al moment...)  Promoure campanyes personalitzades sempre que es respecti el dret a la intimitat i a la protecció de dades  Facilitar canals per a diferents franges d’edat | 16.Facilitate resources for needs assessments and evaluations of interventions that demonstrate changes in habits and that improve their design with innovative methods and channels and with more personalization and alignment with the needs of different communities |
|  |  | Analysis of interventions that lead to the promotion of new habits and not so much to specific actions |  |  |  | Analysis of interventions that lead to the promotion of new habits and not so much to specific actions |  | Anàlisis de l’efectivitat en el foment de que l’actitud i la conducta de prevenció vagin alineades, i de com passar de conductes puntuals a hàbits |  |
|  |  | Lack of rigorous evaluation that considers iatrogenesis (i.e., adverse effects and risks associated with an intervention]) to design interventions with more effective methods and channels and to identify the actors to involve | Lack of rigorous evaluation that considers iatrogenesis to design interventions |  | Manca d'una avaluació rigorosa que tingui en compte la iatrogènesis | Evaluation to design interventions with more effective methods and channels and to identify the actors to involve  Systematic review of the evaluations performed in Catalonia | Quines són les característiques dels programes educatius efectius dirigits a diferents públics i que utilitzen diferents canals?  Anàlisis d’efectivitat de diferents campanyes. Quines són les característiques de les campanyes informatives efectives dirigides a diferents públics i que utilitzen diferents canals?  Avaluar els programes educatius | Avaluar les polítiques públiques, i.e. els programes de promoció de la salut sexual a Catalunya  Anàlisis de l’efectivitat de les campanyes informatives i del grau d’adaptació als seus públics objectius  Quins mecanismes poden ser més efectius per millorar el coneixement sobre prevenció?  Anàlisi de l’efectivitat tenint en compte els actors responsables d’impartir l’educació, mètodes, recursos, canals): Quins mecanismes poden ser més efectius per millorar les intervencions educatives de salut i en especial de salut sexual (en quant a responsables, actors que haurien de participar, mètodes, recursos i canals)?  Avaluar la comunicació i els programes educatius: Promoció de l’avaluació de la comunicació i dels programes educatius que arriba als diferents col·lectius  Intervenir: Revisar i intervenir en els canals més consultats | 17.Facilitate access to rigorous and participatory evaluation methods that include the analysis of iatrogenesis, among other aspects, and that also facilitate the identification of stakeholders to involve in co-designing interventions |
|  |  | Lack of rigorous evaluation methods that include the analysis of iatrogenesis |  |  | Lack of rigorous evaluation methods that include the analysis of iatrogenesis |  | Metodologies per analitzar necessitats: Com es podrien dissenyar estratègies de prevenció que responguin a les necessitats dels diferents col·lectius (que, per exemple, tenen diferents visions de la sexualitat, diferents percepcions del risc...)? Com es podria tenir en compte la iatrogenesis?  **Metodologies d’avaluació d’efectivitat:** Anàlisis de mètodes d’avaluació (incloent estudis longitudinals d’impacte) i de bones pràctiques en intervencions de prevenció  Identificació dels factors d’èxit i fracàs. Indicadors per a revisions sistemàtiques. Incloure també criteris mencionats en els diferents punts d’aquesta agenda, com ara els que es mencionen en les seccions de gènere, ètica i legislació, equitat, estigma, percepció del risc....  Revisió sistemàtica de les avaluacions de les intervencions a Catalunya.  **Data science i avaluació**: Com podria el data science millorar els mètodes d’avaluació analitzant dades de salut i intervencions/campanyes?  **Metodologies d’anàlisis de l’eficiència:** Revisió sobre mètodes d’anàlisis econòmics sobre el retorn de la inversió (cost-efectivitat) (es duen a terme molts més estudis d’efectivitat que d’eficiència)  **Metodologies d’avaluació de la iatrogènia:** Anàlisis dels mètodes per avaluar els efectes colaterals o de l’impacte negatiu de determinades polítiques de prevenció (ex. en estudis de contacte s’avalua la seva efectivitat però no es tenen en compte efectes com l’estigma, la confidencialitat, l’angoixa generada, etc.).  **Polítiques de foment de l’avaluació de les intervencions**  Promoció de l’avaluació de les polítiques públiques de prevenció i de les intervencions seguint la guia de recomanacions sobre com avaluar  Fer ús del data science  Incloure avaluació de les necessitats, l’efectivitat, l’eficàcia i la iatrogènia.  Incorporar l’avaluació a totes les actuacions, tant en intervencions com en campanyes.  **Potenciar proves pilot**  **Segell de qualitat:** Crear un segell de qualitat de les campanyes i intervencions (l’ajuntament de Barcelona hi està treballant) |  |  |
| 8 | Development and innovation of biomedical tools for prevention and diagnosis | Vaccine |  |  |  | Research on the development of a vaccine | Cal trobar una vacuna preventiva. Es pot prevenir amb algun fàrmac? | Desenvolupament de vacunes preventives  Percepció i ús que se’n farà: anticipació de possibles reticències al seu ús i identificació de línies d’actuació a implementar per promoure’n el seu ús | 18.Invest in R&I to develop vaccines, microbicides and other pharmaceutical treatments to reduce STIs in the community |
|  |  | Microbicides |  |  |  | Research on microbicides |  | Desenvolupament de microbicides (per VIH i ITSs) I altres solucions que siguin més equitatives i sostenibles |  |
|  |  | Treatments to reduce STIs in the community | Treatments to reduce STIs in the community |  | Desenvolupament de nous fàrmacs que permetin disminuir la dosis i la freqüència d’administració  Desenvolupament de fàrmacs que també cobreixin altres ITSs a part del VIH | Research on treatments to reduce STIs in the community |  | Com podem disminuir la incidència /prevalença d’ITSs a la comunitat (càrrega d’ITSs comunitària)  Monitorització dels possibles efectes secundaris de la PrEP (especialment en casos de PrEP informal) |  |
