# Supplementary material for: A systemic, multistakeholder participatory approach to research, innovation and action agenda setting for more integrated and effective prevention of HIV and other STIs: the case of Catalonia, Spain
Source: Front Public Health. 2026 Sep 23;14:1783030. doi: 10.3389/fpubh.2026.1783030 (PMC13644724; doi:10.3389/fpubh.2026.1783030)
Supplement: Supplementary file 5 [file Supplementary_file_5.docx]

Additional file 5. Phase 4: Priority-setting phase

*Method*

Phase 4 involved prioritizing topics in the preliminary agenda. Instead of inviting each stakeholder group to prioritize only the topics they had listed themselves, we allowed them to set priorities among all the topics, including those suggested by other stakeholder groups. This way they could reflect on a broader set of solutions, and thus their priorities would more likely cover broader barriers in different parts of the system. The professional and master students participated in this process. However, the secondary school students could not participate, because it was not possible to contact them after their academic course had finished. Priority setting was performed between December 2017 and February 2018.

We asked the professionals and master students who had participated in the previous phases to identify 15 priorities on the shared draft agenda. The priority setting was conducted through an online consultation. A total of 19 people participated (5 professionals from CSOs, 10 academic professionals from the disciplines identified in the first phase, 2 professionals in health science communication and education and 2 master students). Finally, the topics with nine or more votes were prioritized. Participants validated the priority setting during the dialogue meeting.

*Results*

The priority-setting phase concluded with 10 prioritized solutions (see table below). Most of these focused on Missions 3 and 4, on accessibility to information, education and communication and on diagnosis, treatment and prevention tools and services, respectively, (five priorities) and Mission 5, stigma and discrimination (two priorities). The remaining three priorities were distributed among Missions 2, 5 and 7 on the model of governance, cultural values and beliefs and evaluation, respectively (one priority in each Mission).

The diversity of stakeholders voting on the priority solutions was always higher or equal to the diversity of stakeholders who had suggested them. For instance, the professionals on “biomedical prevention and barrier methods” did not list any need to improve how the implementation of current solutions were evaluated. However, three professionals from this group voted for this solution as one of the priorities. Another example is a solution on the need to promote alternative prevention methods to condoms for certain communities. Only professionals in “communication and education” and “behavioural and structural aspects” had suggested it, but then four CSO professionals and one expert on “biomedical prevention” voted for it as a priority. As can be seen in the figure below, this was also the case for the topics on gender, risk perception and sexual and reproductive rights: stakeholders who had not raised issues about these topics then considered them as priorities.

**Figure.** Graphic showing issues receiving the most votes (≥ 9 votes), comparing the variety of stakeholder groups suggesting them versus those voting for them.

Some solutions were considered a high priority by only one stakeholder group and thus were not prioritized. For instance, the CSOs proposed a topic on the lack of balance between biomedical and socio-educational prevention models, claiming that this leads to a lack of attention to social, ethical, equity and human rights issues. Other solutions were not prioritized at all, such as biomedical solutions. During the dialogue meeting, Participant 1 questioned how some stakeholders would interpret the fact that topics such as the development of a vaccine were not prioritized. The first author answered that it seems that participants prioritized by sense of urgency, and they felt there is already investment from other sources in this line of research.

During the dialogue meeting, attendees also highlighted the high degree of consensus in priority setting between the different stakeholder groups. As Participant 2 mentioned, “It was surprising to see that the priority setting was quite similar among all actors”.

**Table B1: Top 10 priorities in the agenda**

|  | **Missions** | **Themes of barriers and needs** | **Examples of research lines** | **Examples of innovation solutions & actions** |
| --- | --- | --- | --- | --- |
| 1 | Mission 2: Governance, regulation and human rights | Need to consider sexual and reproductive rights in a transversal way in all prevention actions | Analysis of the promotion or not of those rights | Policies to promote these rights |
| 2 | Mission 2: Governance, regulation and human rights | Co-responsibility model for decision-making. It is necessary to change the model of interaction between different actors from a communication model towards a co-responsibility model in which prevention is a shared project | Research on how to promote a transformative change in the governance of prevention  Research on how to set up R&I priorities through participatory processes  Methods to agree on prevention methods for use among different actors  Evaluation of the impact of the change of governance model | Participatory R&I agenda setting  Methods to promote participation in decision-making  Guidelines for impact evaluation  Training for professionals |
| 3 | Mission 3: Information, education and communication | Low perception of the risk of contracting HIV and other STIs and their consequences: HIV/AIDS is not fashionable | Analysis on perception of risk, including interactions with drugs and alcohol | Improvement of prevention messages to better adjust them to real risks |
| 4 | Mission 3: Information, education and communication | Lack of affective-sexual education in formal education, which currently has a very biological approach | Research on social perceptions and motivations to promote sexual-affective education  Research on how to promote this education, responding to the needs of the different stakeholders | New curricula that is co-created and includes sexual education  Protocols, strategies and materials to co-create and implement flexible and innovative educational interventions on sexual-affective education  Promote it in formal education, for all ages, and also for families  Training for teachers |
| 5 | Mission 4: Access to prevention, diagnosis, treatment and services | Need to strengthen promotion of HIV testing | Determinants to get tested or not for HIV  Impact of free access to HIV testing  Impact of including the HIV test in ordinary tests  Cost-efficacy issues  Evaluation | Public policies  Interventions to promote access to HIV testing |
| 6 | Mission 4: Access to prevention, diagnosis, treatment and services | Need to promote alternative methods to the condom for certain communities, such as the Pre-Exposure Prophylaxis (PrEP), in combination with non-pharmaceutical interventions | Study of participatory methodologies to decide the targets of PrEP, to ensure it is accessible for the most vulnerable communities and to analyse if it can be a facilitator to accessing the health system | Promote PrEP with participatory methodologies to define the targets |
| 7 | Mission 5: Psycho-social aspects related to culture, values and stigma and discrimination | The high level of stigma and discrimination against people according to their sexual orientation and serological status makes prevention difficult | General analysis of the degree of stigma* and how it affects prevention  *Including discrimination, prejudices, taboos, fears, shame and their consequences on social and sexual behaviours due to different causes of inequality such as gender, seroconversion, sexuality, etc. | Public policies  Create structures  Periodic report on level of stigma |
| 8 | Mission 5: Psycho-social aspects related to culture, values and stigma and discrimination | Lack of gender perspective in research projects and prevention interventions | Analysis on gender perspectives | Instrument to analyse how gender equality is taken into account  Policy Agenda  Training programmes  Awareness |
| 9 | Mission 5: Psycho-social aspects related to culture, values and stigma and discrimination | Paradigm shift in sexuality regarding culture and values that influences prevention | Analysis of different stakeholders’ perceptions of sexuality and prevention behaviours | Training for healthcare providers and non-formal and formal educators on current trends |
| 10 | Mission 7: Assessment of stakeholders’ needs and interventions | Lack of rigorous evaluation methods that include the analysis of iatrogenesis [ i.e., the adverse effects and risks associated with an intervention] | Methods to evaluate effectivity  Data science and evaluation  Methods to analyse iatrogenic effects | Public policies to promote evaluation  Promotion of pilot evaluations  Quality accreditation |
