# Supplementary material for: A systemic, multistakeholder participatory approach to research, innovation and action agenda setting for more integrated and effective prevention of HIV and other STIs: the case of Catalonia, Spain
Source: Front Public Health. 2026 Sep 23;14:1783030. doi: 10.3389/fpubh.2026.1783030 (PMC13644724; doi:10.3389/fpubh.2026.1783030)
Supplement: Supplementary file 6 [file Supplementary_file_6.docx]

***Additional file 6. Final agenda with themes of needs and barriers and themes of solutions within 8 missions***

|  | **Missions** | **Themes of needs and barriers** | **Themes of solutions** (including R&I and actions in relation to prevention strategies, healthcare access, community-based activities and policy development) |
| --- | --- | --- | --- |
| 1 | Resources for Research and Innovation (R&I), prevention interventions and health inequalities | Lack of funding for the implementation of HIV- and other STI-prevention interventions (lack of human, structural and economic resources) | 1.Better balance between biomedical and socio-educational models with more human, structural and economic resources for communication and educational interventions, for research and rigorous evaluation (qualitative and longitudinal), for early detection and control of STIs and for complementary services, such as social services, especially focused on vulnerable communities |
|  |  | Lack of resources for R&I |  |
|  |  | Lack of resources for communication and educational interventions, as priority is given to medicalized interventions that respond to industry interests (pharmaceutical, alcohol, drugs, etc.) |  |
|  |  | Lack of resources for early detection and control of STIs and for social services |  |
|  |  | Lack of resources for rigorous evaluations (qualitative and longitudinal) |  |
|  |  | Lack of non-precarious resources to change inequalities, with political support for the most vulnerable sectors |  |
|  |  | Urgent need to review the financing model for interventions, which must be co-defined between administrations and third sector entities | 2.Redesign of the funding model for interventions using a participatory process that includes administration and the third sector |
|  | **Total for Mission 1** | **7** | **2** |
| 2 | Governance, regulation and human rights | Need to consider sexual and reproductive rights in a transversal way in all prevention actions | 3.Raise awareness and facilitate open discussions on sexual and reproductive rights and other human rights to improve their coverage in all prevention actions and research projects, to avoid controversies regarding whether it is necessary to define new specific legislations for people living with STIs and to clarify when health professionals can bypass the right to confidentiality because of HIV transmission risk |
|  |  | Need to disseminate peoples’ rights and duties regarding topics related to HIV and other STIs and the reasons why it is not necessary to define new specific legislation |  |
|  |  | Clarify the rights and duties of health professionals when they bypass the right to confidentiality in order to comprehensively address an HIV transmission risk |  |
|  |  | There is a lack of effort to preserve ethical aspects in research projects, especially regarding the rights of volunteers |  |
|  |  | Prevention model isolated from the cascade of health services for people living with HIV | 4.Redesign the prevention model to make it more focused on health promotion rather than disease prevention, more integrated (i.e., with other health problems and healthcare services, with social services from different geographical areas) and more focused on ethics, human rights and social aspects, such as inequalities. The governance of the new model should be based on co-responsibility in decision-making (e.g., on R&I priorities, priority target groups, design and implementation of interventions) including the participation of diverse stakeholders (i.e. policy makers, healthcare professionals, researchers from different disciplines, social services and the social community), should use innovative relational dynamics with a special focus on dealing with power relations |
|  |  | Prevention model based on biomedical interventions without sufficiently addressing ethical issues, human rights and social aspects, such as inequalities |  |
|  |  | Prevention model conditioned by a non-collaborative economic model |  |
|  |  | Co-responsibility model in decision-making (e.g., shared decision-making for research and innovation priorities and priority target groups): It is necessary to change the model of interaction between different actors in the biomedical community (healthcare professionals, researchers from different specialities), the social services, the social community and others using innovative relational dynamics currently based on a communication model, to evolve towards a co-responsibility model in which prevention is a shared project |  |
|  |  | Need for a model of shared responsibility for the design and implementation of actions that are least fragmented, have a better relationship between policy, research and application, and include greater participation of the social community |  |
|  |  | Difficulty for some groups to participate due to power relations |  |
|  |  | Interventions are fragmented among certain health problems, diseases and geographical areas and focus on diseases rather than health promotion |  |
|  |  | Prevention model too influenced by HIV |  |
|  |  | Need to improve the doctor-patient relationship: the patient perceives the doctor as someone who is paternalistic, solves problems, does not empathize, blames them and does not understand the new paradigm of sexuality | 5.Improve the doctor-patient relationship: the patient currently perceives the doctor as someone who is paternalistic, solves problems, does not empathize, blames them and does not understand the new paradigm of sexuality |
|  | **Total for Mission 2** | **13** | **3** |
| 3 | Information, education and communication | Low knowledge on HIV and STIs as well as on transmission routes, preventive methods, including alternative methods such as PrEP*, the consequences of infections and the need to take care of one’s health, including one’s sexual health | 6.Increase literacy on STIs with quantity and quality of information and with (non-)formal education interventions that use a salutogenesis approach, make use of different channels, facilitate reflection spaces, bring research and education closer together and target both different audiences with personalized approaches and the general public to increase:  - Knowledge on transmission routes, preventive methods (including alternative methods such as PrEP*), the consequences of infections and the need to take care of one’s health (including but not limited to one’s sexual health, wrong information that acts as barriers to getting tested, sexuality with a good balance between the perception of risk and the focus on pleasure)   - Competencies of researchers to increase application of results, of professionals to promote affective-sexual education, and of all stakeholders to distinguish between non-rigorous information and rigorous and contrasted information   7.Facilitate training for professionals on how to educate about affective-sexual aspects (currently, they mostly focus on biological aspects) |
|  |  | Perception and wrong information about HIV and other STIs as a barrier to getting tested |  |
|  |  | Need to improve the quantity and quality of information and education interventions: making use of different channels, bringing research and education closer together, aiming at different audiences with personalized approaches and, combined with interventions, targeting more general audiences and focusing less on preventing risk and more on promoting pleasure |  |
|  |  | Low level of social reflection on sexuality |  |
|  |  | Lack of awareness of the need to take care of one’s health, including one’s sexual health and of how to prevent HIV and other STIs; low effectiveness of the current pathogenic model |  |
|  |  | Lack of affective-sexual education in formal education, which currently has a very biological approach |  |
|  |  | Lack of affective-sexual education in non-formal education targeting different stakeholders, such as diverse professionals and citizens; it currently has a strong focus on biological education |  |
|  |  | Difficulty in transferring knowledge from the scientific community to the media and community agents |  |
|  |  | Difficulty for citizens to distinguish between non-rigorous and rigorous information |  |
|  |  | Low perception of the risk of contracting HIV and other STIs and their consequences: HIV/AIDS is not fashionable |  |
|  |  | Communication and education for people living with HIV: lack of knowledge on how to communicate during sexual relationships | 8.Offer communication and education interventions for people living with STIs on how to communicate during sexual relationships and how to avoid transmitting the infection, especially for high risk communities |
|  |  | Low or no perception of risk by some infected people who do not always take precautions, sometimes because they do not mind transmitting the infection |  |
|  | **Total for Mission 3** | **13** | **3** |
| 4 | Access to prevention, diagnosis, treatment and services | Lack of universal access and use of diagnostic and prevention methods (i.e., tests, condoms, PrEP and PEP*, treatment as prevention, syringes, a vaccine if it becomes effective, etc.) | 9.Strengthen and improve accessibility to diagnostic and prevention methods in combination with other interventions, both pharmaceutical and non-pharmaceutical (e.g., tests, condoms, PrEP and PEP*, treatment as prevention, syringes, a vaccine if it becomes effective). Accessibility should be improved by: (1) using personalized approaches for people at risk, (2) investing in research and innovation with initiatives such as facilitating spaces for face-to-face and online interactions between different actors (i.e., counsellors, psychologists, teachers, families, young people, citizens, from different social communities and communities at high risk), e.g. by using online tools, such as sexual networks, and (3) focusing on social equity and protection factors for certain vulnerable groups (vulnerability due to factors such as intra-family and institutional violence or disadvantaged socioeconomic situations) in Spain and in countries with low per capita incomes. |
|  |  | Need to improve access through sexual networks that can facilitate the spread of infections but can also be a good tool for prevention |  |
|  |  | Lack of perspective about social equity and protection factors in research projects and prevention interventions |  |
|  |  | High cost of preventive methods |  |
|  |  | Need to strengthen promotion of HIV testing |  |
|  |  | Need to promote alternative methods to condoms for certain communities, such as PrEP, in combination with non-pharmaceutical interventions |  |
|  |  | Lack of access to prevention for certain vulnerable groups due to various factors (intra-family and institutional violence, disadvantaged socioeconomic situations, etc.) in Spain and in countries with low per capita incomes. |  |
|  |  | Low use of prevention methods; risks associated with the use of certain contraceptive methods; and early detection for people at risk |  |
|  |  | Few spaces for face-to-face and online interaction between different stakeholders (i.e., psychologists, teachers, families, young people, citizens from different social communities and communities with high risk, etc.) |  |
|  |  | Rejection or lack of adherence to antiretroviral treatment and its impact on transmission risk | 10.Improve and strengthen programmes targeted at people who reject or do not adherence to antiretroviral treatment, highlighting the impact on the risk of HIV transmission |
|  | **Total for Mission 4** | **10** | **2** |
| 5 | Psycho-social aspects related to culture, values, stigma and discrimination | Lack of a gender perspective in research projects and prevention interventions | 11.Design and implement policies to ensure that all prevention research and interventions address the fight against stigma, discrimination*, violence and taboos while balancing this with the promotion of pleasure, as it is key for effectiveness |
|  |  | Need to consider the prevention of sexual violence in STIs prevention interventions |  |
|  |  | The high level of stigma and discrimination against people according to their sexual orientation and serological status makes prevention difficult |  |
|  |  | The fight against stigma and discrimination** is not part of the prevention model in a transversal way |  |
|  |  | Difficult balance between promoting pleasure and prevention while dealing with stigma |  |
|  |  | Paradox between trivialization and stigmatization: prevention is trivialized, but once the person is addicted to drugs (such as those used in chemsex) or infected, they are discriminated against and stigmatized |  |
|  |  | Sex is taboo, there are prejudices and feelings of shame and discrimination, which make prevention difficult |  |
|  |  | Lack of knowledge about sexual, functional and cultural diversity and its incorporation into research projects and prevention interventions | 12.Increase literacy about sexual, functional and cultural diversity and how to incorporate them in prevention research and interventions |
|  |  | Stigma can influence changes in values and how sexuality is conceived | 13.Design and implement policies to ensure that all prevention research and interventions address cultural aspects, values (e.g., when people get infected it is their own fault due to their immoral behaviour) and the current paradigm shift, including how these values are conditioned by stigma and religions and how they influence prevention plans, conceptions of sexuality, and discrimination against people for sexual, cultural and functional diversities |
|  |  | Rejection of people who become infected, they are considered guilty because of the values religions associate with certain sexual behaviours  because of values associated with certain sexual behaviours conditioned by religions |  |
|  |  | Paradigm shift in sexuality regarding culture and values that influences prevention |  |
|  |  | Lack of training on values and how they affect informed and thoughtful decision-making for prevention | 14.Offer training in values involved in informed and thoughtful decision-making for the prevention of HIV and other STIs |
|  | **Total for Mission 5** | **12** | **4** |
| 6 | Use of psychotropic substances and night leisure | Perceptions about the nightlife model and controversy about how to regulate accessibility to alcohol and other drugs | 15.Promote a participatory process to reflect on nightlife leisure activities and the increased perception of their association with sex, group sexual practices and drug use, including chemsex, the controversy surrounding the regulation of access to alcohol and other drugs, and the group pressure that makes it difficult to make informed and reflective decisions in this leisure environment |
|  |  | The nightlife leisure model and group pressure makes it difficult to make informed and reflective decisions in this leisure environment |  |
|  |  | Increase in the perception of a nightlife model associated with sex and group sexual practices and with the use of drugs (i.e., chemsex) |  |
|  | **Total for Mission 6** | **3** | **1** |
| **7** | Assessment of stakeholders’ needs and interventions | Lack of needs analysis to design innovative solutions more aligned with the needs of different communities and more personalized | 16.Facilitate resources for needs assessments and evaluations of interventions that demonstrate changes in habits and that improve their design with innovative methods and channels and with more personalization and alignment with the needs of different communities |
|  |  | Analysis of interventions that lead to the promotion of new habits and not so much to specific actions |  |
|  |  | Lack of rigorous evaluation that considers iatrogenesis (i.e., adverse effects and risks associated with an intervention]) to design interventions with more effective methods and channels and to identify the actors to involve | 17.Facilitate access to rigorous and participatory evaluation methods that include the analysis of iatrogenesis, among other aspects, and that also facilitate the identification of stakeholders to involve in co-designing interventions |
|  |  | Lack of rigorous evaluation methods that include the analysis of iatrogenesis |  |
|  | **Total for Mission 7** | **4** | **2** |
| 8 | Development and innovation of biomedical tools for prevention and diagnosis | Vaccine | 18.Invest in R&I to develop vaccines, microbicides and other pharmaceutical treatments to reduce STIs in the community |
|  |  | Microbicides |  |
|  |  | Treatments to reduce STIs in the community |  |
|  | **Total for Mission 8** | **3** | **1** |
|  | **TOTAL** | **65** | **18** |

*PrEP (Pre-Exposure Prophylaxis) and PEP (Post-Exposure Prophylaxis) are drug treatments to prevent HIV.

**According to sexual orientation or LGTBI-phobia, serophobia, social inequalities, accessibility to the health system, gender inequalities and masculinities.
